# Supplementary material for: Cystinosin regulates Na+/H+ exchanger 3 trafficking and function in kidney proximal tubular cells
Source: EMBO Rep. 2026 Mar 24;27(8):2088–117. doi: 10.1038/s44319-026-00736-1 (PMC13121807; doi:10.1038/s44319-026-00736-1)
Supplement: Supplementary file 3 — Movie EV2 [file 44319_2026_736_MOESM3_ESM.zip › Movie EV2 Figure Legend.rtf]

READ ME:Movie EV2: Video showing GFP-tagged NHE3 trafficking in cystinosis patient (CT) PTCs using pseudo-Total Internal Reflection Fluorescence microscopy (pTIRFM).
