# Supplementary material for: Cystinosin regulates Na+/H+ exchanger 3 trafficking and function in kidney proximal tubular cells
Source: EMBO Rep. 2026 Mar 24;27(8):2088–117. doi: 10.1038/s44319-026-00736-1 (PMC13121807; doi:10.1038/s44319-026-00736-1)
Supplement: Supplementary file 4 — Movie EV3 [file 44319_2026_736_MOESM4_ESM.zip › Movie EV3 Figure Legend .rtf]

READ ME:Movie EV3: Video showing GFP-tagged NHE3 trafficking in CT cells transduced with LV-CTNS-DsRed using pseudo-Total Internal Reflection Fluorescence microscopy (pTIRFM).
