# Supplementary figures and images for: Cystinosin regulates Na+/H+ exchanger 3 trafficking and function in kidney proximal tubular cells (part 1 of 2)
Source: EMBO Rep. 2026 Mar 24;27(8):2088–117. doi: 10.1038/s44319-026-00736-1 (PMC13121807; doi:10.1038/s44319-026-00736-1)

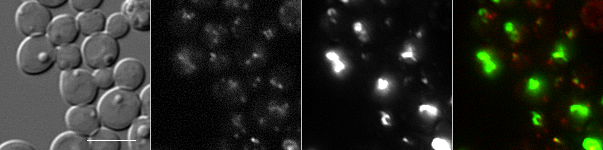

Supplement: Supplementary file 6 — Source data Fig. 1 [file 44319_2026_736_MOESM6_ESM.zip › Figure 1/1A/Bottom.tif]

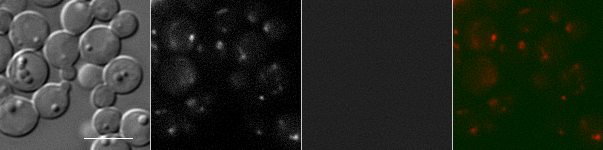

Supplement: Supplementary file 6 — Source data Fig. 1 [file 44319_2026_736_MOESM6_ESM.zip › Figure 1/1A/Middle.tif]

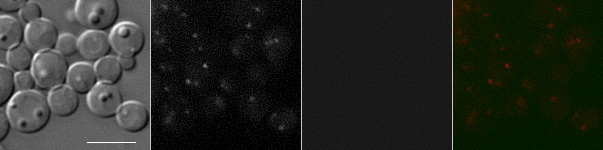

Supplement: Supplementary file 6 — Source data Fig. 1 [file 44319_2026_736_MOESM6_ESM.zip › Figure 1/1A/Top.tif]

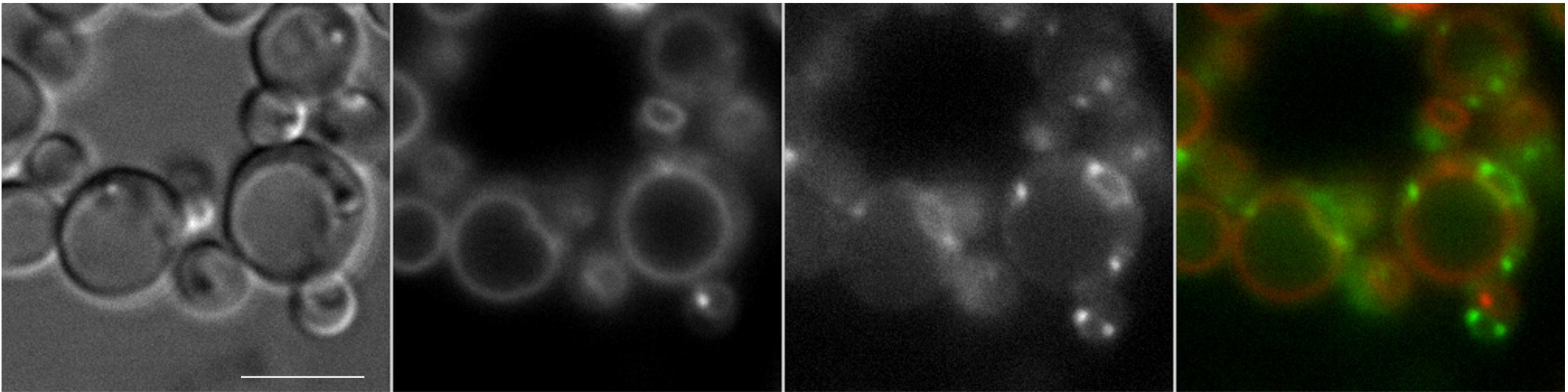

Supplement: Supplementary file 6 — Source data Fig. 1 [file 44319_2026_736_MOESM6_ESM.zip › Figure 1/1B/Bottom Panel.jpg]

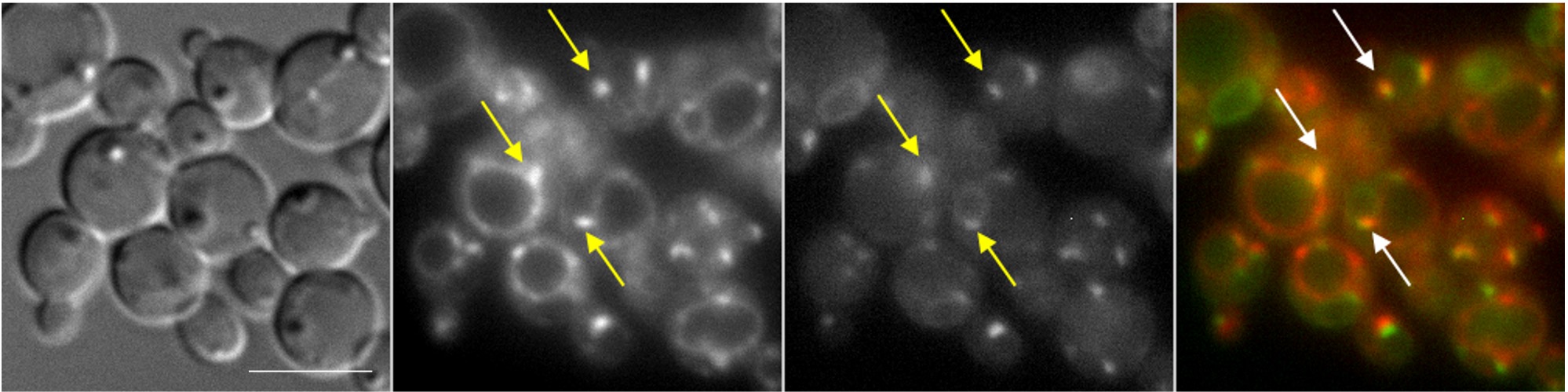

Supplement: Supplementary file 6 — Source data Fig. 1 [file 44319_2026_736_MOESM6_ESM.zip › Figure 1/1B/Top Panel.jpg]

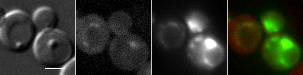

Supplement: Supplementary file 6 — Source data Fig. 1 [file 44319_2026_736_MOESM6_ESM.zip › Figure 1/1D/Dvps1.tif]

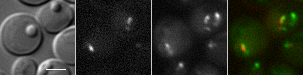

Supplement: Supplementary file 6 — Source data Fig. 1 [file 44319_2026_736_MOESM6_ESM.zip › Figure 1/1D/Dvps15.tif]

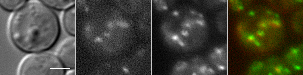

Supplement: Supplementary file 6 — Source data Fig. 1 [file 44319_2026_736_MOESM6_ESM.zip › Figure 1/1D/Dypt7.tif]

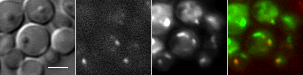

Supplement: Supplementary file 6 — Source data Fig. 1 [file 44319_2026_736_MOESM6_ESM.zip › Figure 1/1C/Ders1S.tif]

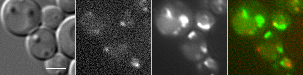

Supplement: Supplementary file 6 — Source data Fig. 1 [file 44319_2026_736_MOESM6_ESM.zip › Figure 1/1C/Ders1s Ders1L.tif]

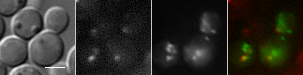

Supplement: Supplementary file 6 — Source data Fig. 1 [file 44319_2026_736_MOESM6_ESM.zip › Figure 1/1C/Ders1L.tif]

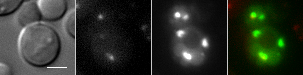

Supplement: Supplementary file 6 — Source data Fig. 1 [file 44319_2026_736_MOESM6_ESM.zip › Figure 1/1C/WT.tif]

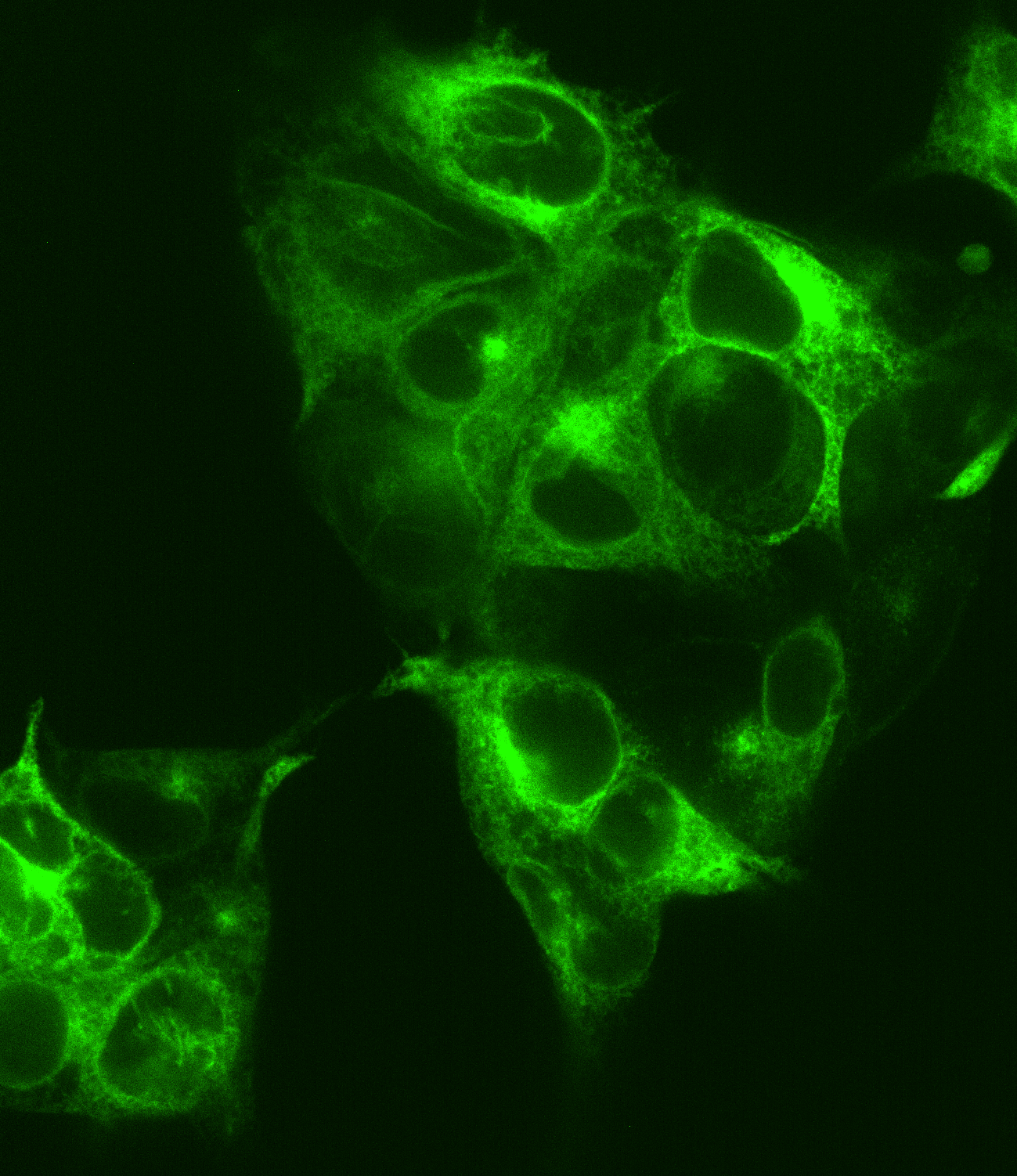

Supplement: Supplementary file 7 — Source data Fig. 2 [file 44319_2026_736_MOESM7_ESM.zip › Figure 2/2B Top panel_Replicate/NHE3 GFP.tif]

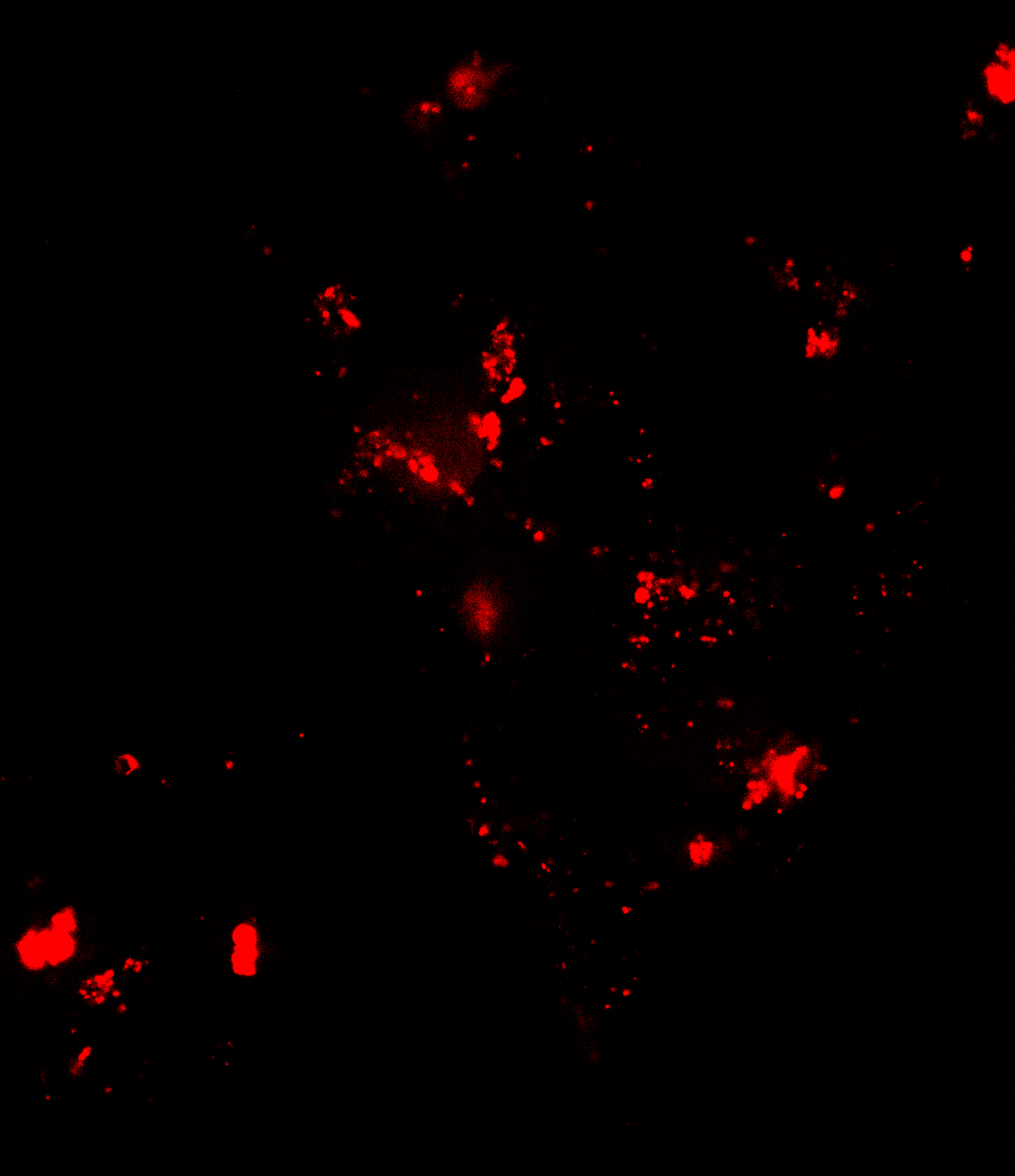

Supplement: Supplementary file 7 — Source data Fig. 2 [file 44319_2026_736_MOESM7_ESM.zip › Figure 2/2B Top panel_Replicate/CTNS DsRed.tif]

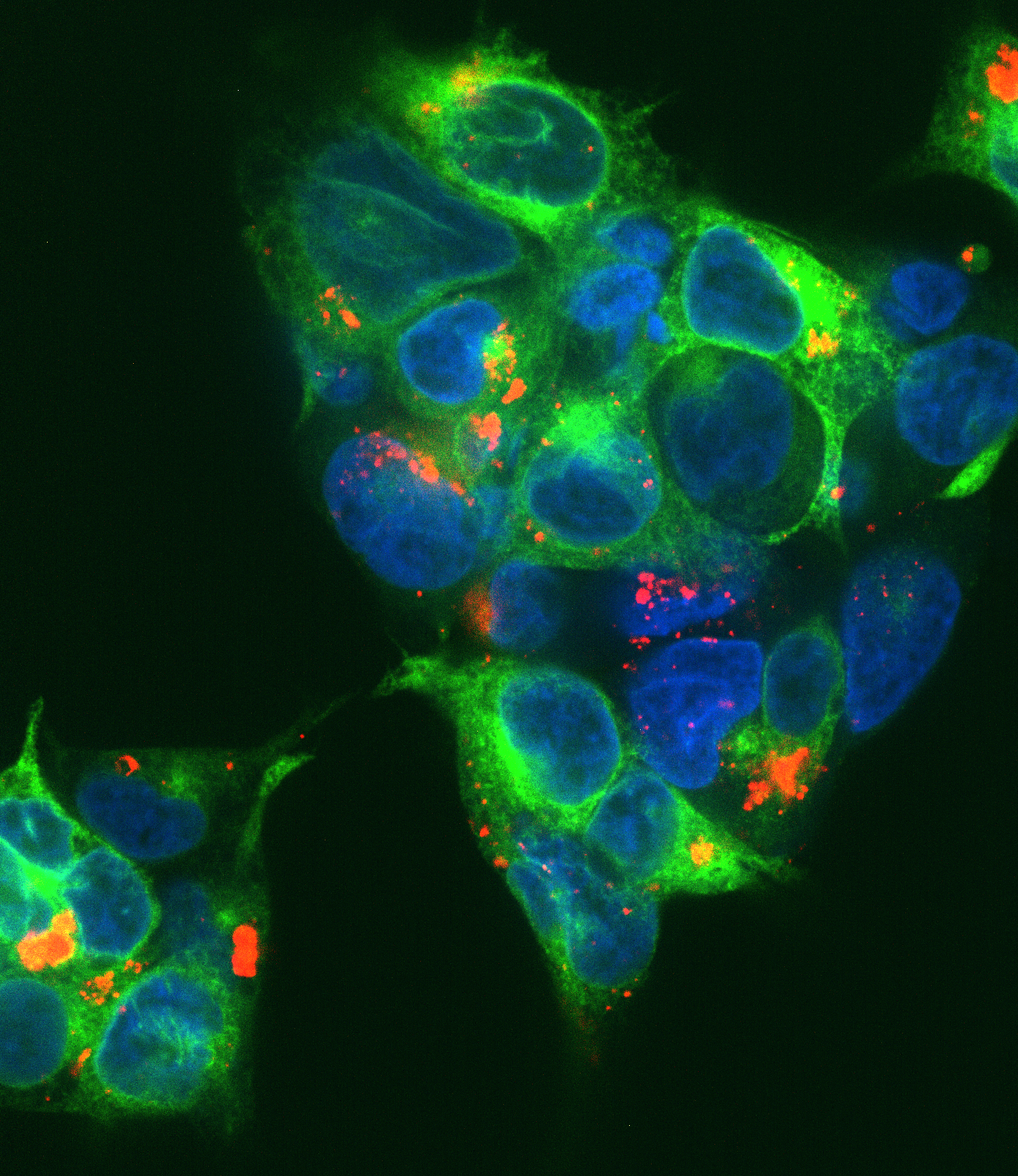

Supplement: Supplementary file 7 — Source data Fig. 2 [file 44319_2026_736_MOESM7_ESM.zip › Figure 2/2B Top panel_Replicate/Merged.tif]

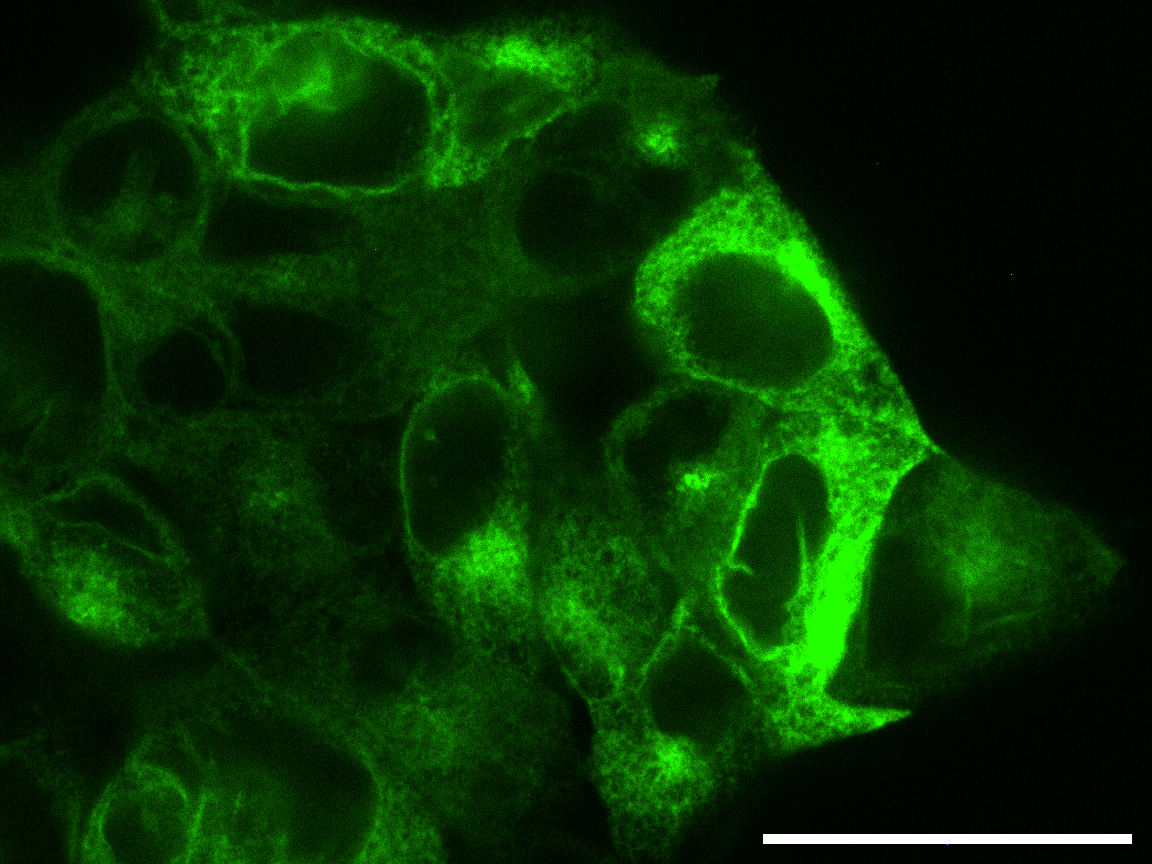

Supplement: Supplementary file 7 — Source data Fig. 2 [file 44319_2026_736_MOESM7_ESM.zip › Figure 2/2B Top panel/NHE3 GFP.tif]

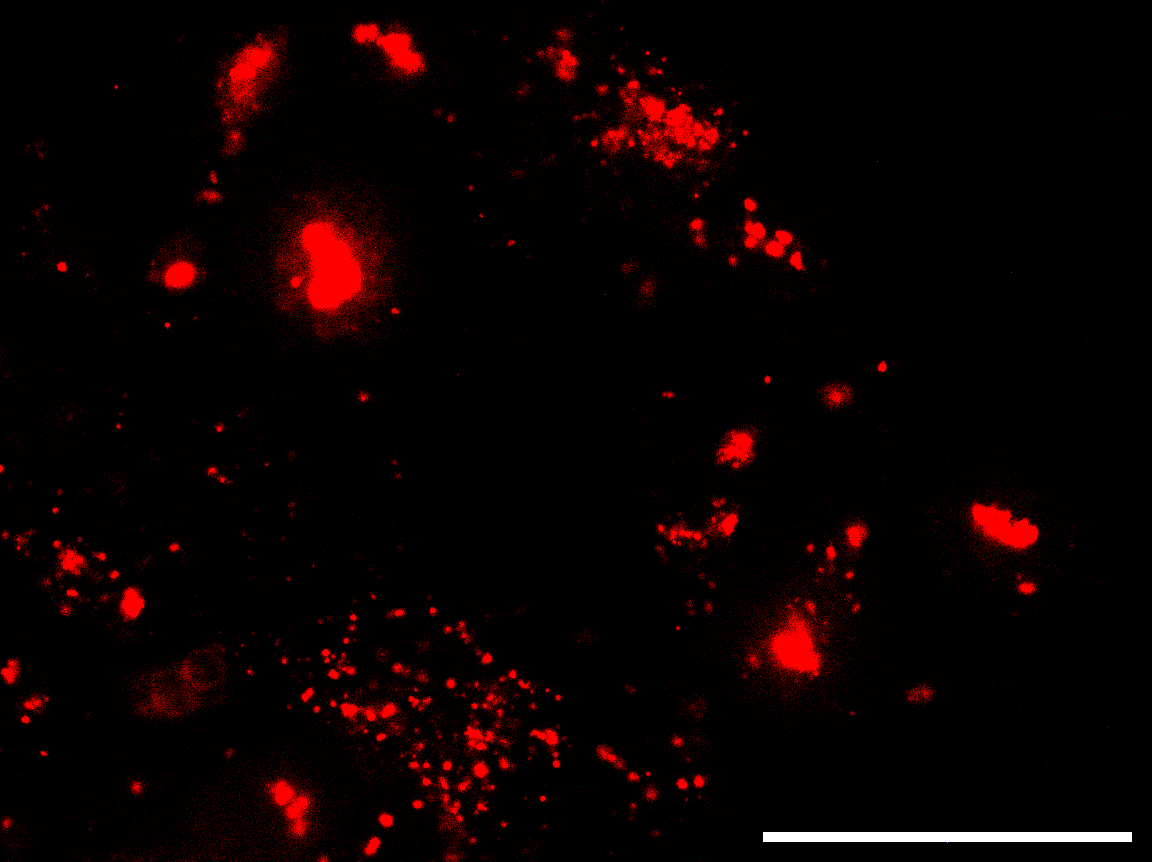

Supplement: Supplementary file 7 — Source data Fig. 2 [file 44319_2026_736_MOESM7_ESM.zip › Figure 2/2B Top panel/CTNS DsRed.tif]

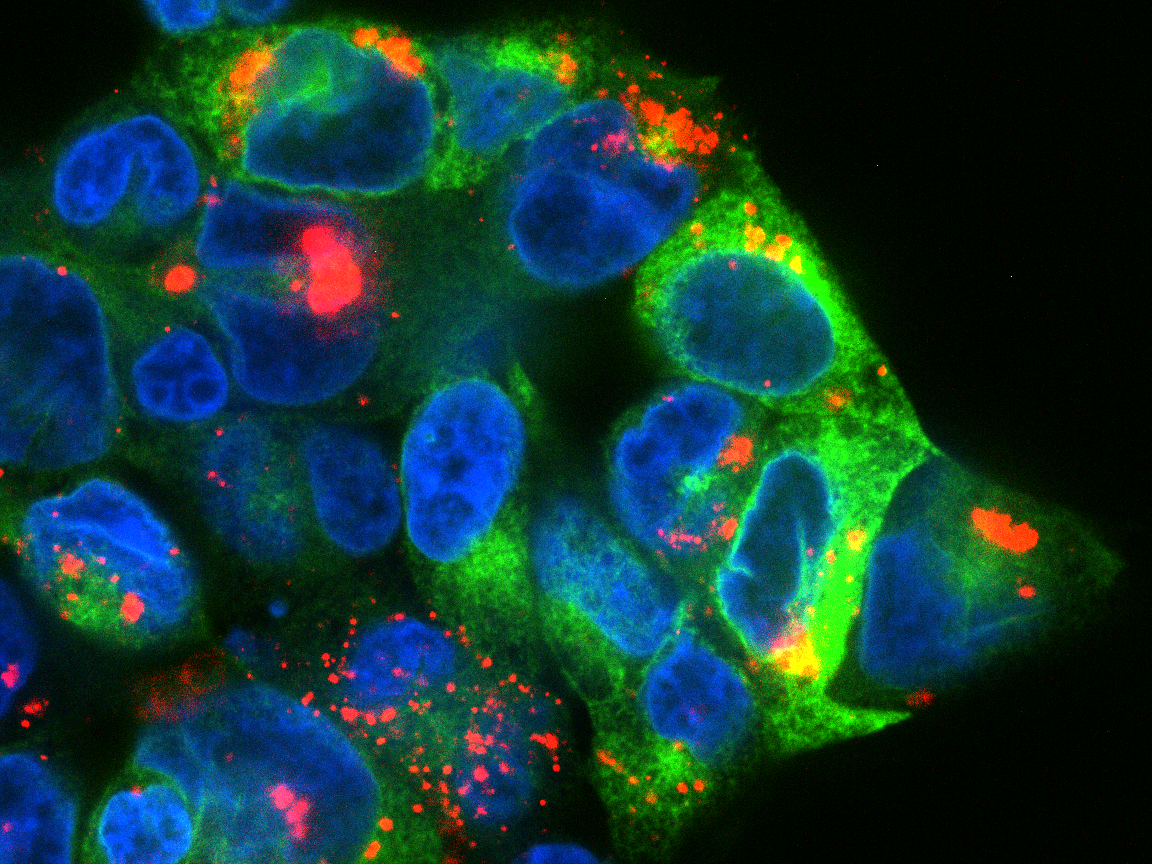

Supplement: Supplementary file 7 — Source data Fig. 2 [file 44319_2026_736_MOESM7_ESM.zip › Figure 2/2B Top panel/Merged.tif]

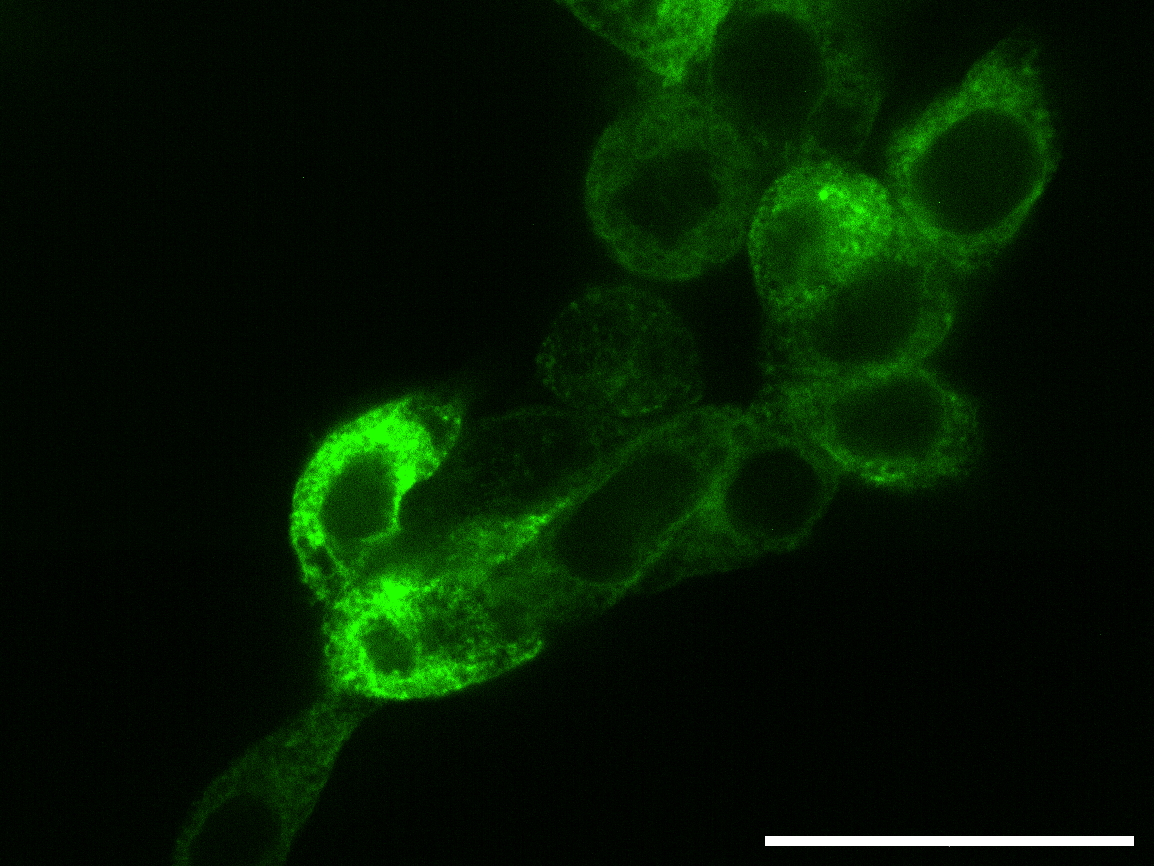

Supplement: Supplementary file 7 — Source data Fig. 2 [file 44319_2026_736_MOESM7_ESM.zip › Figure 2/2B Bottom panel/NHE3 GFP.tif]

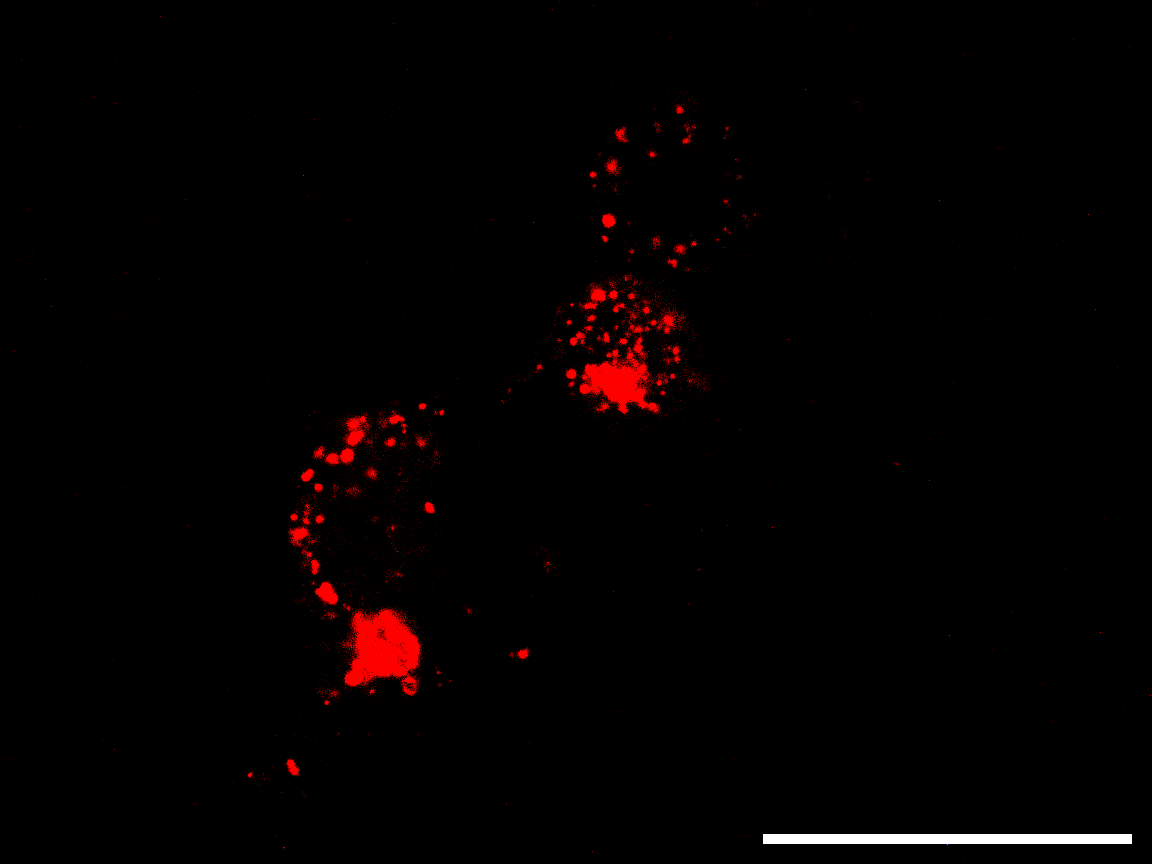

Supplement: Supplementary file 7 — Source data Fig. 2 [file 44319_2026_736_MOESM7_ESM.zip › Figure 2/2B Bottom panel/CTNS LKG DsRed.tif]

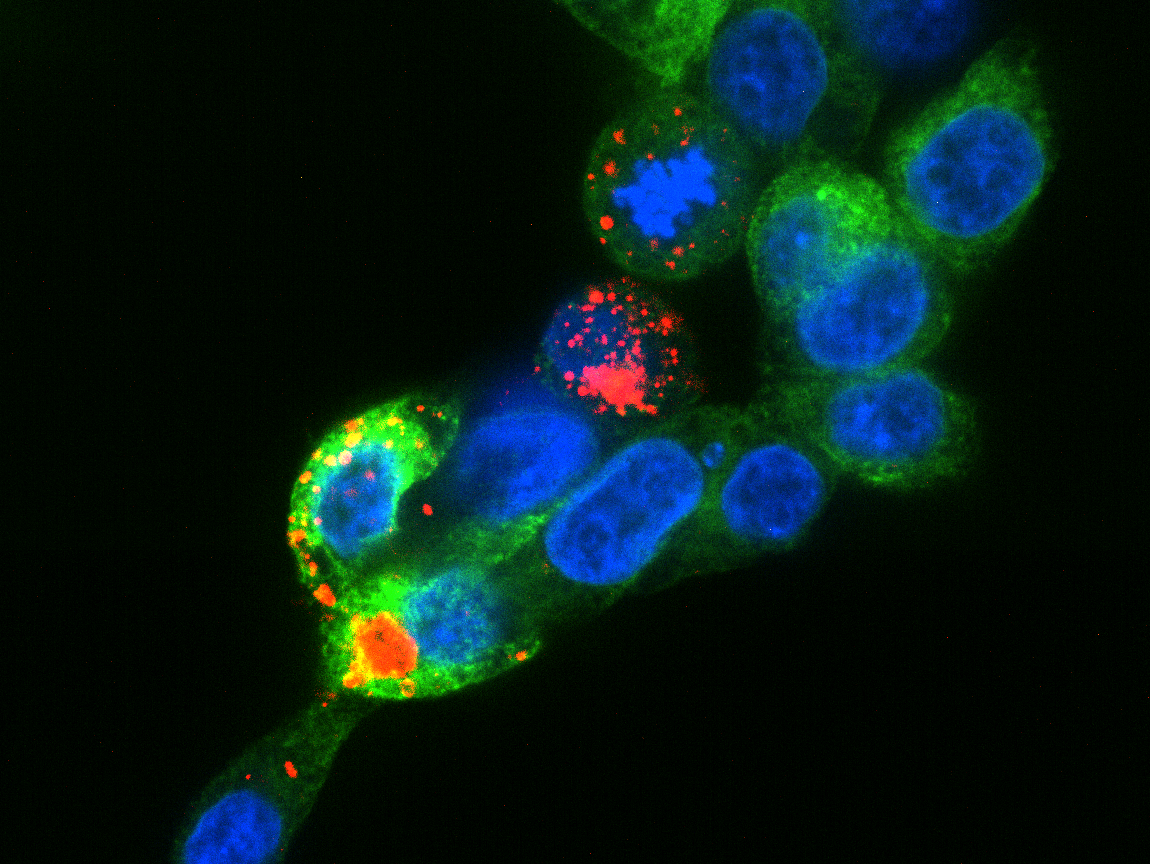

Supplement: Supplementary file 7 — Source data Fig. 2 [file 44319_2026_736_MOESM7_ESM.zip › Figure 2/2B Bottom panel/Merged.tif]

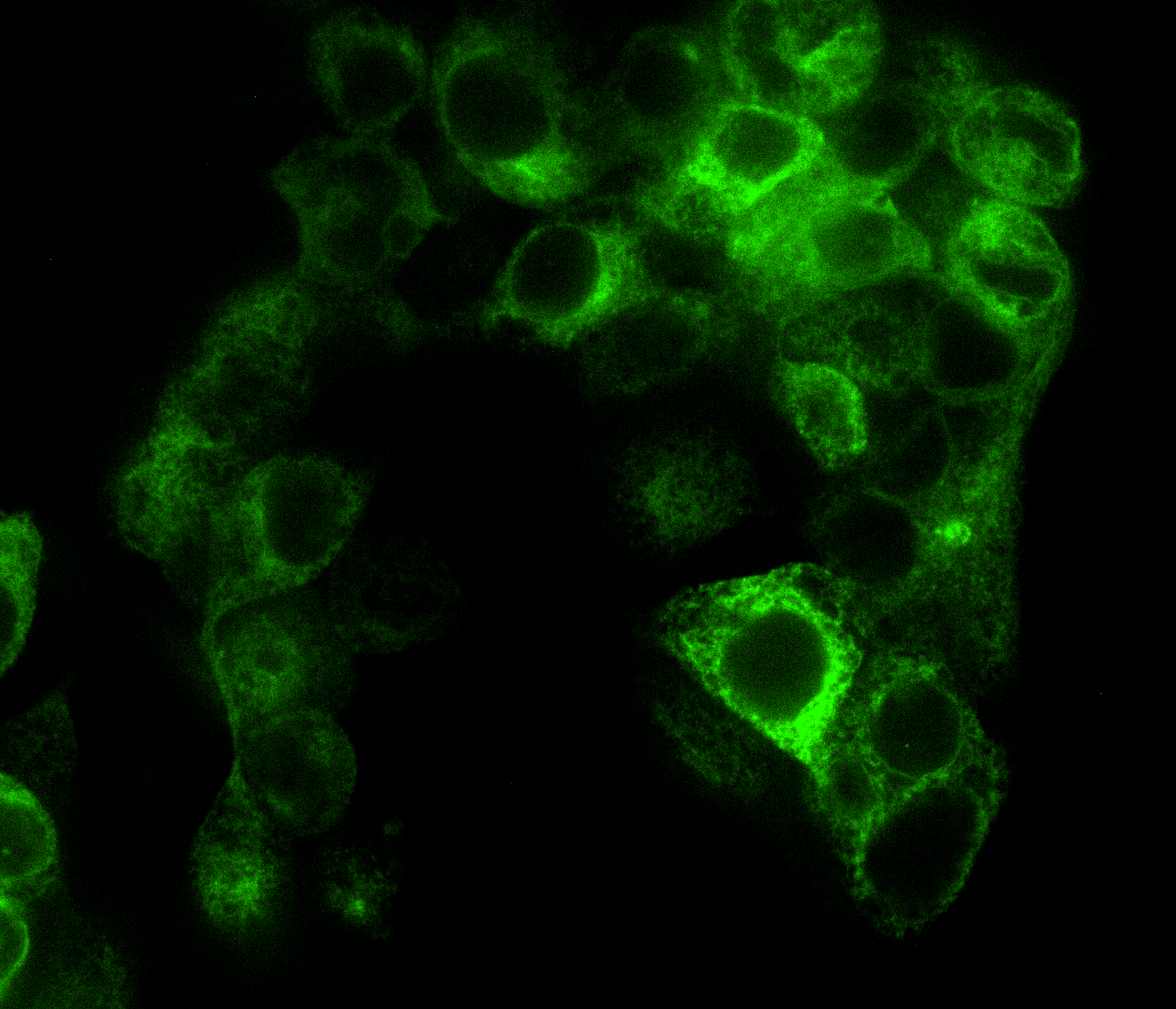

Supplement: Supplementary file 7 — Source data Fig. 2 [file 44319_2026_736_MOESM7_ESM.zip › Figure 2/2B Bottom panel_Replicate/NHE3 GFP.tif]

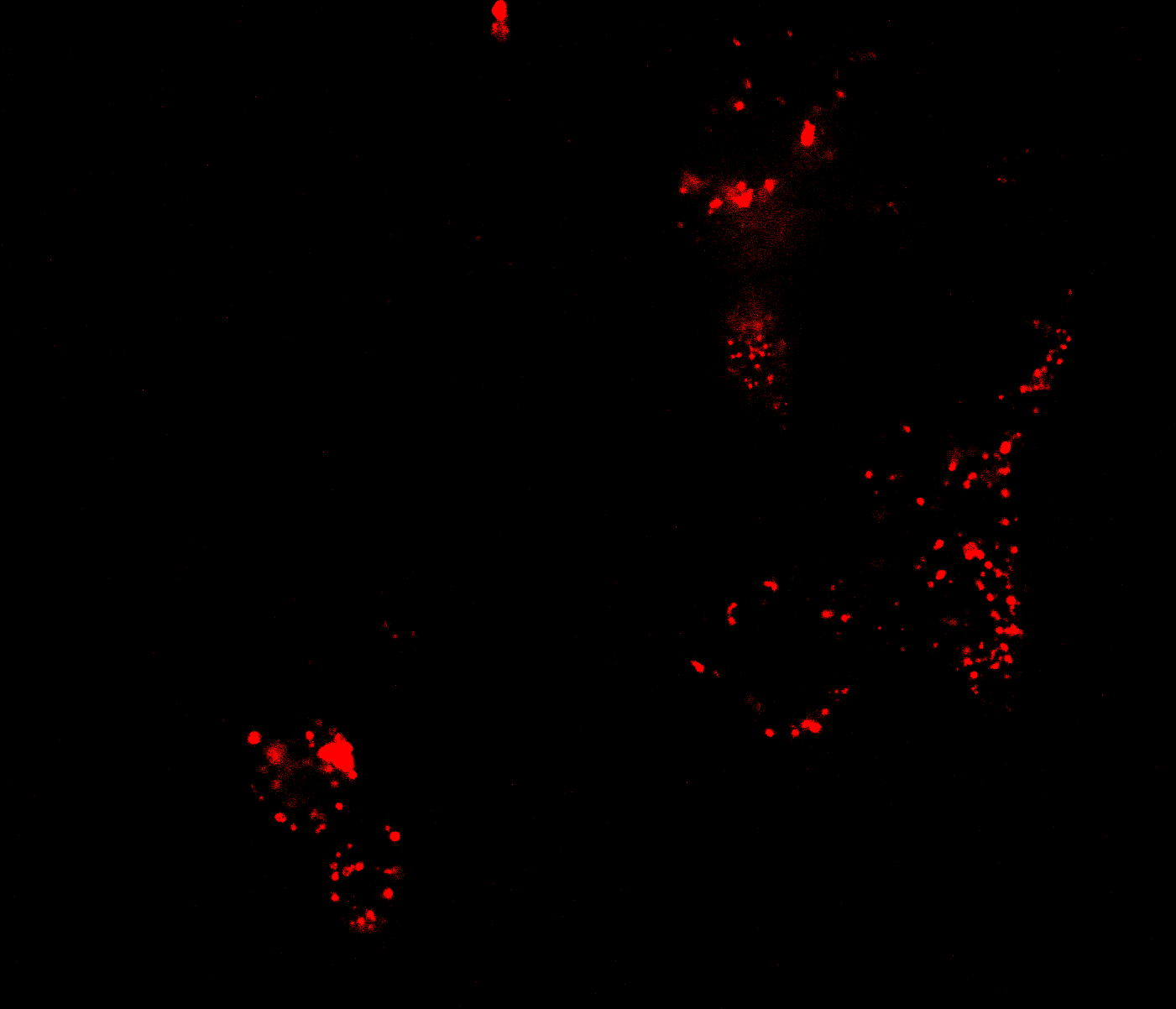

Supplement: Supplementary file 7 — Source data Fig. 2 [file 44319_2026_736_MOESM7_ESM.zip › Figure 2/2B Bottom panel_Replicate/CTNS LKG DsREd.tif]

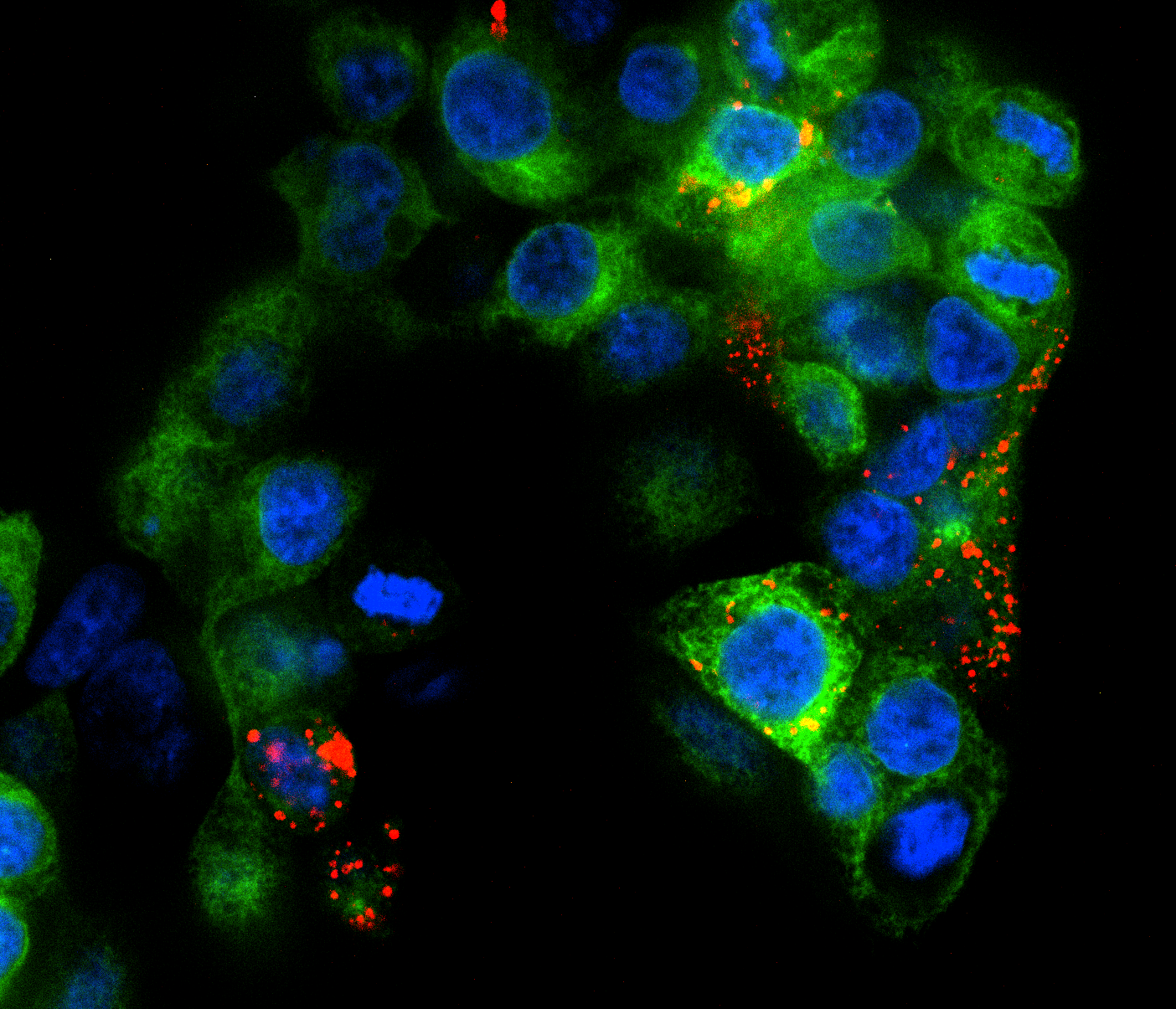

Supplement: Supplementary file 7 — Source data Fig. 2 [file 44319_2026_736_MOESM7_ESM.zip › Figure 2/2B Bottom panel_Replicate/Merged.tif]

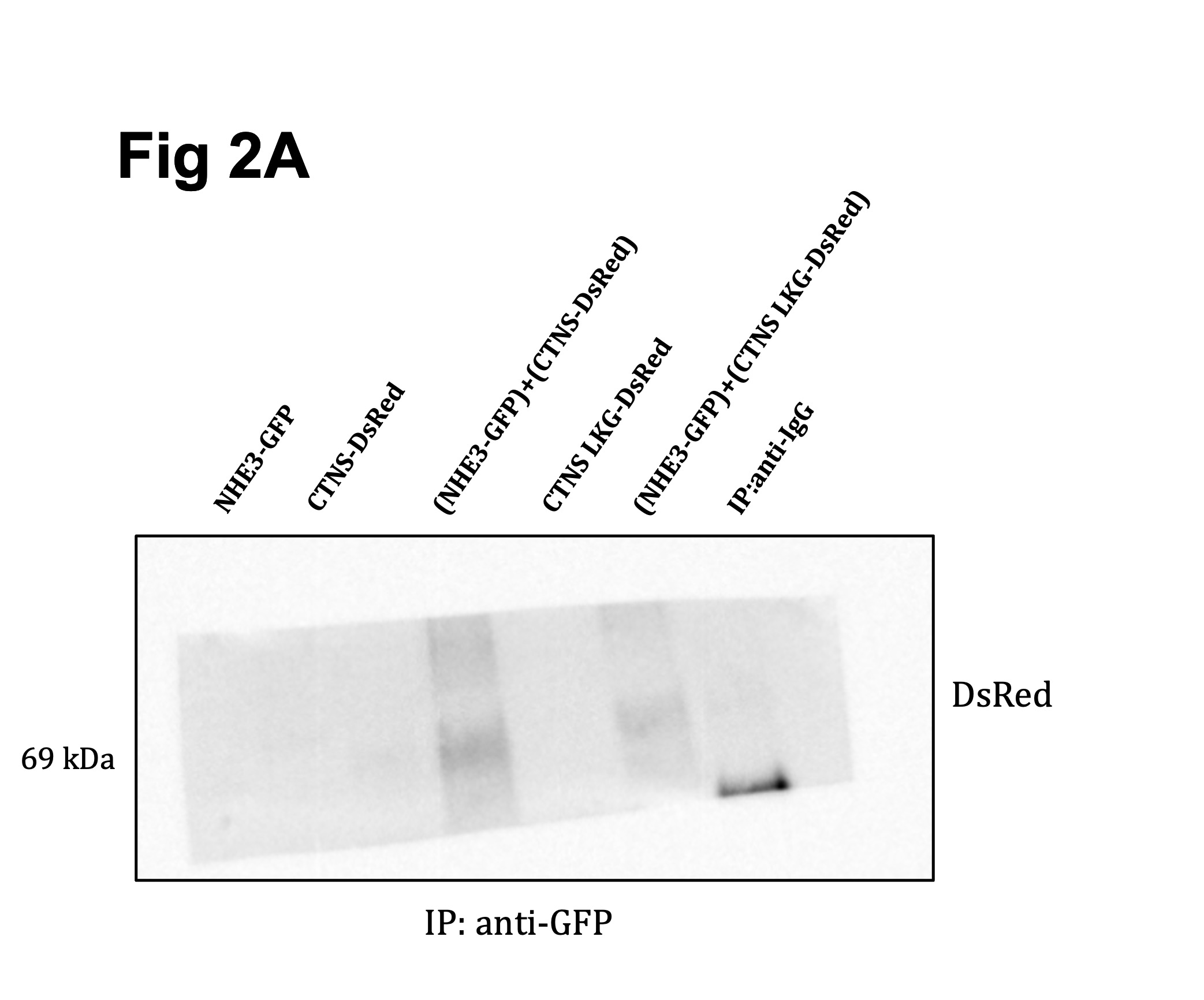

Supplement: Supplementary file 7 — Source data Fig. 2 [file 44319_2026_736_MOESM7_ESM.zip › Figure 2/2A/IP Anti-GFP/IP Anti GFP Western DsRed.jpg]

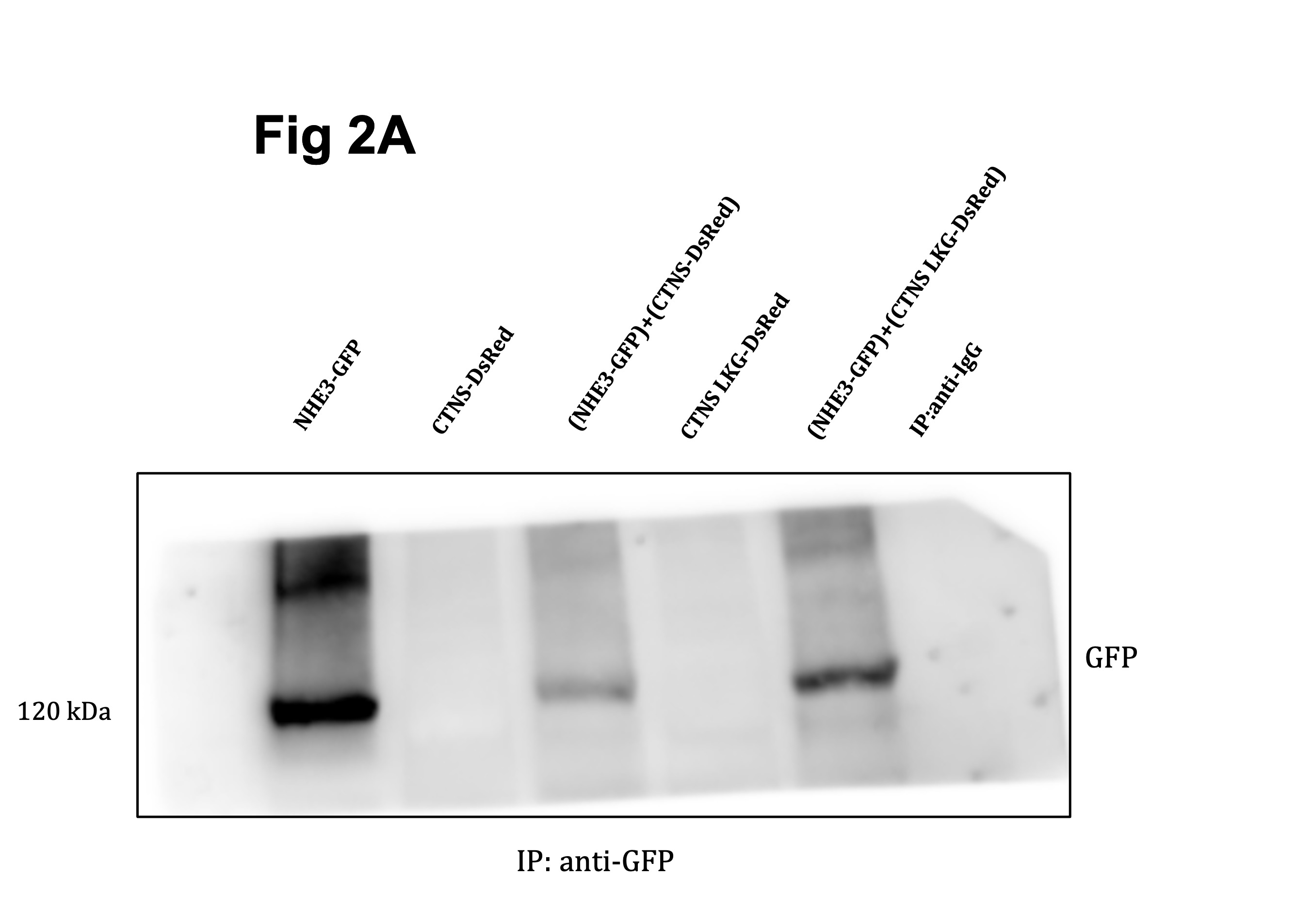

Supplement: Supplementary file 7 — Source data Fig. 2 [file 44319_2026_736_MOESM7_ESM.zip › Figure 2/2A/IP Anti-GFP/IP Anti GFP Western GFP.jpg]

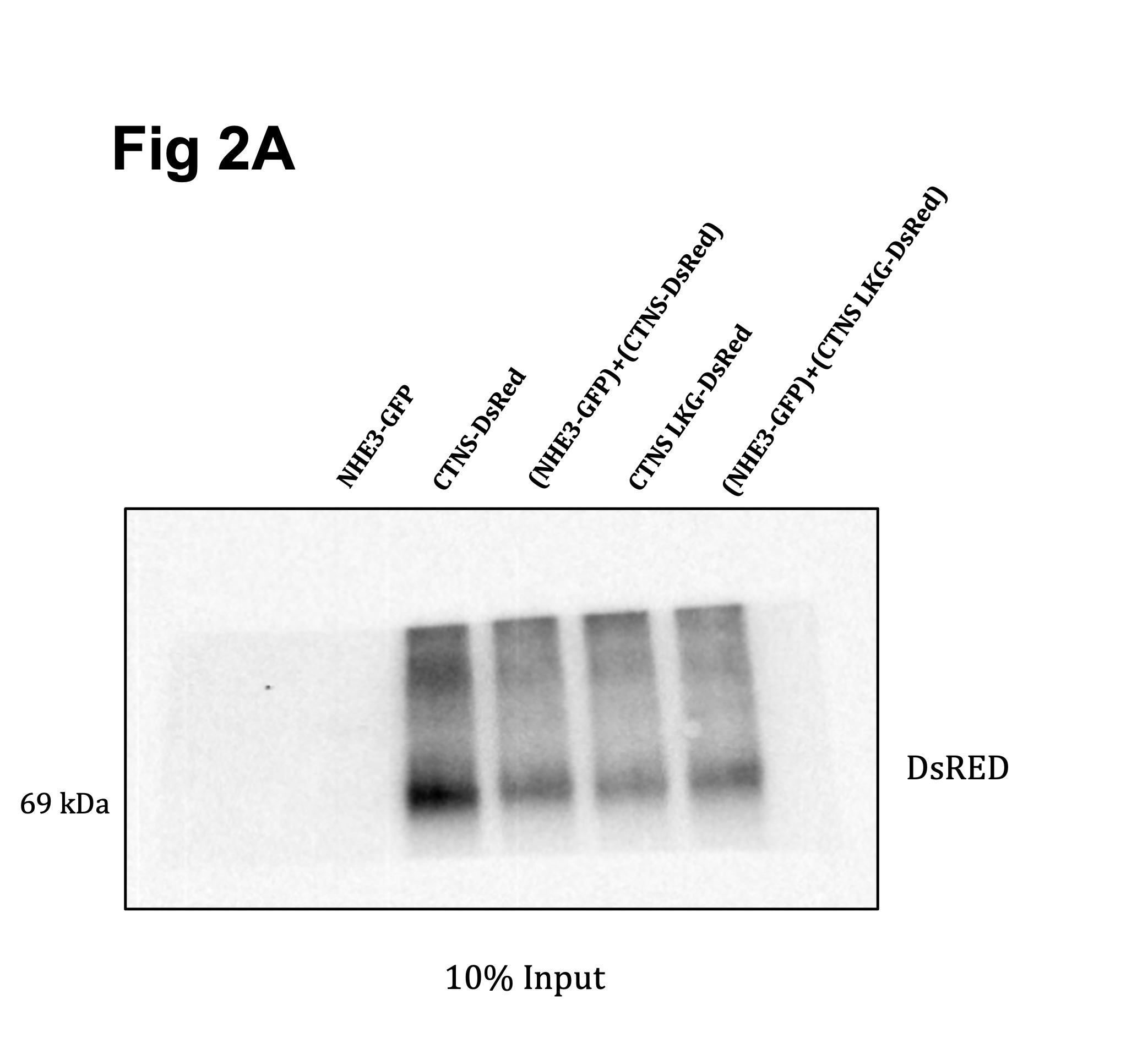

Supplement: Supplementary file 7 — Source data Fig. 2 [file 44319_2026_736_MOESM7_ESM.zip › Figure 2/2A/Input/Input_DsRed.jpg]

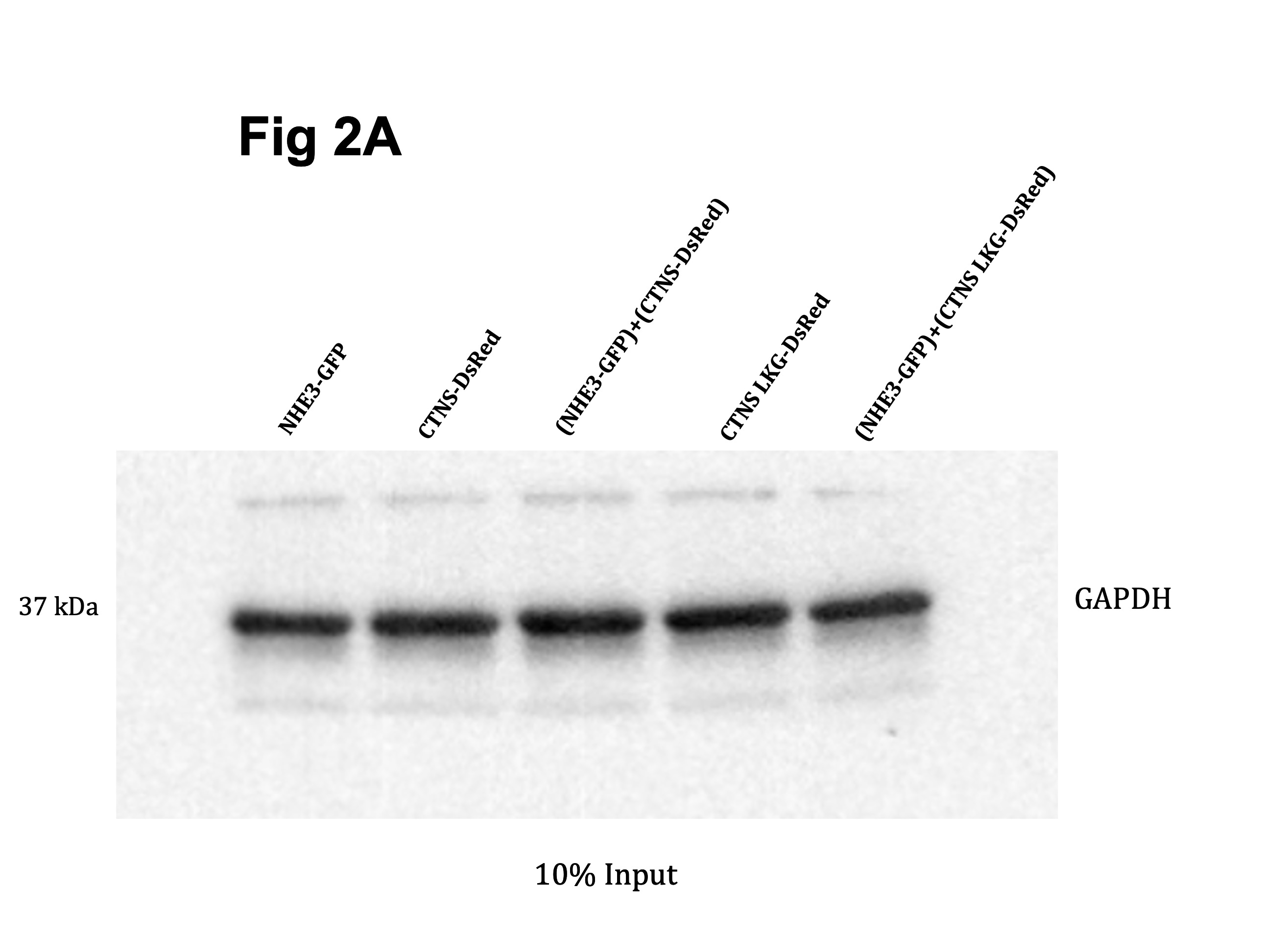

Supplement: Supplementary file 7 — Source data Fig. 2 [file 44319_2026_736_MOESM7_ESM.zip › Figure 2/2A/Input/Input_GAPDH.jpg]

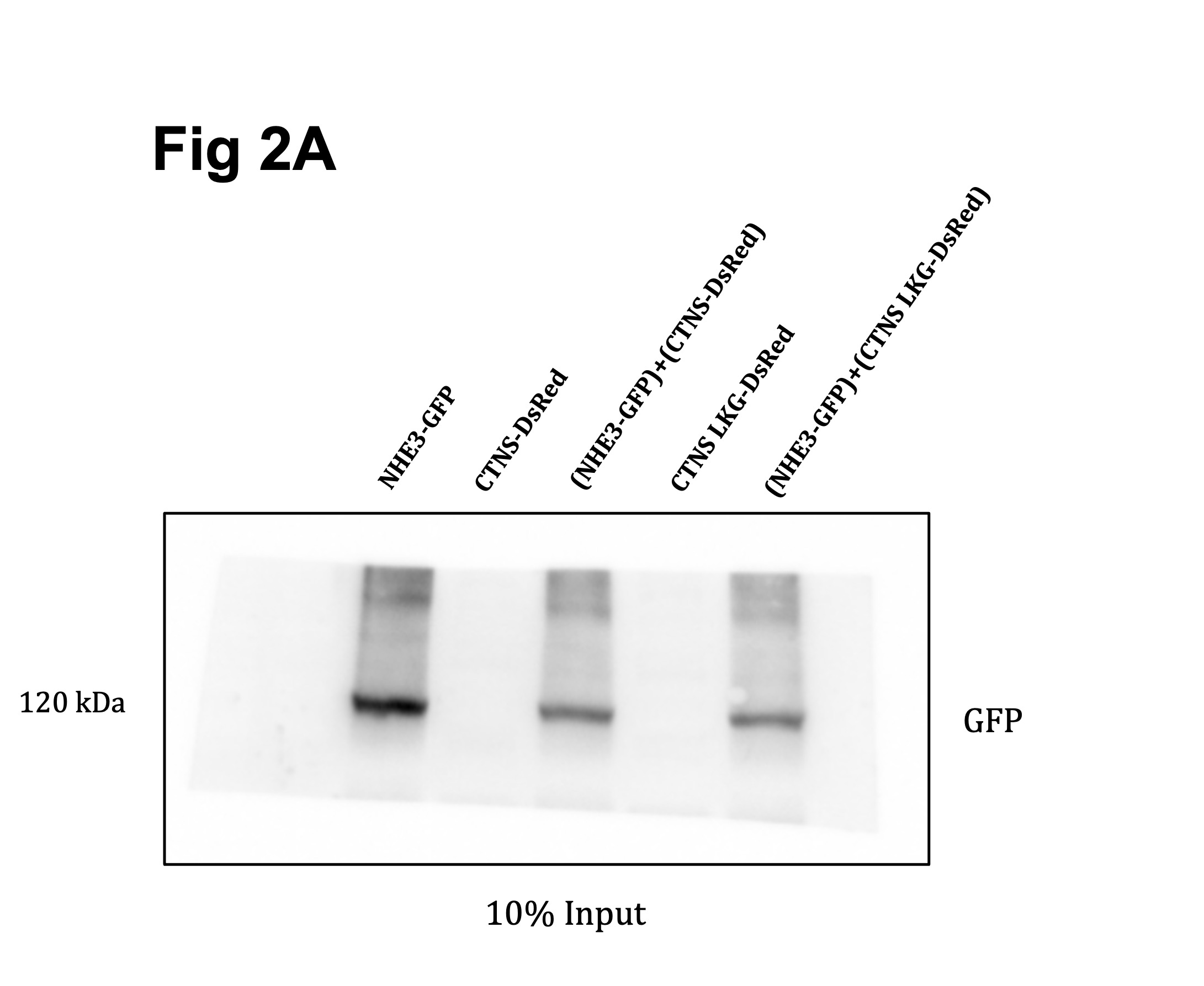

Supplement: Supplementary file 7 — Source data Fig. 2 [file 44319_2026_736_MOESM7_ESM.zip › Figure 2/2A/Input/Input_GFP.jpg]

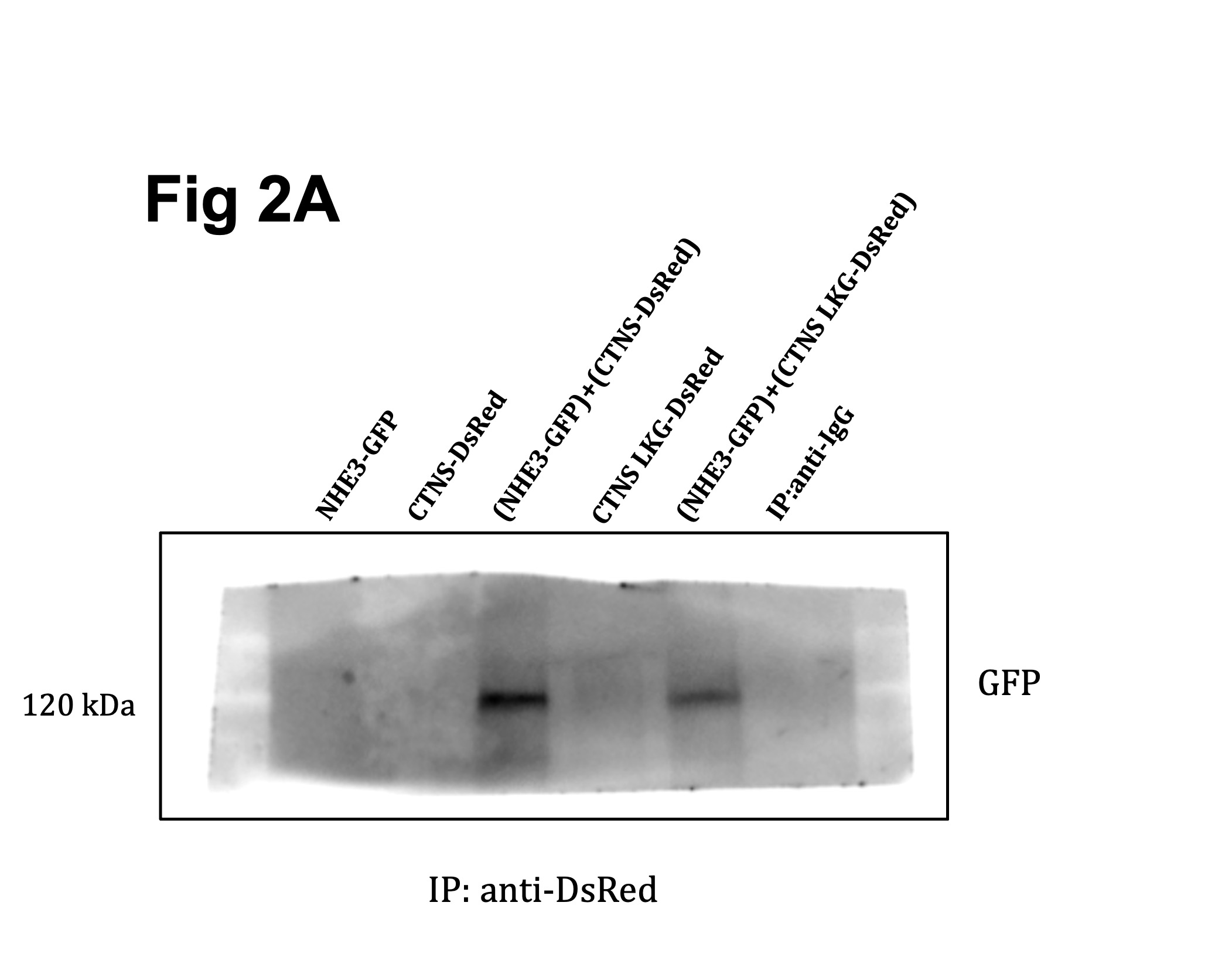

Supplement: Supplementary file 7 — Source data Fig. 2 [file 44319_2026_736_MOESM7_ESM.zip › Figure 2/2A/IP DsRed/IP anti DsRed Western GFP.jpg]

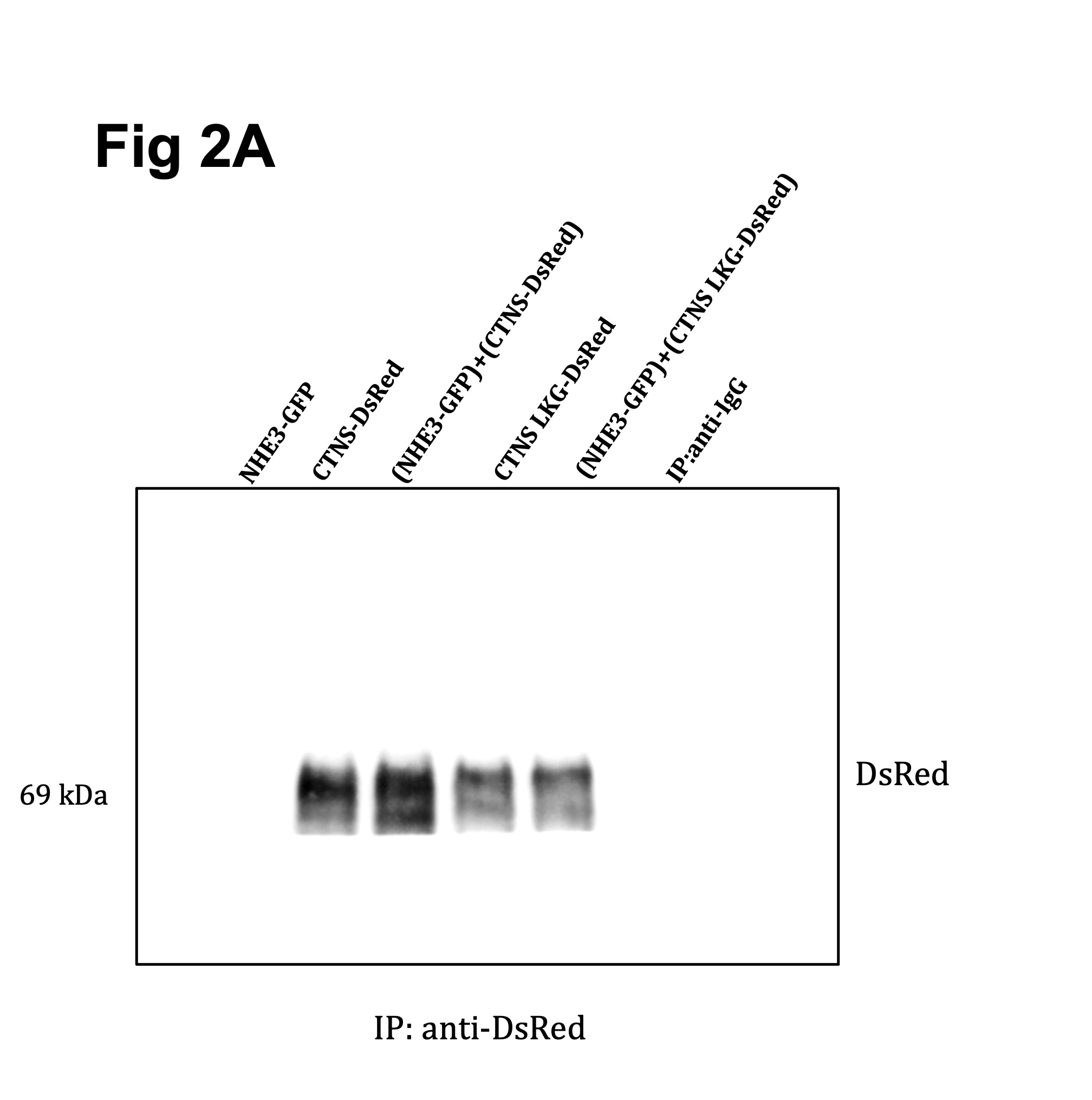

Supplement: Supplementary file 7 — Source data Fig. 2 [file 44319_2026_736_MOESM7_ESM.zip › Figure 2/2A/IP DsRed/IP Anti DsRed Western DsRed.jpg]

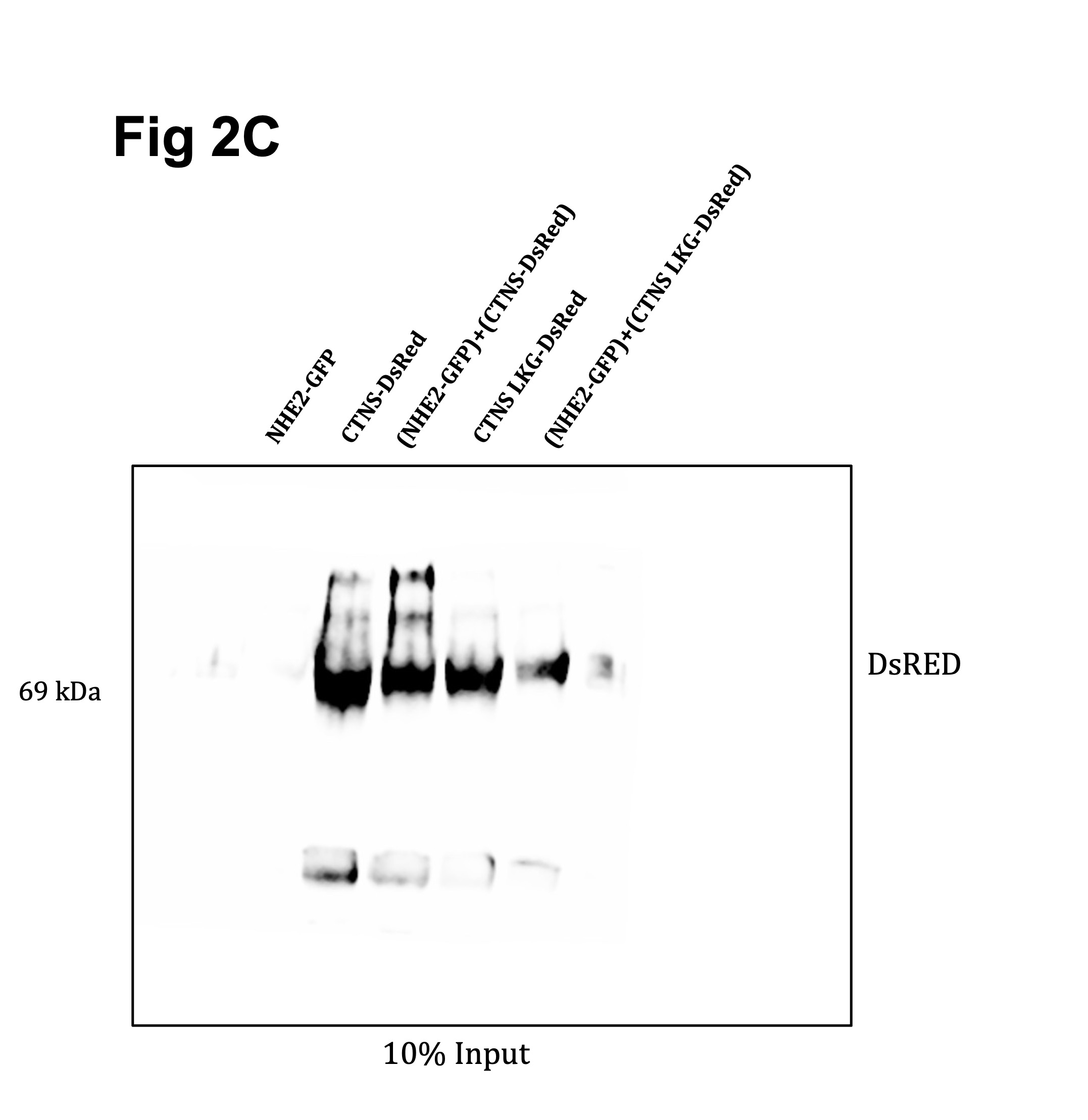

Supplement: Supplementary file 7 — Source data Fig. 2 [file 44319_2026_736_MOESM7_ESM.zip › Figure 2/2C/Input NHE2/Input_DsRed.jpg]

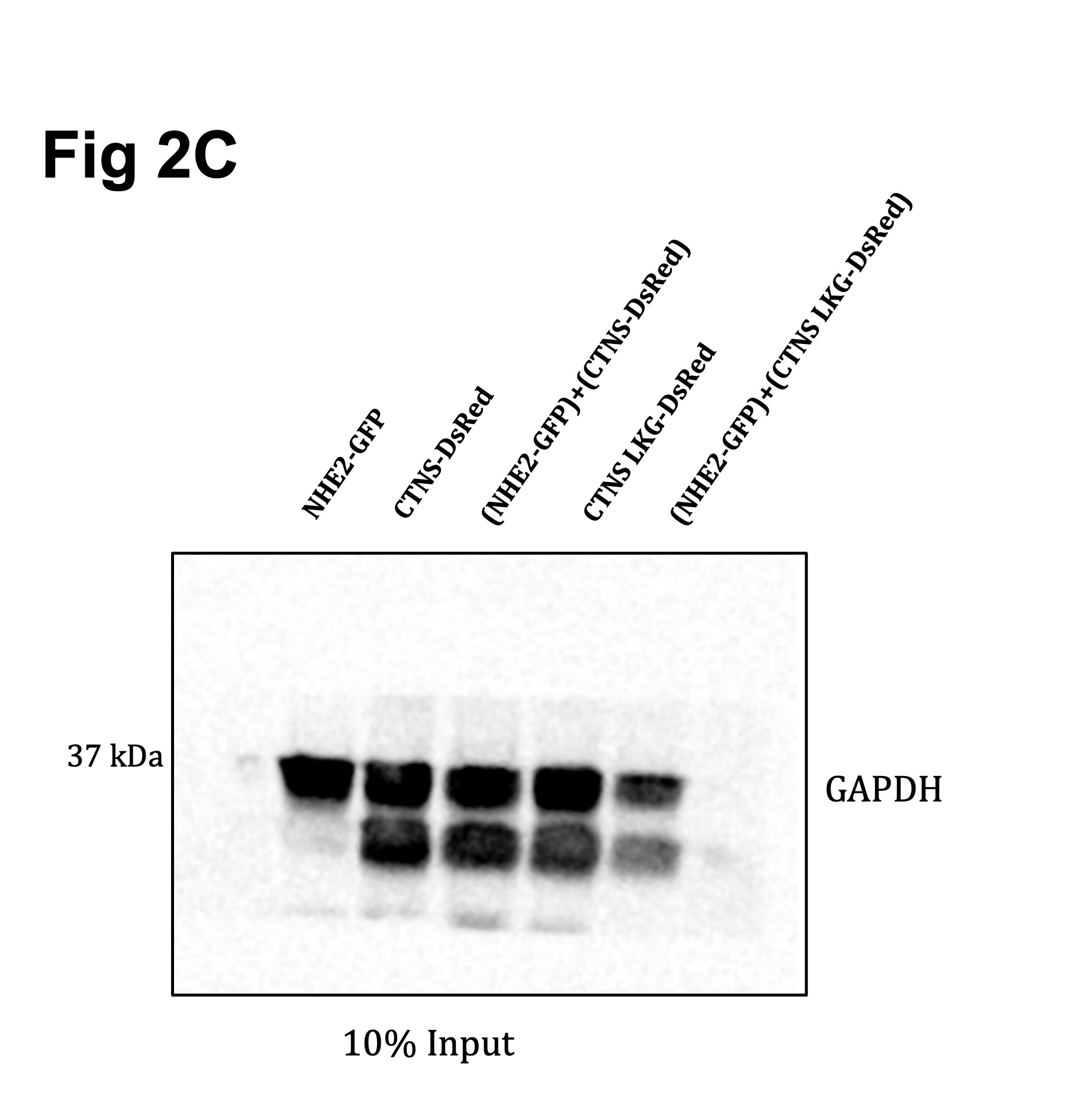

Supplement: Supplementary file 7 — Source data Fig. 2 [file 44319_2026_736_MOESM7_ESM.zip › Figure 2/2C/Input NHE2/Input_GAPDH.jpg]

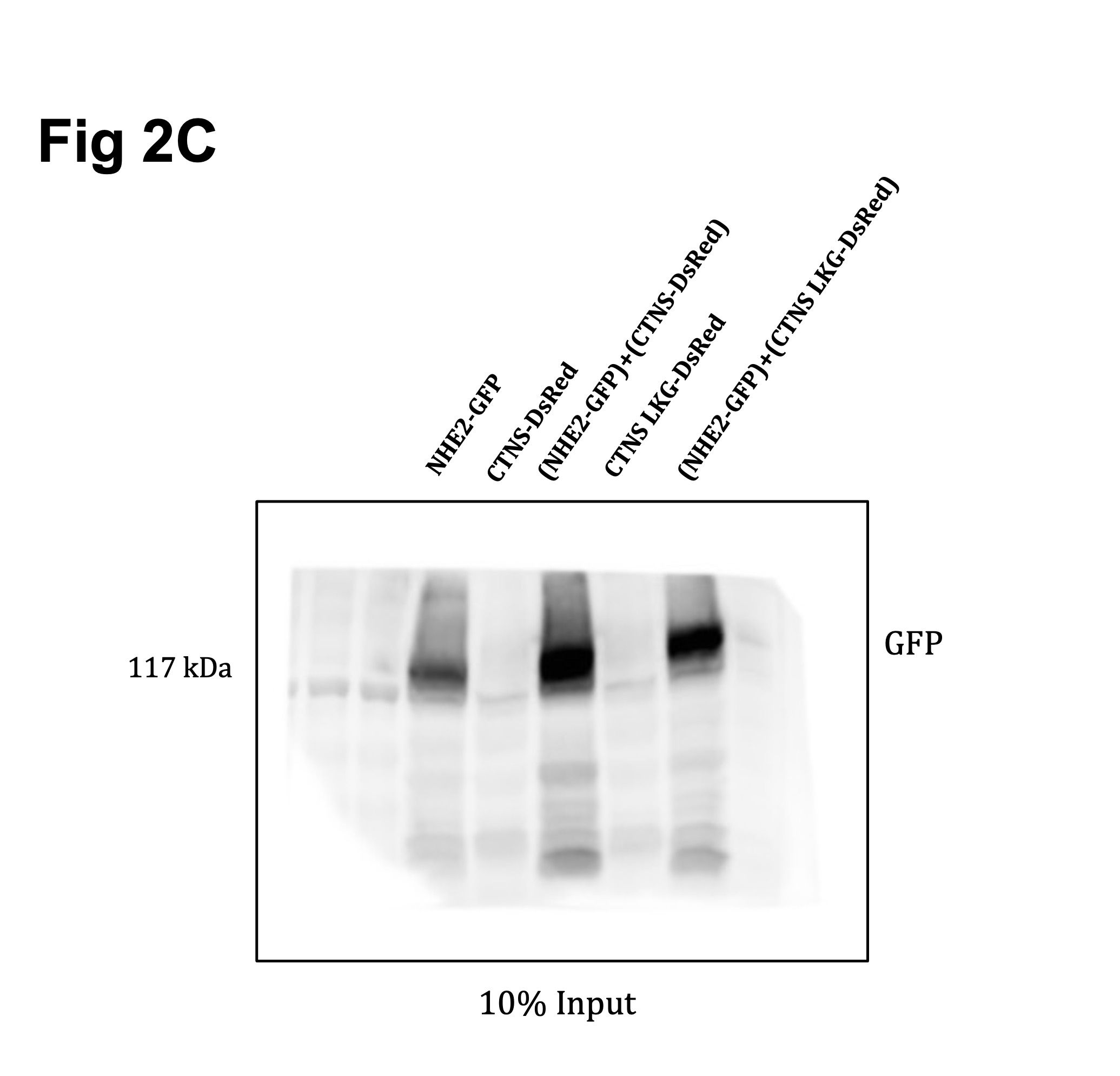

Supplement: Supplementary file 7 — Source data Fig. 2 [file 44319_2026_736_MOESM7_ESM.zip › Figure 2/2C/Input NHE2/Input_GFP.jpg]

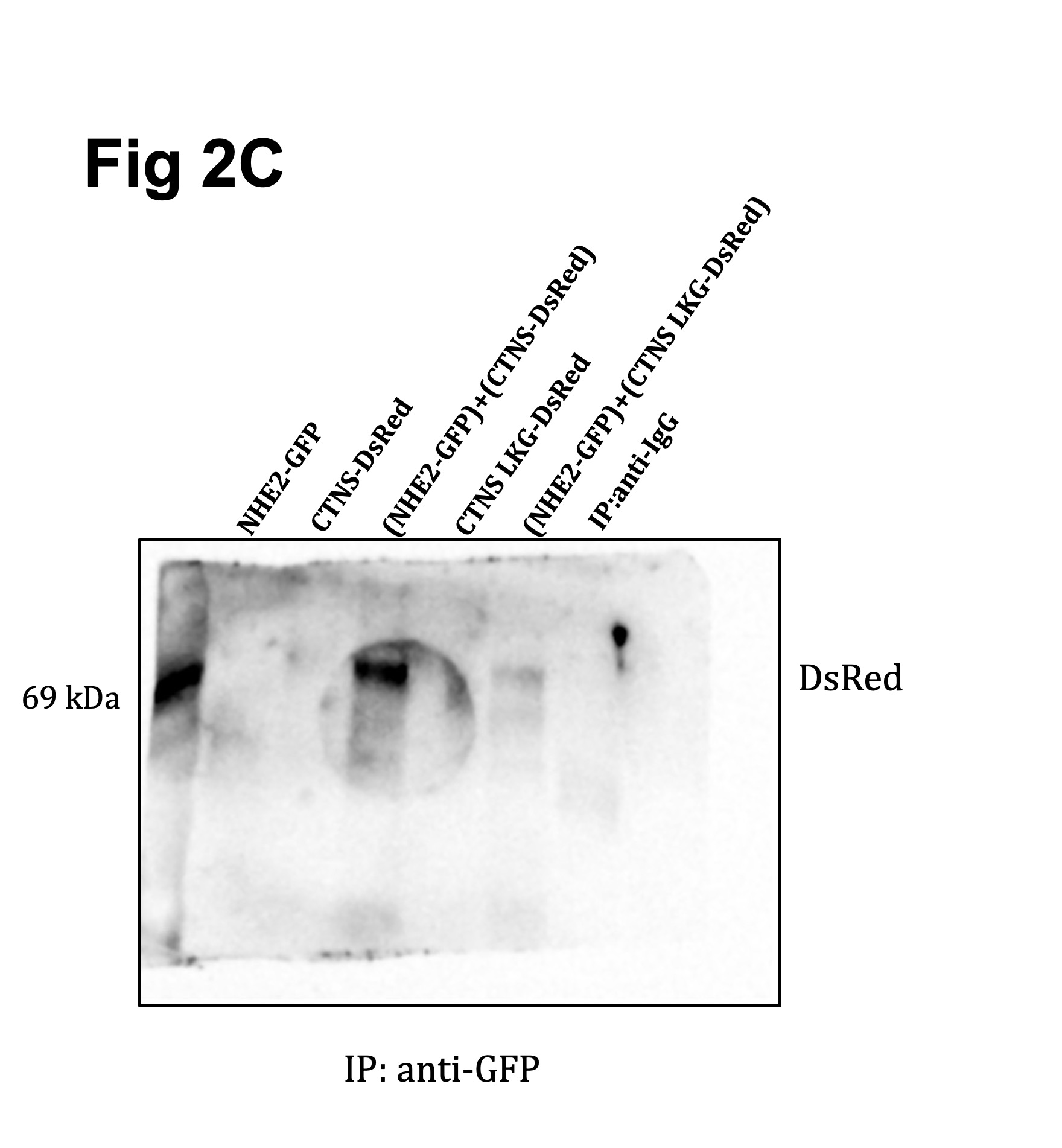

Supplement: Supplementary file 7 — Source data Fig. 2 [file 44319_2026_736_MOESM7_ESM.zip › Figure 2/2C/IP GFP/IP Anti GFP Western DsRed.jpg]

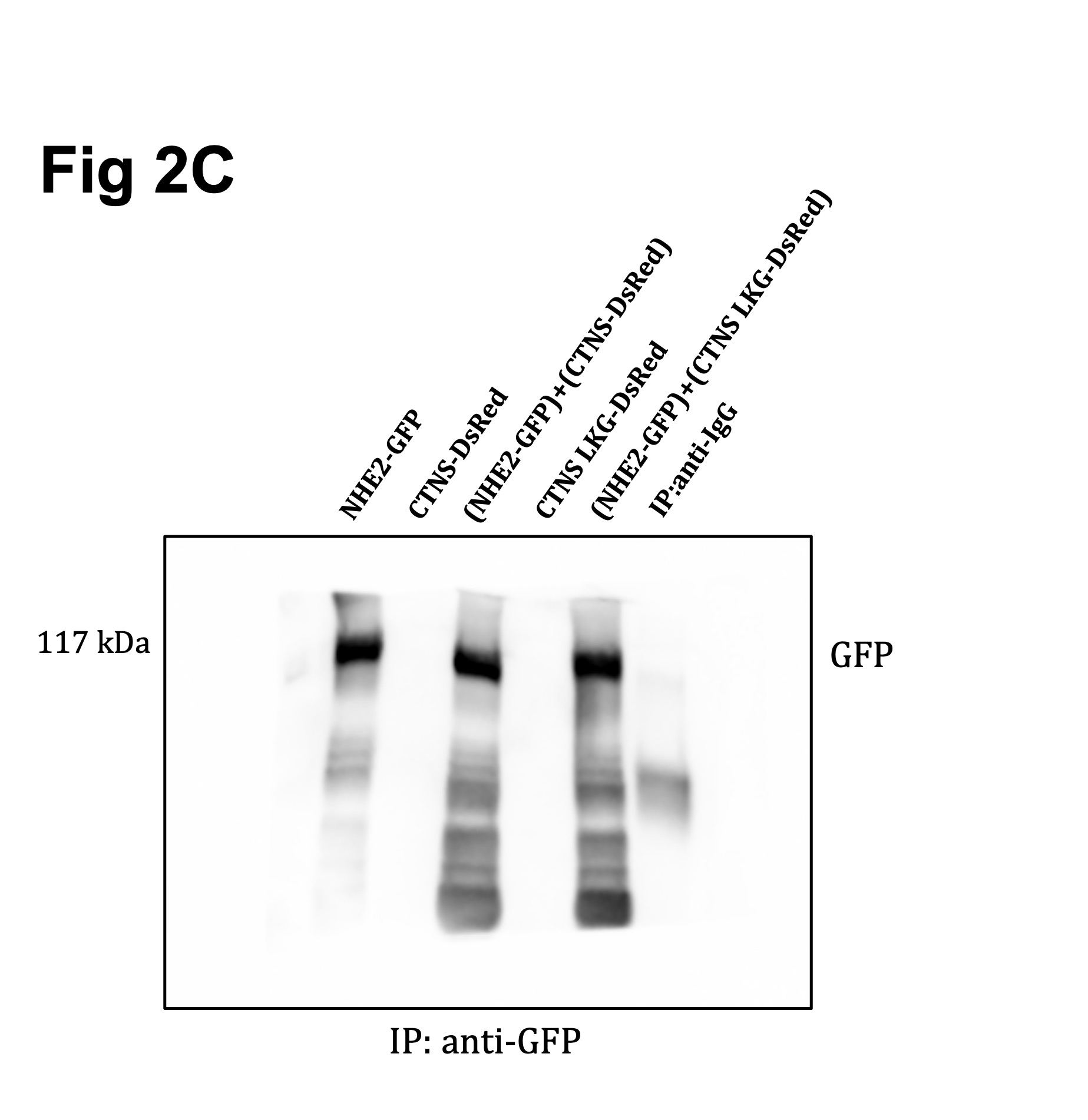

Supplement: Supplementary file 7 — Source data Fig. 2 [file 44319_2026_736_MOESM7_ESM.zip › Figure 2/2C/IP GFP/IP Anti GFP Western GFP.jpg]

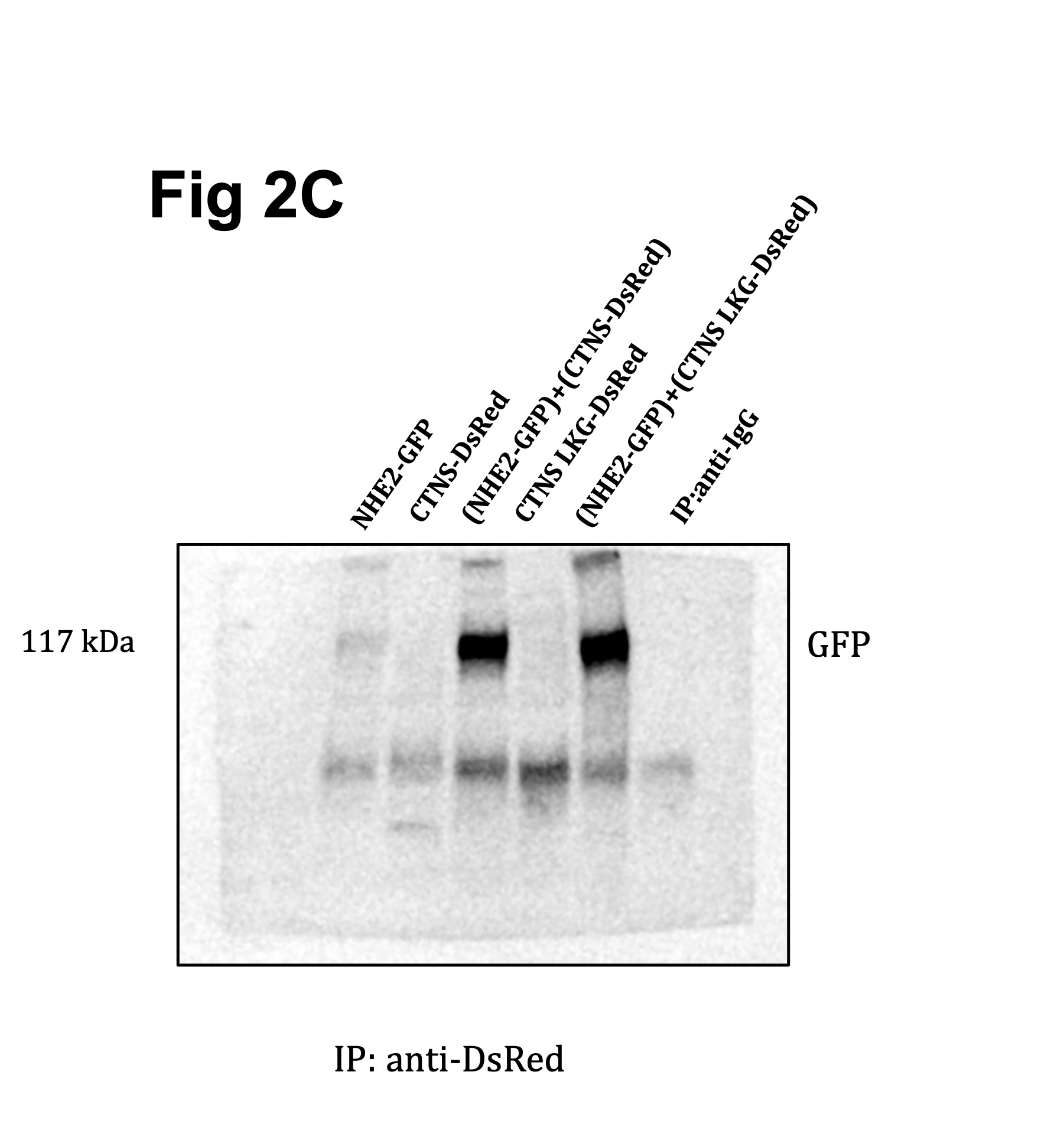

Supplement: Supplementary file 7 — Source data Fig. 2 [file 44319_2026_736_MOESM7_ESM.zip › Figure 2/2C/IP DsRed/IP anti DsRed Western GFP.jpg]

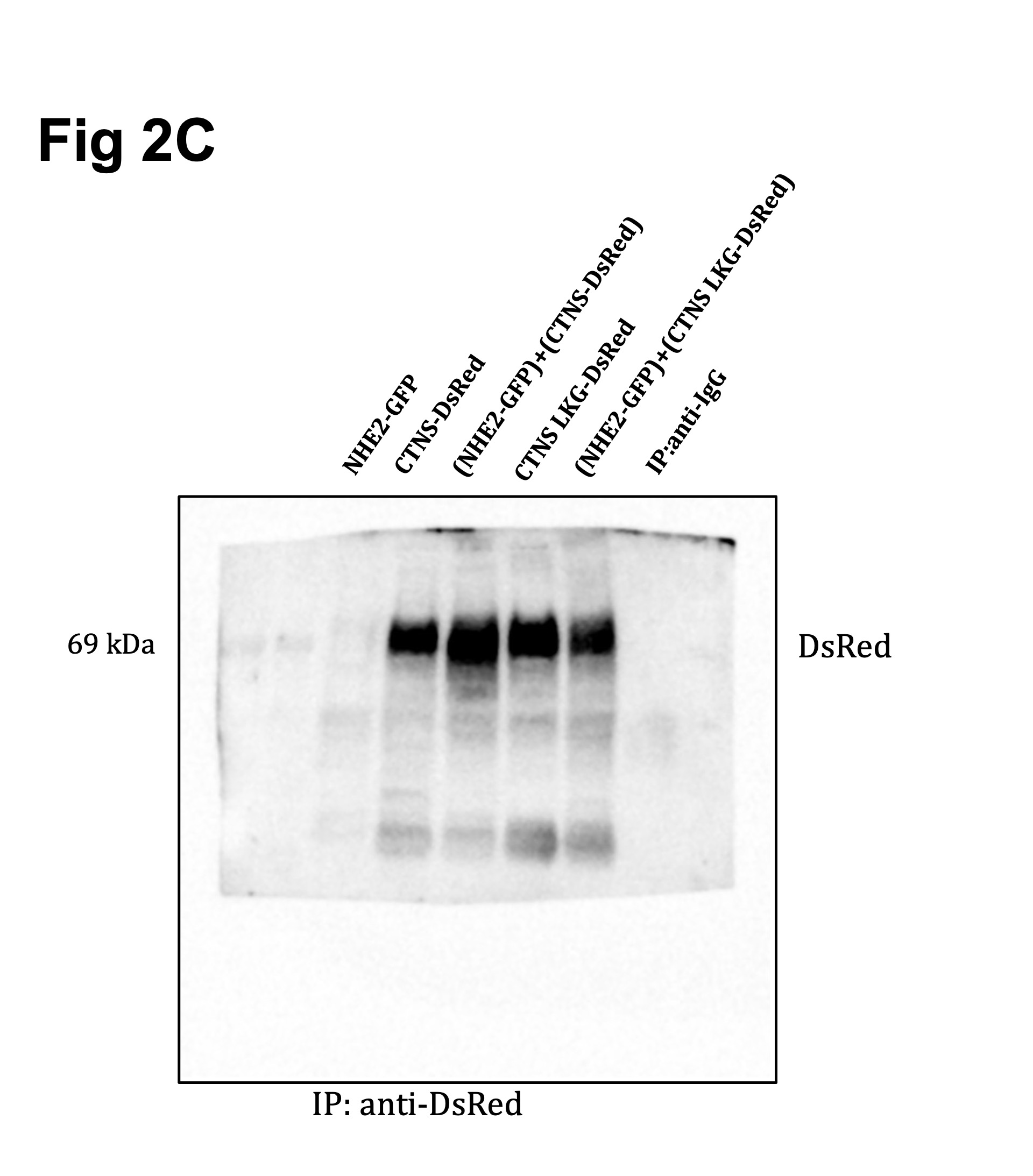

Supplement: Supplementary file 7 — Source data Fig. 2 [file 44319_2026_736_MOESM7_ESM.zip › Figure 2/2C/IP DsRed/IP anti DsRed Western DsRed.jpg]

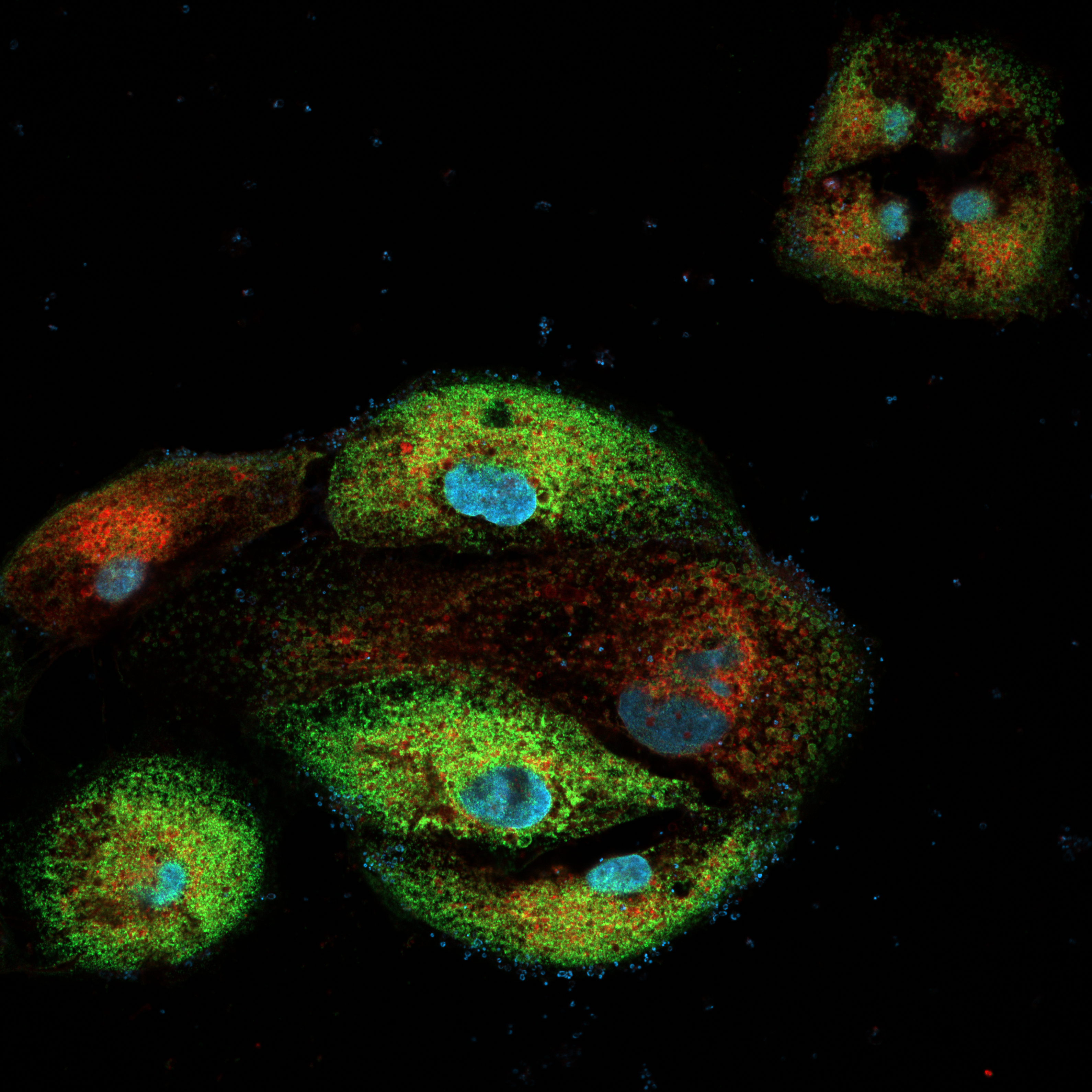

Supplement: Supplementary file 8 — Source data Fig. 3 [file 44319_2026_736_MOESM8_ESM.zip › Figure 3/3D/3D_CTNS KO_Replicate/HK-2 CTNS KO_NHE3-GFP ER tracker_Merged.tif]

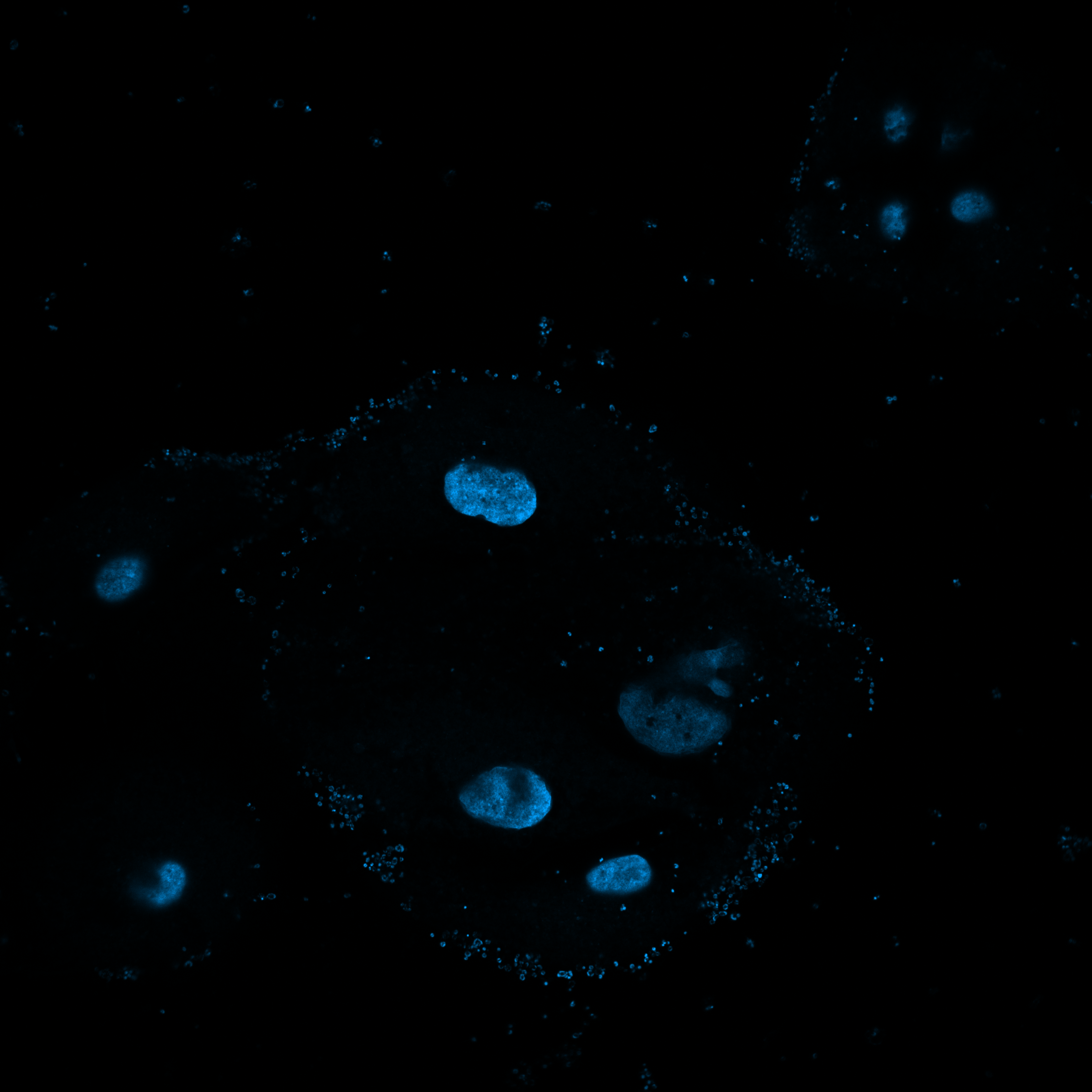

Supplement: Supplementary file 8 — Source data Fig. 3 [file 44319_2026_736_MOESM8_ESM.zip › Figure 3/3D/3D_CTNS KO_Replicate/HK-2 CTNS KO_NHE3-GFP ER tracker_DAPI.tif]

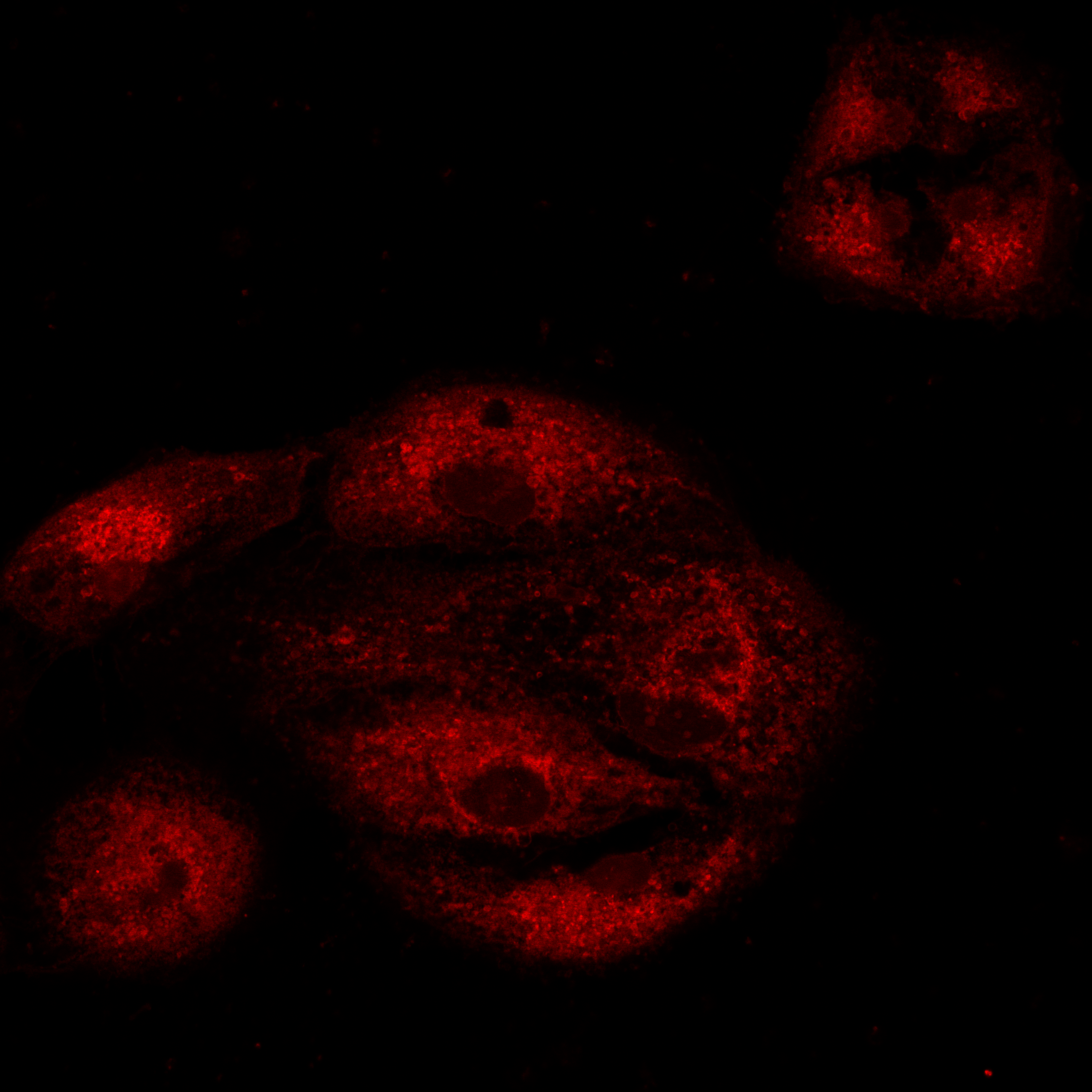

Supplement: Supplementary file 8 — Source data Fig. 3 [file 44319_2026_736_MOESM8_ESM.zip › Figure 3/3D/3D_CTNS KO_Replicate/HK-2 CTNS KO_NHE3-GFP ER tracker_ER Tracker.tif]

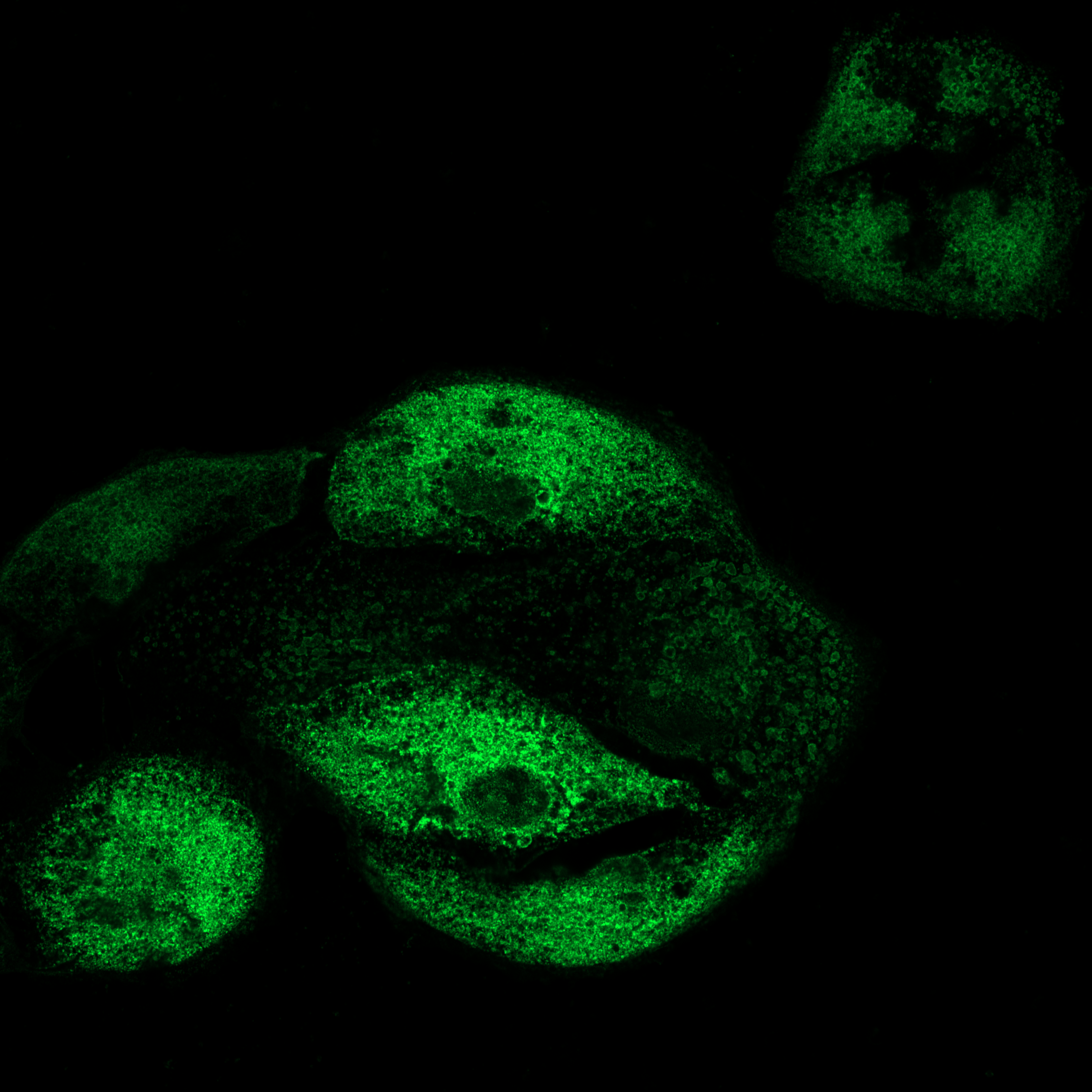

Supplement: Supplementary file 8 — Source data Fig. 3 [file 44319_2026_736_MOESM8_ESM.zip › Figure 3/3D/3D_CTNS KO_Replicate/HK-2 CTNS KO_NHE3-GFP ER tracker_NHE3 GFP.tif]

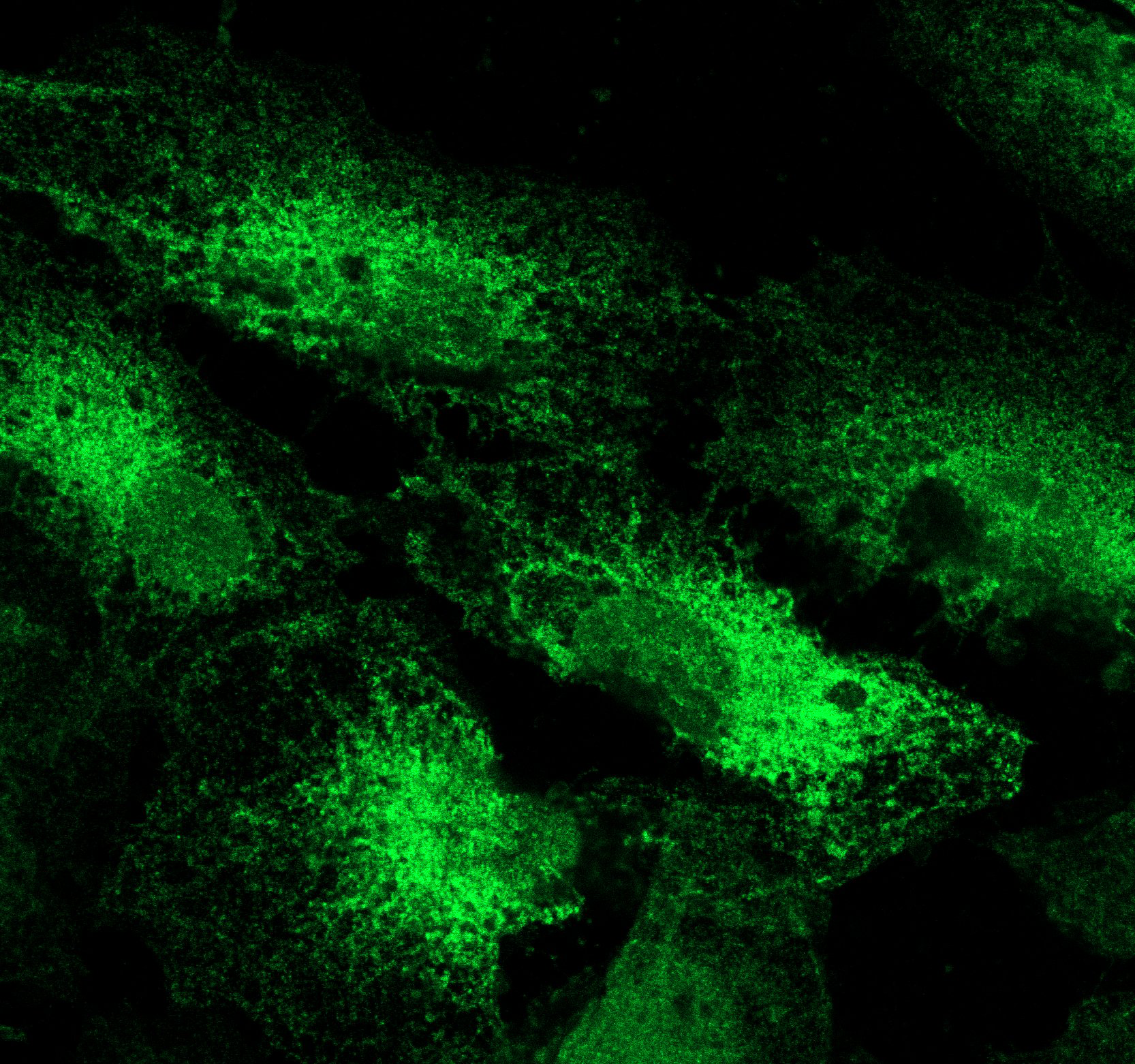

Supplement: Supplementary file 8 — Source data Fig. 3 [file 44319_2026_736_MOESM8_ESM.zip › Figure 3/3D/3D_WT/HK-2 WT_ER tracker_NHE3-GFP.tif]

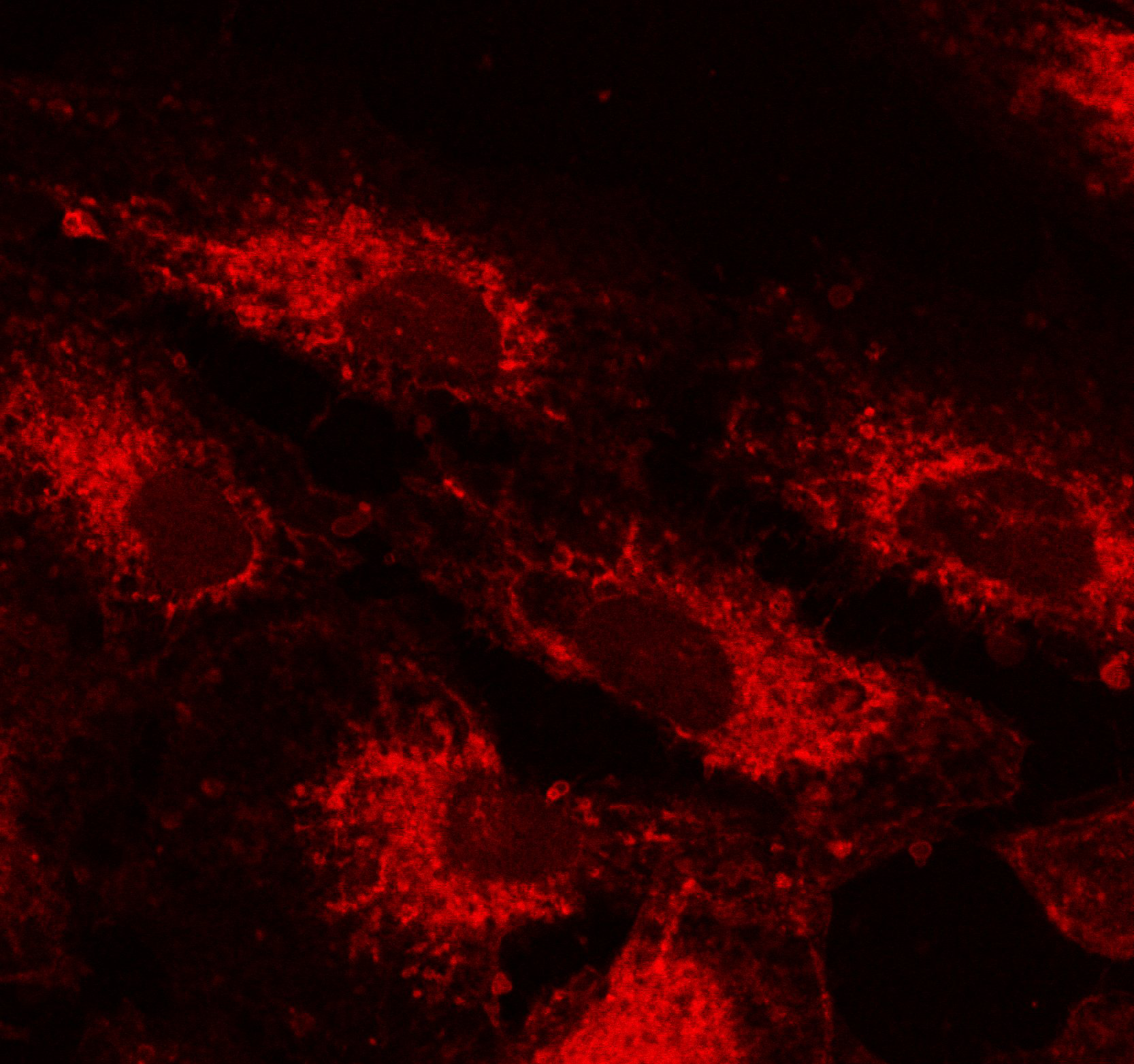

Supplement: Supplementary file 8 — Source data Fig. 3 [file 44319_2026_736_MOESM8_ESM.zip › Figure 3/3D/3D_WT/HK-2 WT_ER tracker_ER tracker.tif]

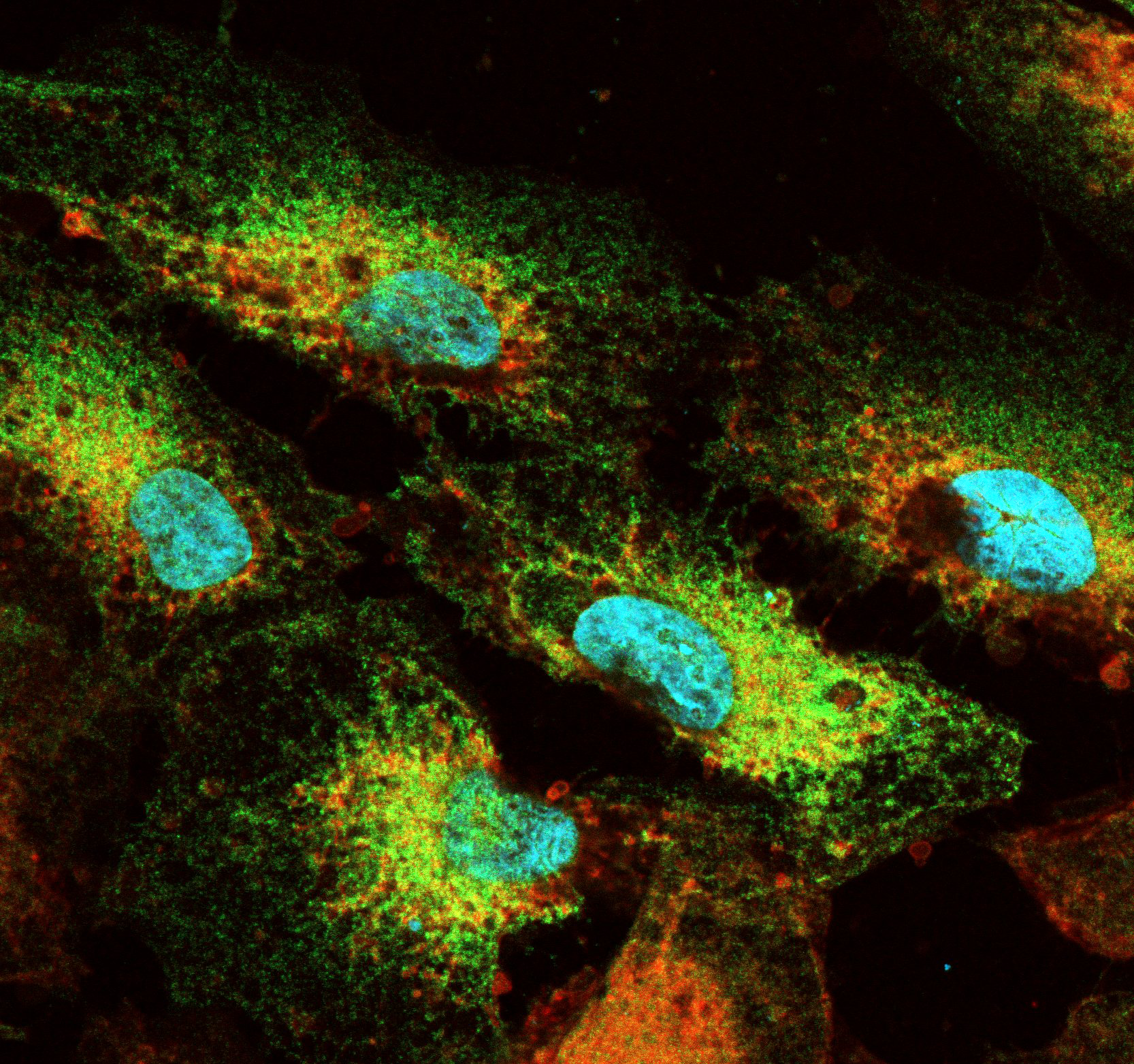

Supplement: Supplementary file 8 — Source data Fig. 3 [file 44319_2026_736_MOESM8_ESM.zip › Figure 3/3D/3D_WT/HK-2 WT_ER tracker_Merged.tif]

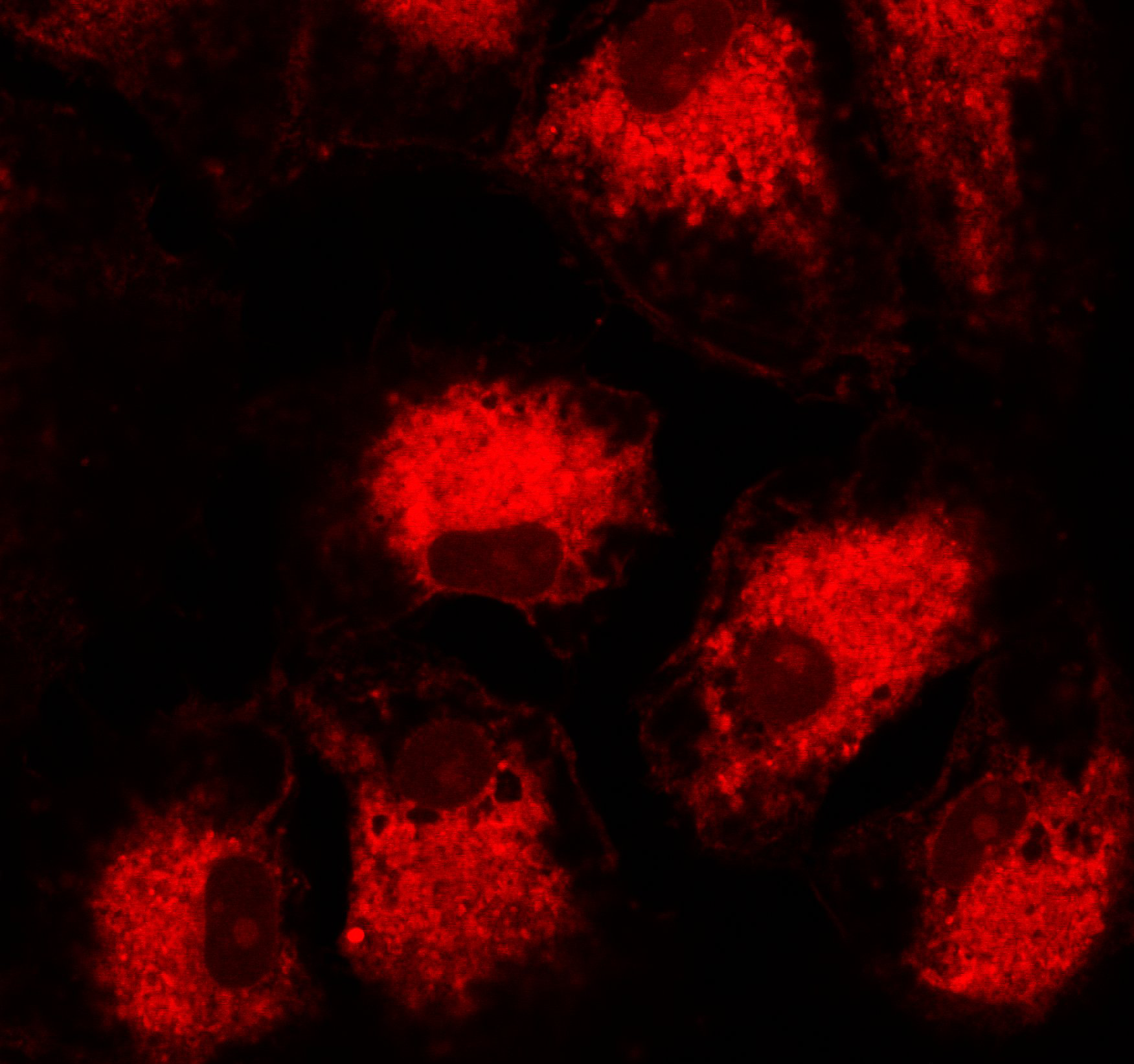

Supplement: Supplementary file 8 — Source data Fig. 3 [file 44319_2026_736_MOESM8_ESM.zip › Figure 3/3D/3D_CTNS KO/HK-2 CTNS KO_ER tracker_ER tracker.tif]

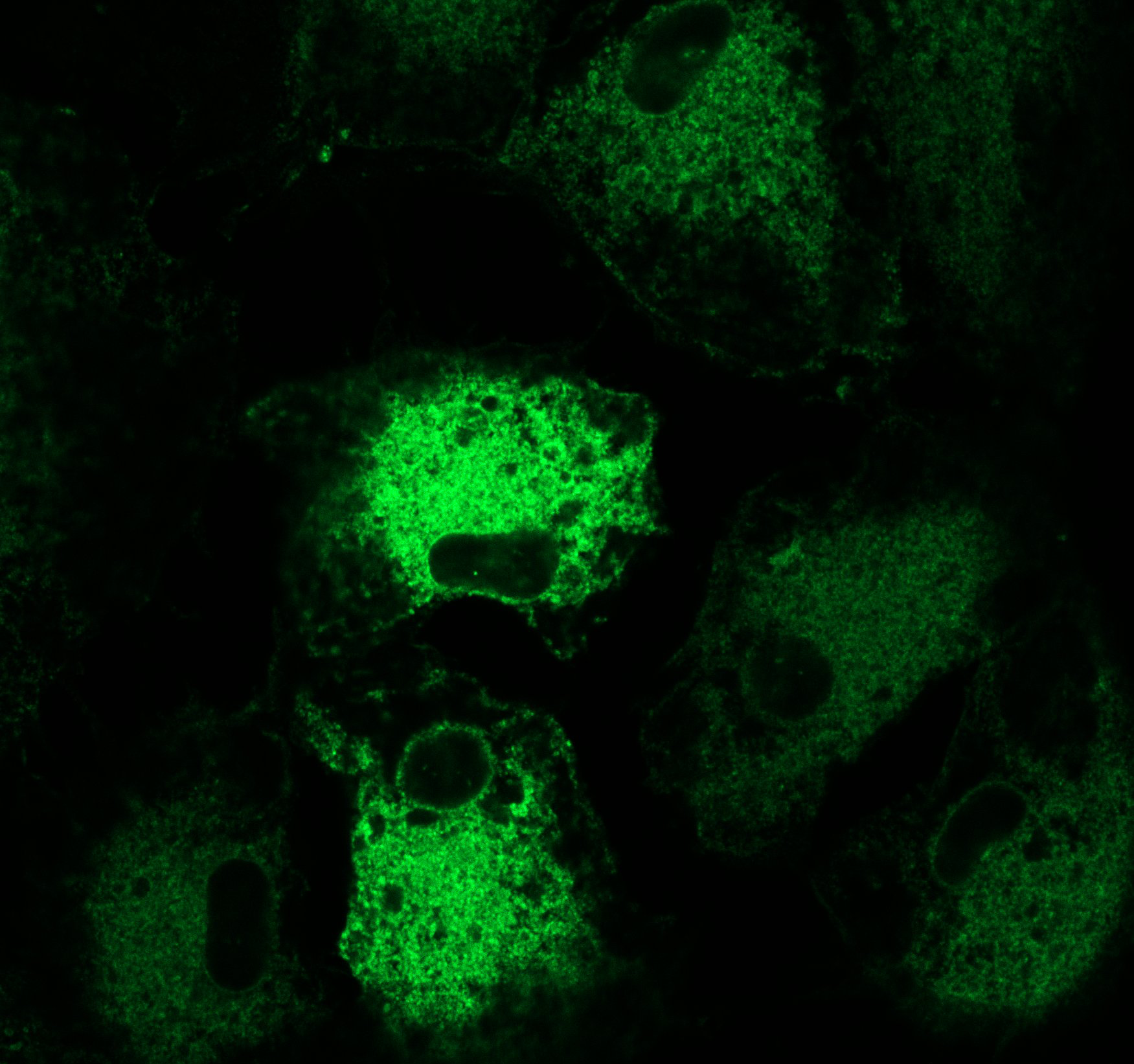

Supplement: Supplementary file 8 — Source data Fig. 3 [file 44319_2026_736_MOESM8_ESM.zip › Figure 3/3D/3D_CTNS KO/HK-2 CTNS KO_ER tracker_NHE3 GFP.tif]

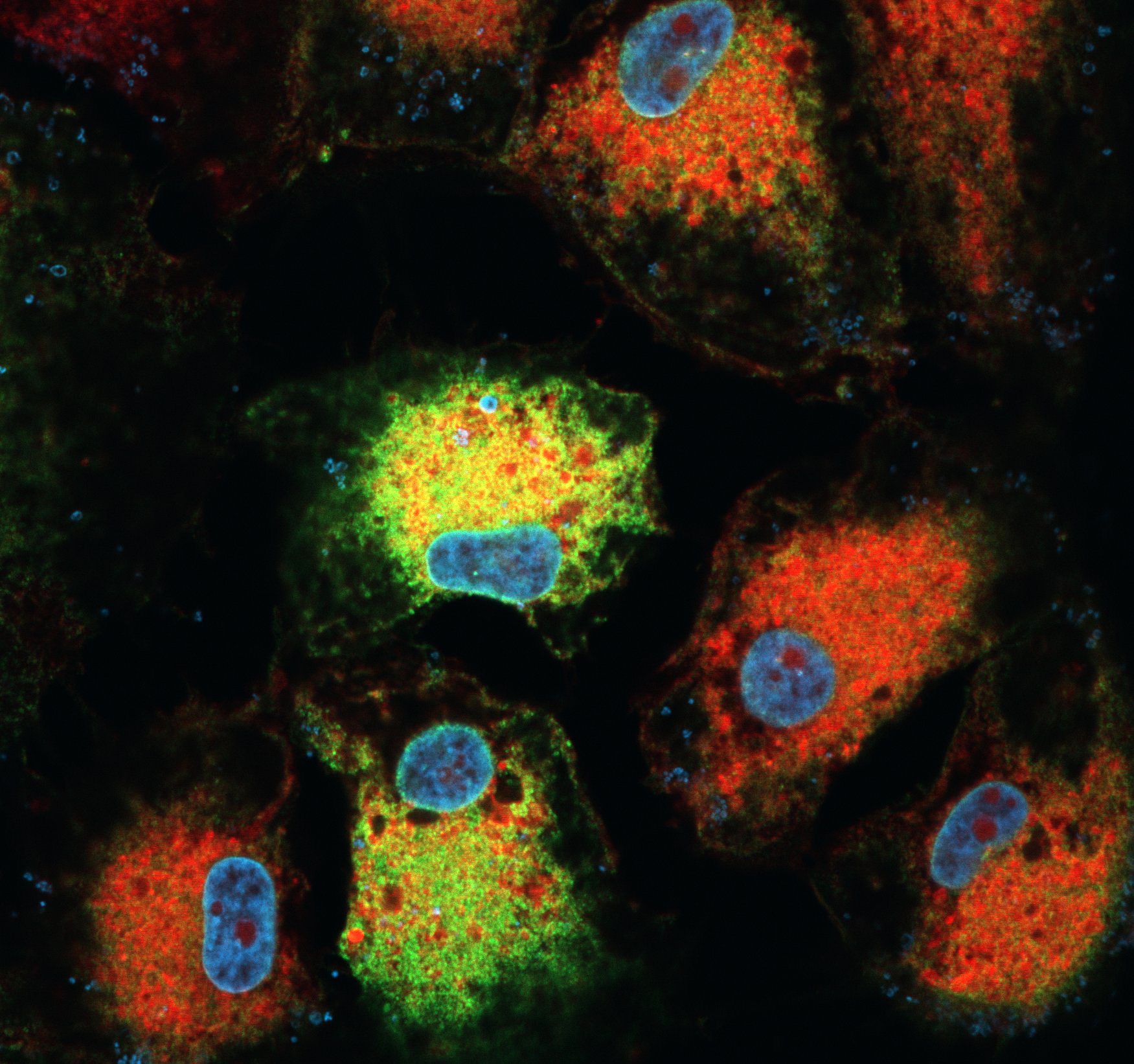

Supplement: Supplementary file 8 — Source data Fig. 3 [file 44319_2026_736_MOESM8_ESM.zip › Figure 3/3D/3D_CTNS KO/HK-2 CTNS KO_ER tracker_Merged.tif]

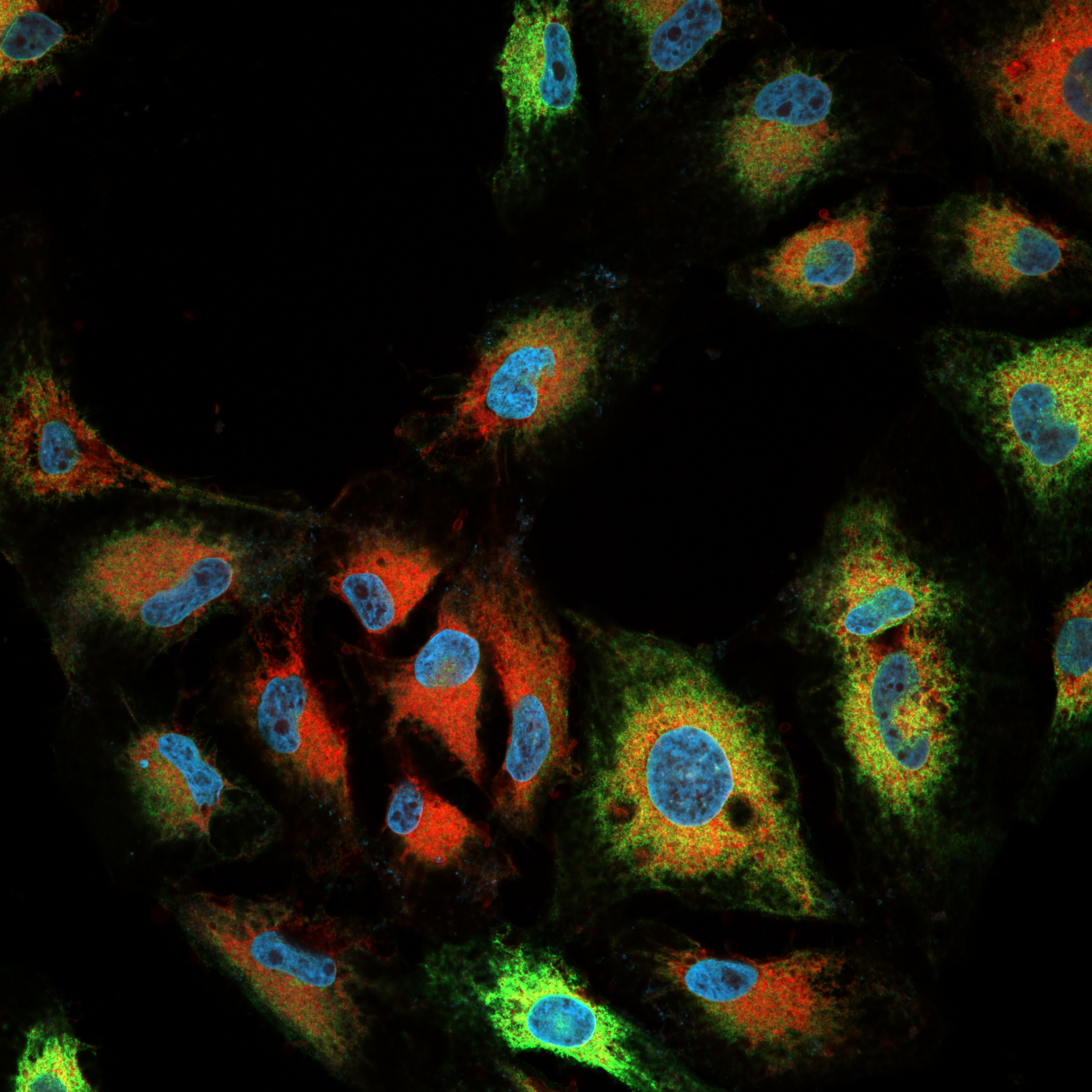

Supplement: Supplementary file 8 — Source data Fig. 3 [file 44319_2026_736_MOESM8_ESM.zip › Figure 3/3D/3D_WT_Replicate/HK-2 WT NHE3-GFP ER tracker_Merged.tif]

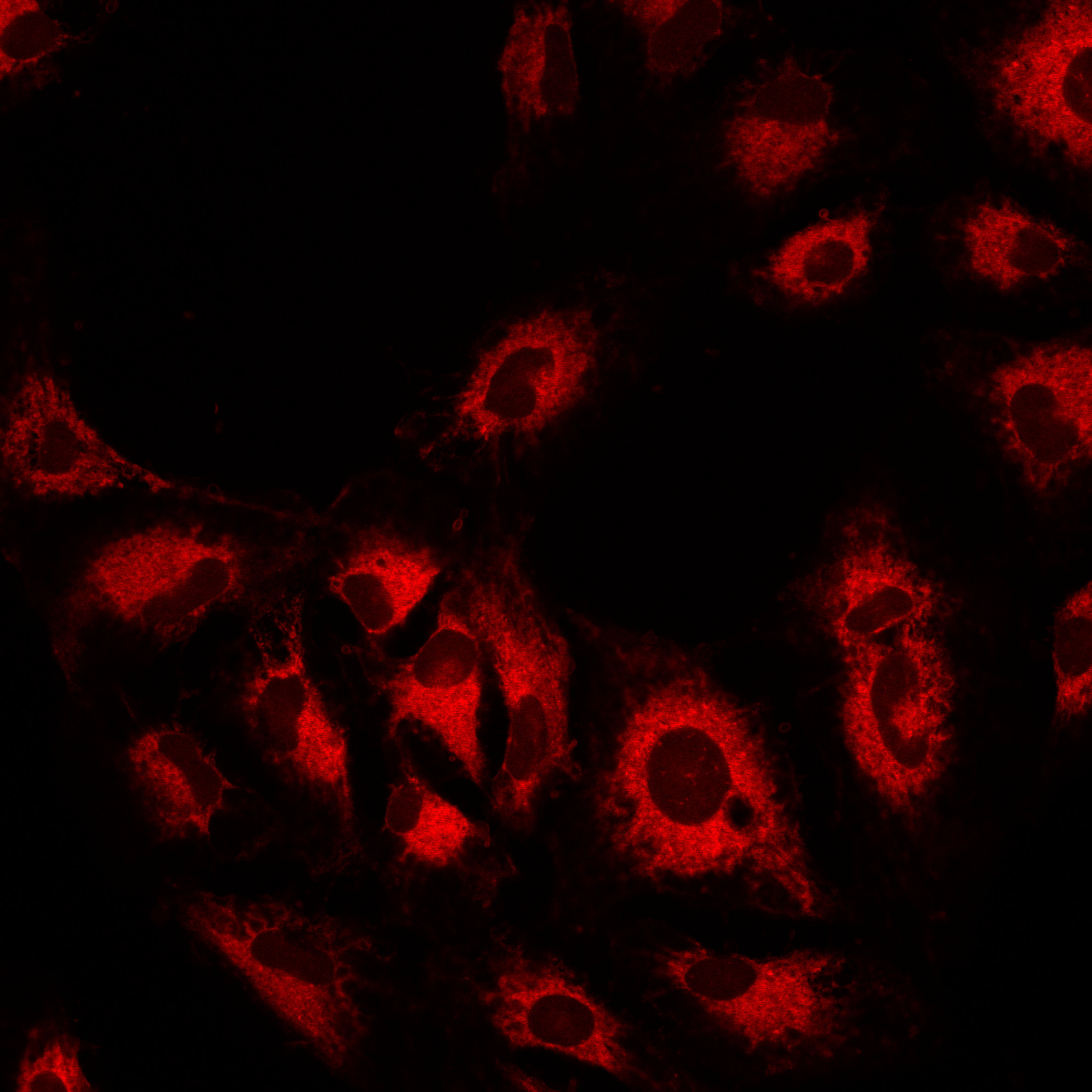

Supplement: Supplementary file 8 — Source data Fig. 3 [file 44319_2026_736_MOESM8_ESM.zip › Figure 3/3D/3D_WT_Replicate/HK-2 WT NHE3-GFP ER tracker_ER Tracker.tif]

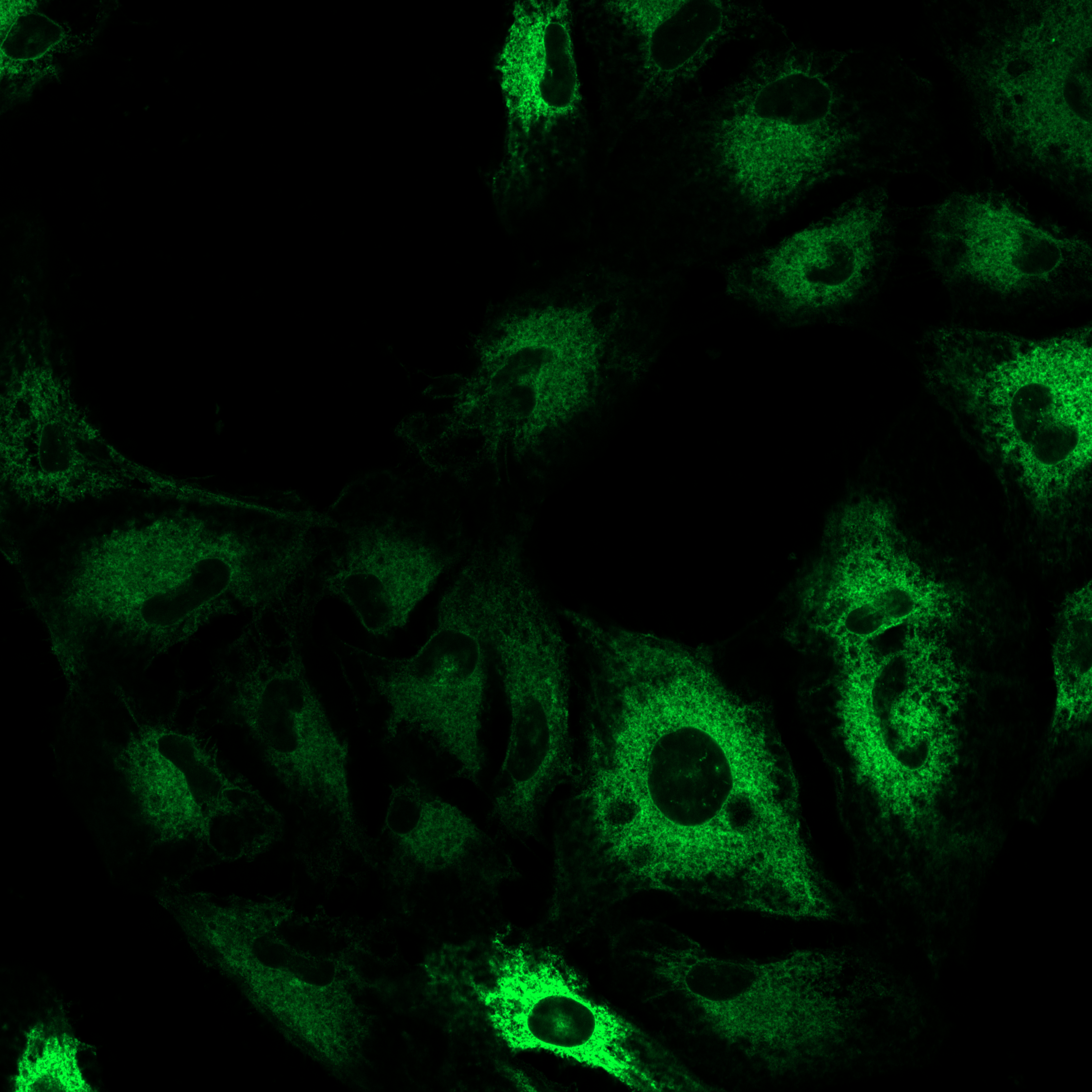

Supplement: Supplementary file 8 — Source data Fig. 3 [file 44319_2026_736_MOESM8_ESM.zip › Figure 3/3D/3D_WT_Replicate/HK-2 WT NHE3-GFP ER tracker_NHE3 GFP.tif]

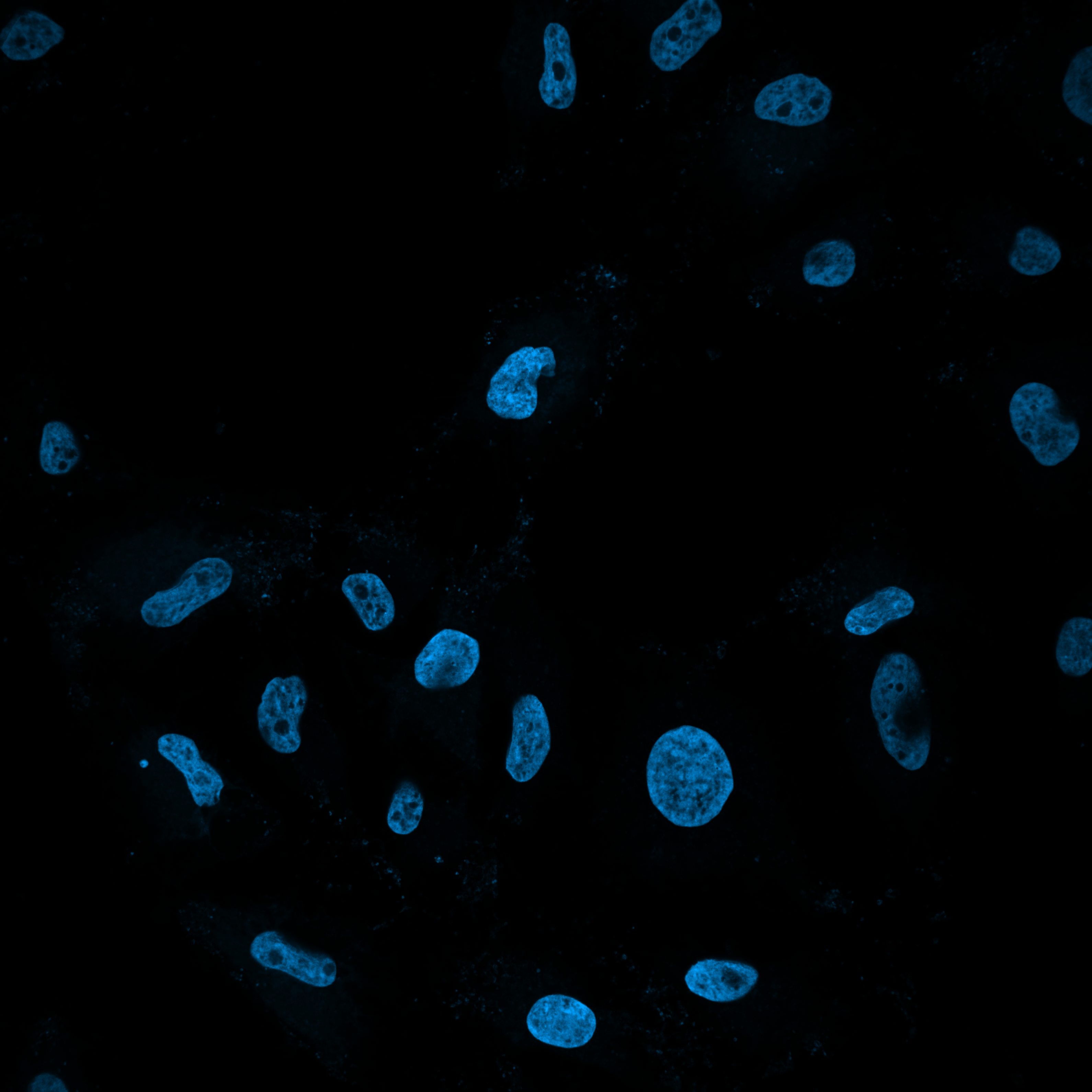

Supplement: Supplementary file 8 — Source data Fig. 3 [file 44319_2026_736_MOESM8_ESM.zip › Figure 3/3D/3D_WT_Replicate/HK-2 WT NHE3-GFP ER tracker_DAPI.tif]

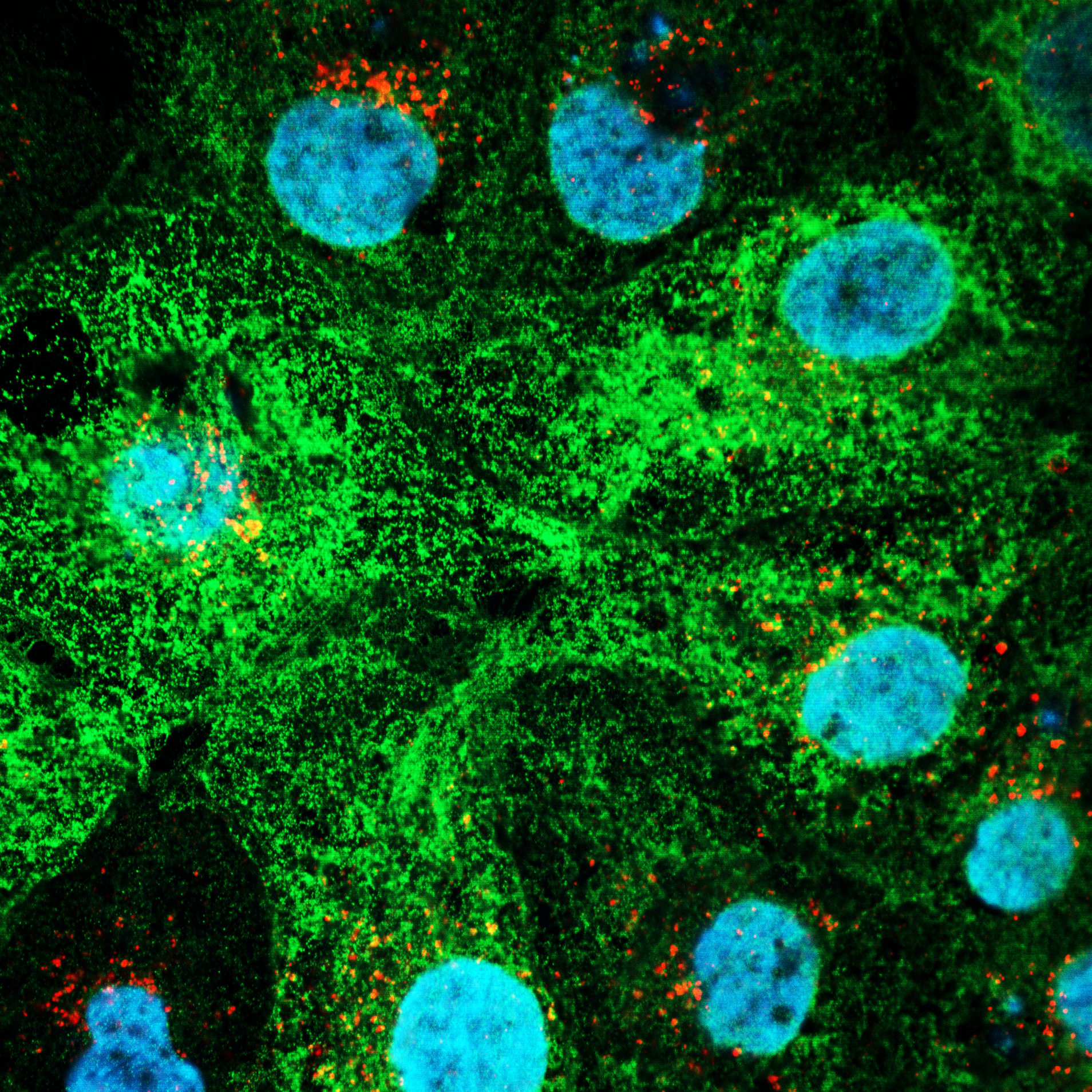

Supplement: Supplementary file 8 — Source data Fig. 3 [file 44319_2026_736_MOESM8_ESM.zip › Figure 3/3C /3C_CTNS KO/HK-2 CTNS KO_EEA1_Merged.tif]

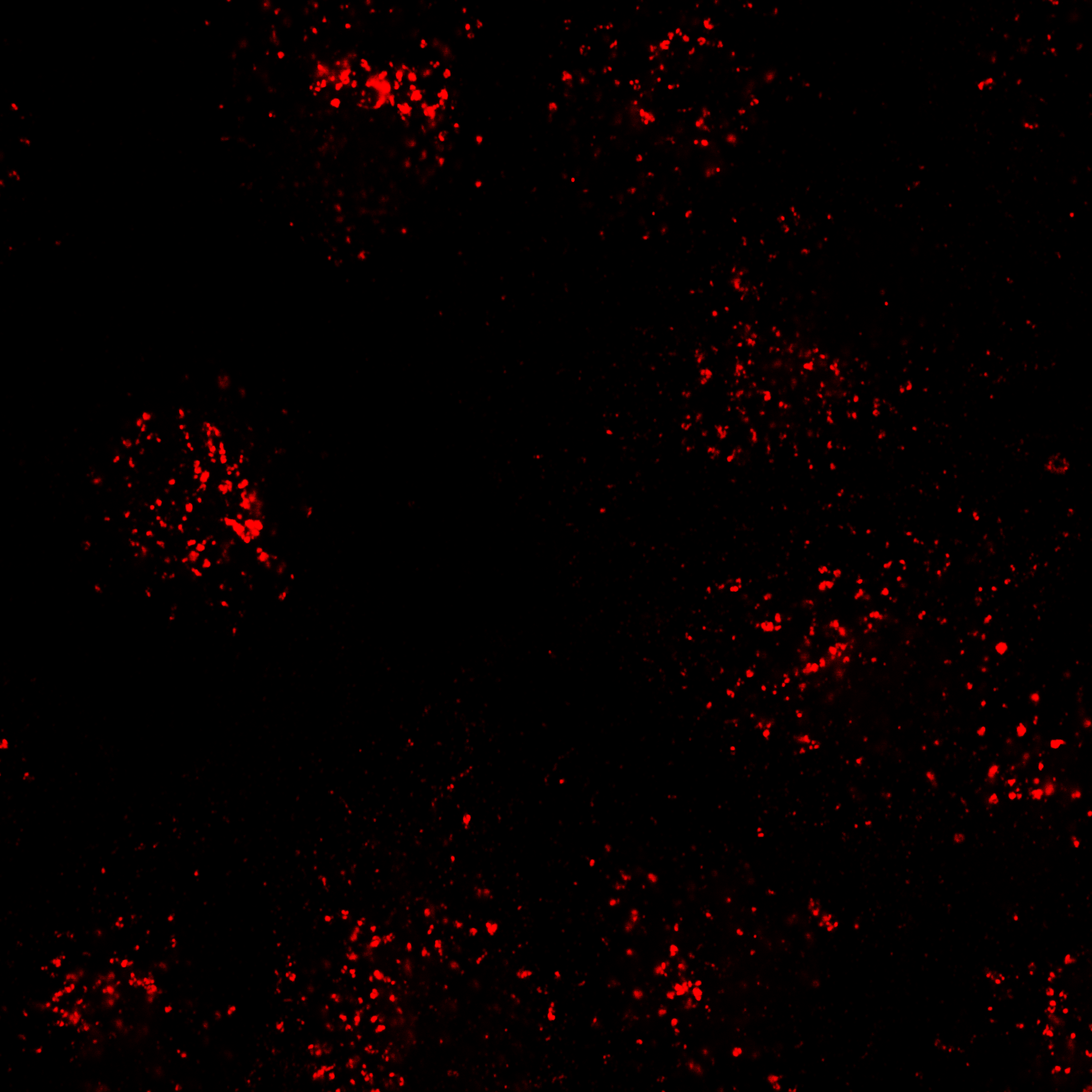

Supplement: Supplementary file 8 — Source data Fig. 3 [file 44319_2026_736_MOESM8_ESM.zip › Figure 3/3C /3C_CTNS KO/HK-2 CTNS KO_EEA1_EEA1.tif]

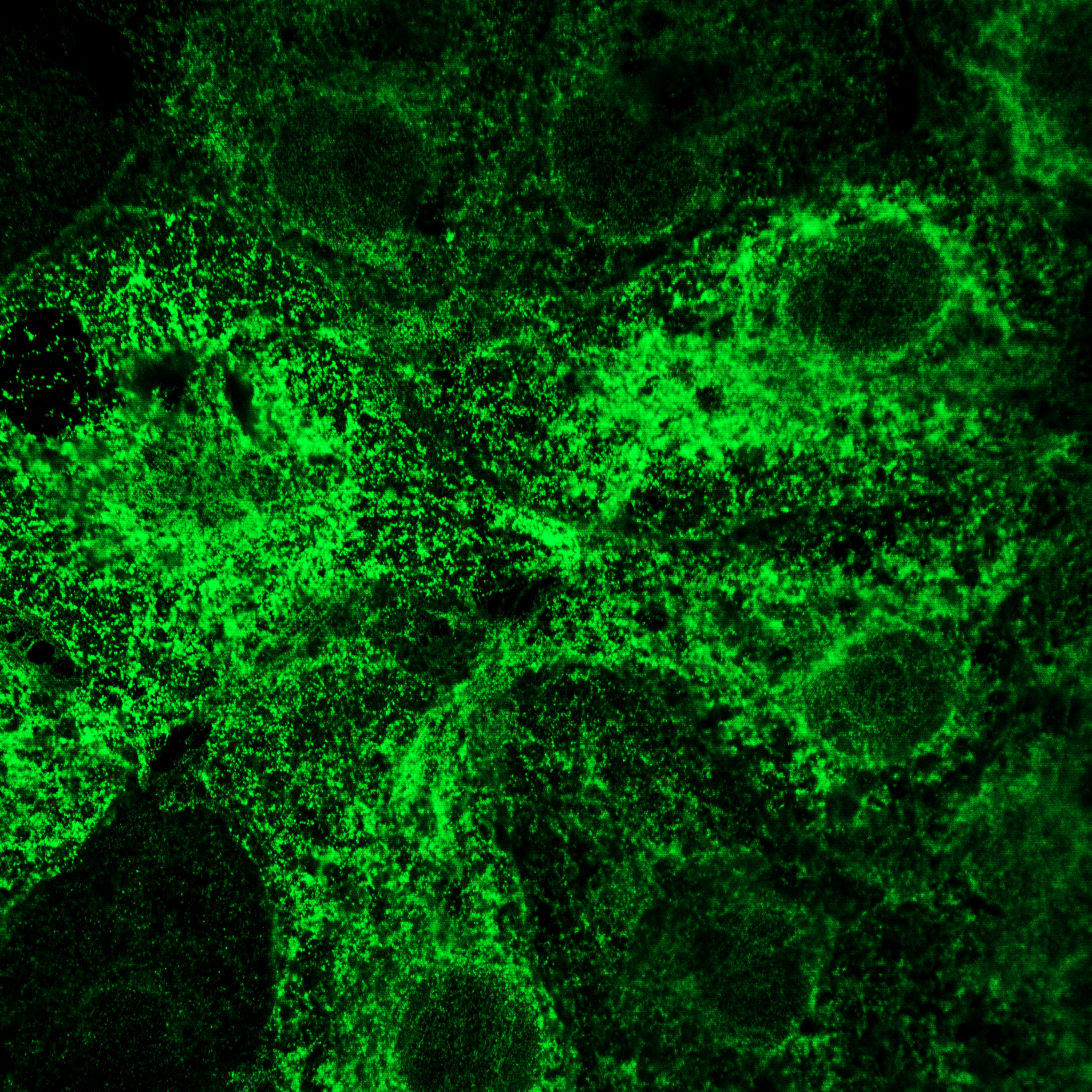

Supplement: Supplementary file 8 — Source data Fig. 3 [file 44319_2026_736_MOESM8_ESM.zip › Figure 3/3C /3C_CTNS KO/HK-2 CTNS KO_EEA1_NHE3-GFP.tif]

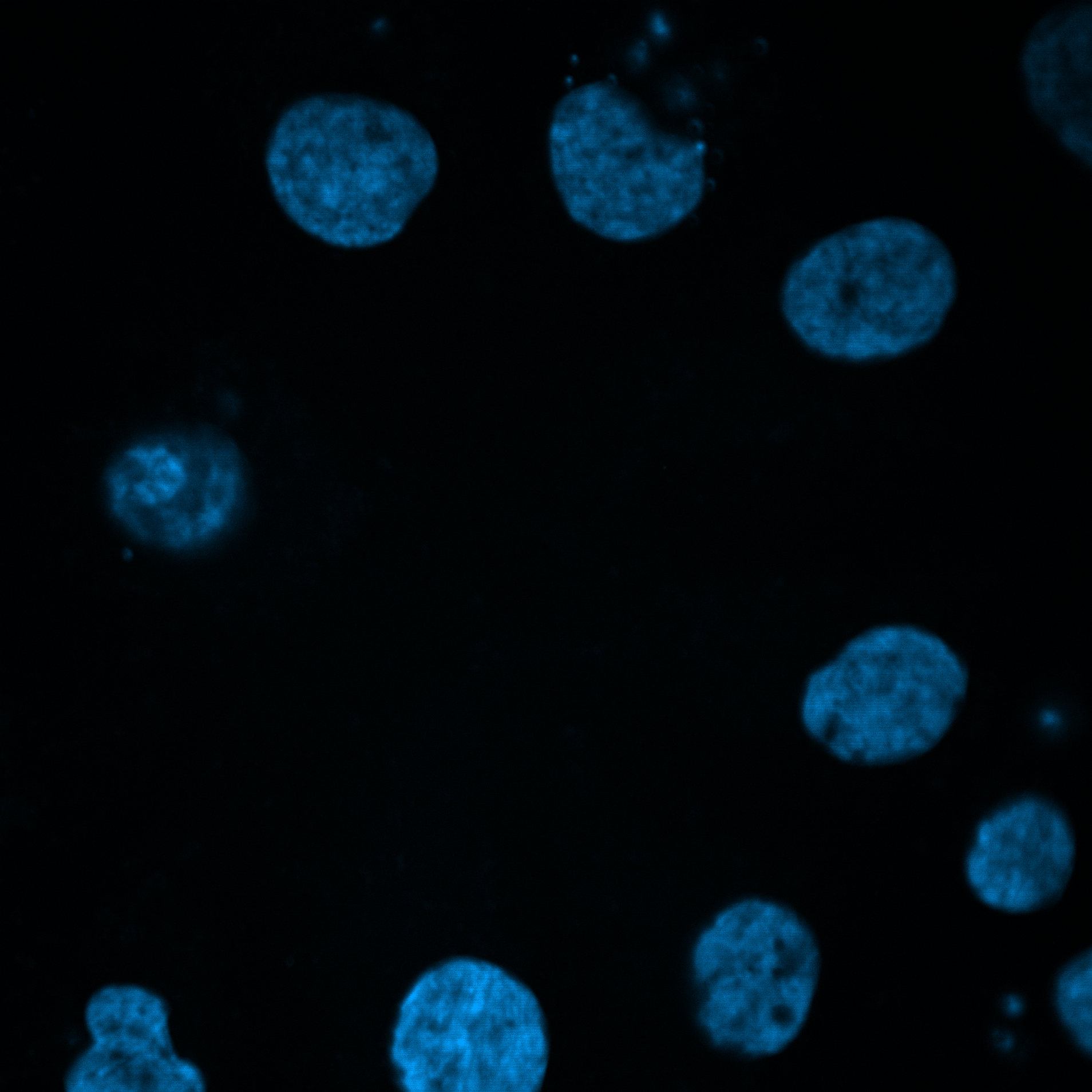

Supplement: Supplementary file 8 — Source data Fig. 3 [file 44319_2026_736_MOESM8_ESM.zip › Figure 3/3C /3C_CTNS KO/HK-2 CTNS KO_EEA1_DAPI.tif]

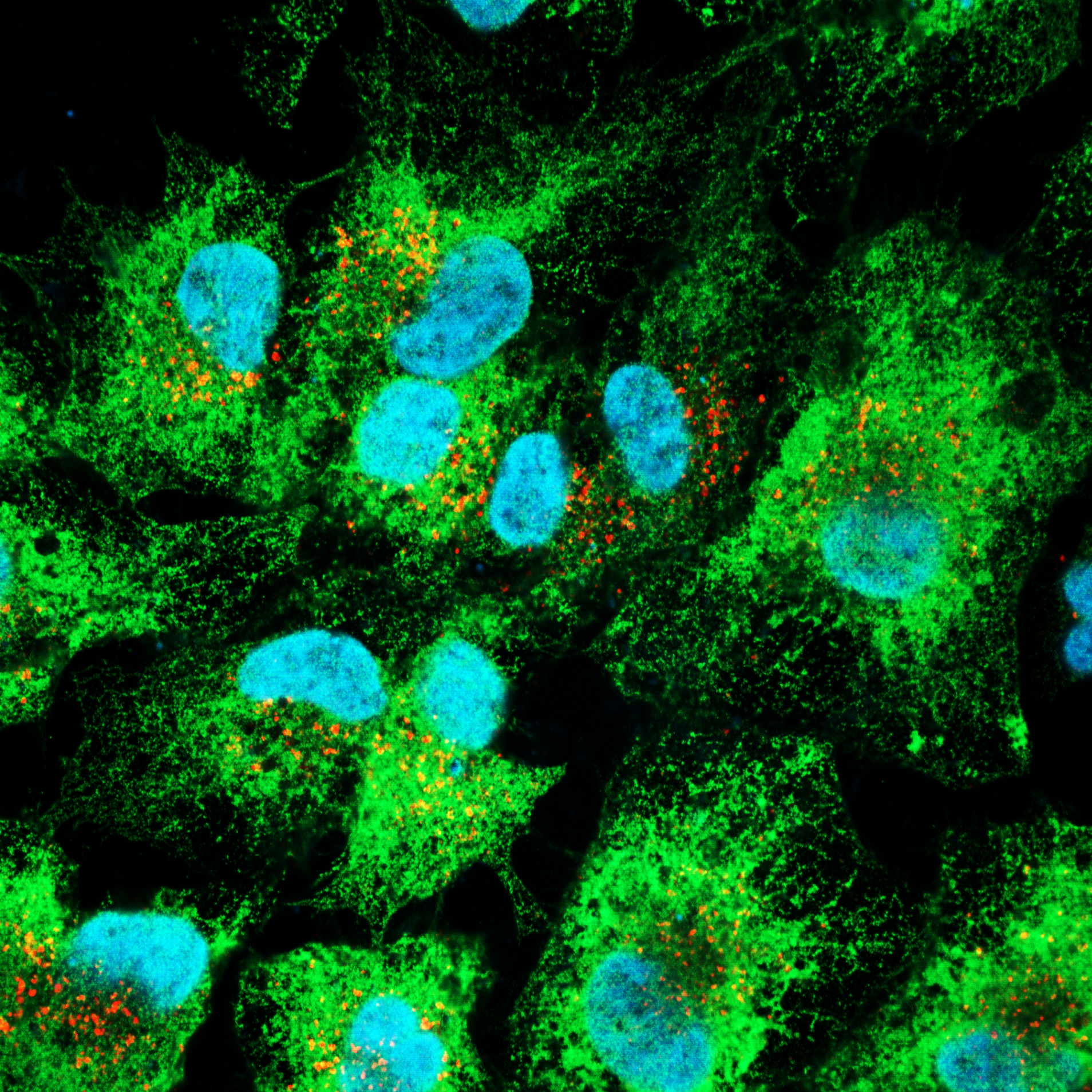

Supplement: Supplementary file 8 — Source data Fig. 3 [file 44319_2026_736_MOESM8_ESM.zip › Figure 3/3C /3C_WT/HK-2 WT EEA1_Merged.tif]

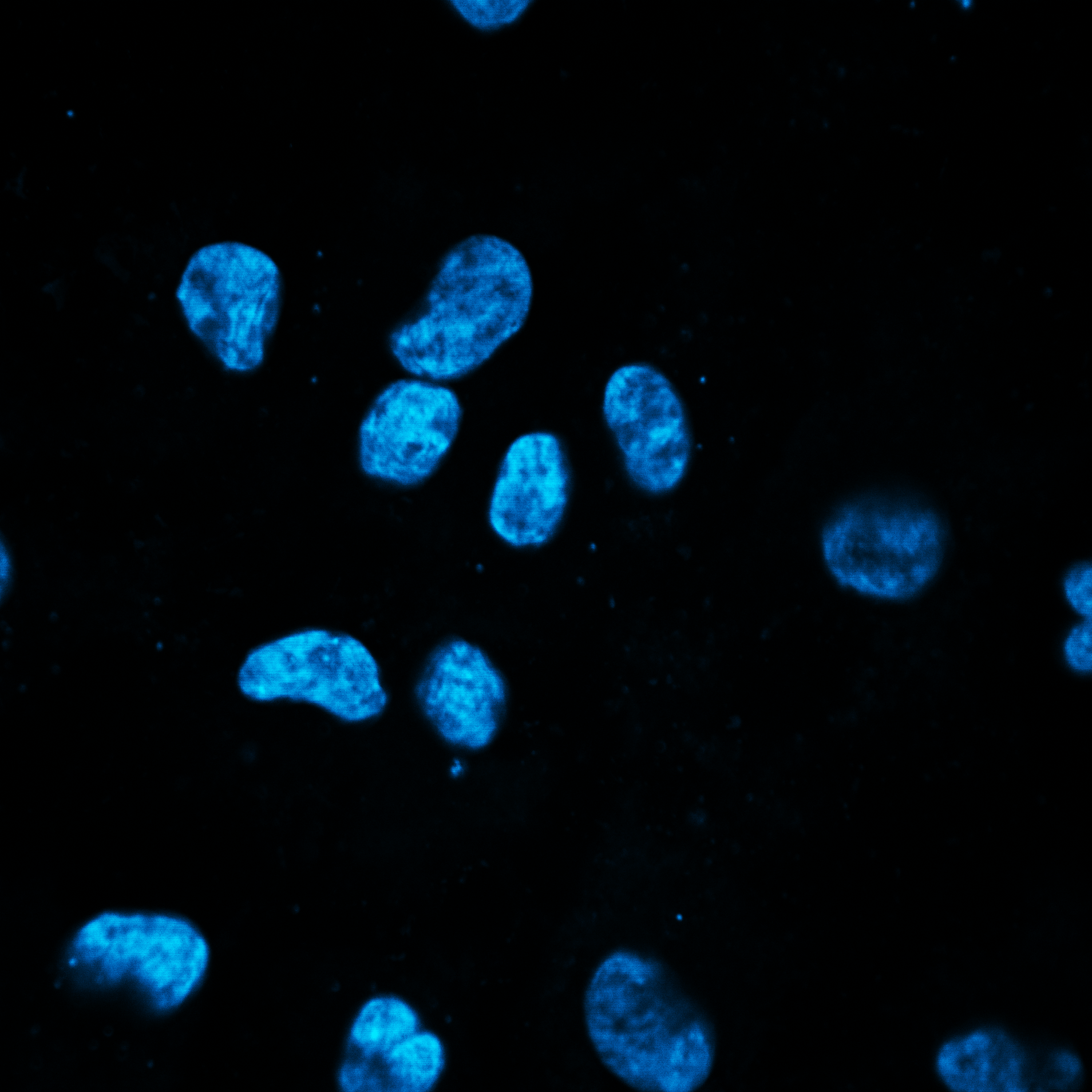

Supplement: Supplementary file 8 — Source data Fig. 3 [file 44319_2026_736_MOESM8_ESM.zip › Figure 3/3C /3C_WT/HK-2 WT EEA1_DAPI.tif]

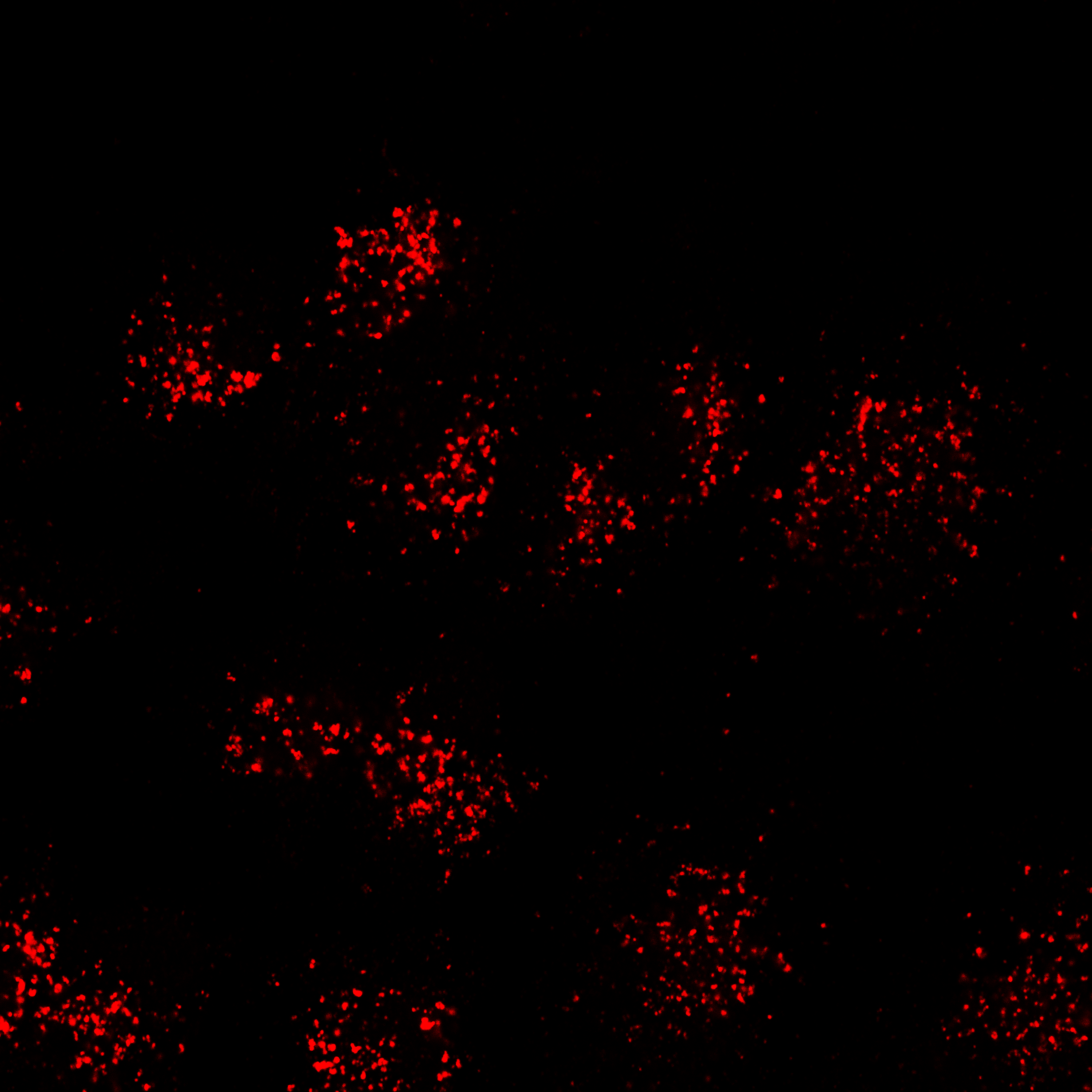

Supplement: Supplementary file 8 — Source data Fig. 3 [file 44319_2026_736_MOESM8_ESM.zip › Figure 3/3C /3C_WT/HK-2 WT EEA1_EEA1.tif]

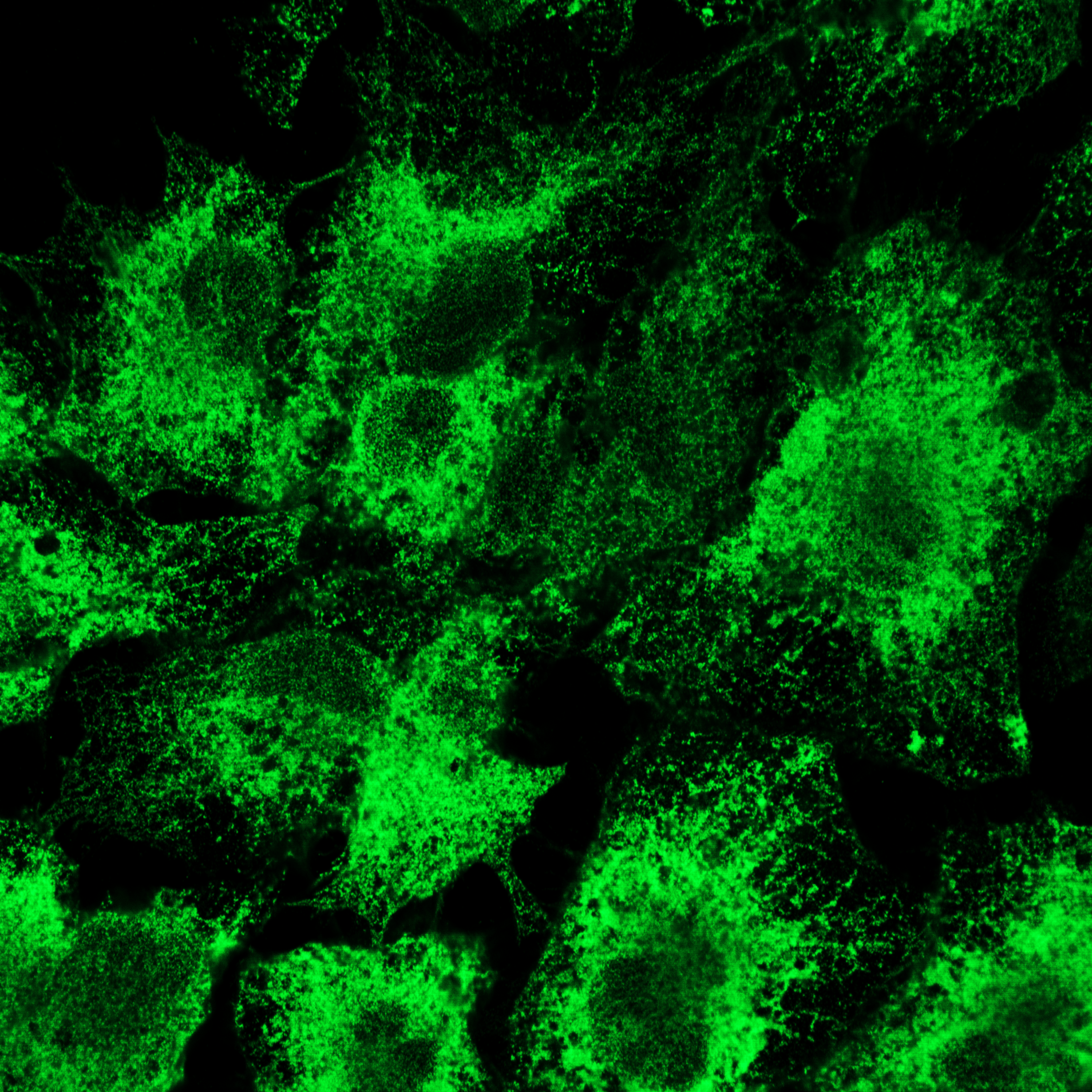

Supplement: Supplementary file 8 — Source data Fig. 3 [file 44319_2026_736_MOESM8_ESM.zip › Figure 3/3C /3C_WT/HK-2 WT EEA1_NHE3-GFP .tif]

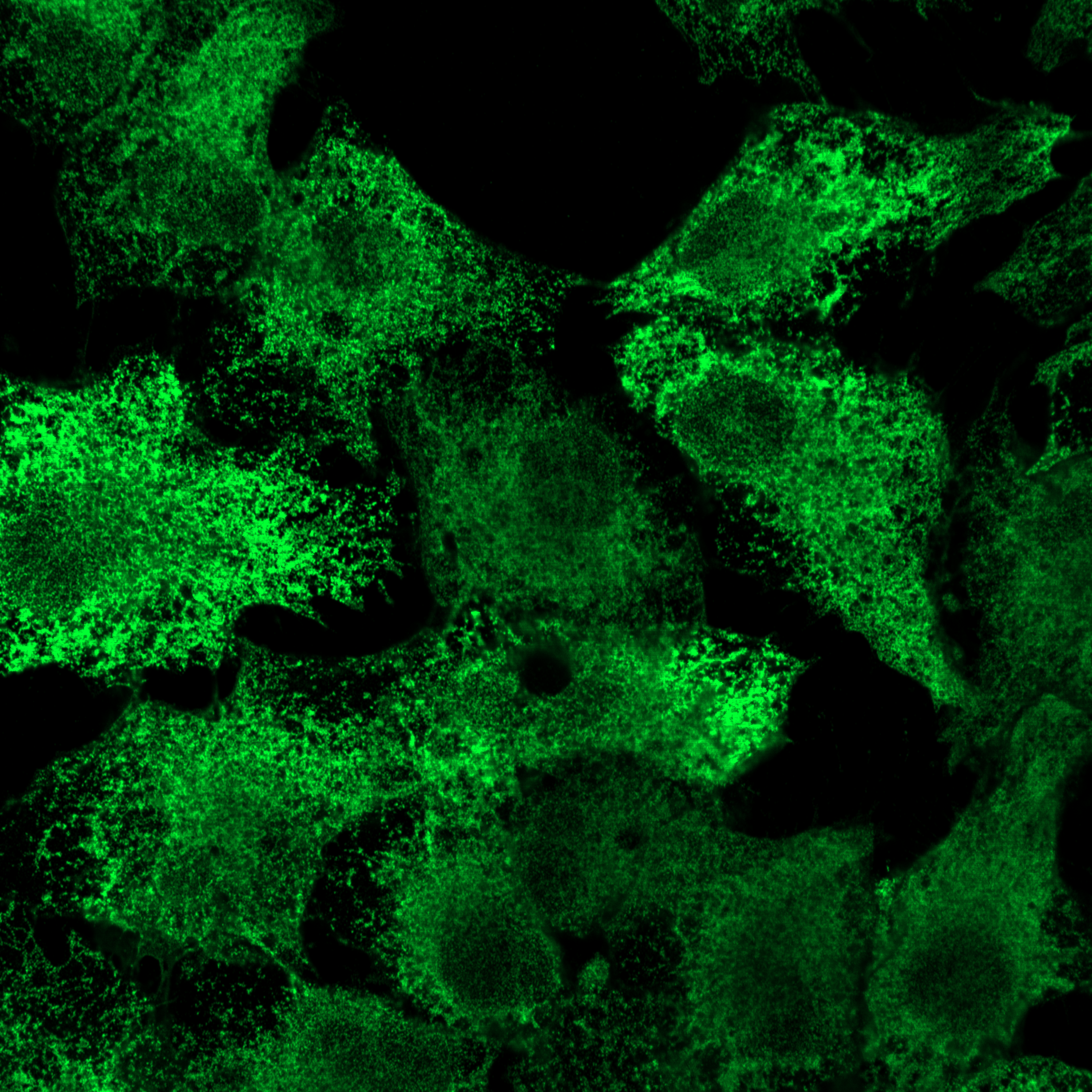

Supplement: Supplementary file 8 — Source data Fig. 3 [file 44319_2026_736_MOESM8_ESM.zip › Figure 3/3C /3C_WT_Replicate/NHE3-GFP HK-2 WT EEA1_NHE3-GFP.tif]

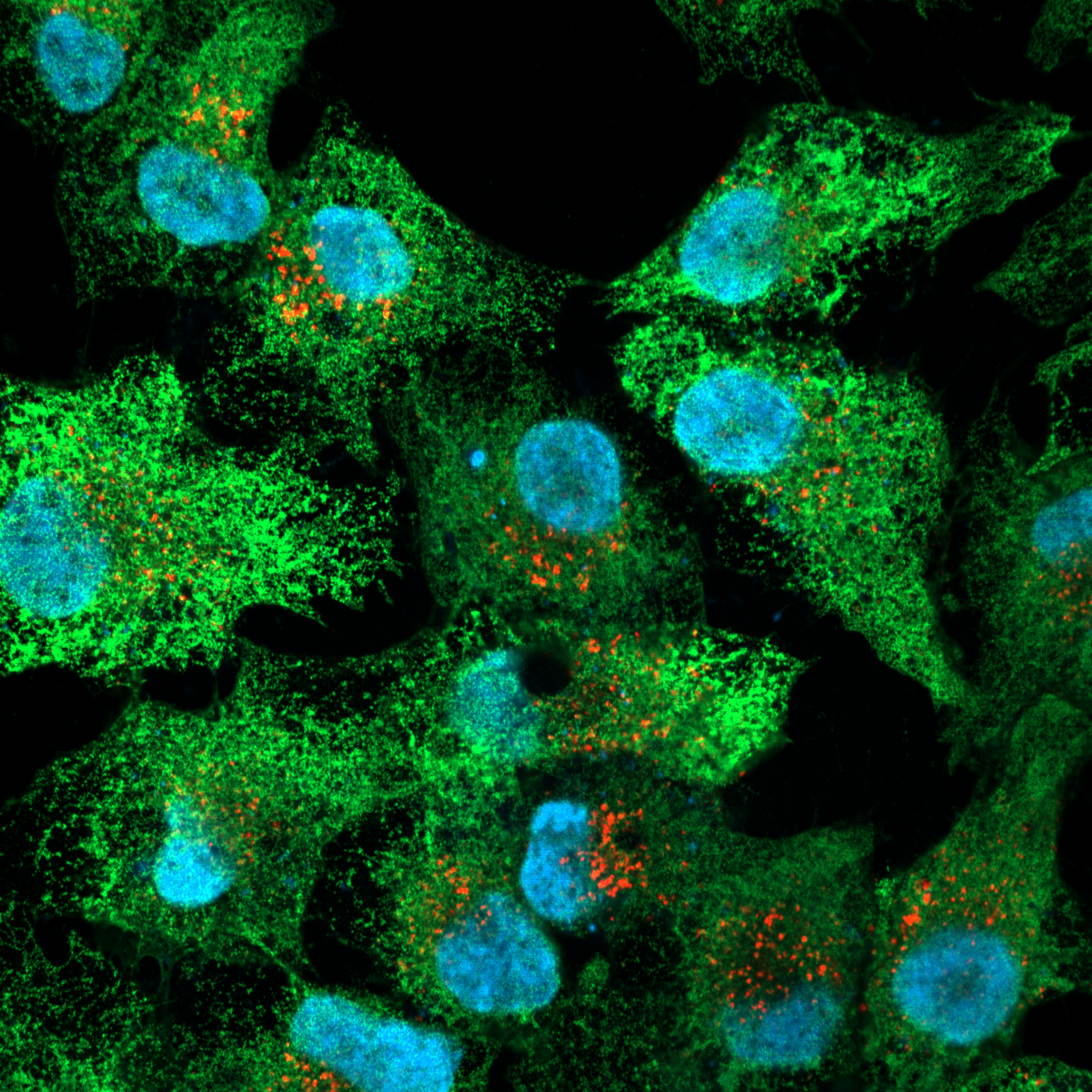

Supplement: Supplementary file 8 — Source data Fig. 3 [file 44319_2026_736_MOESM8_ESM.zip › Figure 3/3C /3C_WT_Replicate/NHE3-GFP HK-2 WT EEA1_Merged.tif]

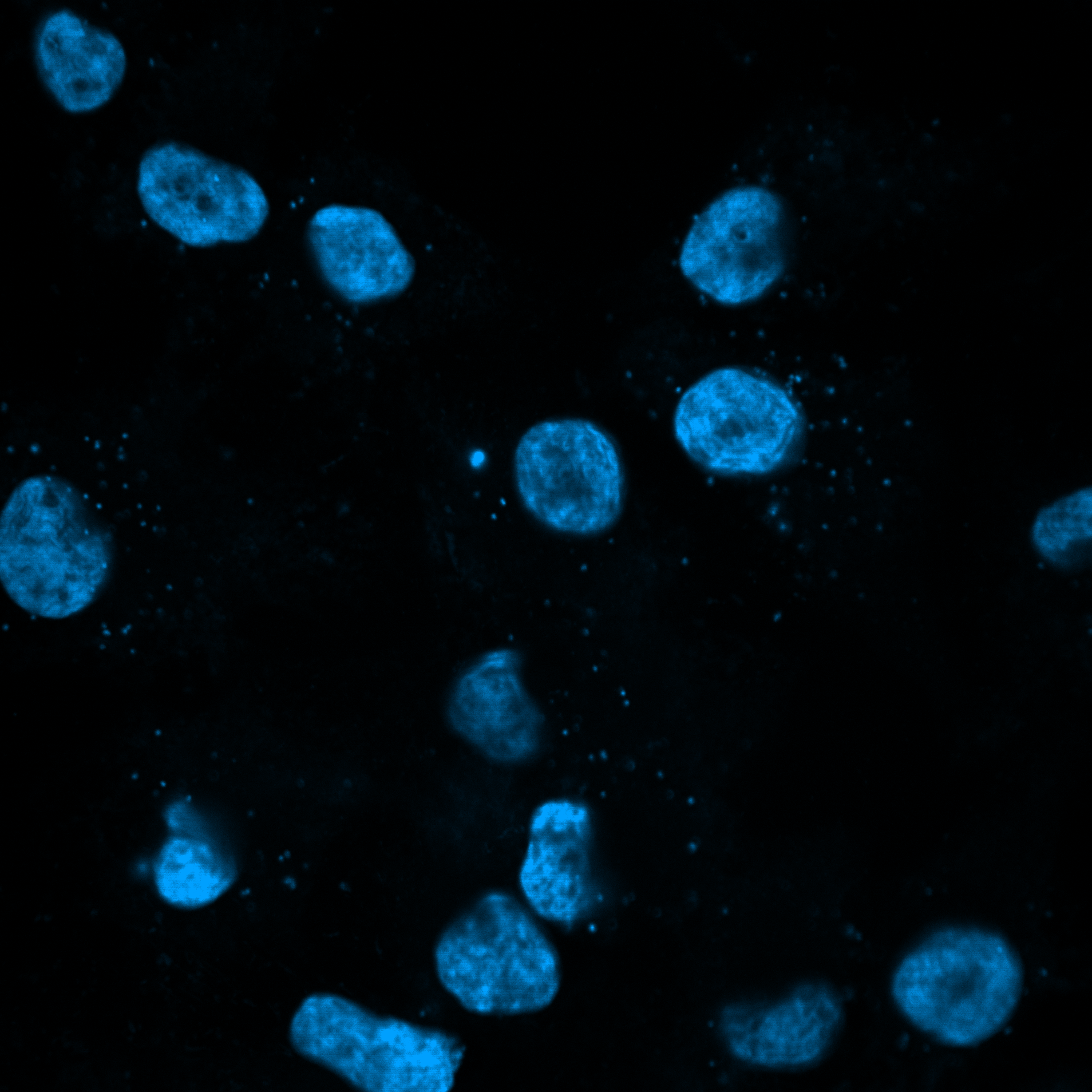

Supplement: Supplementary file 8 — Source data Fig. 3 [file 44319_2026_736_MOESM8_ESM.zip › Figure 3/3C /3C_WT_Replicate/NHE3-GFP HK-2 WT EEA1_DAPI.tif]

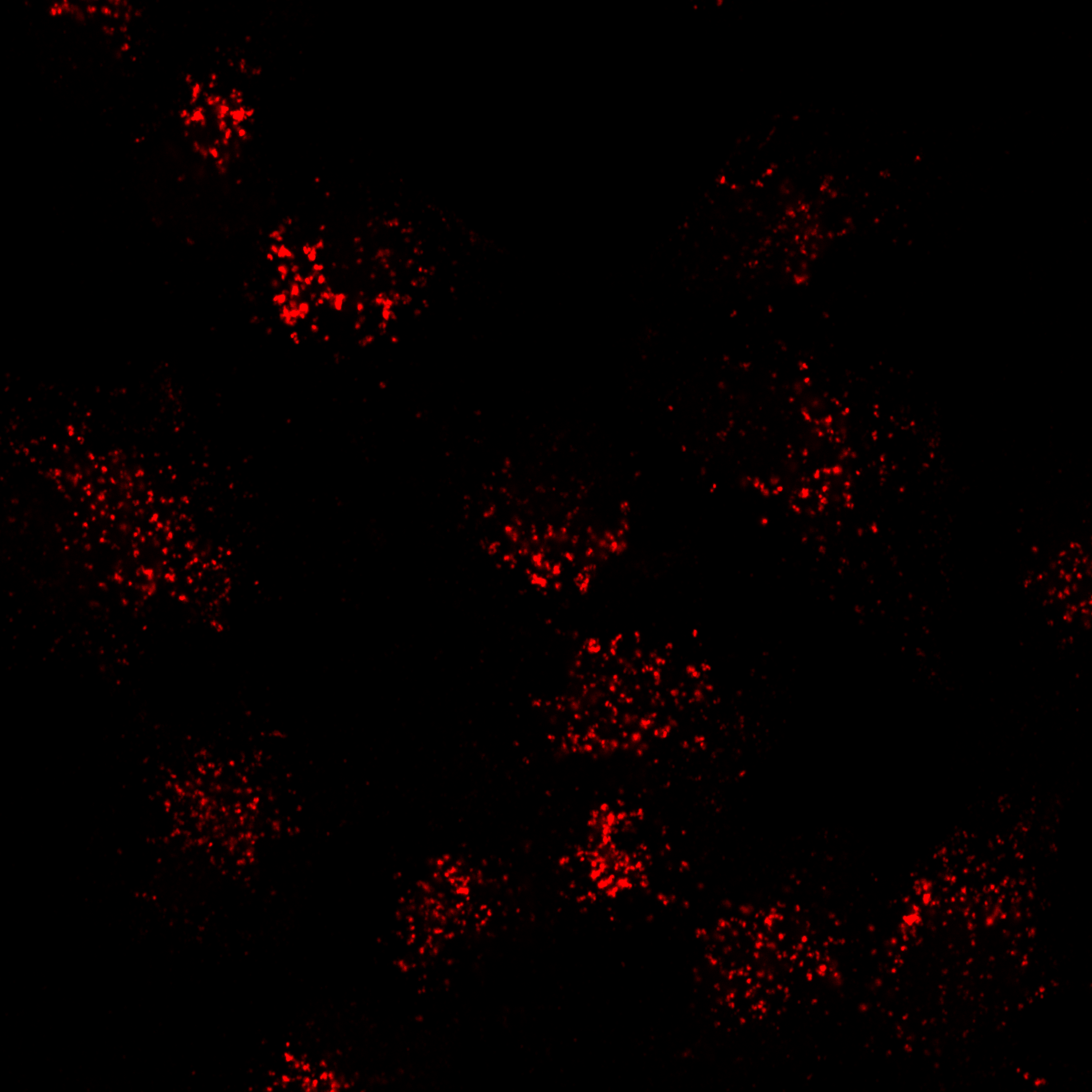

Supplement: Supplementary file 8 — Source data Fig. 3 [file 44319_2026_736_MOESM8_ESM.zip › Figure 3/3C /3C_WT_Replicate/NHE3-GFP HK-2 WT EEA1_EEA1.tif]

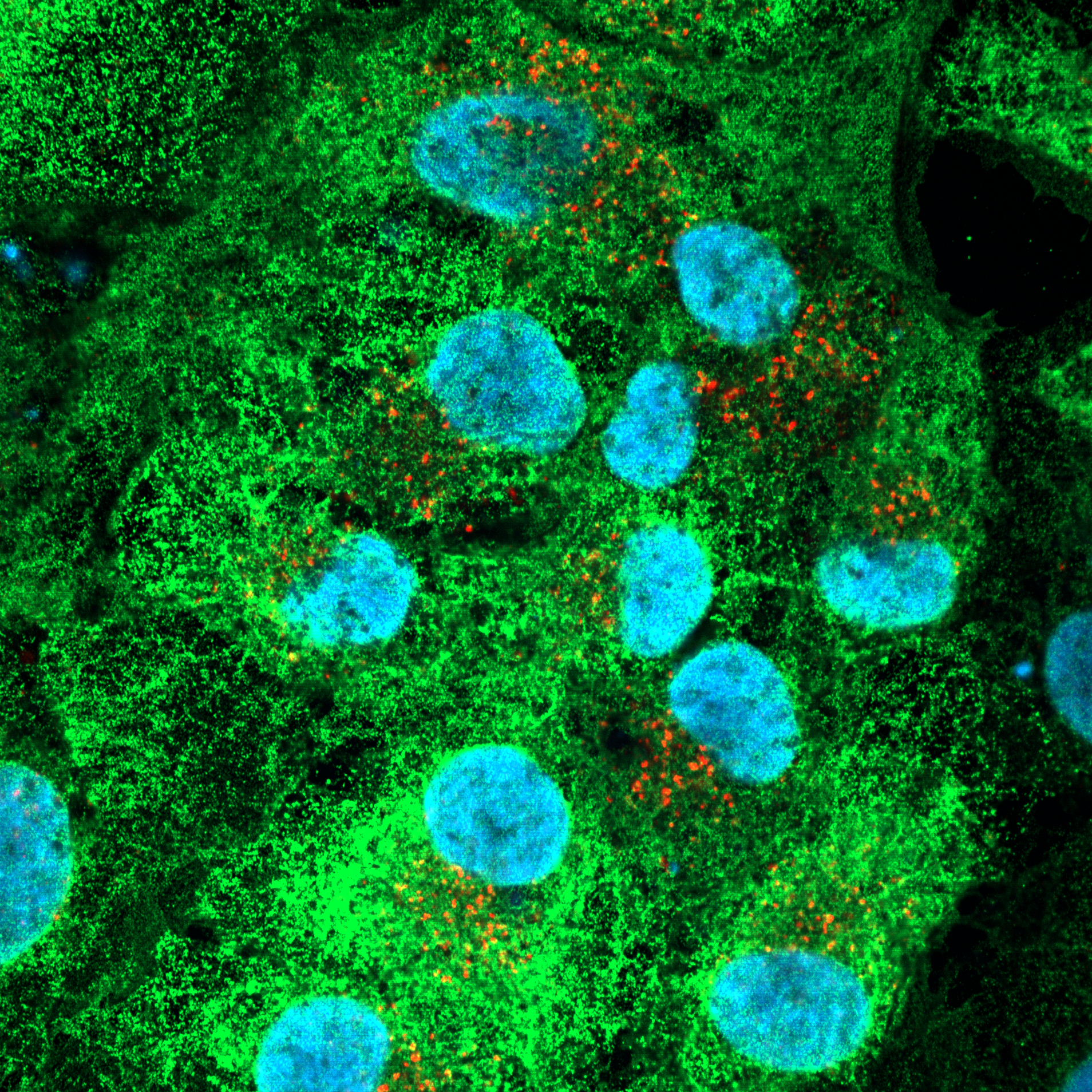

Supplement: Supplementary file 8 — Source data Fig. 3 [file 44319_2026_736_MOESM8_ESM.zip › Figure 3/3C /3C_CTNS KO_Replicate/NHE3-GFP HK-2 CTNS KO_EEA1_Merged.tif]

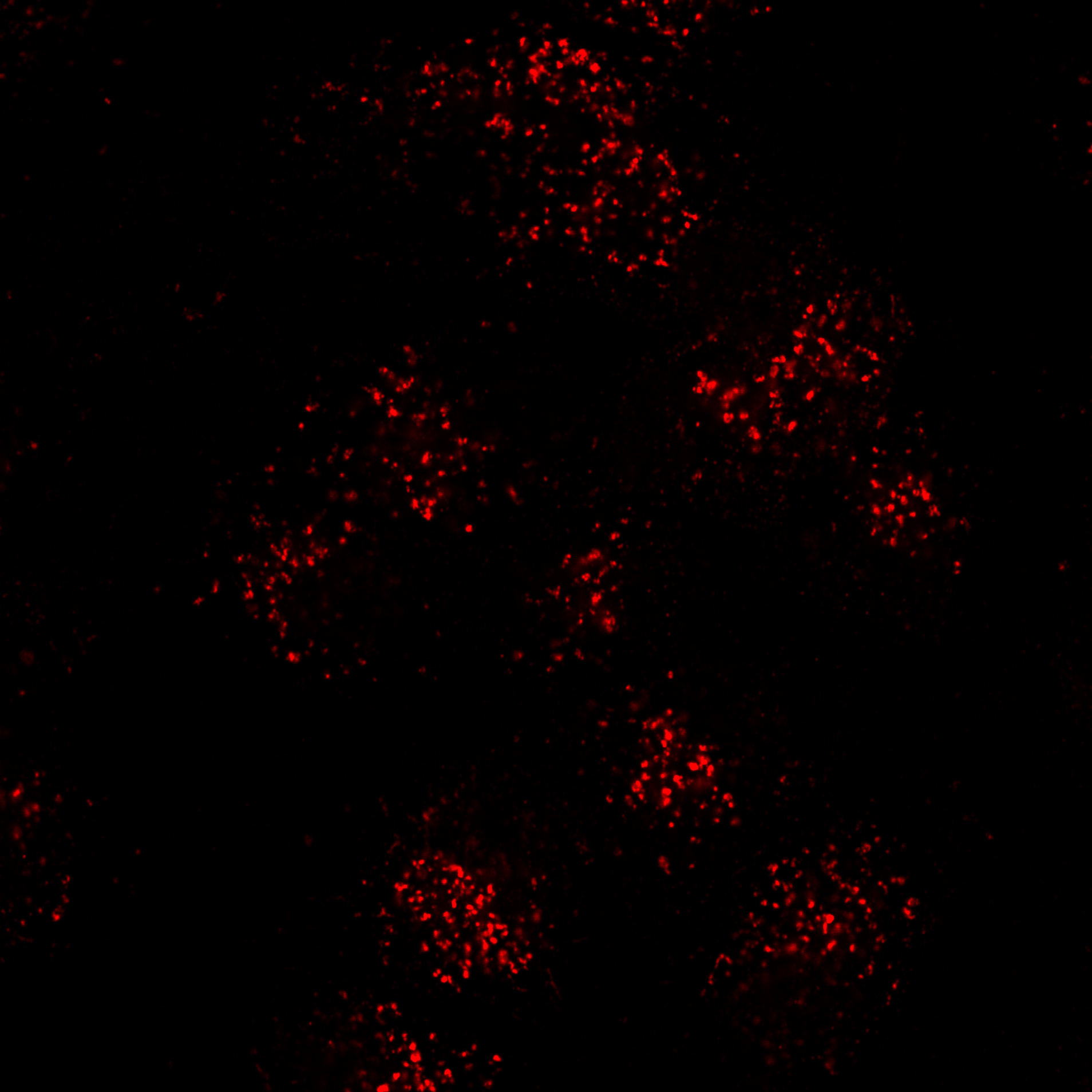

Supplement: Supplementary file 8 — Source data Fig. 3 [file 44319_2026_736_MOESM8_ESM.zip › Figure 3/3C /3C_CTNS KO_Replicate/NHE3-GFP HK-2 CTNS KO_EEA1_EEA1.tif]

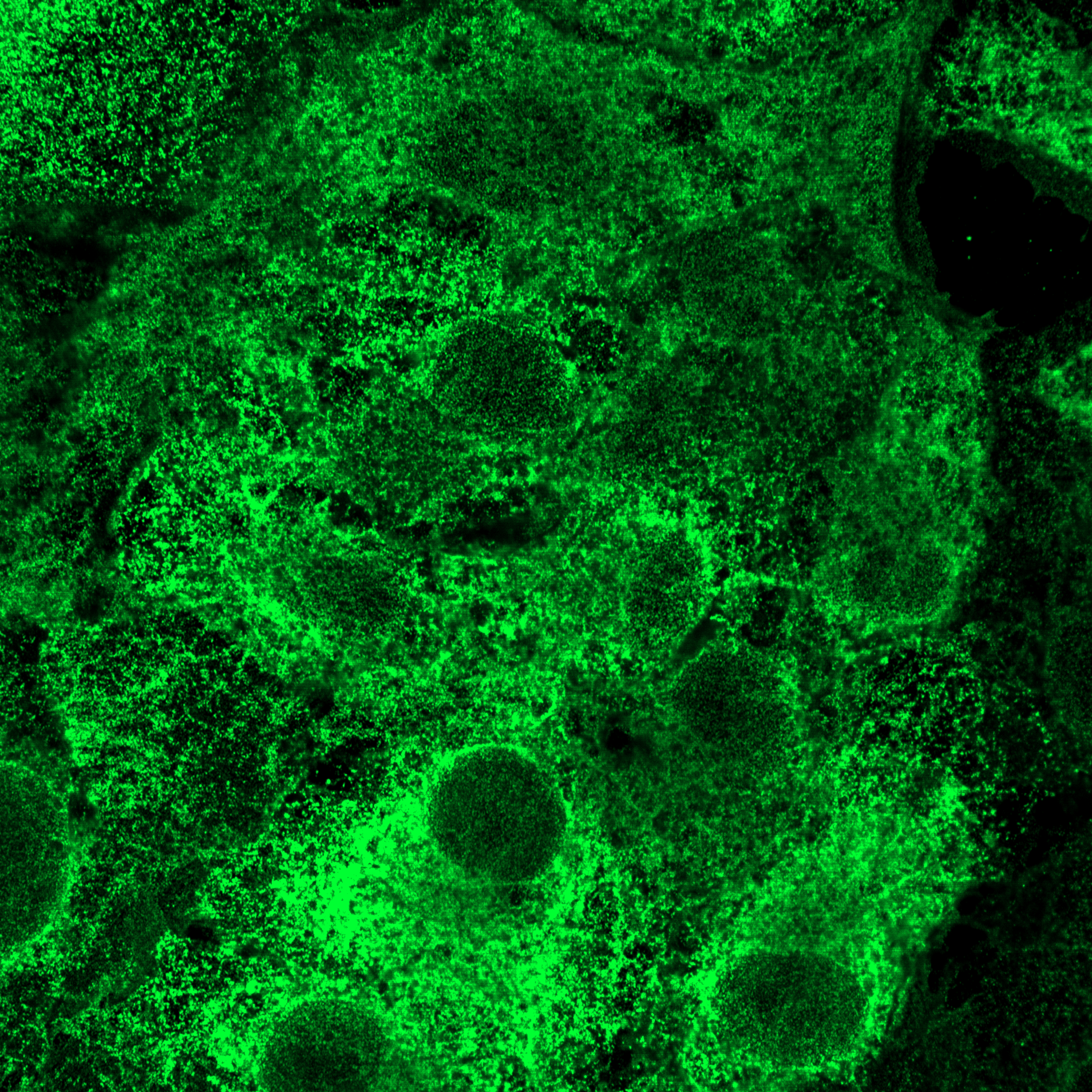

Supplement: Supplementary file 8 — Source data Fig. 3 [file 44319_2026_736_MOESM8_ESM.zip › Figure 3/3C /3C_CTNS KO_Replicate/NHE3-GFP HK-2 CTNS KO_EEA1_NHE3-GFP.tif]

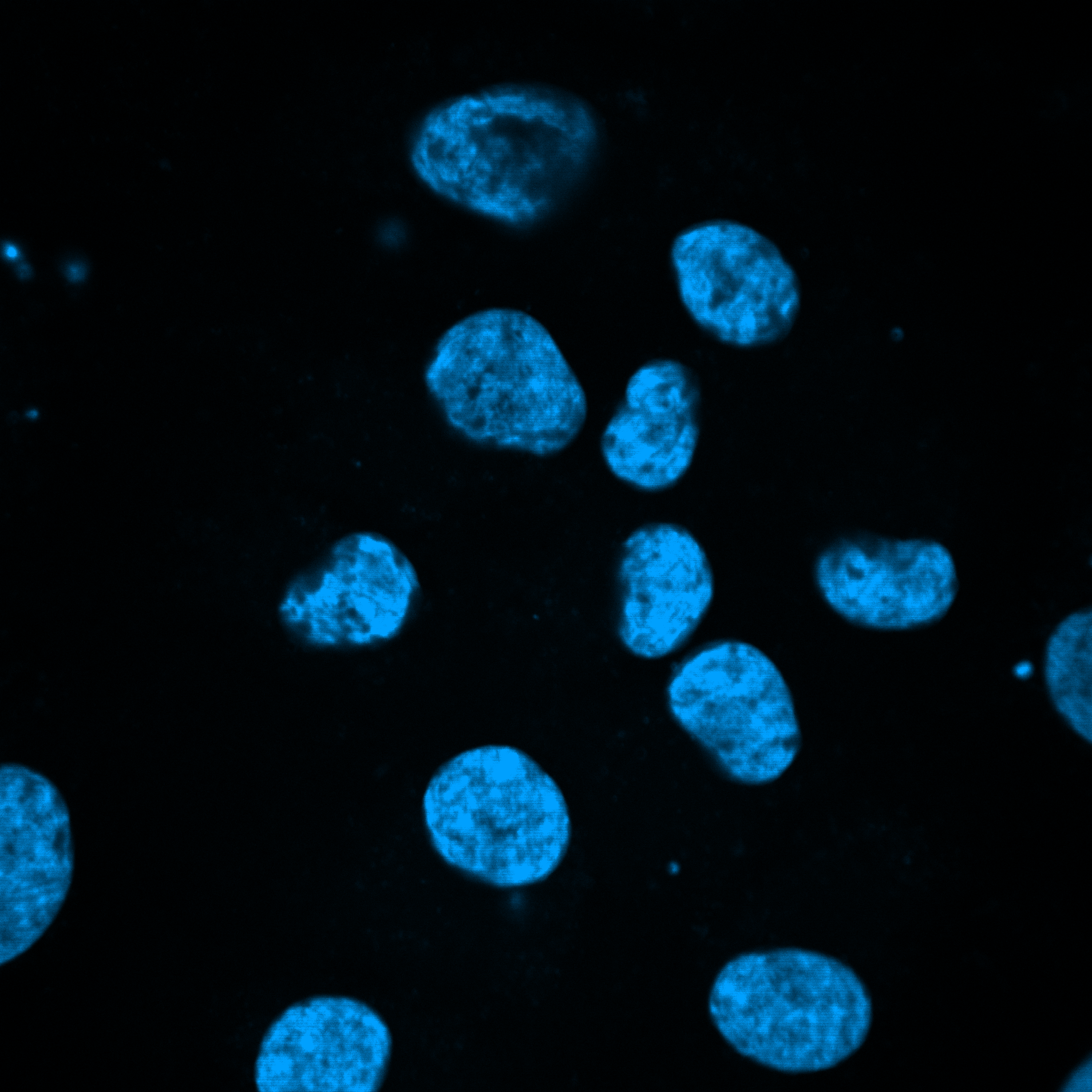

Supplement: Supplementary file 8 — Source data Fig. 3 [file 44319_2026_736_MOESM8_ESM.zip › Figure 3/3C /3C_CTNS KO_Replicate/NHE3-GFP HK-2 CTNS KO_EEA1_DAPI.tif]

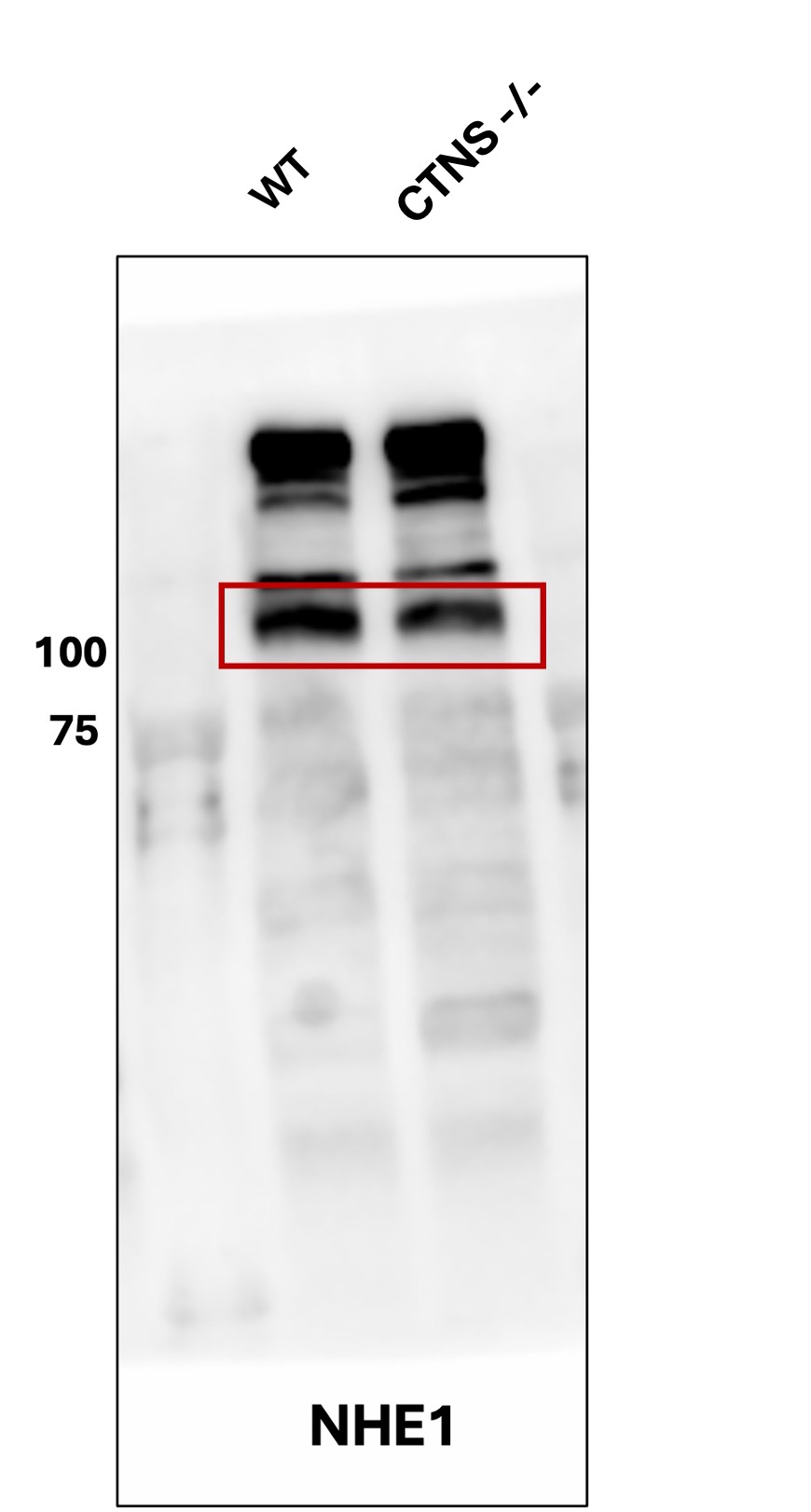

Supplement: Supplementary file 8 — Source data Fig. 3 [file 44319_2026_736_MOESM8_ESM.zip › Figure 3/3B/3B NHE1/Western/NHE1/Fig 3B NHE1.jpg]

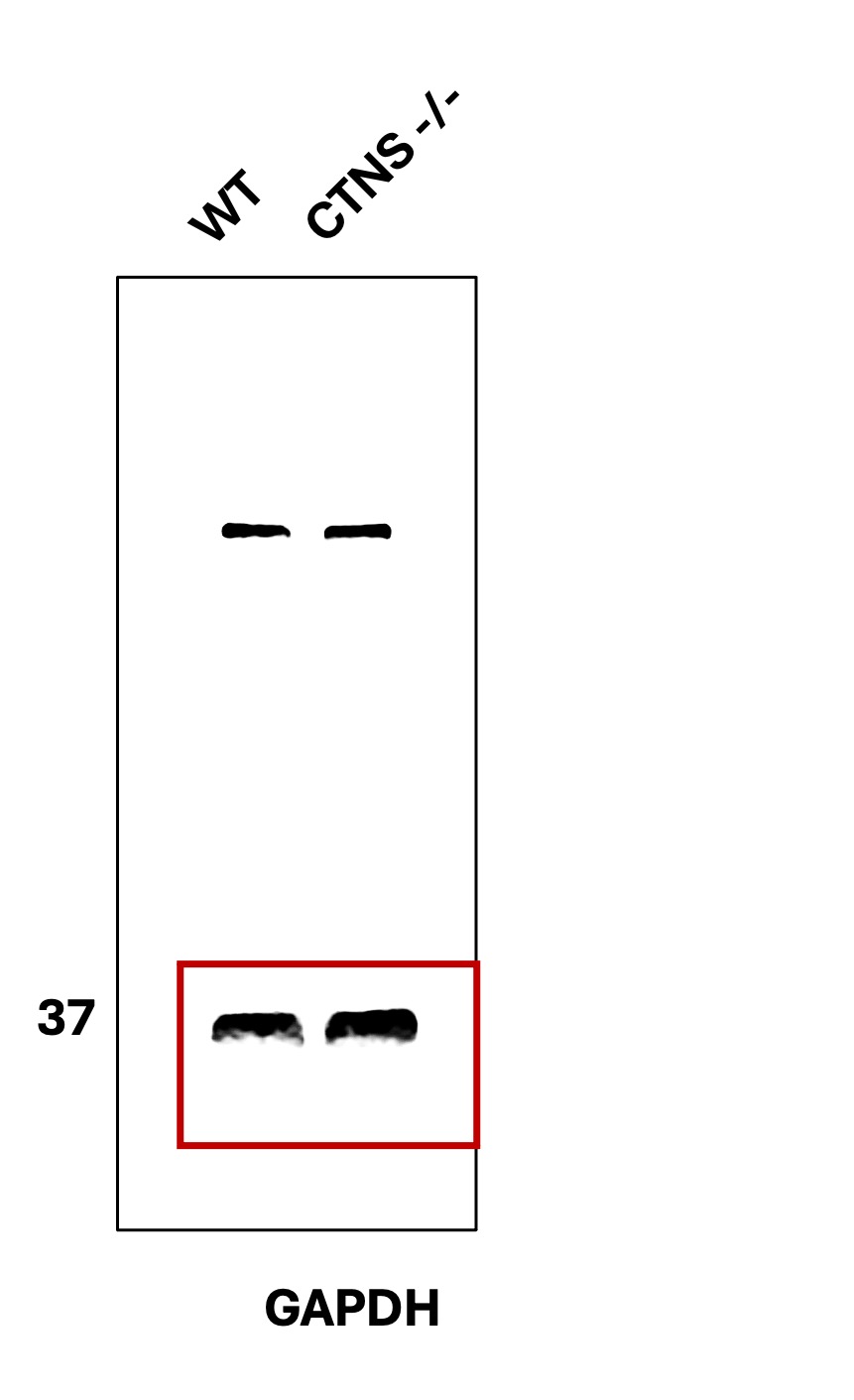

Supplement: Supplementary file 8 — Source data Fig. 3 [file 44319_2026_736_MOESM8_ESM.zip › Figure 3/3B/3B NHE1/Western/NHE1/Fig 3B GAPDH for NHE1.jpg]

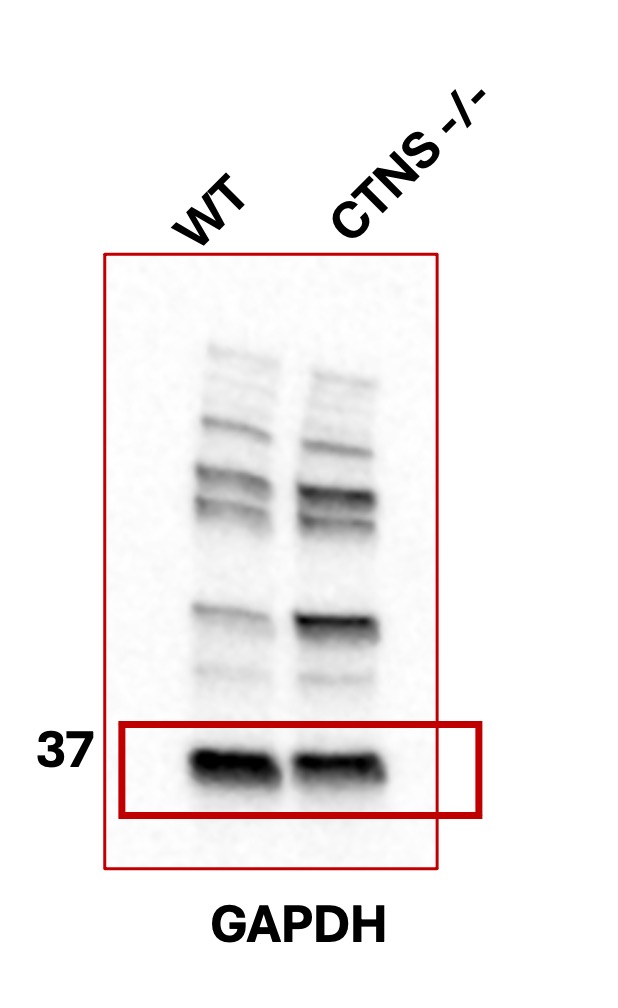

Supplement: Supplementary file 8 — Source data Fig. 3 [file 44319_2026_736_MOESM8_ESM.zip › Figure 3/3B/3B NHE2/Western/NHE2/GAPDH FOR NHE2.jpg]

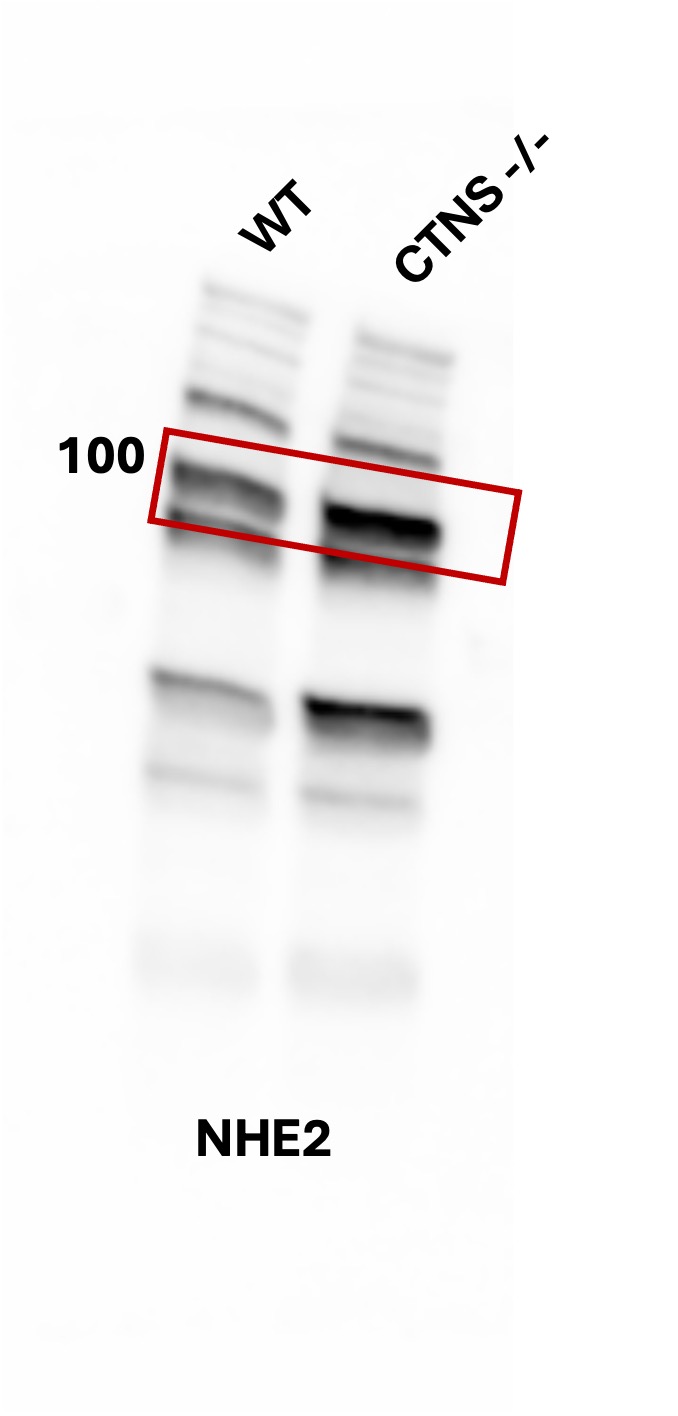

Supplement: Supplementary file 8 — Source data Fig. 3 [file 44319_2026_736_MOESM8_ESM.zip › Figure 3/3B/3B NHE2/Western/NHE2/NHE2.jpg]

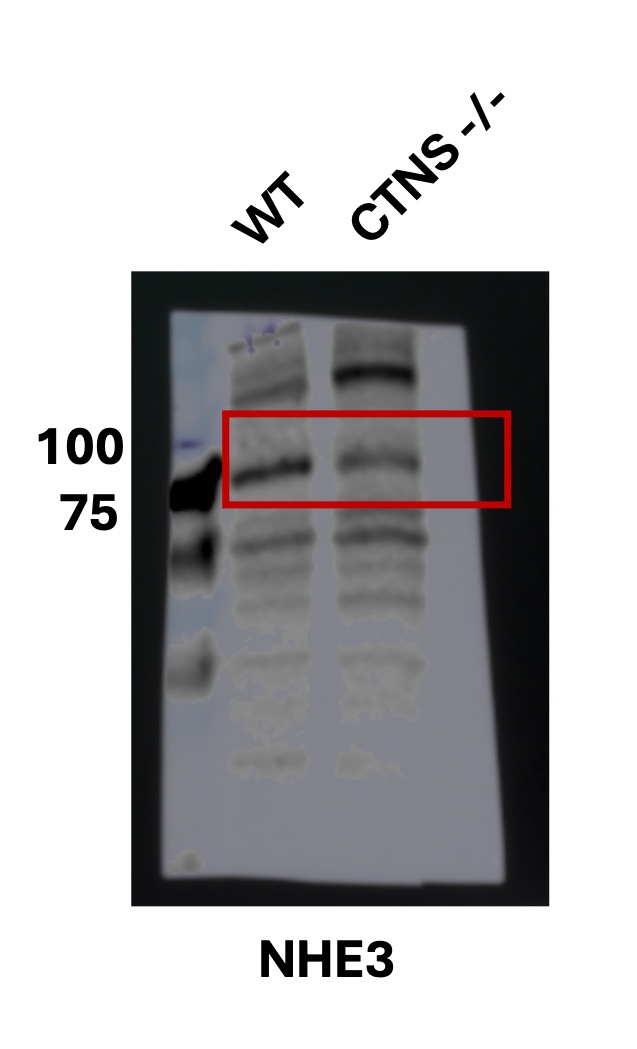

Supplement: Supplementary file 8 — Source data Fig. 3 [file 44319_2026_736_MOESM8_ESM.zip › Figure 3/3B/3B NHE3/Western/NHE3/Fig 3B NHE3.jpg]

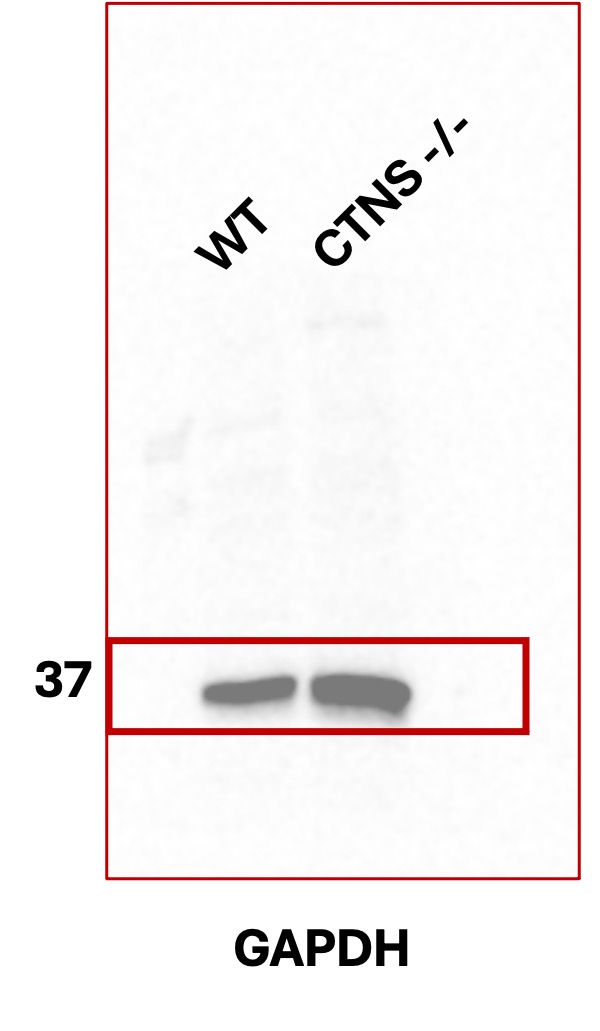

Supplement: Supplementary file 8 — Source data Fig. 3 [file 44319_2026_736_MOESM8_ESM.zip › Figure 3/3B/3B NHE3/Western/NHE3/Fig 3B_GAPDH for NHE3.jpg]

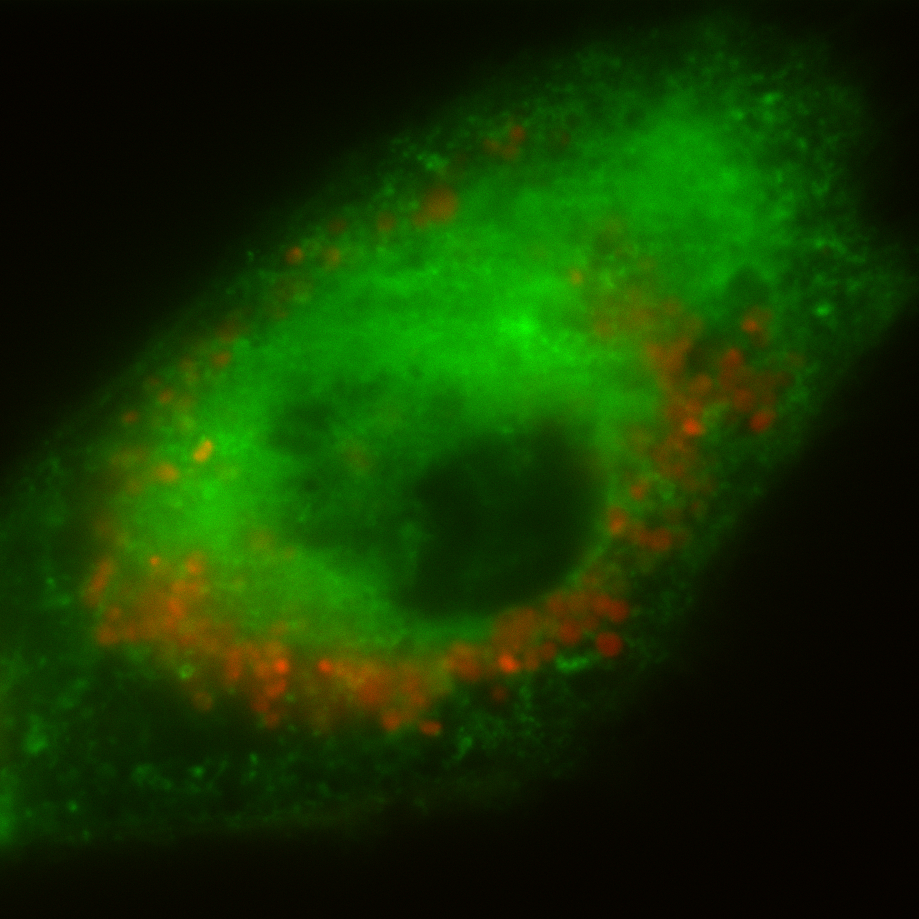

Supplement: Supplementary file 9 — Source data Fig. 4 [file 44319_2026_736_MOESM9_ESM.zip › Figure 4/4A/Fig 4A_CT-NHE3+LKG.tif]

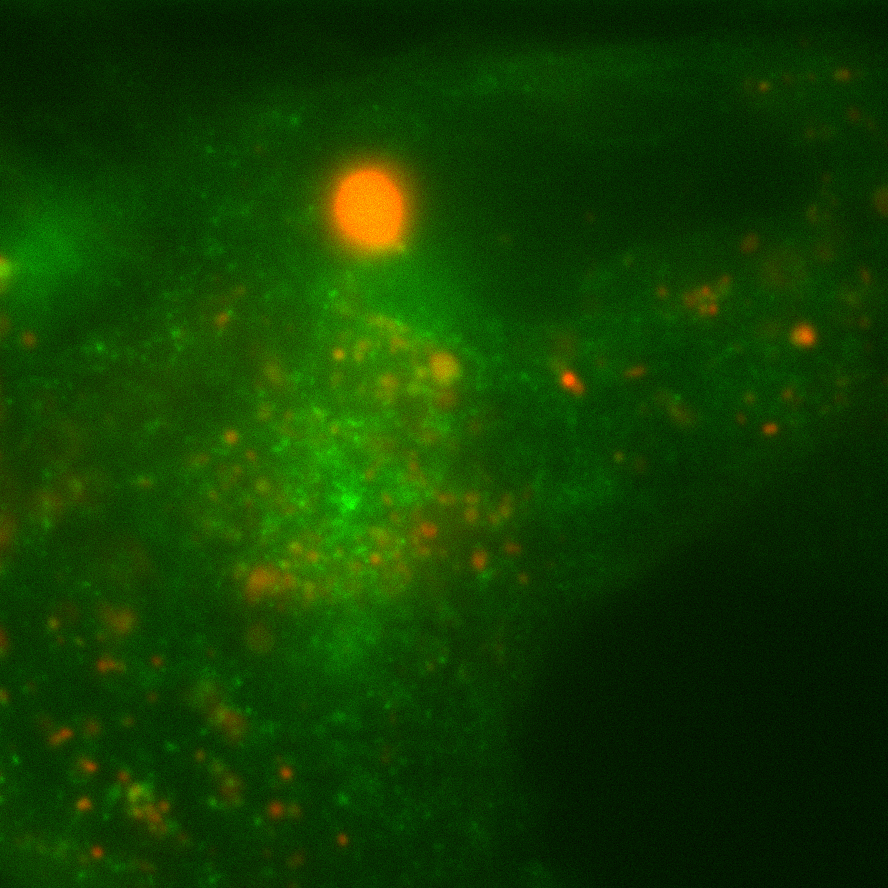

Supplement: Supplementary file 9 — Source data Fig. 4 [file 44319_2026_736_MOESM9_ESM.zip › Figure 4/4A/Fig 4A_CT-NHE3+CTNS.tif]

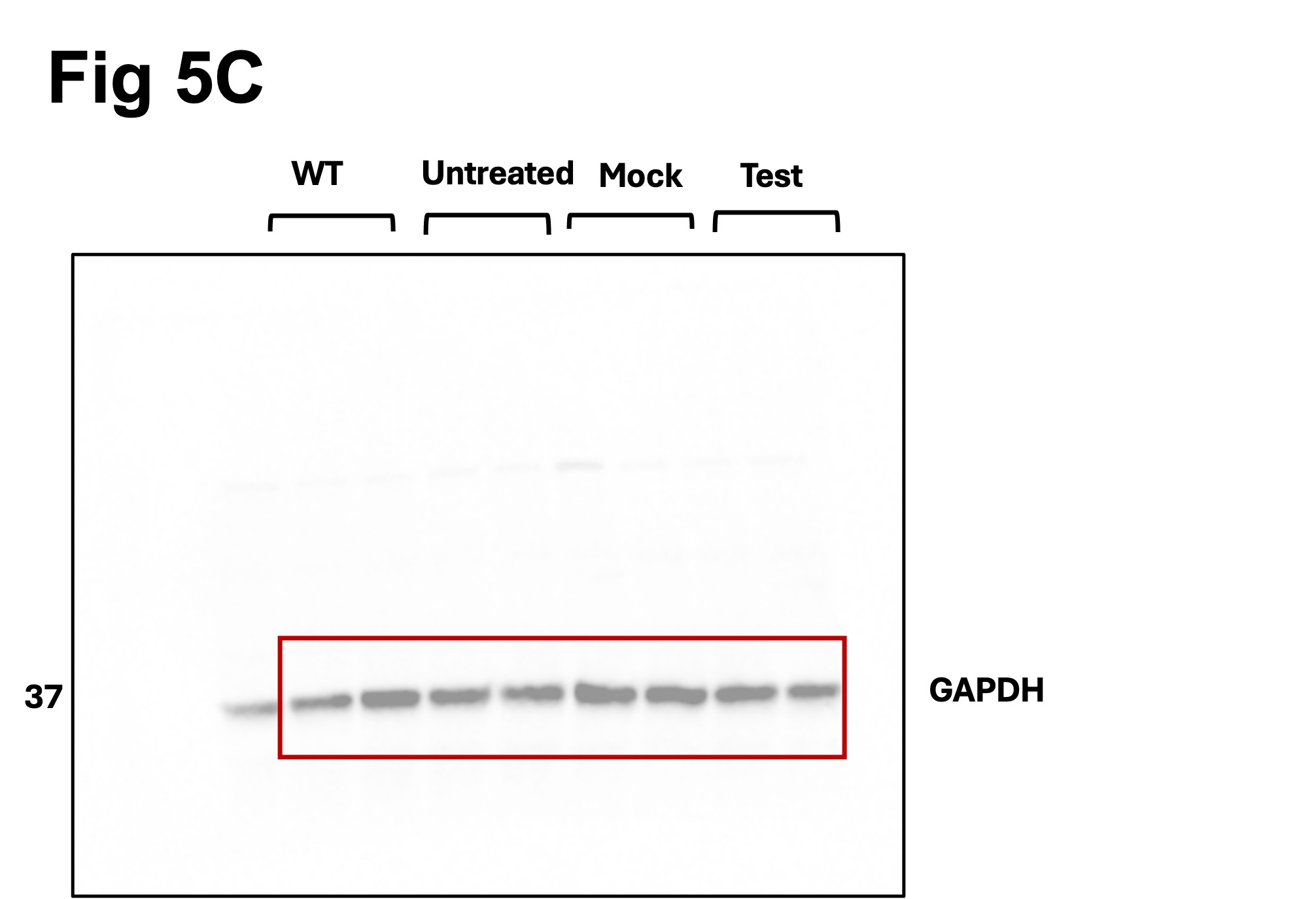

Supplement: Supplementary file 10 — Source data Fig. 5 [file 44319_2026_736_MOESM10_ESM.zip › Figure 5/5C/C_Western/GAPDH FOR Nhe2.jpg]

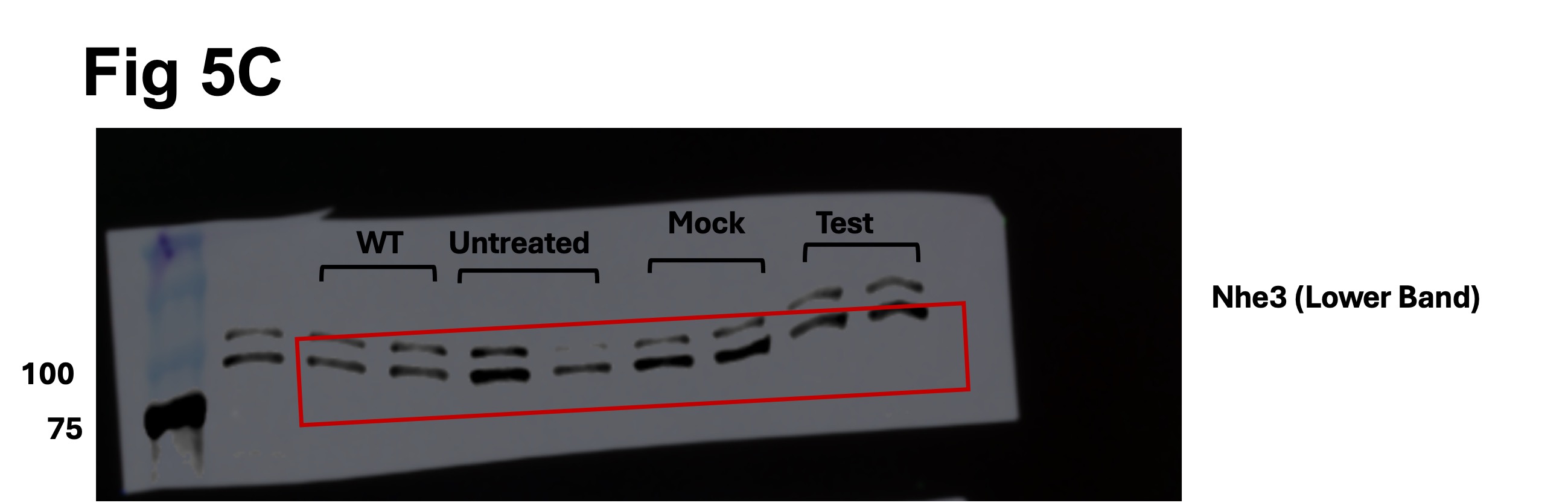

Supplement: Supplementary file 10 — Source data Fig. 5 [file 44319_2026_736_MOESM10_ESM.zip › Figure 5/5C/C_Western/Fig 5C Nhe3.jpg]

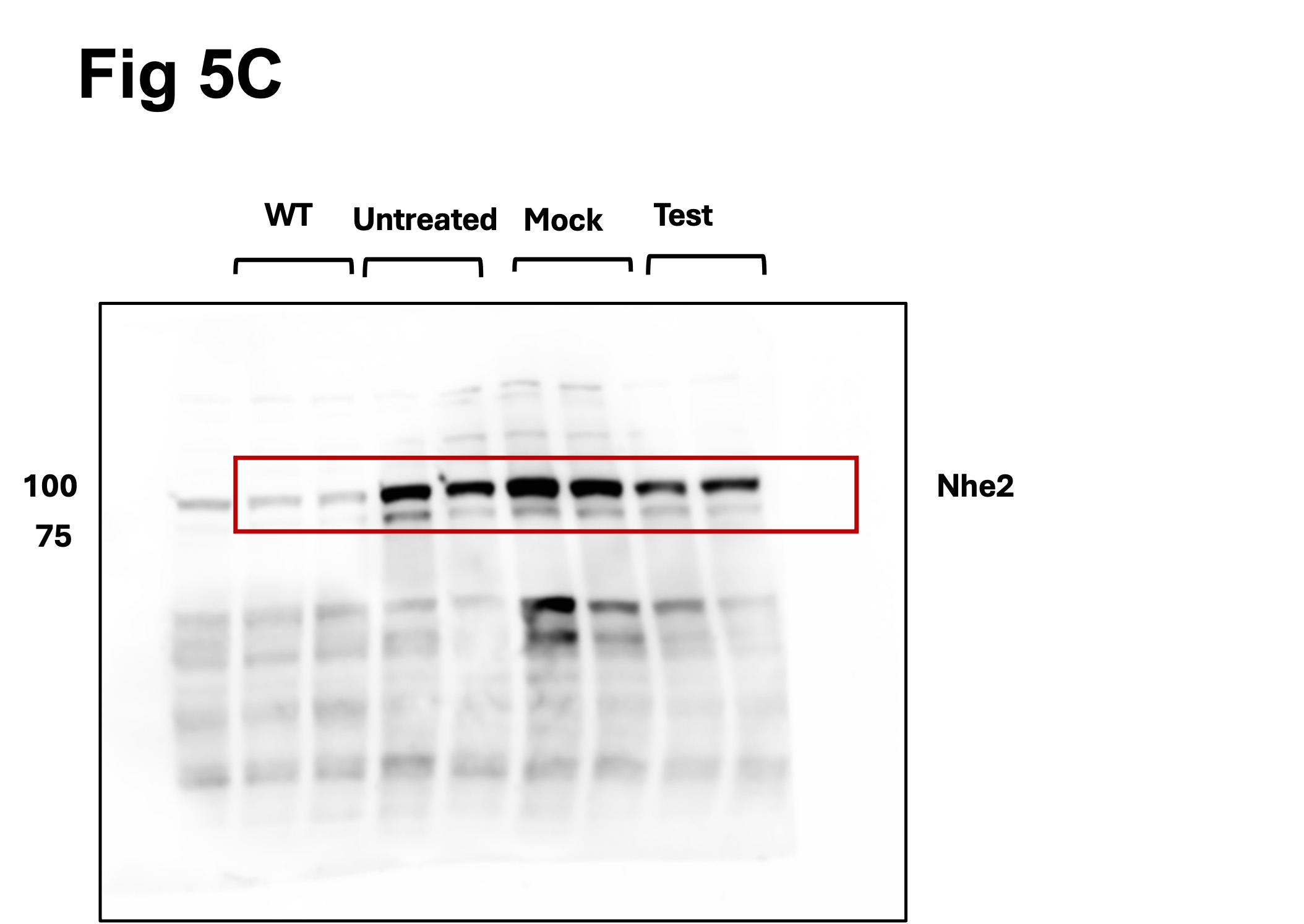

Supplement: Supplementary file 10 — Source data Fig. 5 [file 44319_2026_736_MOESM10_ESM.zip › Figure 5/5C/C_Western/Fig 5C_Nhe2.jpg]

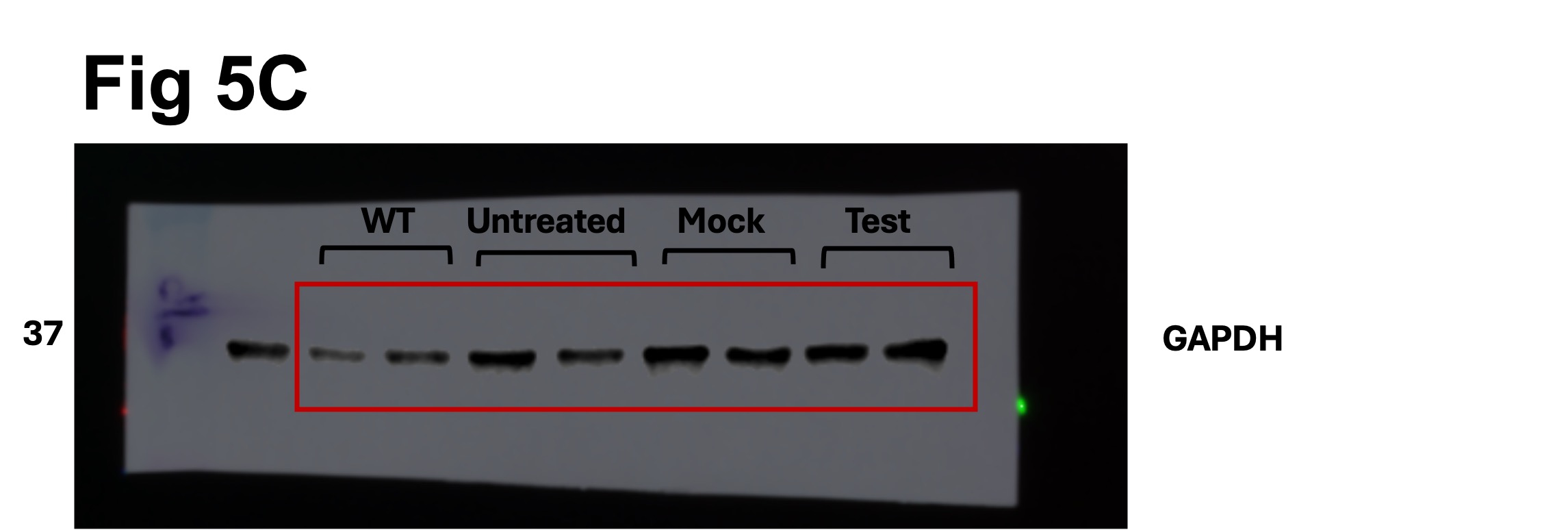

Supplement: Supplementary file 10 — Source data Fig. 5 [file 44319_2026_736_MOESM10_ESM.zip › Figure 5/5C/C_Western/Fig 5C_GAPDH for Nhe3.jpg]

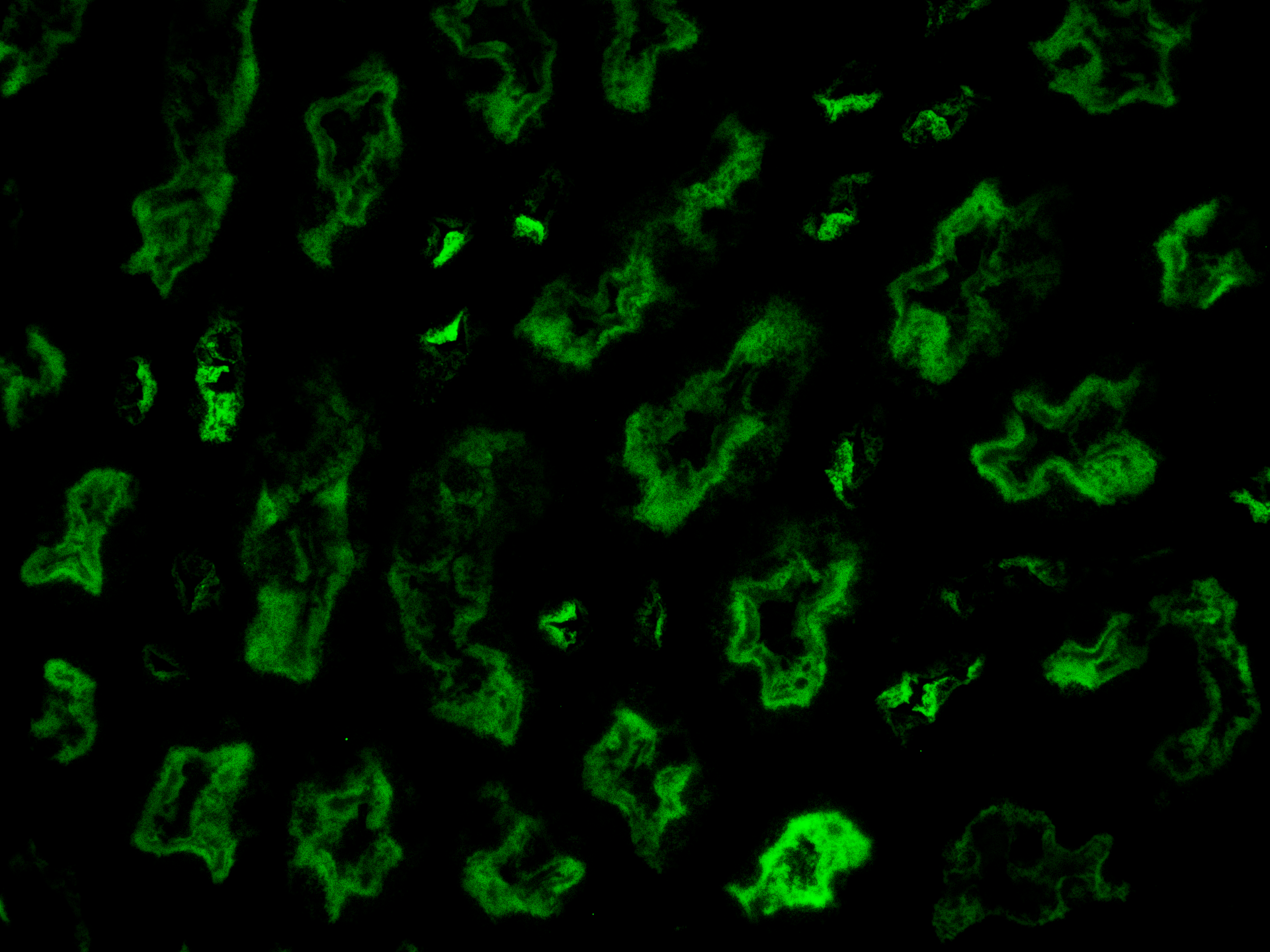

Supplement: Supplementary file 10 — Source data Fig. 5 [file 44319_2026_736_MOESM10_ESM.zip › Figure 5/5D/D_Images/WT NHE3.tif]

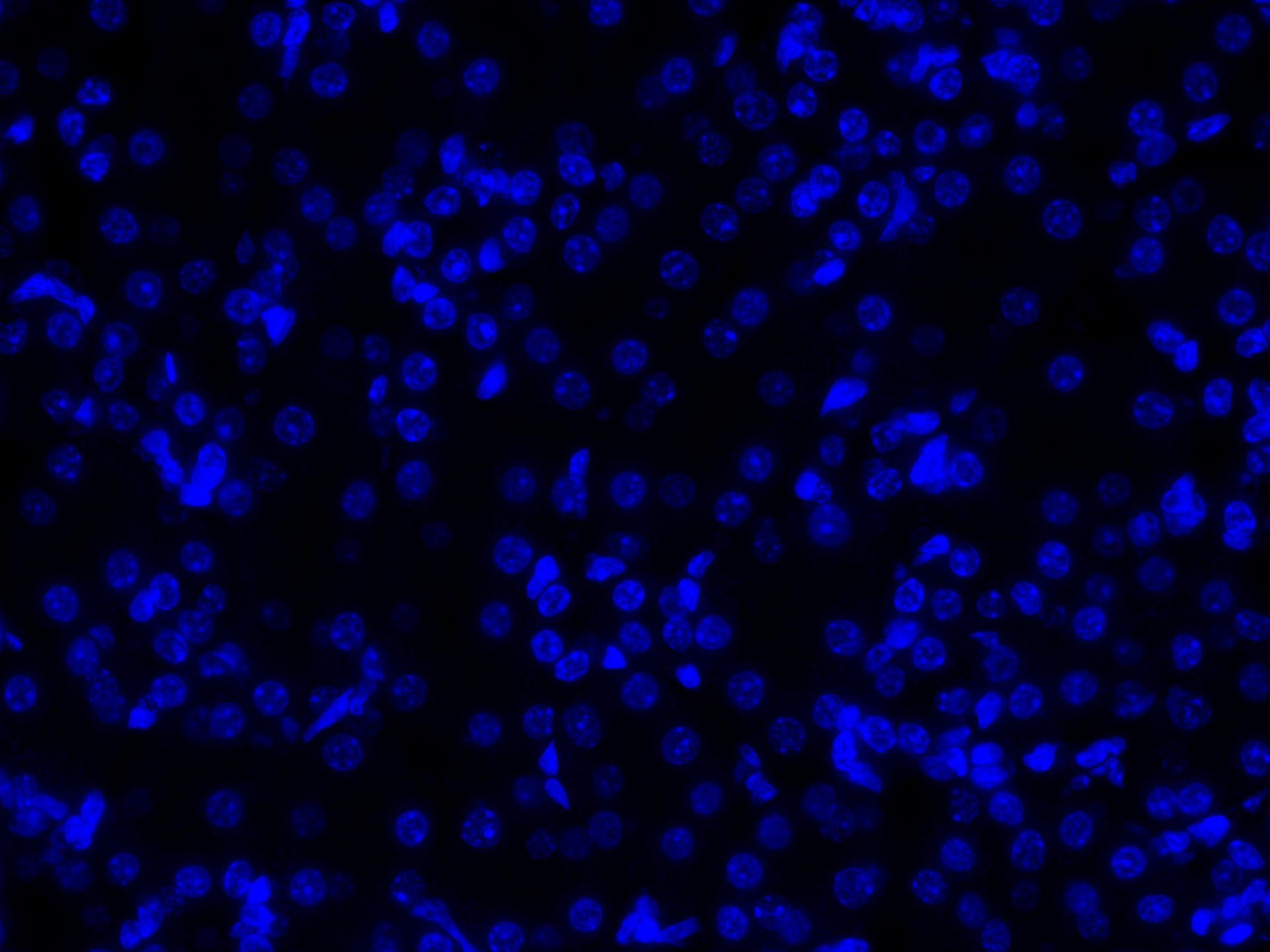

Supplement: Supplementary file 10 — Source data Fig. 5 [file 44319_2026_736_MOESM10_ESM.zip › Figure 5/5D/D_Images/WT DAPI.tif]

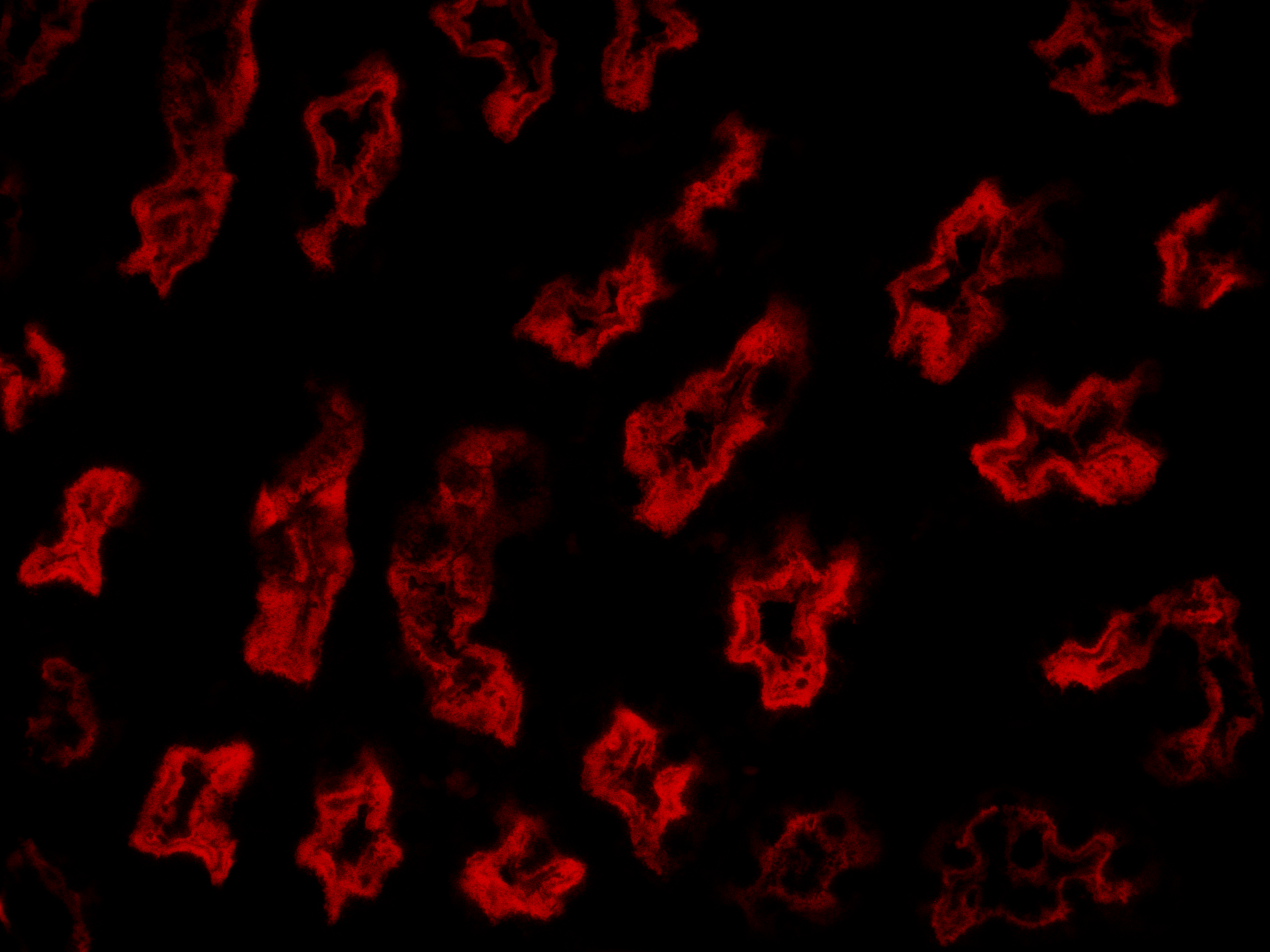

Supplement: Supplementary file 10 — Source data Fig. 5 [file 44319_2026_736_MOESM10_ESM.zip › Figure 5/5D/D_Images/WT LTL.tif]

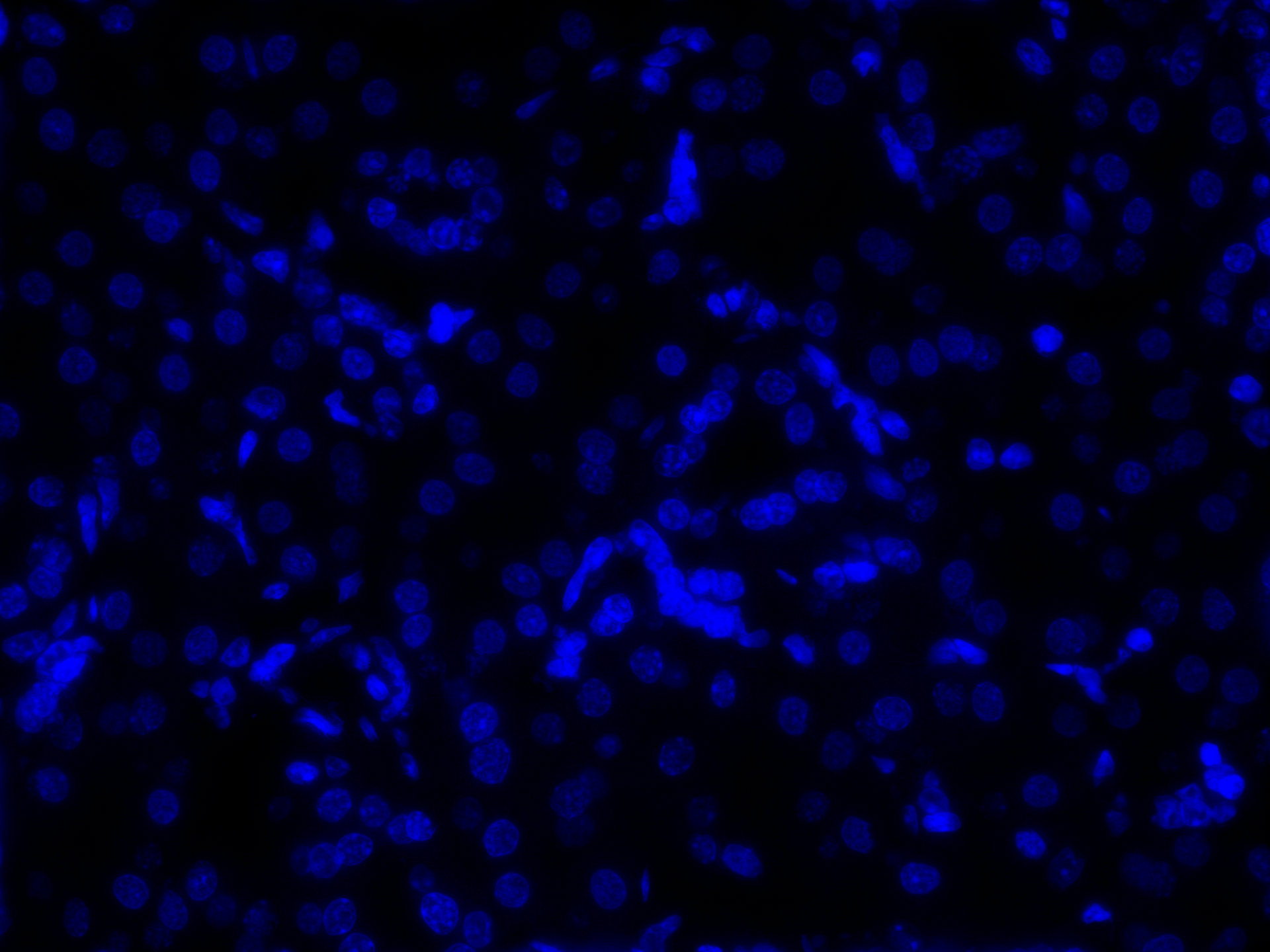

Supplement: Supplementary file 10 — Source data Fig. 5 [file 44319_2026_736_MOESM10_ESM.zip › Figure 5/5D/D_Images/Test DAPI.tif]

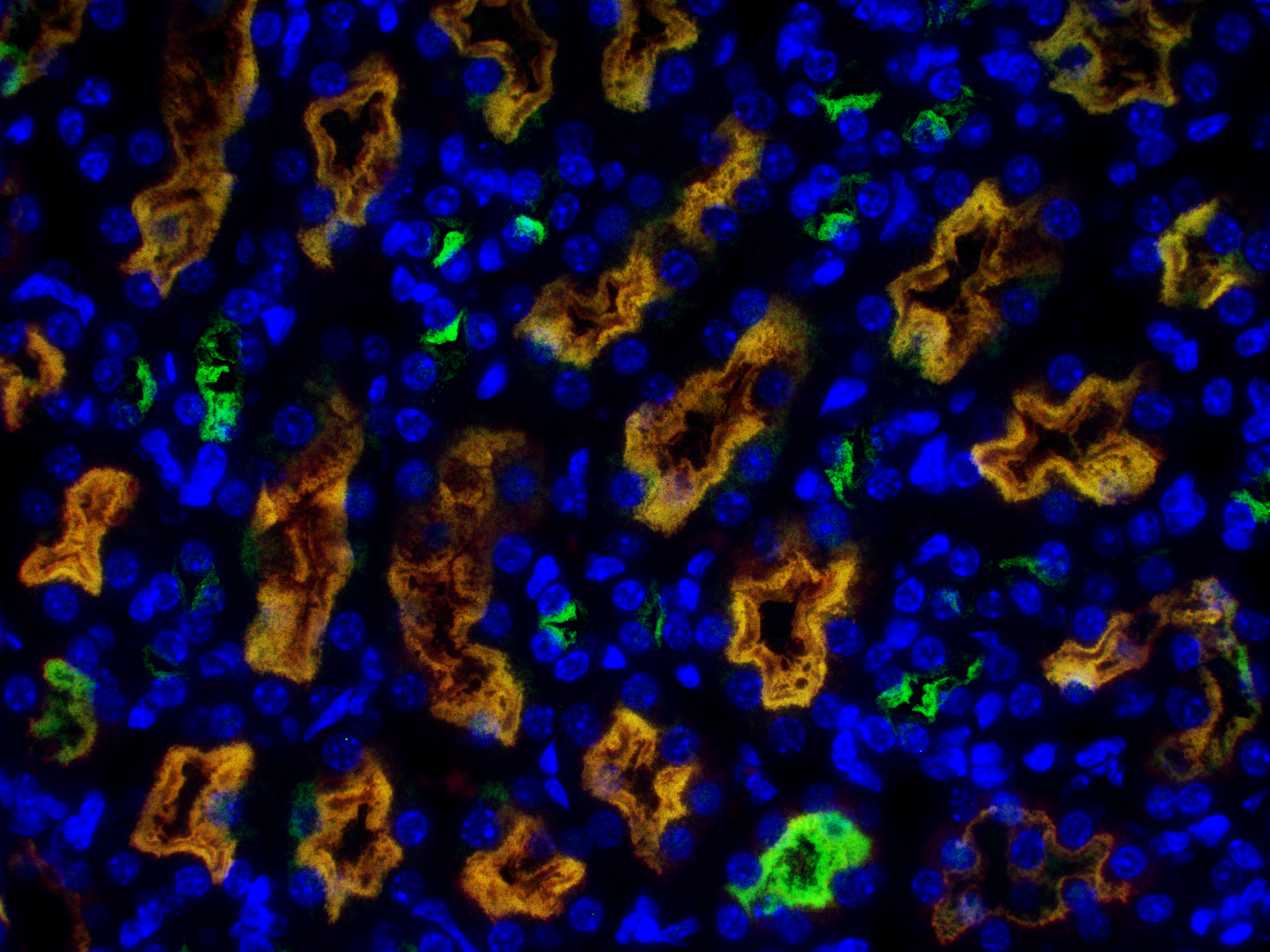

Supplement: Supplementary file 10 — Source data Fig. 5 [file 44319_2026_736_MOESM10_ESM.zip › Figure 5/5D/D_Images/WT Merged.tif]

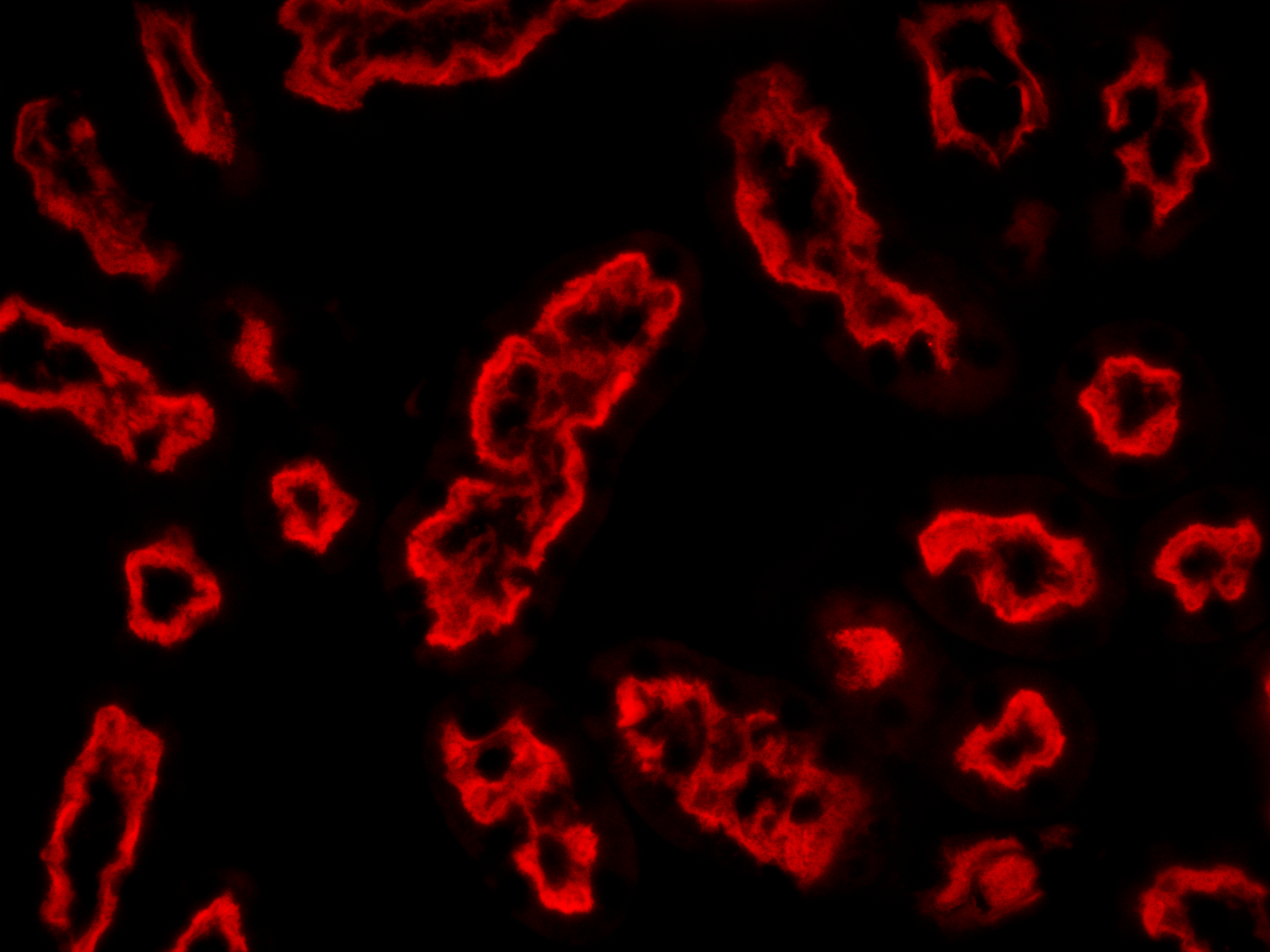

Supplement: Supplementary file 10 — Source data Fig. 5 [file 44319_2026_736_MOESM10_ESM.zip › Figure 5/5D/D_Images/Test LTL.tif]

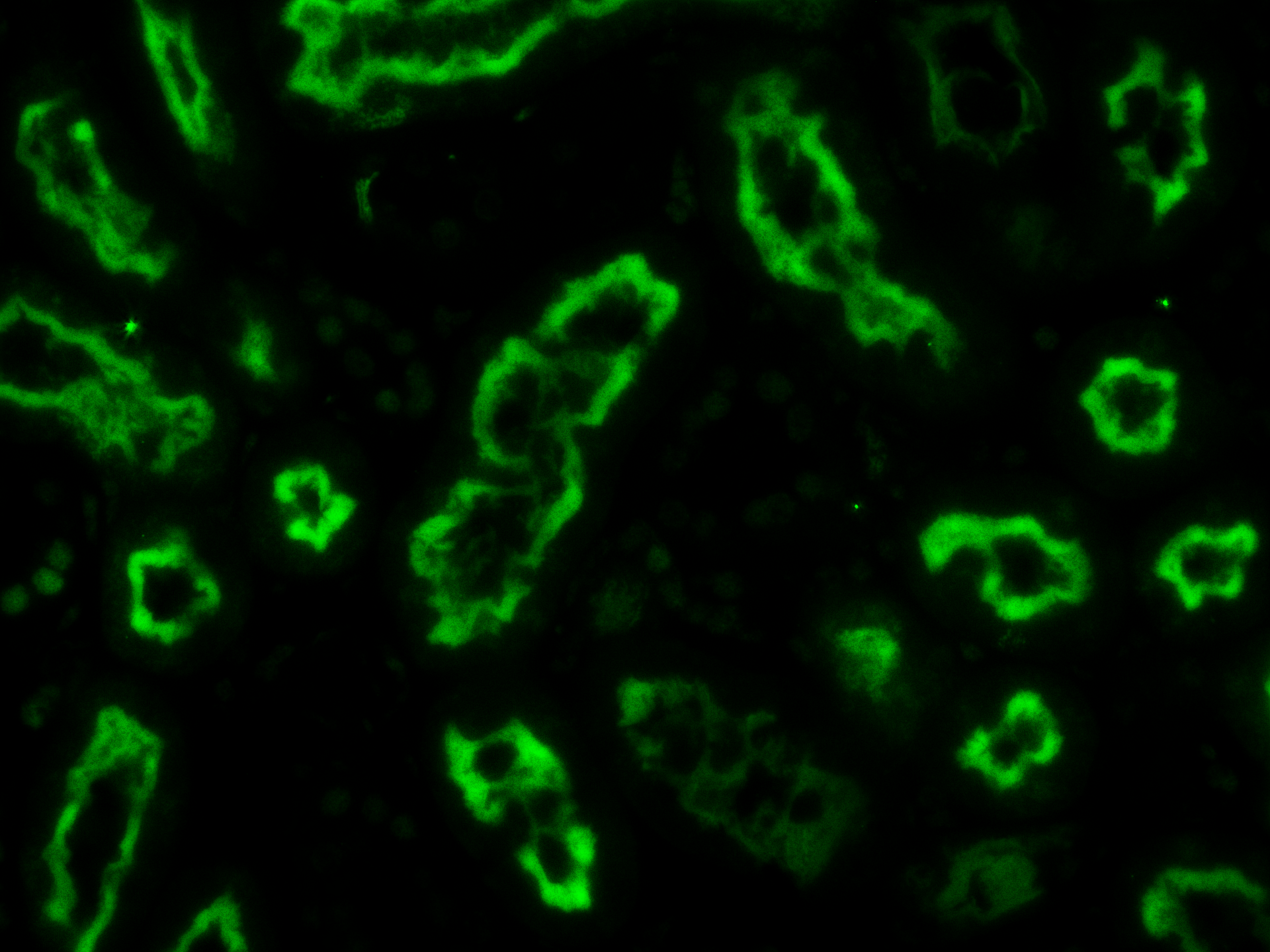

Supplement: Supplementary file 10 — Source data Fig. 5 [file 44319_2026_736_MOESM10_ESM.zip › Figure 5/5D/D_Images/Test NHE3.tif]

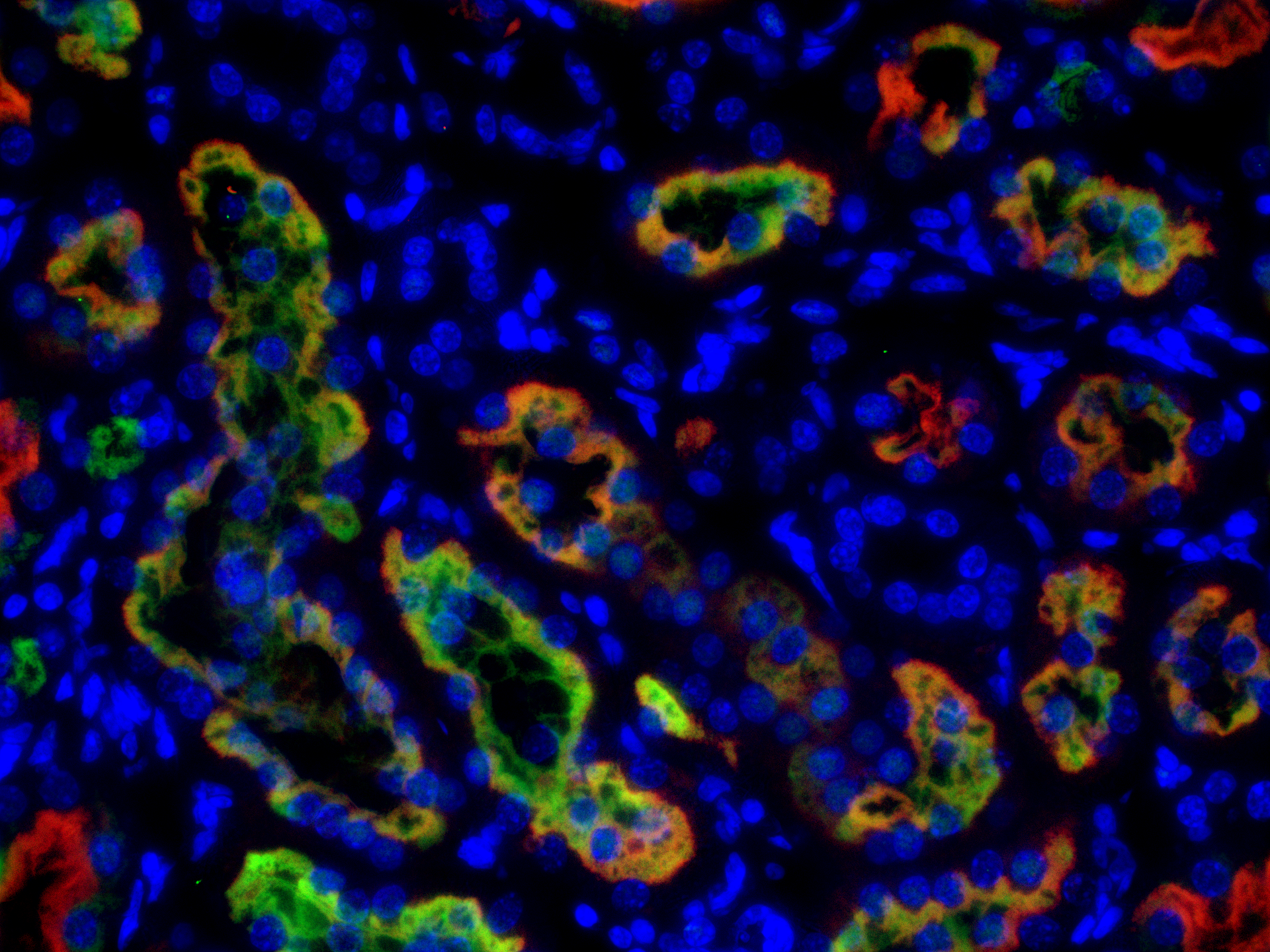

Supplement: Supplementary file 10 — Source data Fig. 5 [file 44319_2026_736_MOESM10_ESM.zip › Figure 5/5D/D_Images/Untreated Merged.tif]

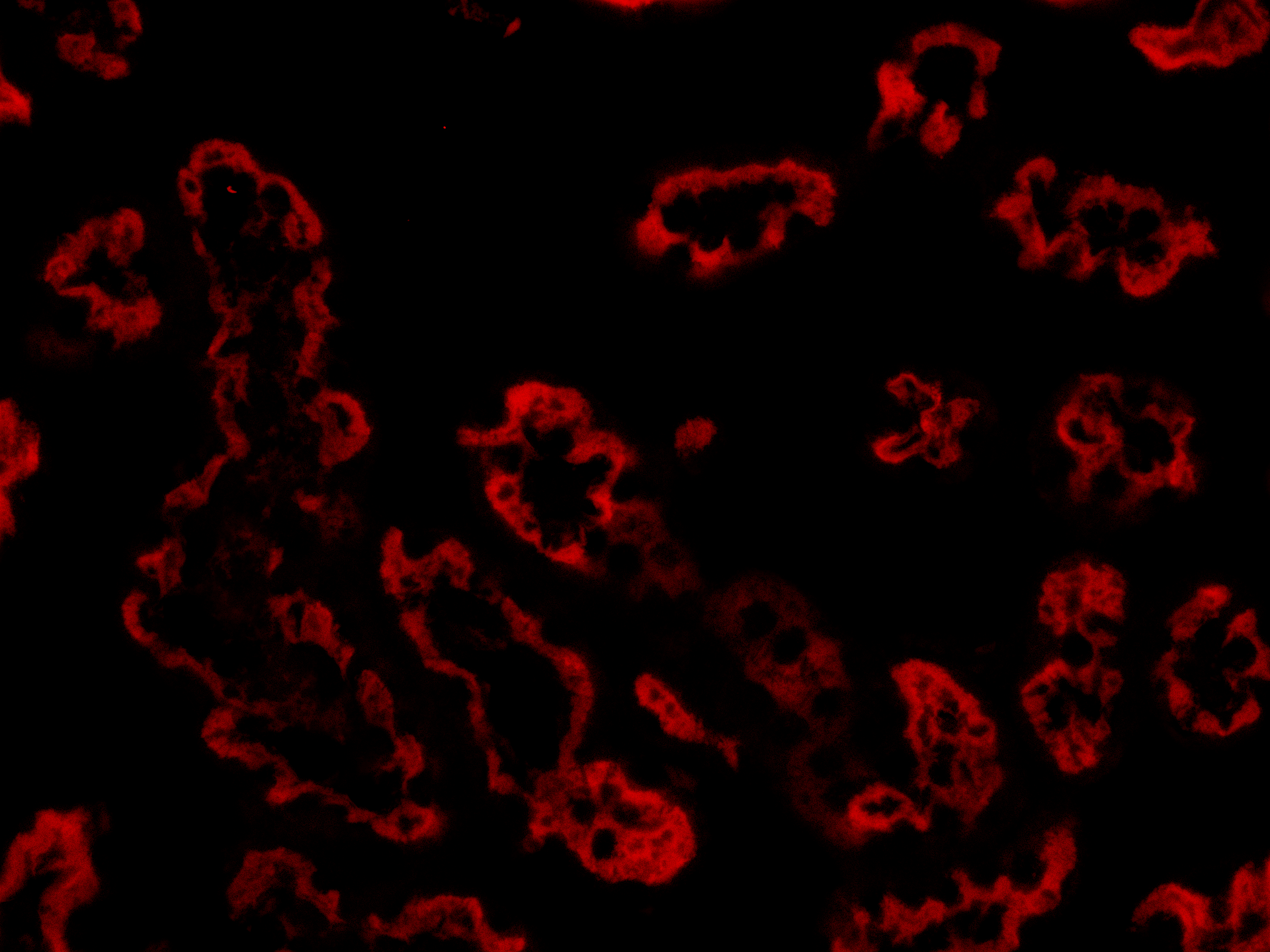

Supplement: Supplementary file 10 — Source data Fig. 5 [file 44319_2026_736_MOESM10_ESM.zip › Figure 5/5D/D_Images/Untreated LTL.tif]

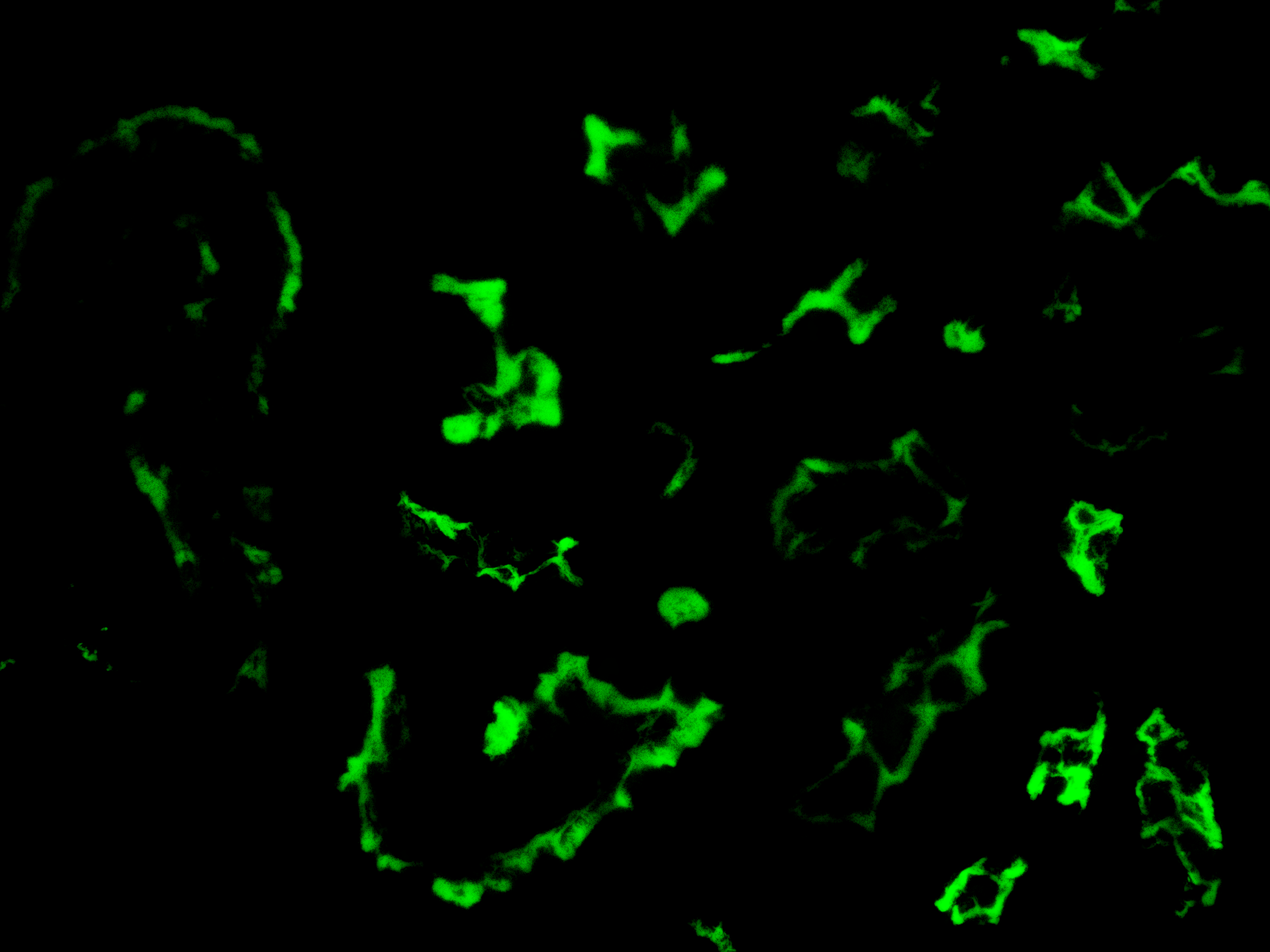

Supplement: Supplementary file 10 — Source data Fig. 5 [file 44319_2026_736_MOESM10_ESM.zip › Figure 5/5D/D_Images/MOCK NHE3.tif]

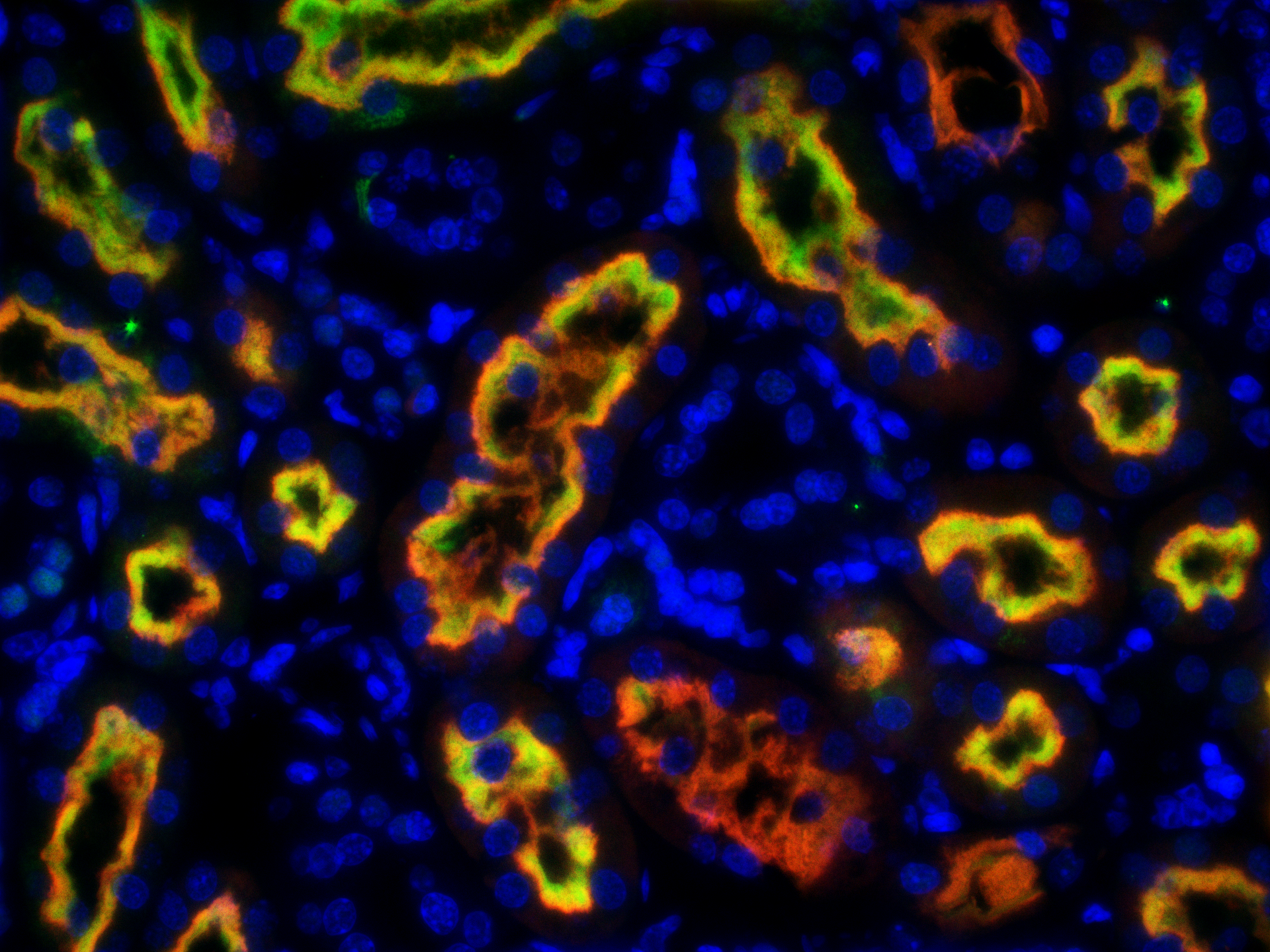

Supplement: Supplementary file 10 — Source data Fig. 5 [file 44319_2026_736_MOESM10_ESM.zip › Figure 5/5D/D_Images/Test Merged.tif]

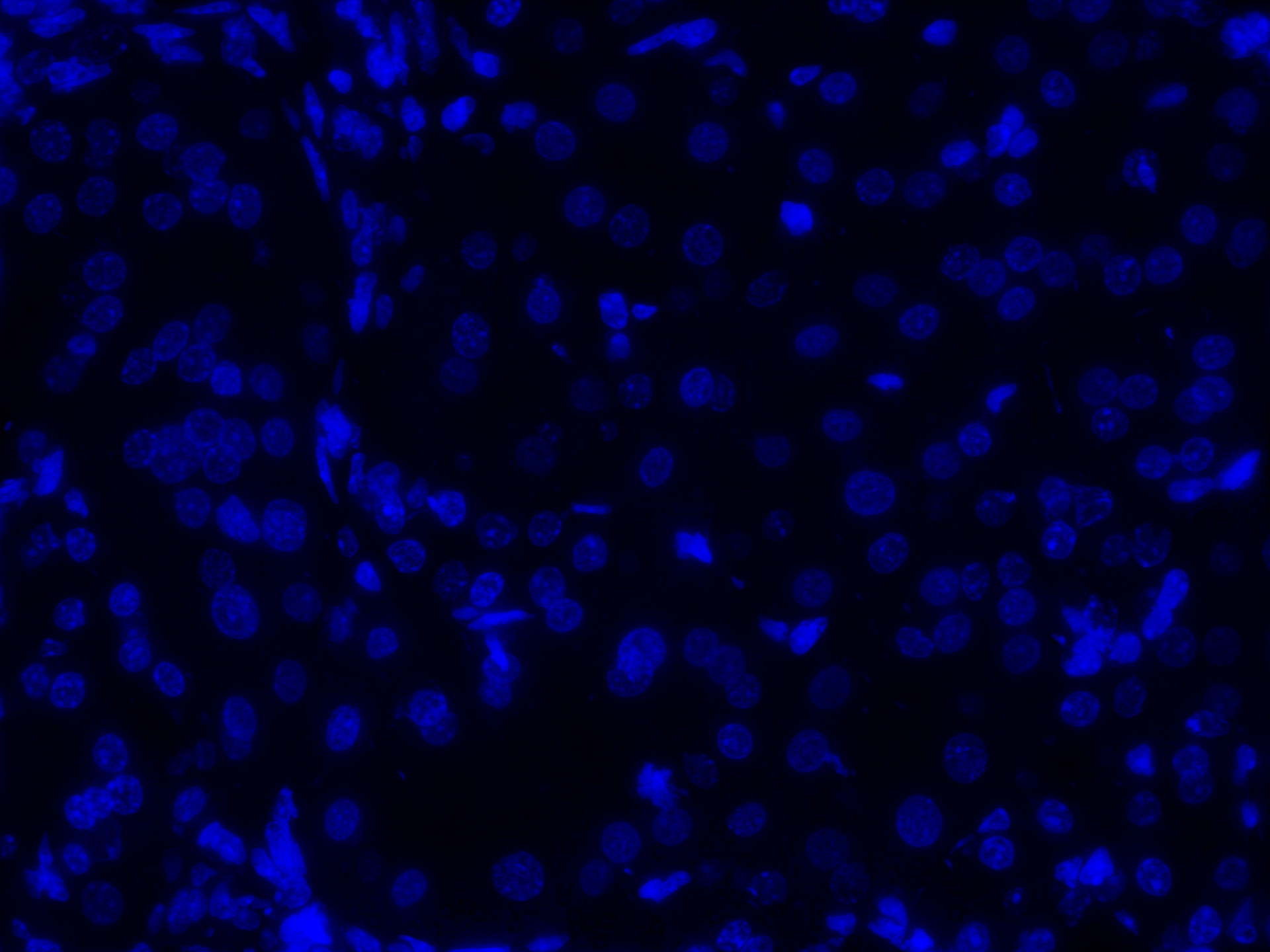

Supplement: Supplementary file 10 — Source data Fig. 5 [file 44319_2026_736_MOESM10_ESM.zip › Figure 5/5D/D_Images/Mock DAPI.tif]

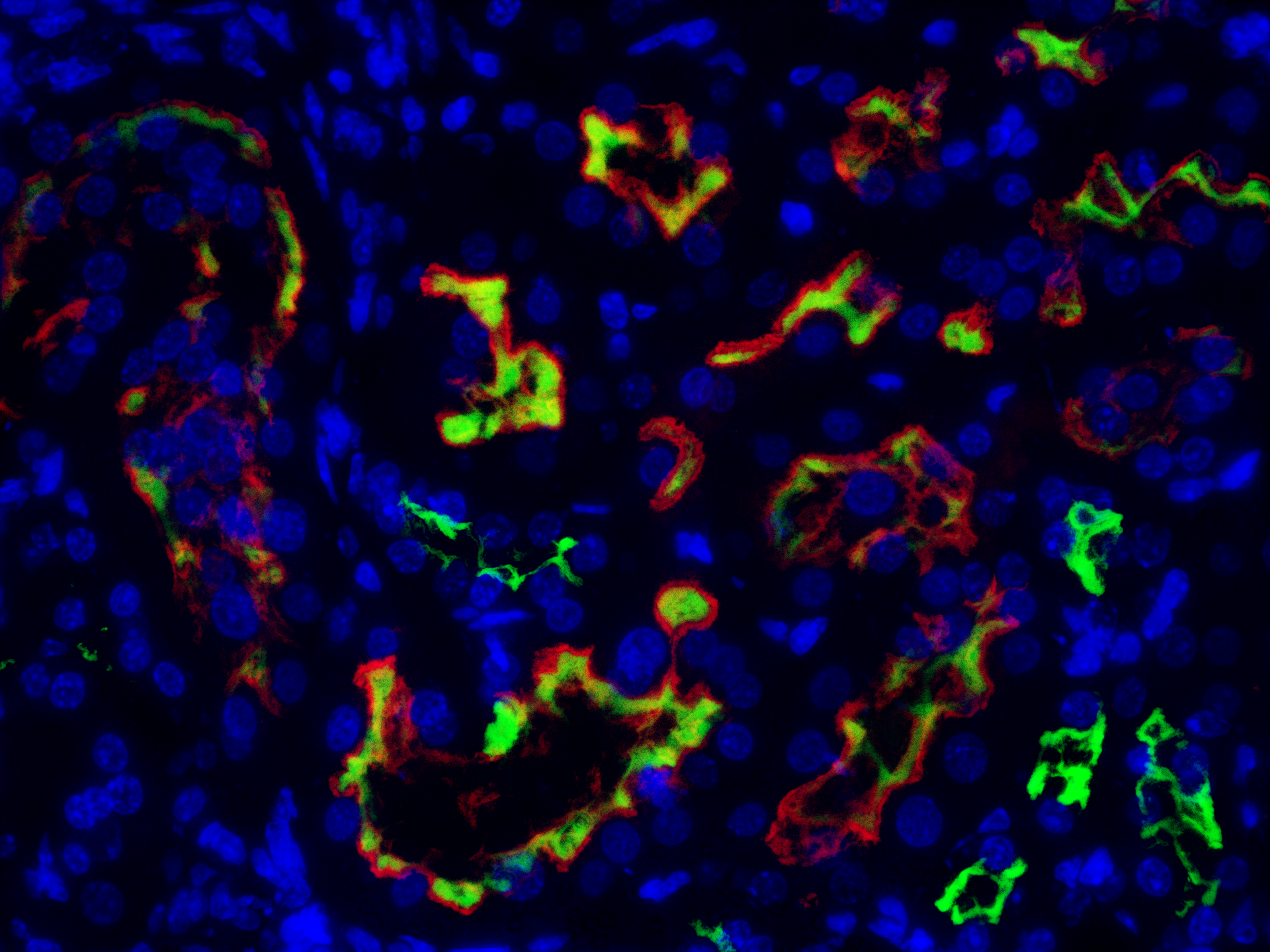

Supplement: Supplementary file 10 — Source data Fig. 5 [file 44319_2026_736_MOESM10_ESM.zip › Figure 5/5D/D_Images/Mock Merged.tif]

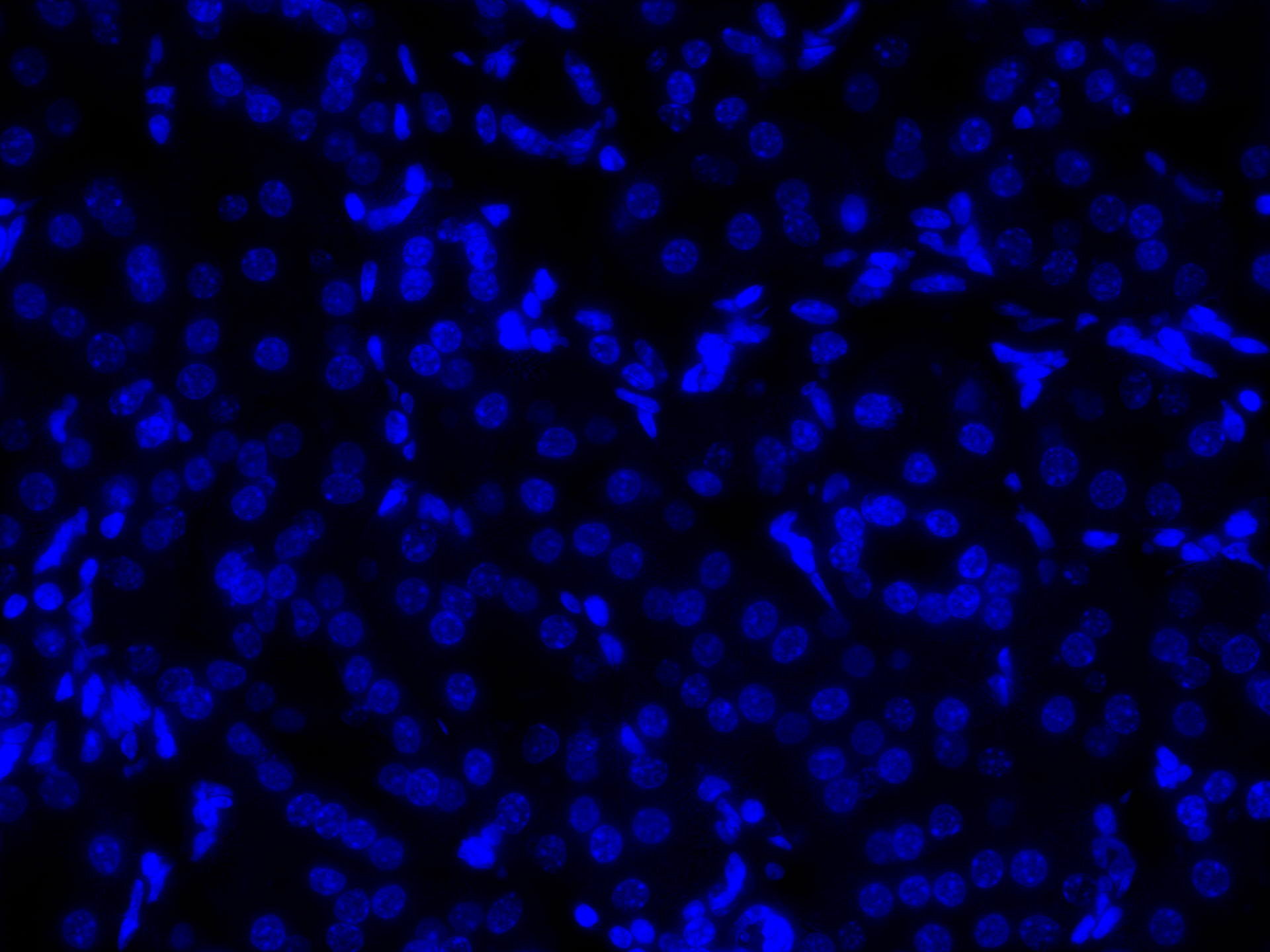

Supplement: Supplementary file 10 — Source data Fig. 5 [file 44319_2026_736_MOESM10_ESM.zip › Figure 5/5D/D_Images/Untreated DAPI.tif]

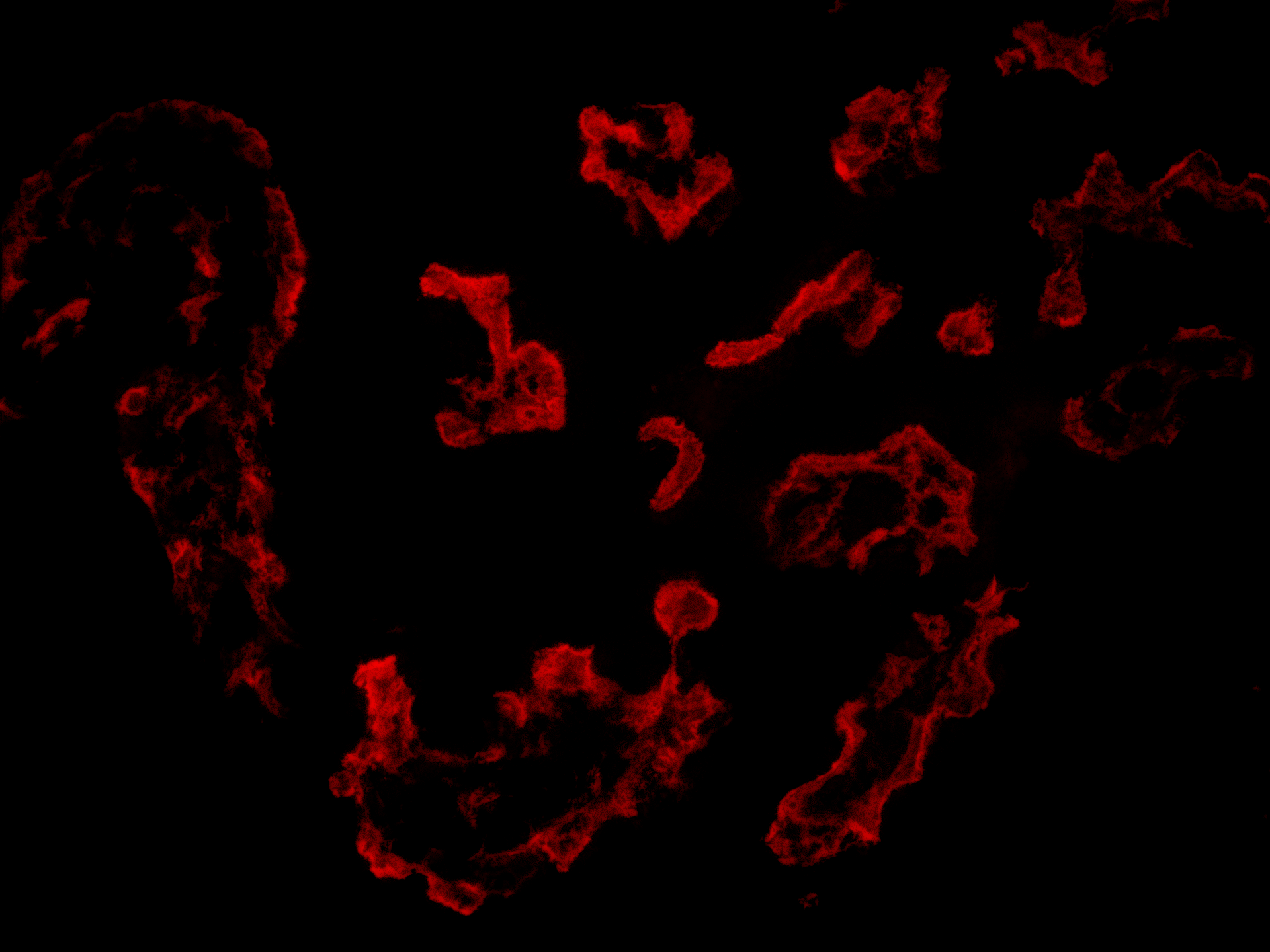

Supplement: Supplementary file 10 — Source data Fig. 5 [file 44319_2026_736_MOESM10_ESM.zip › Figure 5/5D/D_Images/MOCK LTL.tif]

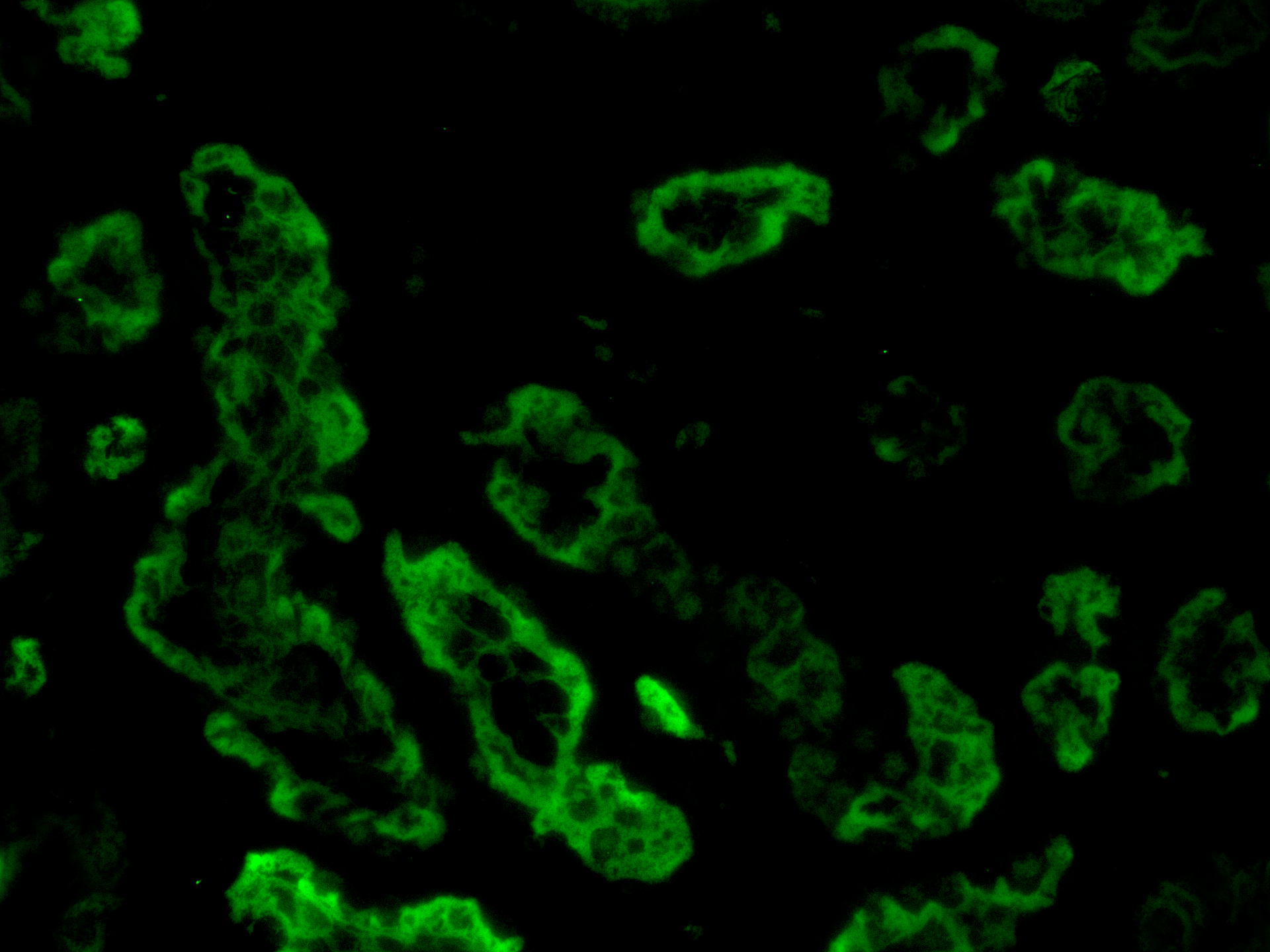

Supplement: Supplementary file 10 — Source data Fig. 5 [file 44319_2026_736_MOESM10_ESM.zip › Figure 5/5D/D_Images/Untreated NHE3.tif]

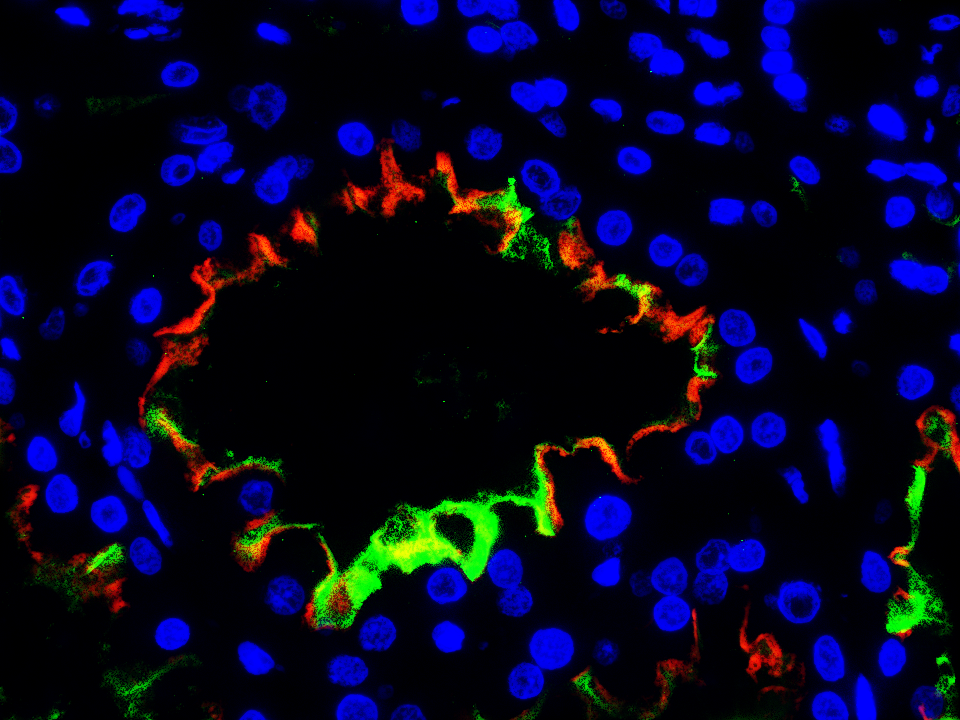

Supplement: Supplementary file 11 — Source data Fig. 6 [file 44319_2026_736_MOESM11_ESM.zip › Figure 6/Images/Cystinosis Mereged.tif]

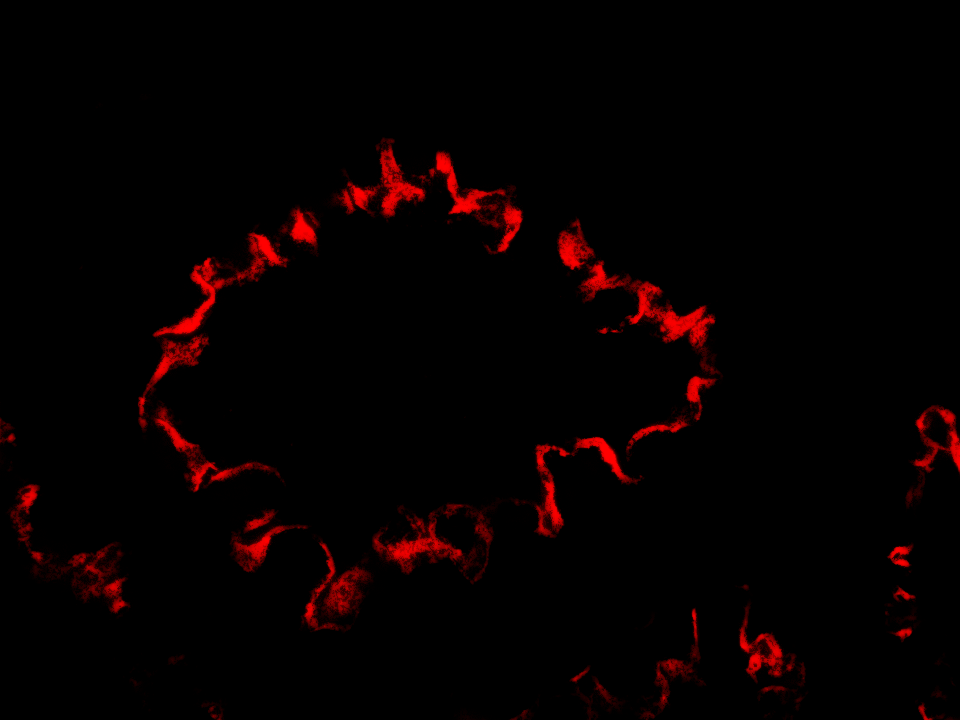

Supplement: Supplementary file 11 — Source data Fig. 6 [file 44319_2026_736_MOESM11_ESM.zip › Figure 6/Images/Cystinosis_LTL.tif]

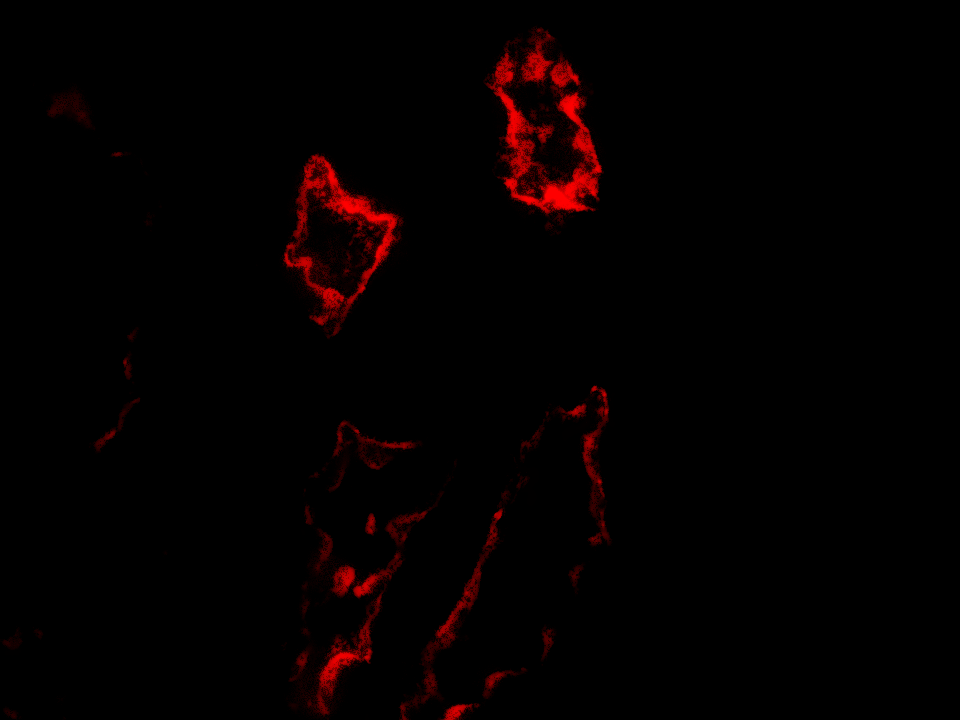

Supplement: Supplementary file 11 — Source data Fig. 6 [file 44319_2026_736_MOESM11_ESM.zip › Figure 6/Images/Control LTL.tif]

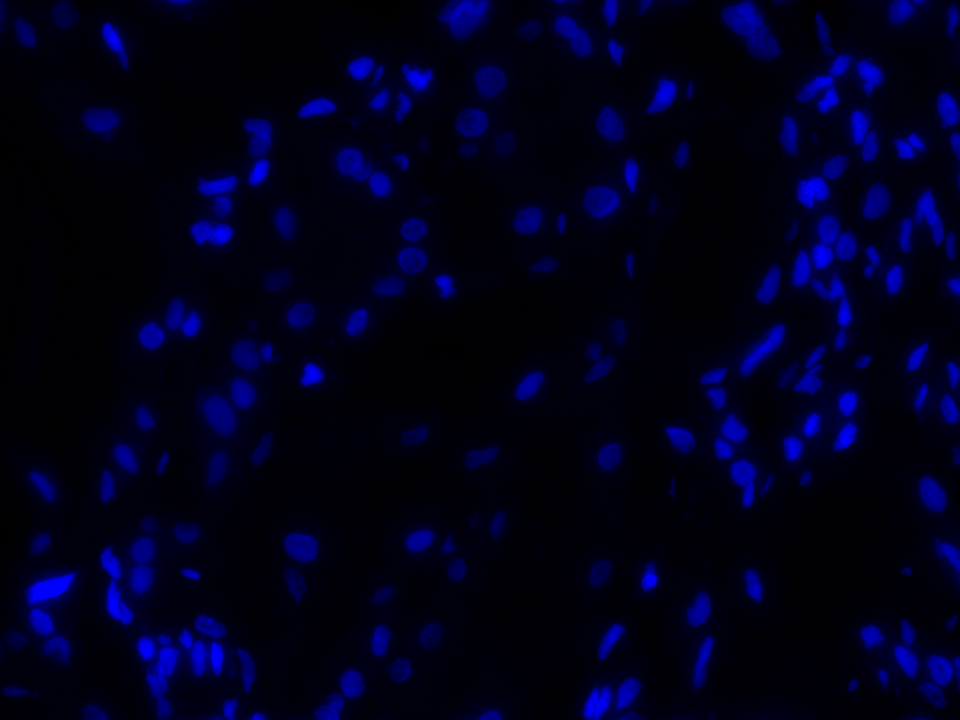

Supplement: Supplementary file 11 — Source data Fig. 6 [file 44319_2026_736_MOESM11_ESM.zip › Figure 6/Images/Control DAPI.tif]
