# Supplementary figures and images for: Cystinosin regulates Na+/H+ exchanger 3 trafficking and function in kidney proximal tubular cells (part 2 of 2)
Source: EMBO Rep. 2026 Mar 24;27(8):2088–117. doi: 10.1038/s44319-026-00736-1 (PMC13121807; doi:10.1038/s44319-026-00736-1)

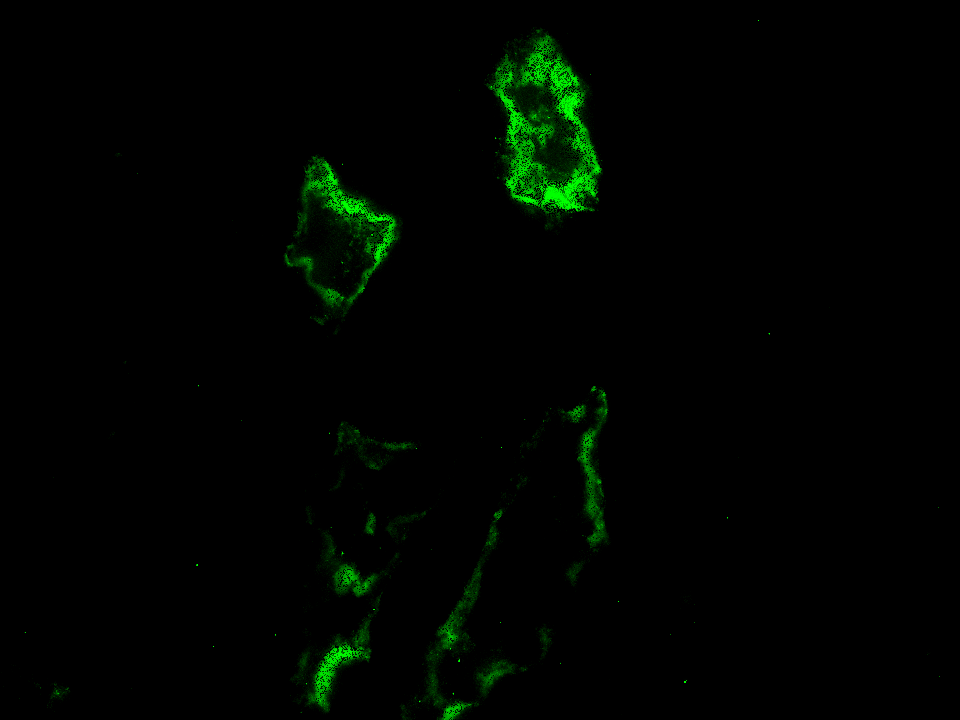

Supplement: Supplementary file 11 — Source data Fig. 6 [file 44319_2026_736_MOESM11_ESM.zip › Figure 6/Images/Control NHE3.tif]

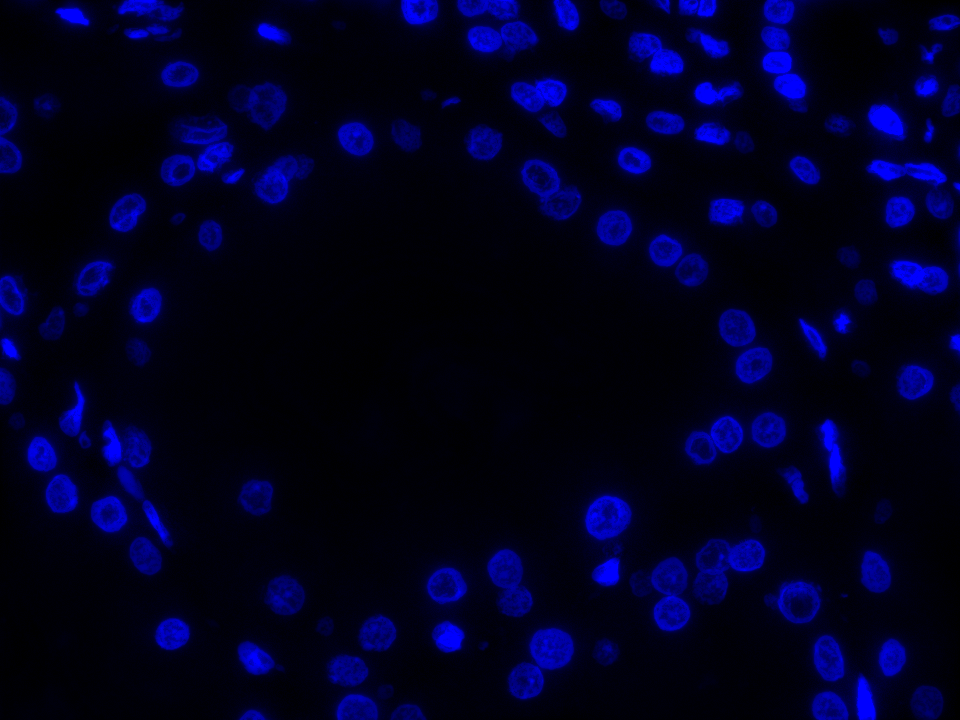

Supplement: Supplementary file 11 — Source data Fig. 6 [file 44319_2026_736_MOESM11_ESM.zip › Figure 6/Images/Cystinosis_DAPI.tif]

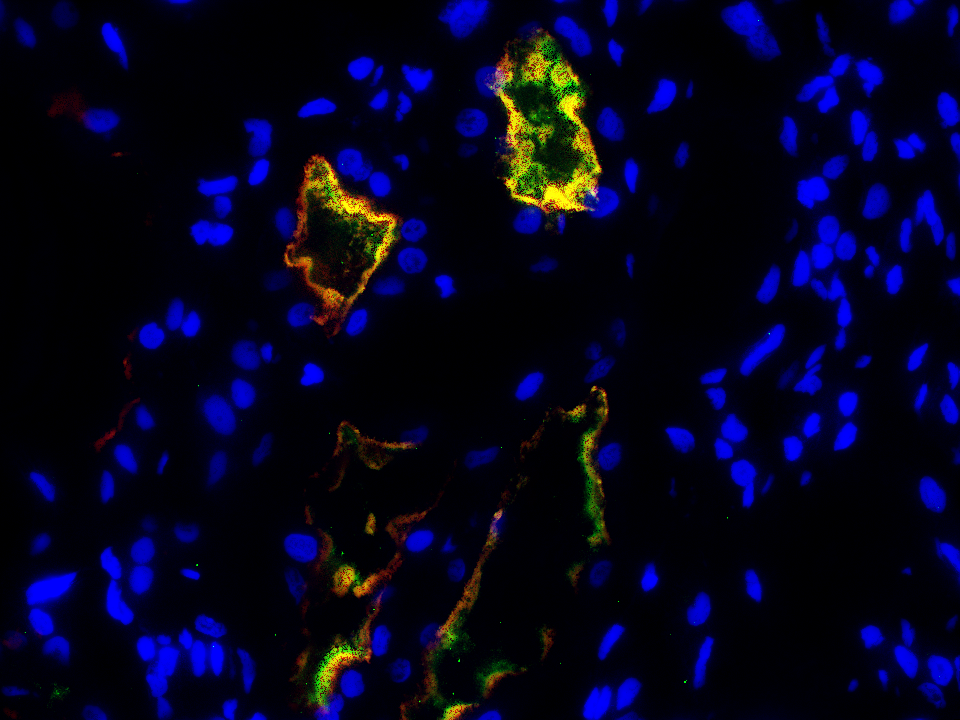

Supplement: Supplementary file 11 — Source data Fig. 6 [file 44319_2026_736_MOESM11_ESM.zip › Figure 6/Images/Control Merged.tif]

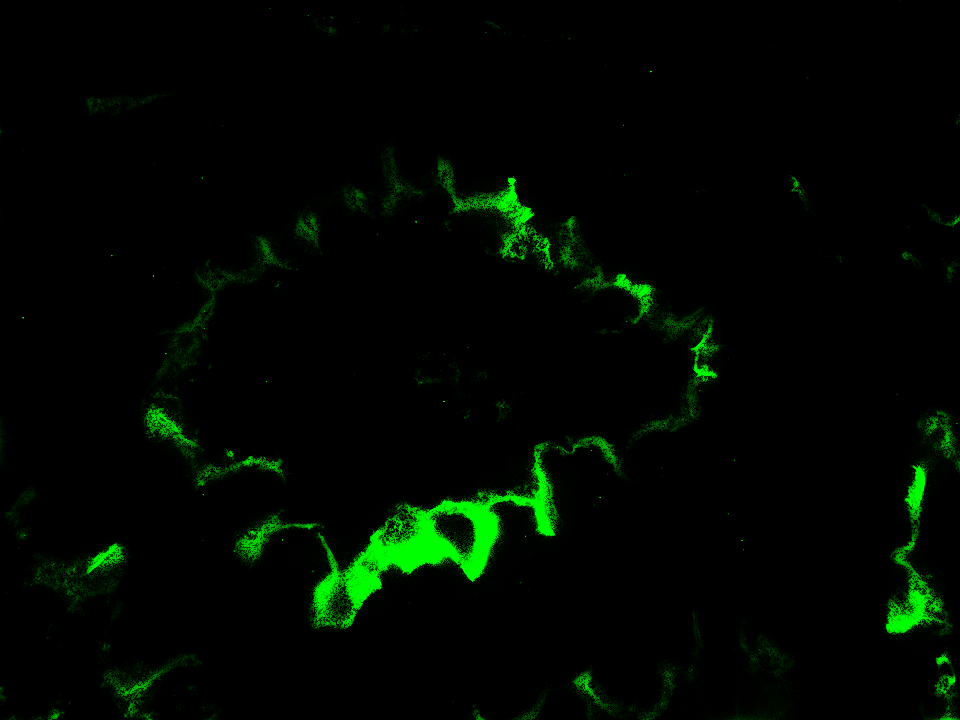

Supplement: Supplementary file 11 — Source data Fig. 6 [file 44319_2026_736_MOESM11_ESM.zip › Figure 6/Images/Cystinosis_NHE3.tif]

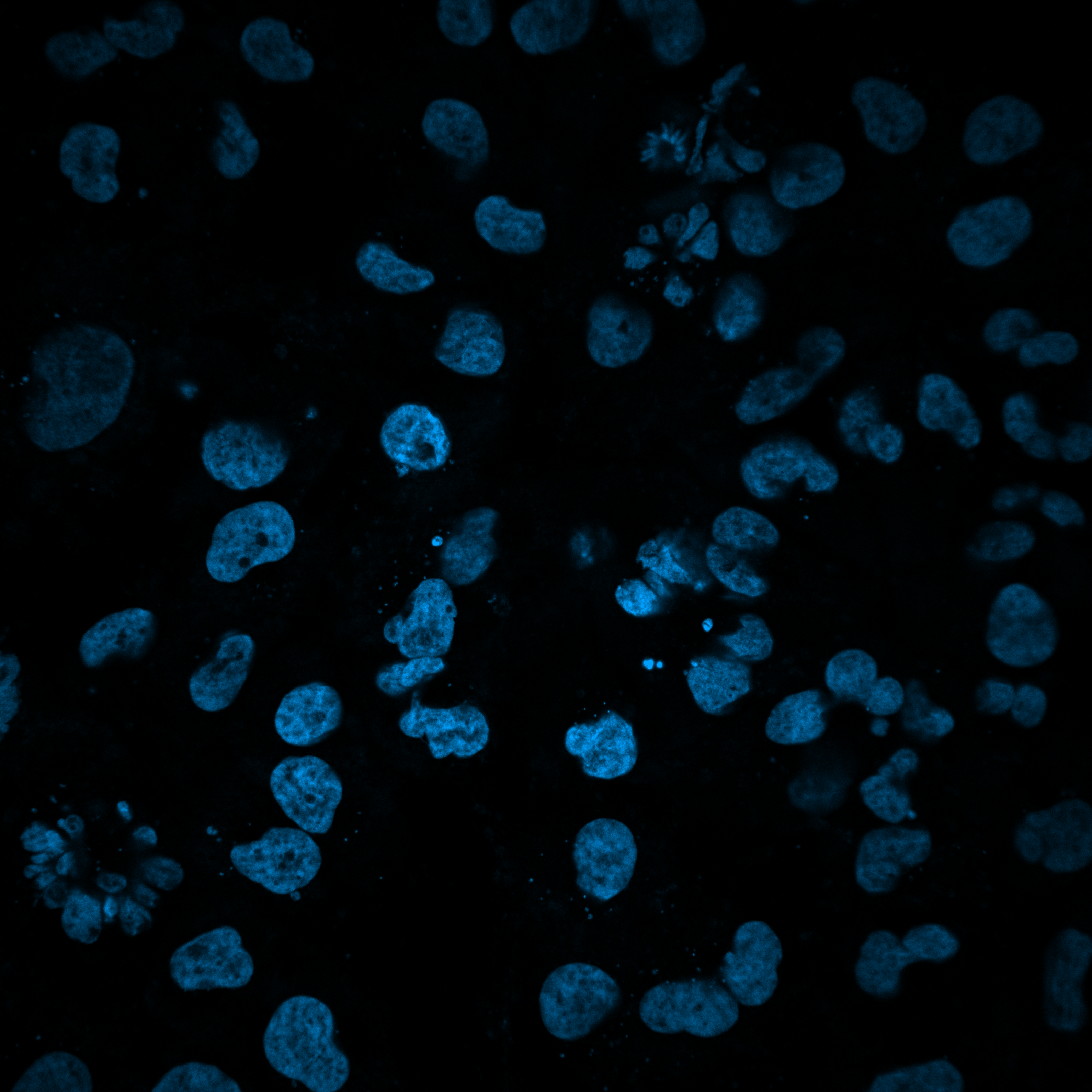

Supplement: Supplementary file 12 — Figure EV3 Source Data [file 44319_2026_736_MOESM12_ESM.zip › Figure EV3/EV3D/KO/KO cysteamine_DAPI.tif]

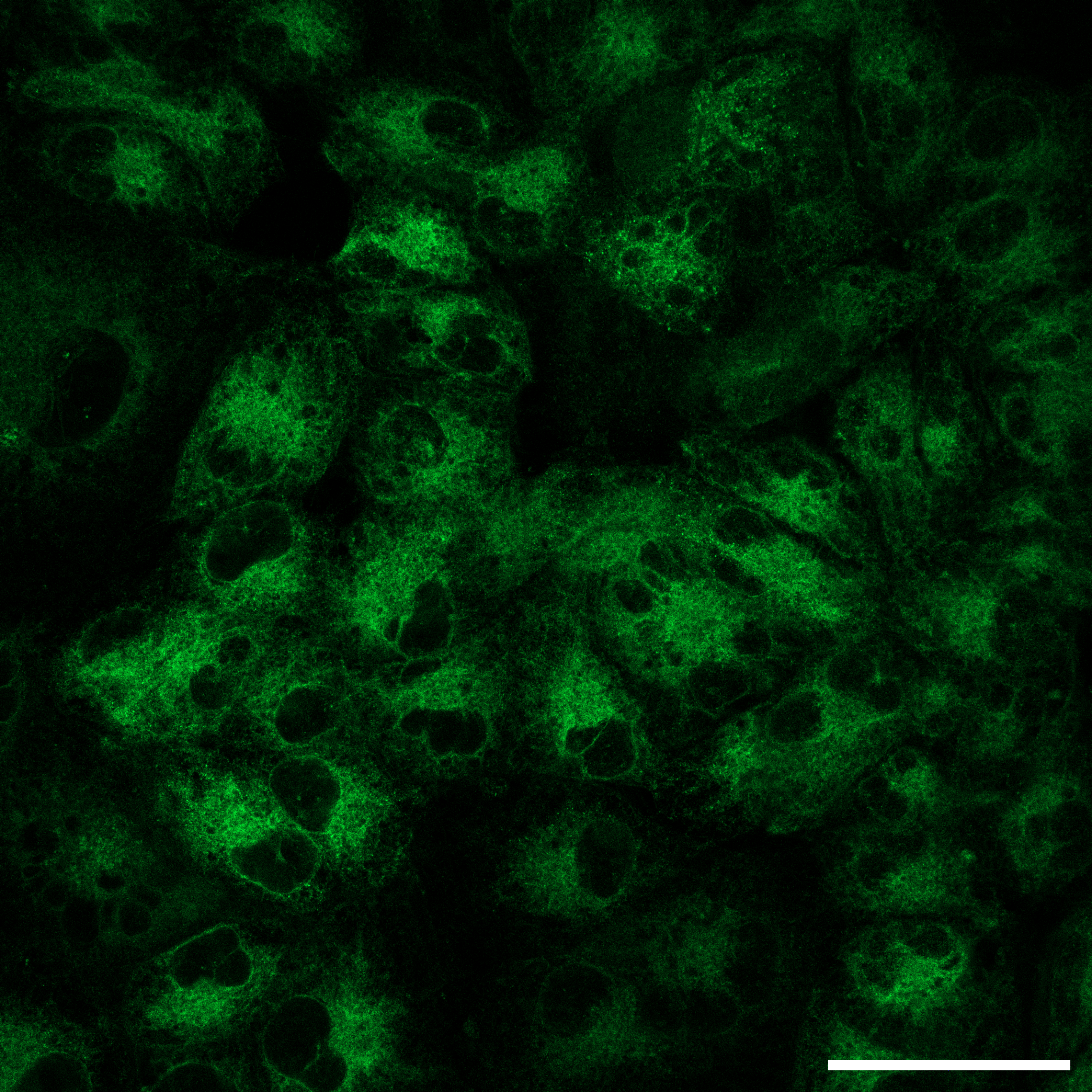

Supplement: Supplementary file 12 — Figure EV3 Source Data [file 44319_2026_736_MOESM12_ESM.zip › Figure EV3/EV3D/KO/KO cysteamine-NHE3.tif]

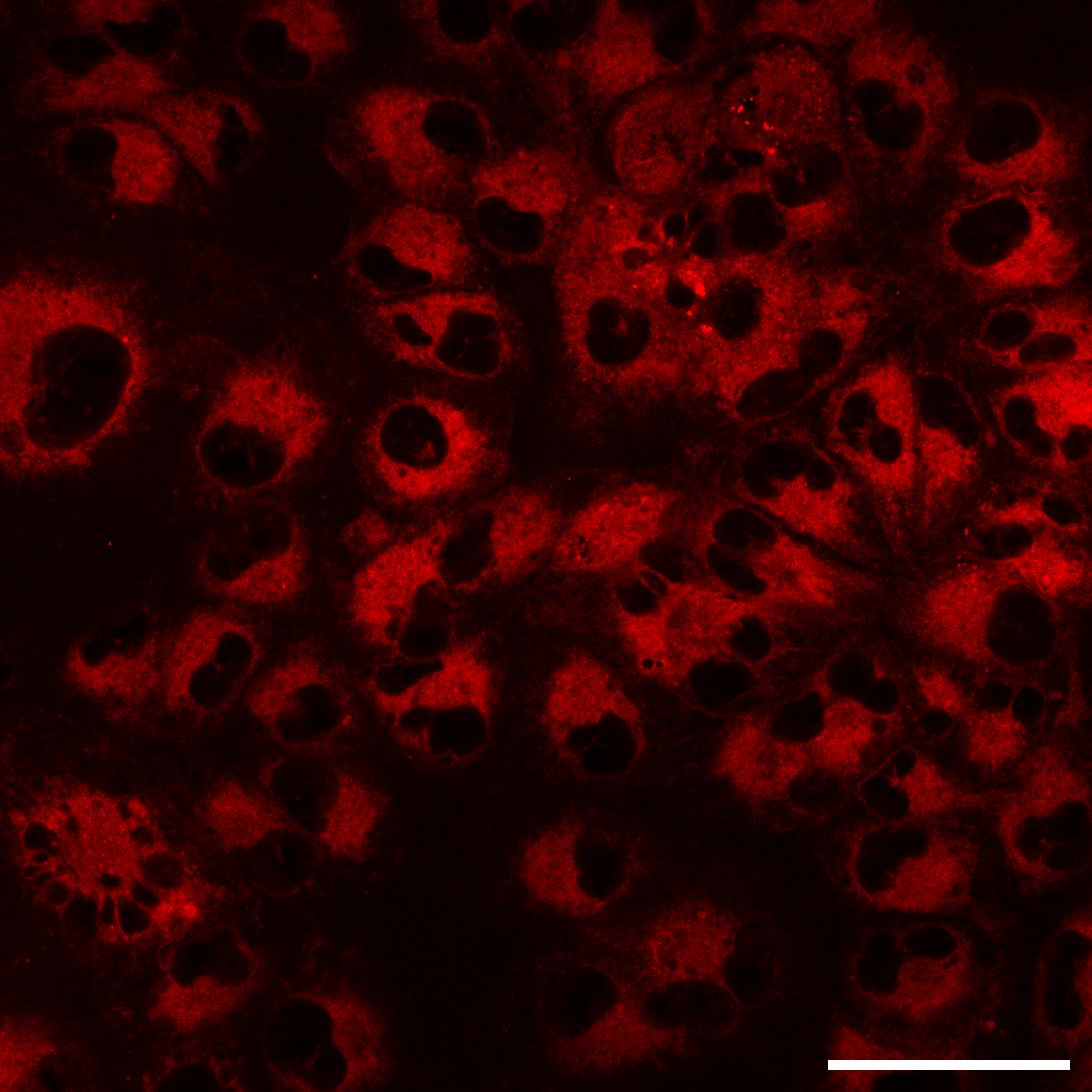

Supplement: Supplementary file 12 — Figure EV3 Source Data [file 44319_2026_736_MOESM12_ESM.zip › Figure EV3/EV3D/KO/KO cysteamine_ER Tracker.tif]

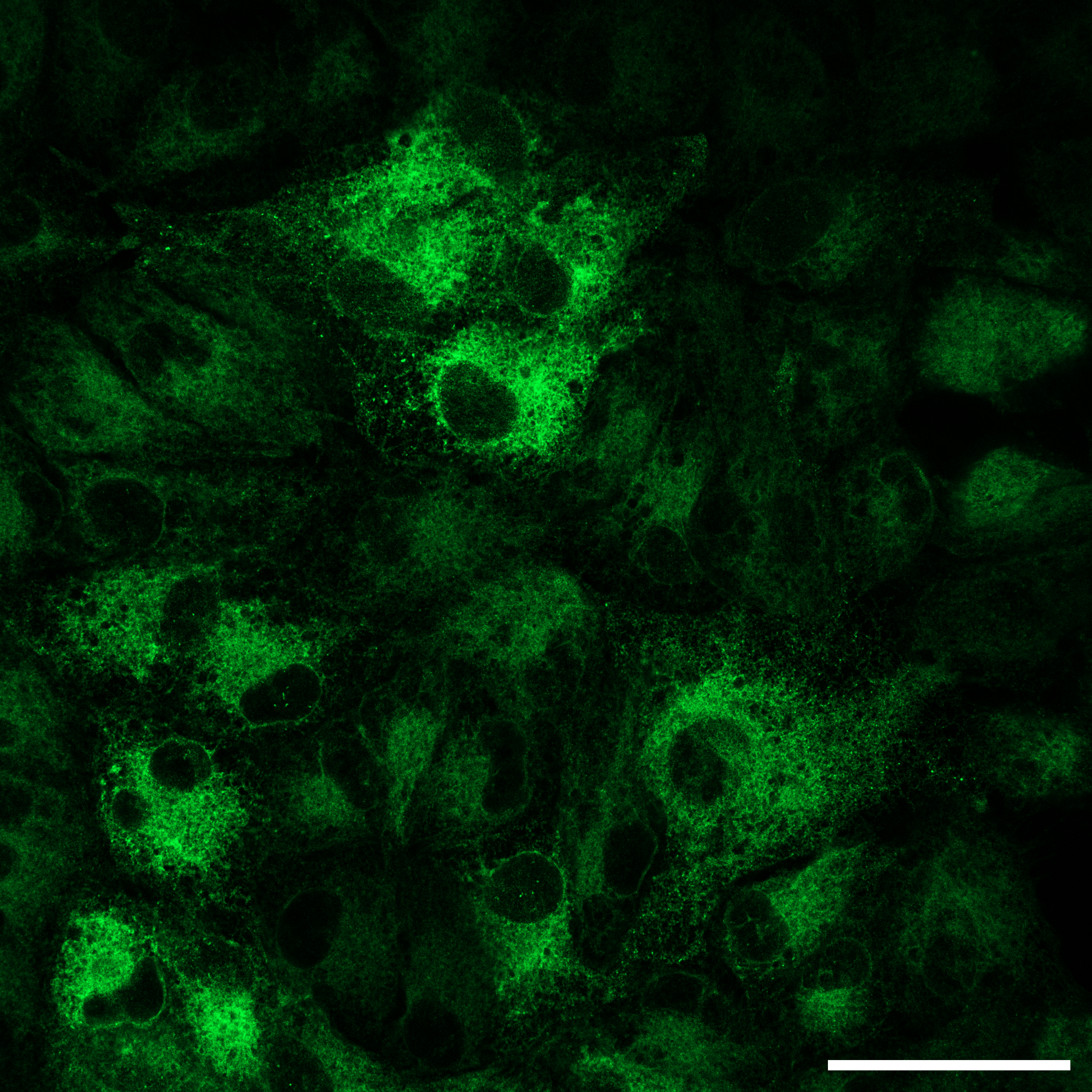

Supplement: Supplementary file 12 — Figure EV3 Source Data [file 44319_2026_736_MOESM12_ESM.zip › Figure EV3/EV3D/KO/KO control_NHE3.tif]

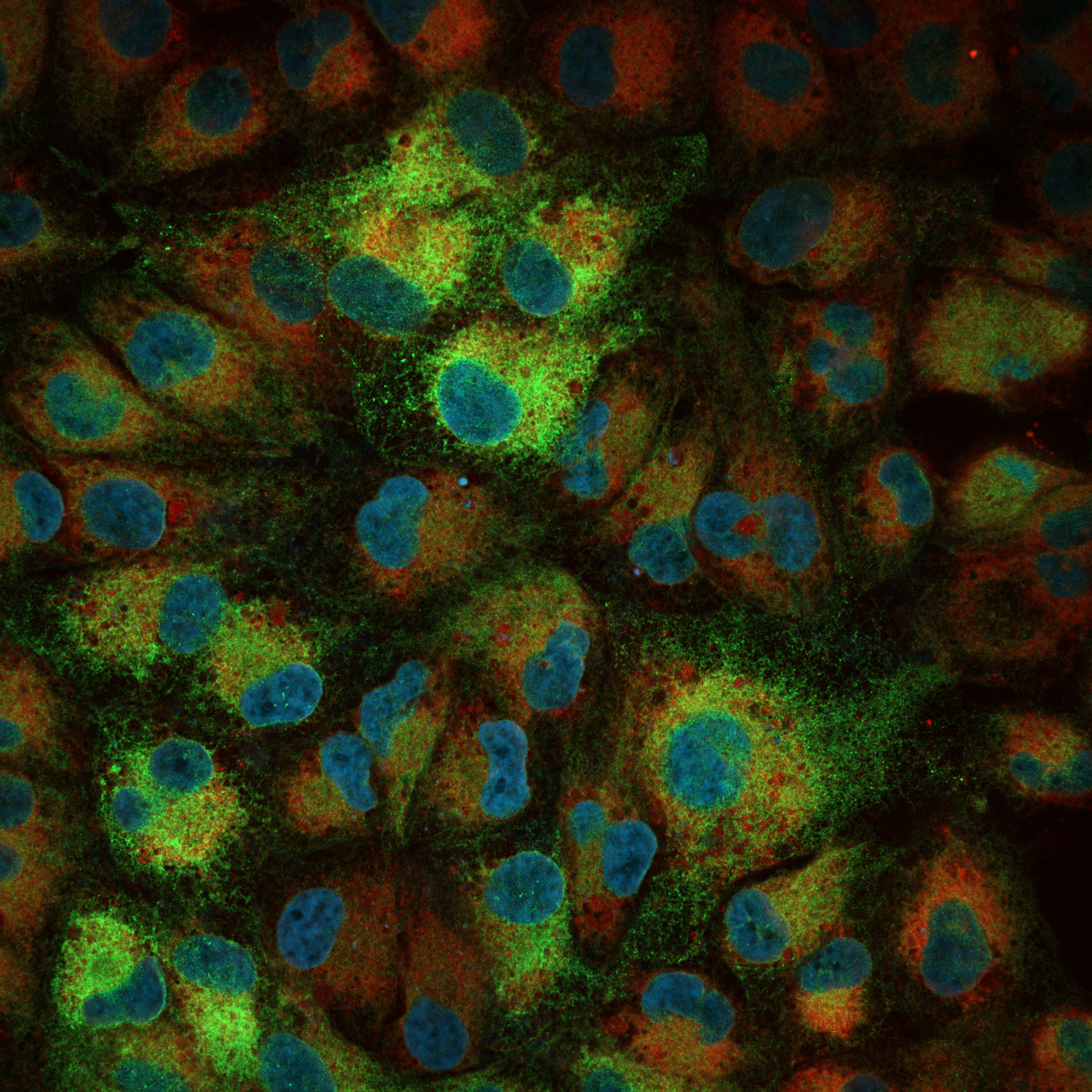

Supplement: Supplementary file 12 — Figure EV3 Source Data [file 44319_2026_736_MOESM12_ESM.zip › Figure EV3/EV3D/KO/KO control_Merged.tif]

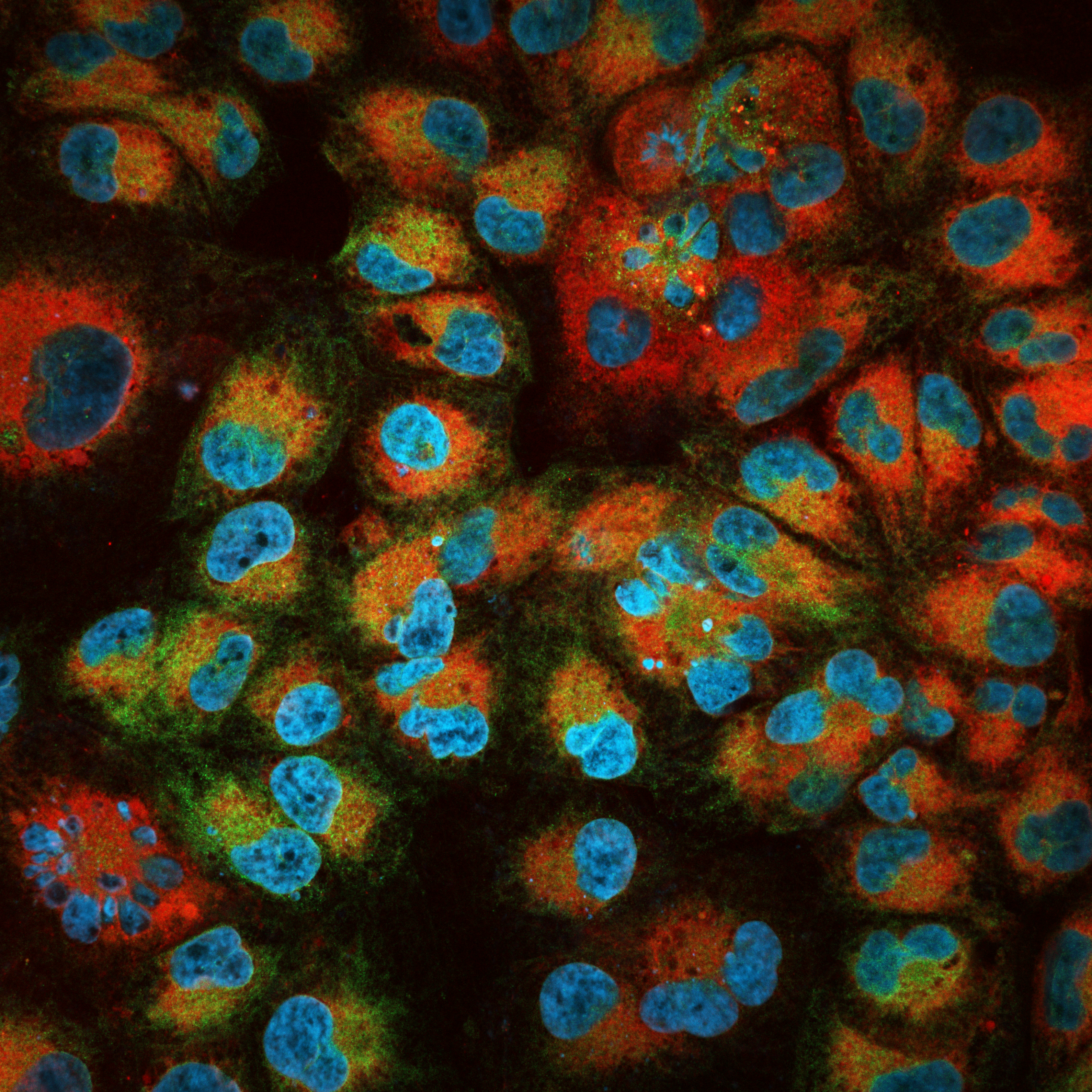

Supplement: Supplementary file 12 — Figure EV3 Source Data [file 44319_2026_736_MOESM12_ESM.zip › Figure EV3/EV3D/KO/KO cysteamine_Merged.tif]

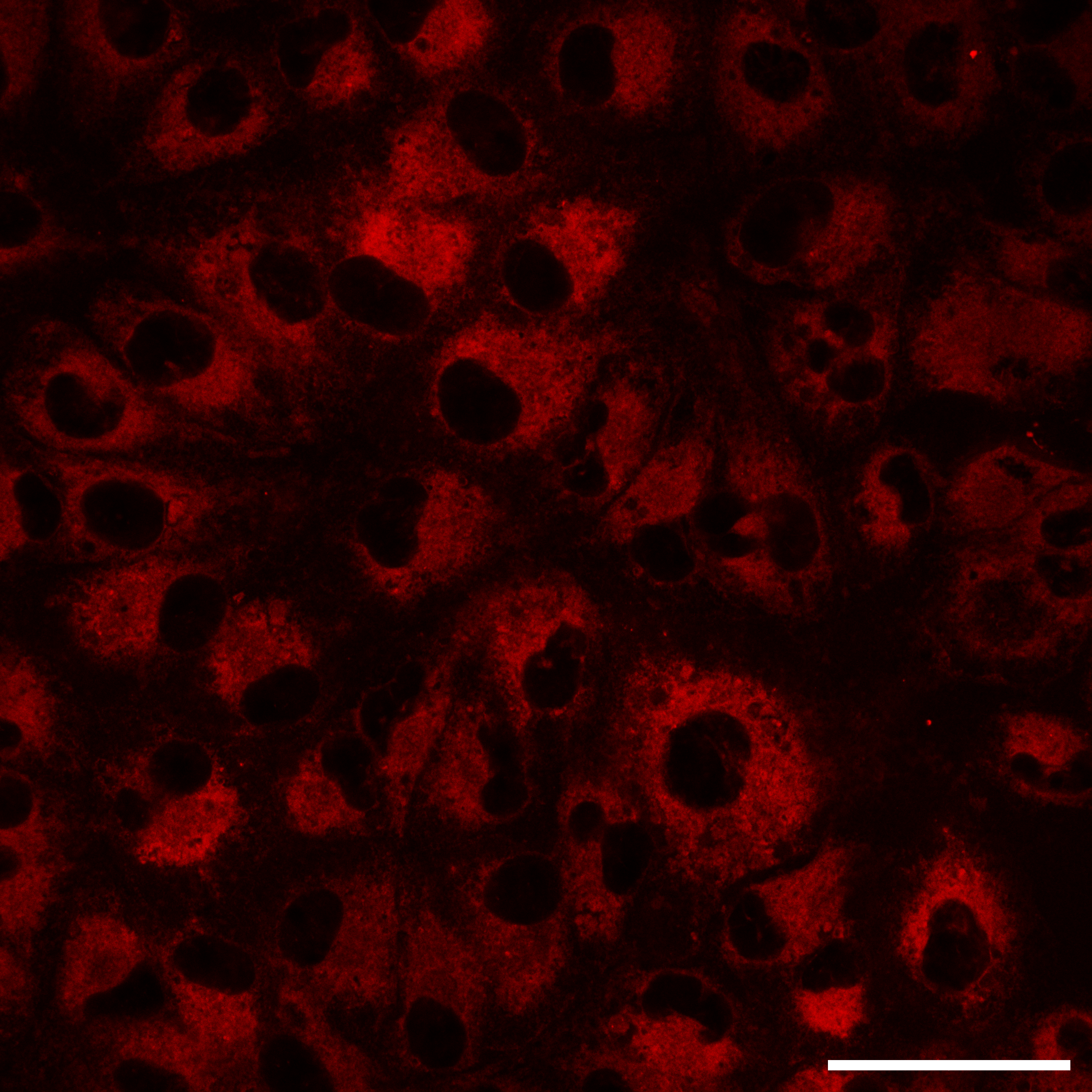

Supplement: Supplementary file 12 — Figure EV3 Source Data [file 44319_2026_736_MOESM12_ESM.zip › Figure EV3/EV3D/KO/KO control_ER tracker.tif]

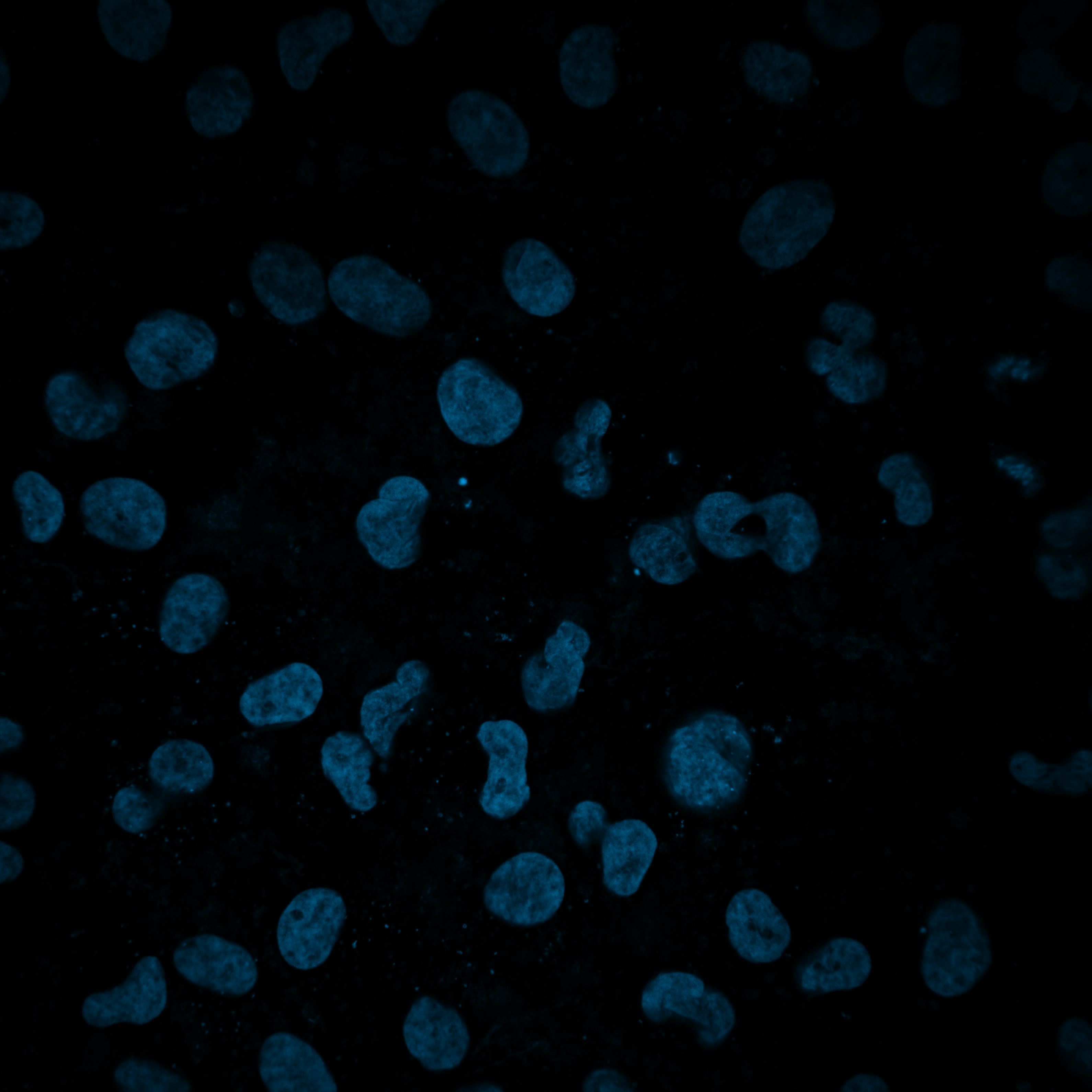

Supplement: Supplementary file 12 — Figure EV3 Source Data [file 44319_2026_736_MOESM12_ESM.zip › Figure EV3/EV3D/KO/KO control_DAPI.tif]

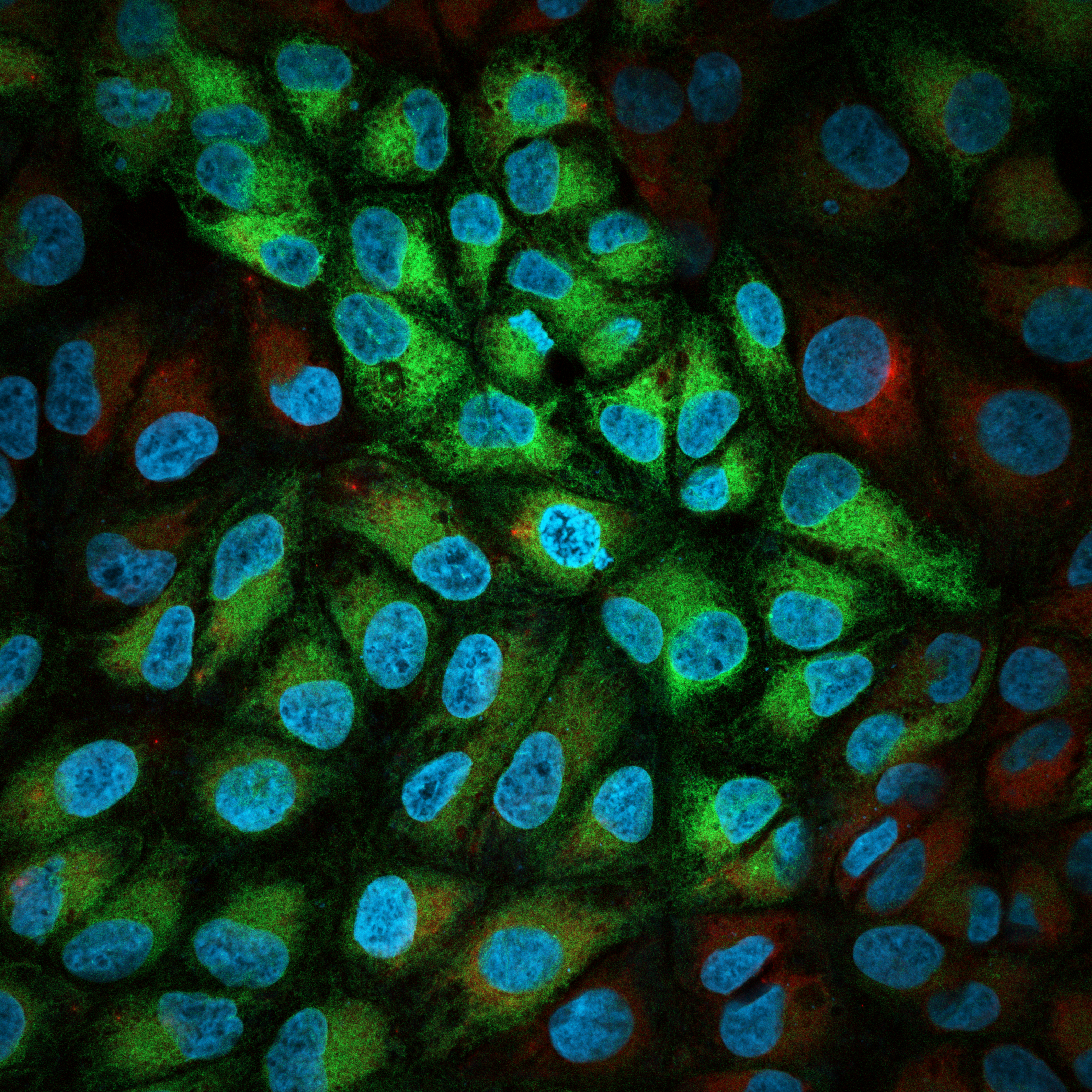

Supplement: Supplementary file 12 — Figure EV3 Source Data [file 44319_2026_736_MOESM12_ESM.zip › Figure EV3/EV3D/WT/WT Cysteamine_Merged.tif]

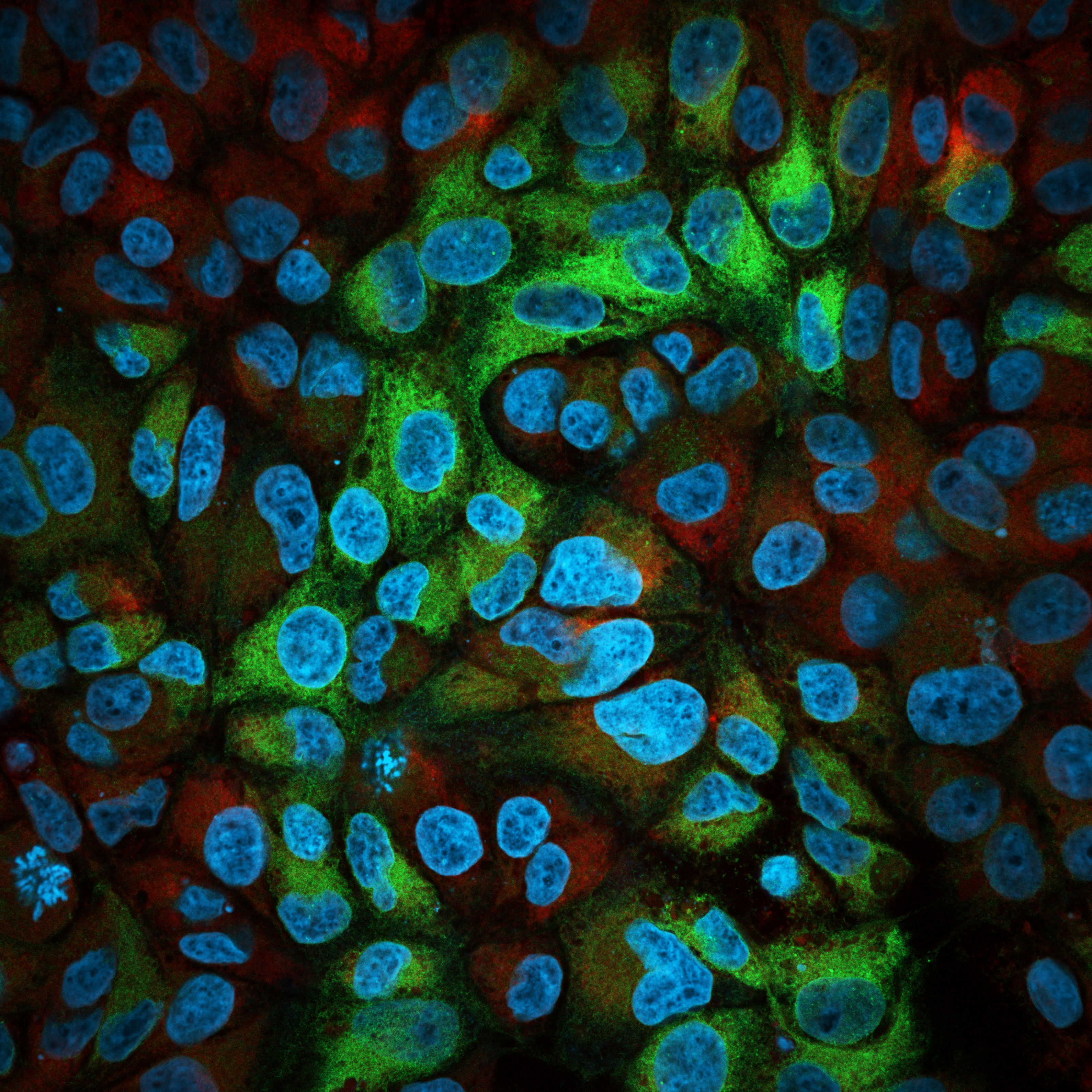

Supplement: Supplementary file 12 — Figure EV3 Source Data [file 44319_2026_736_MOESM12_ESM.zip › Figure EV3/EV3D/WT/WT control_Merged.tif]

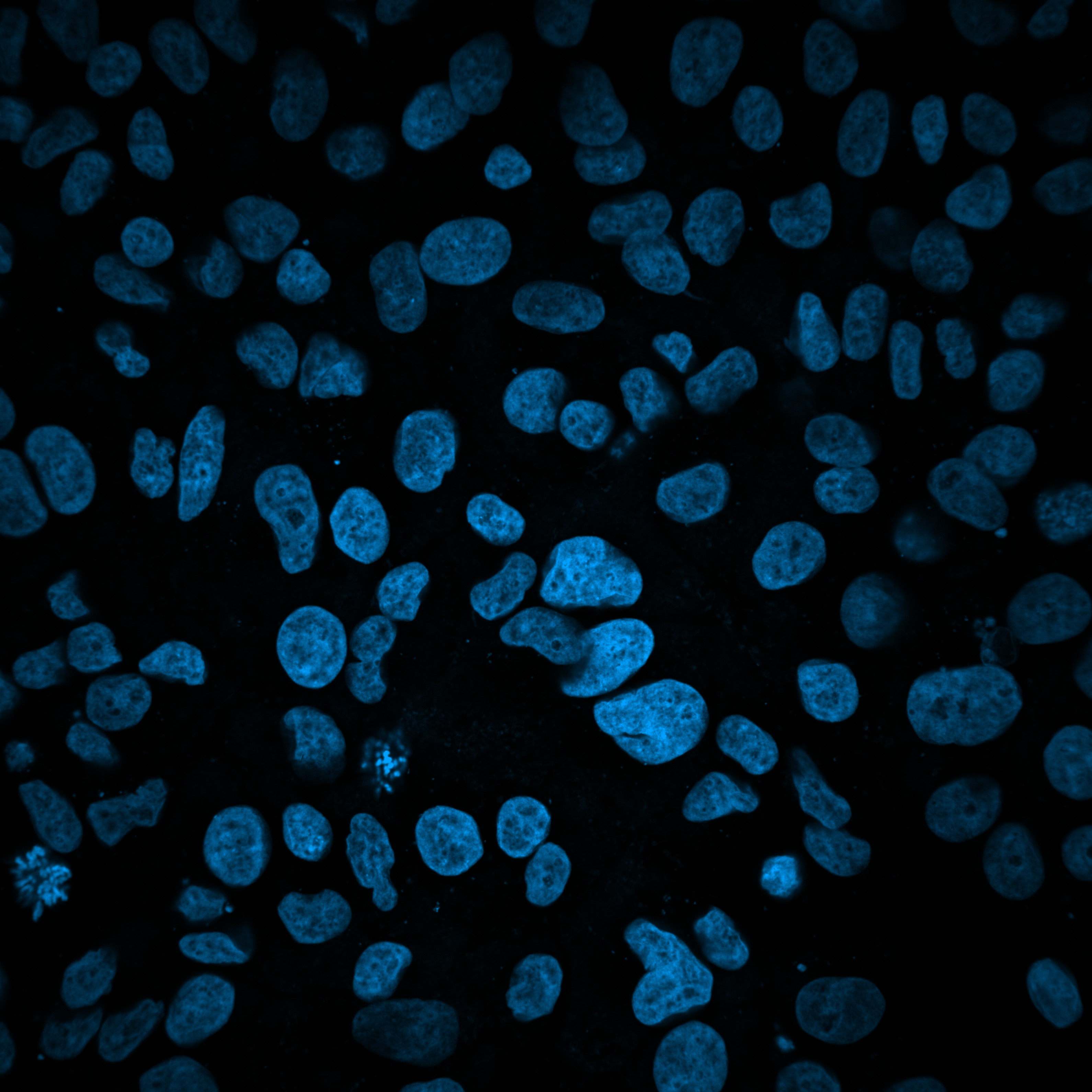

Supplement: Supplementary file 12 — Figure EV3 Source Data [file 44319_2026_736_MOESM12_ESM.zip › Figure EV3/EV3D/WT/WT control_DAPI.tif]

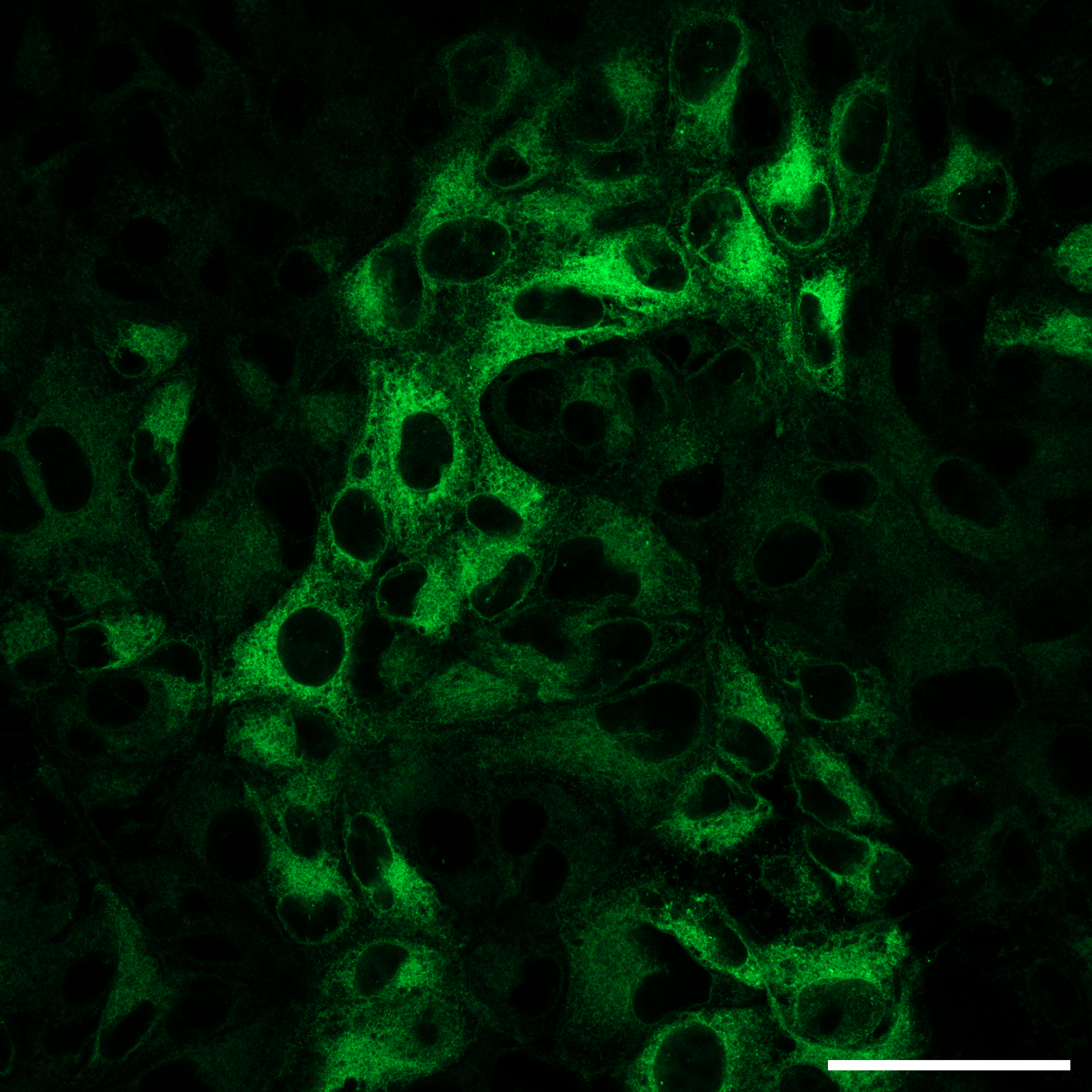

Supplement: Supplementary file 12 — Figure EV3 Source Data [file 44319_2026_736_MOESM12_ESM.zip › Figure EV3/EV3D/WT/WT control_NHE3.tif]

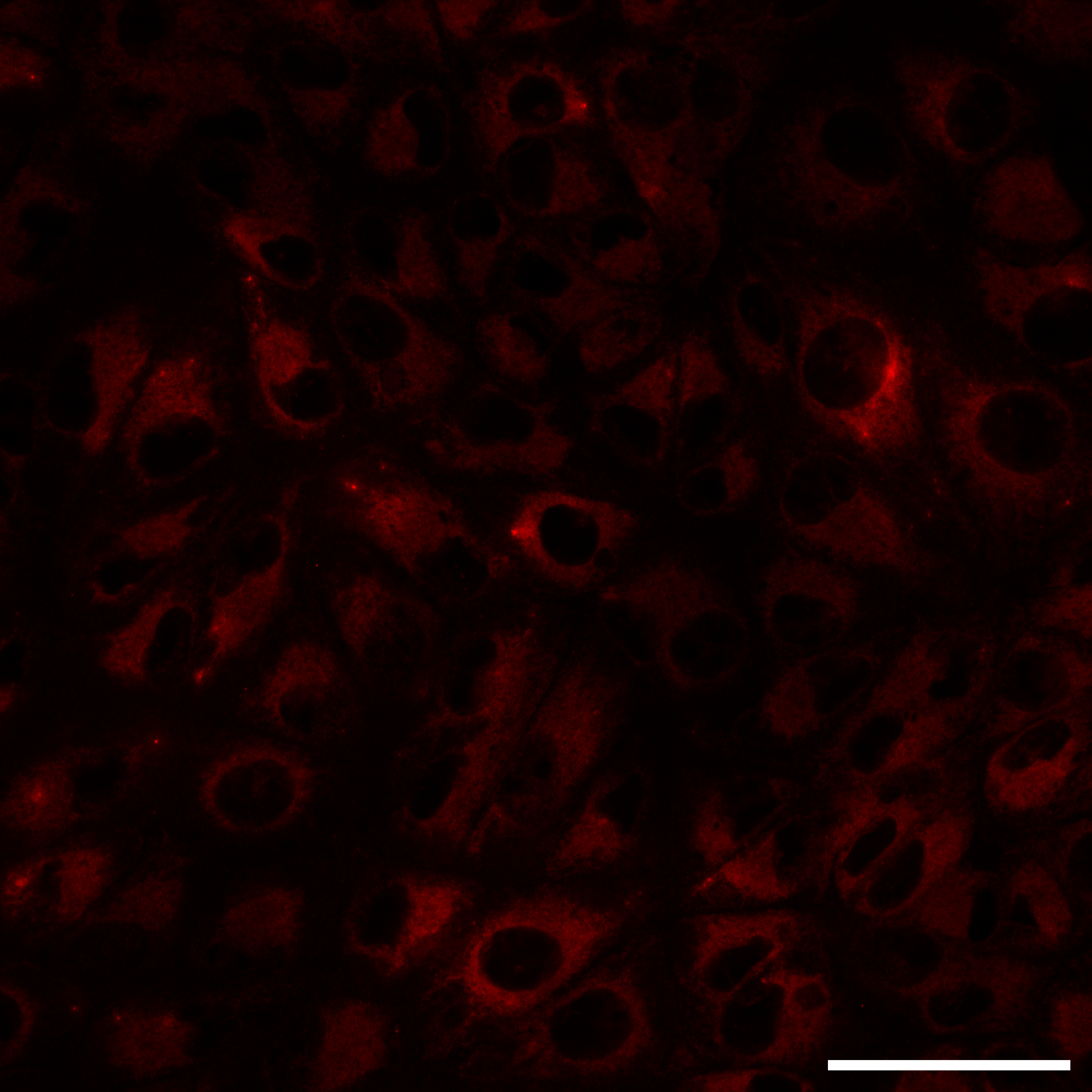

Supplement: Supplementary file 12 — Figure EV3 Source Data [file 44319_2026_736_MOESM12_ESM.zip › Figure EV3/EV3D/WT/WT Cysteamine_ER Tracker.tif]

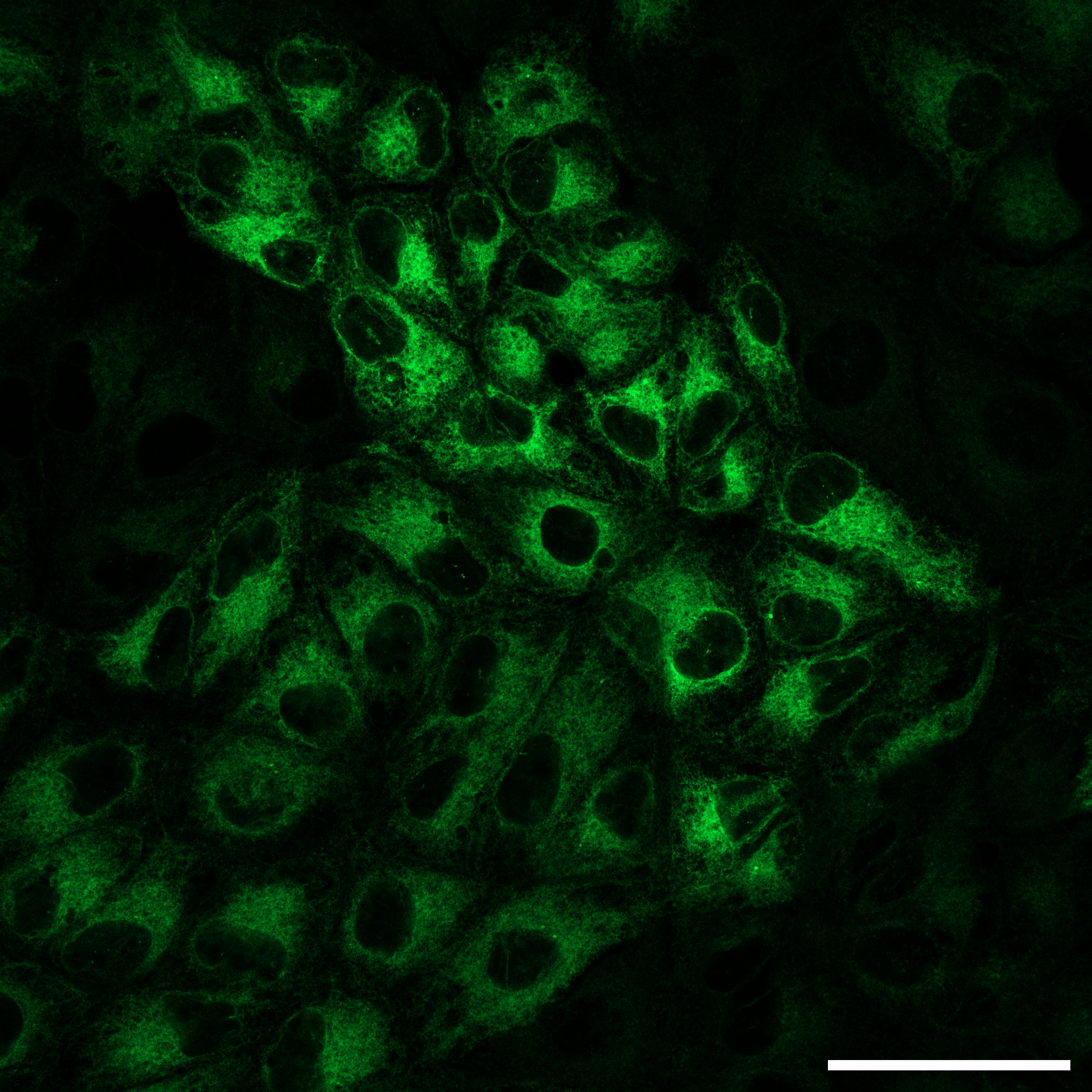

Supplement: Supplementary file 12 — Figure EV3 Source Data [file 44319_2026_736_MOESM12_ESM.zip › Figure EV3/EV3D/WT/WT Cysteamine_NHE3.tif]

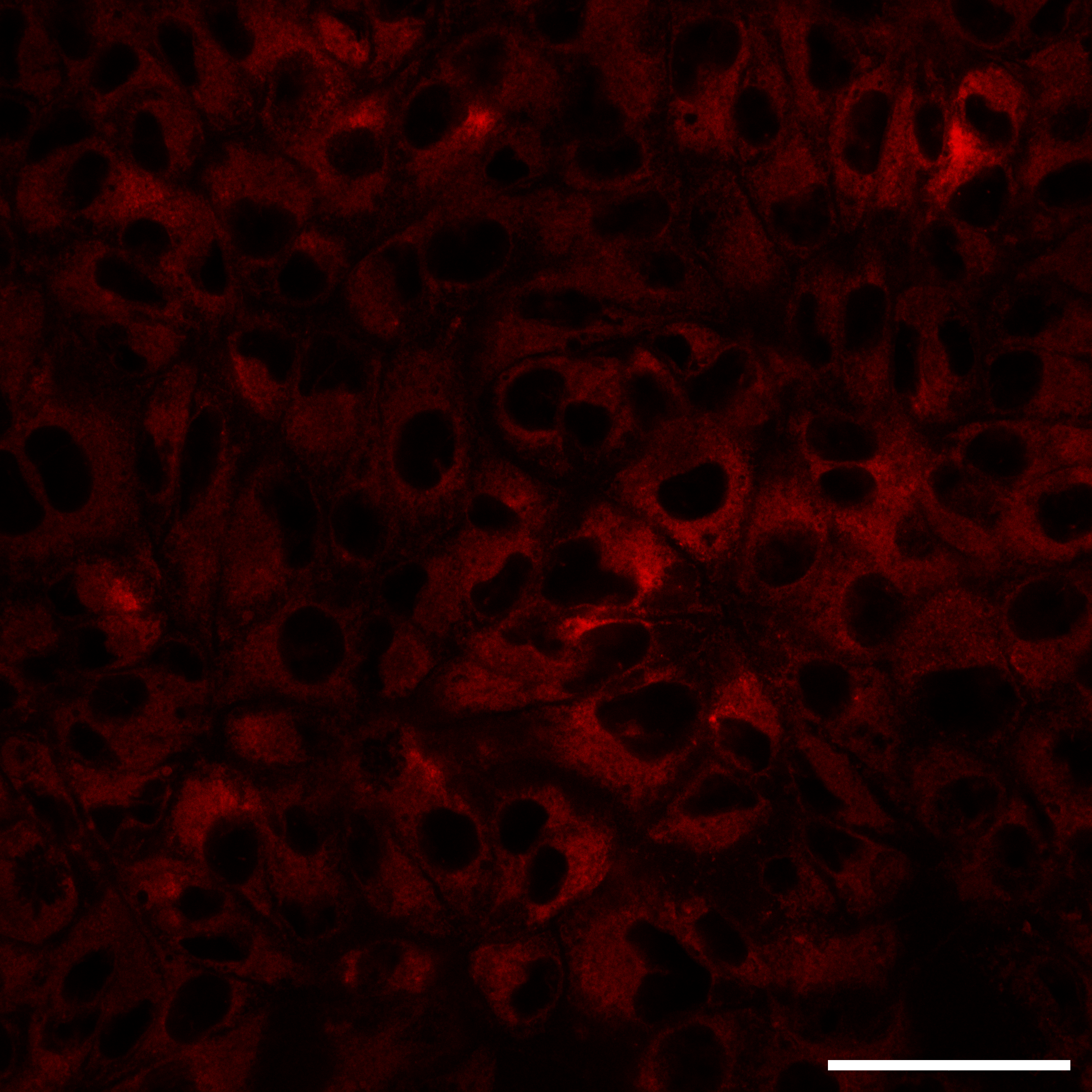

Supplement: Supplementary file 12 — Figure EV3 Source Data [file 44319_2026_736_MOESM12_ESM.zip › Figure EV3/EV3D/WT/WT control_ER Tracker.tif]

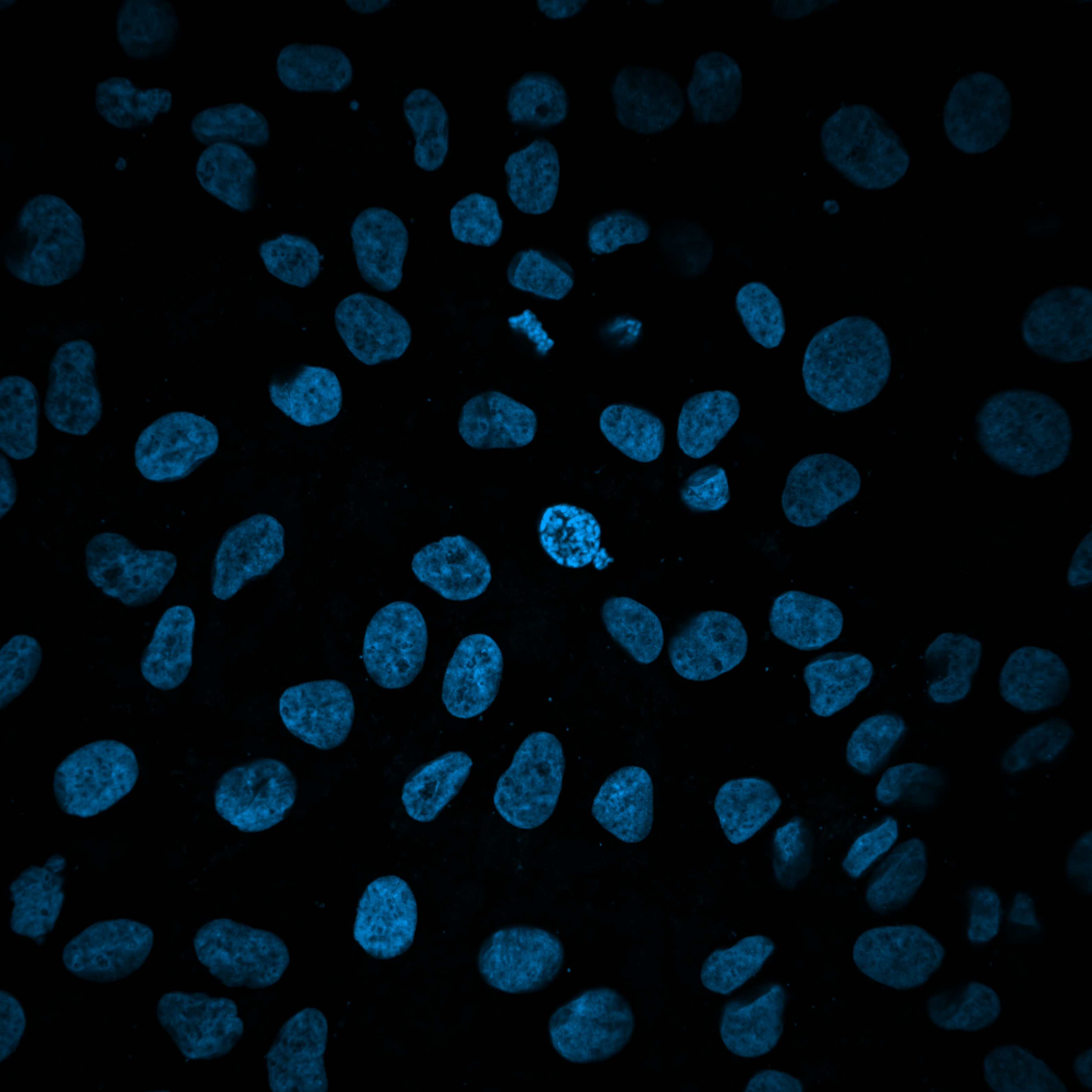

Supplement: Supplementary file 12 — Figure EV3 Source Data [file 44319_2026_736_MOESM12_ESM.zip › Figure EV3/EV3D/WT/WT Cysteamine_DAPI.tif]

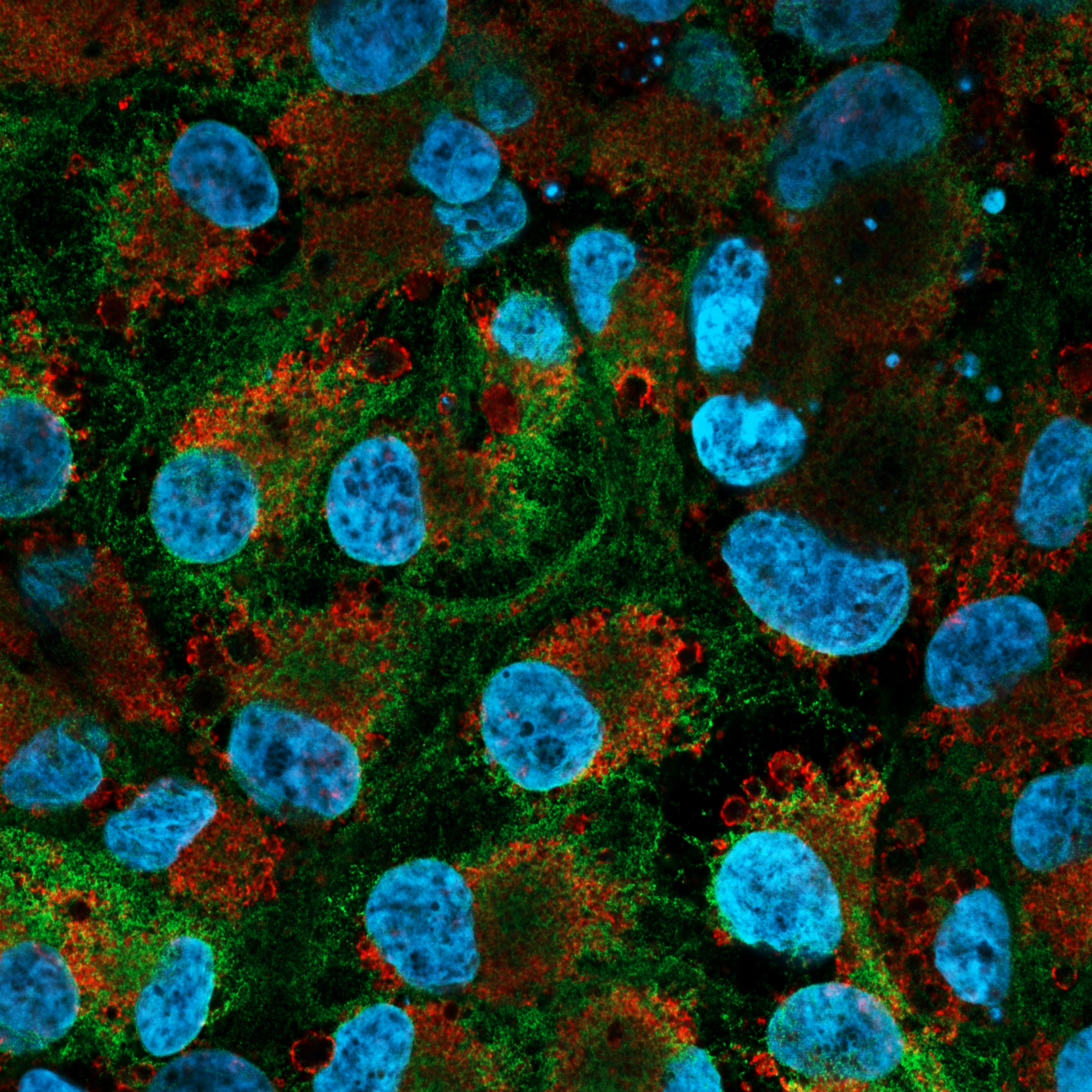

Supplement: Supplementary file 12 — Figure EV3 Source Data [file 44319_2026_736_MOESM12_ESM.zip › Figure EV3/EV3C/KO/Merged_HK-2_CTNS KO.tif]

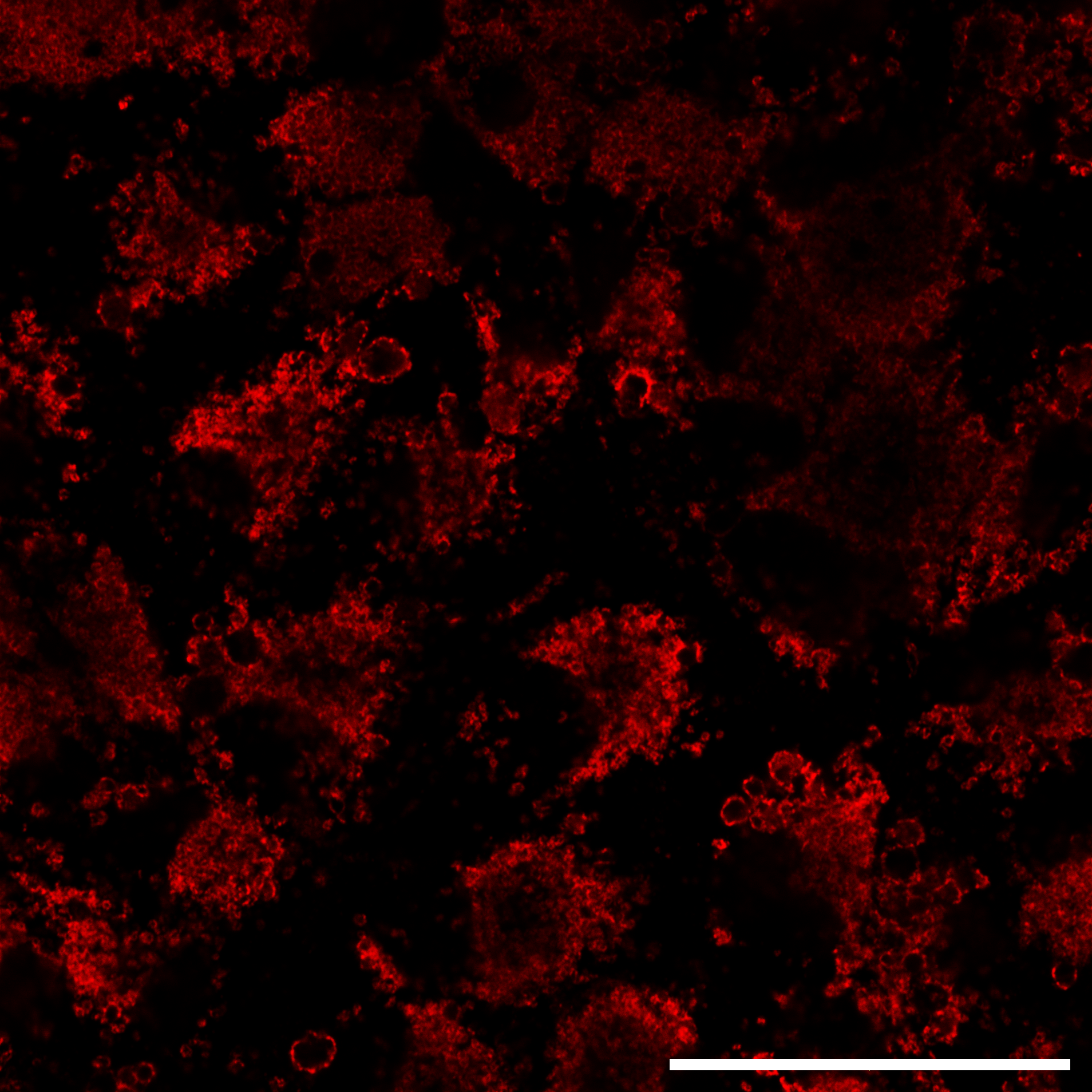

Supplement: Supplementary file 12 — Figure EV3 Source Data [file 44319_2026_736_MOESM12_ESM.zip › Figure EV3/EV3C/KO/LAMP1_HK-2_CTNS KO.tif]

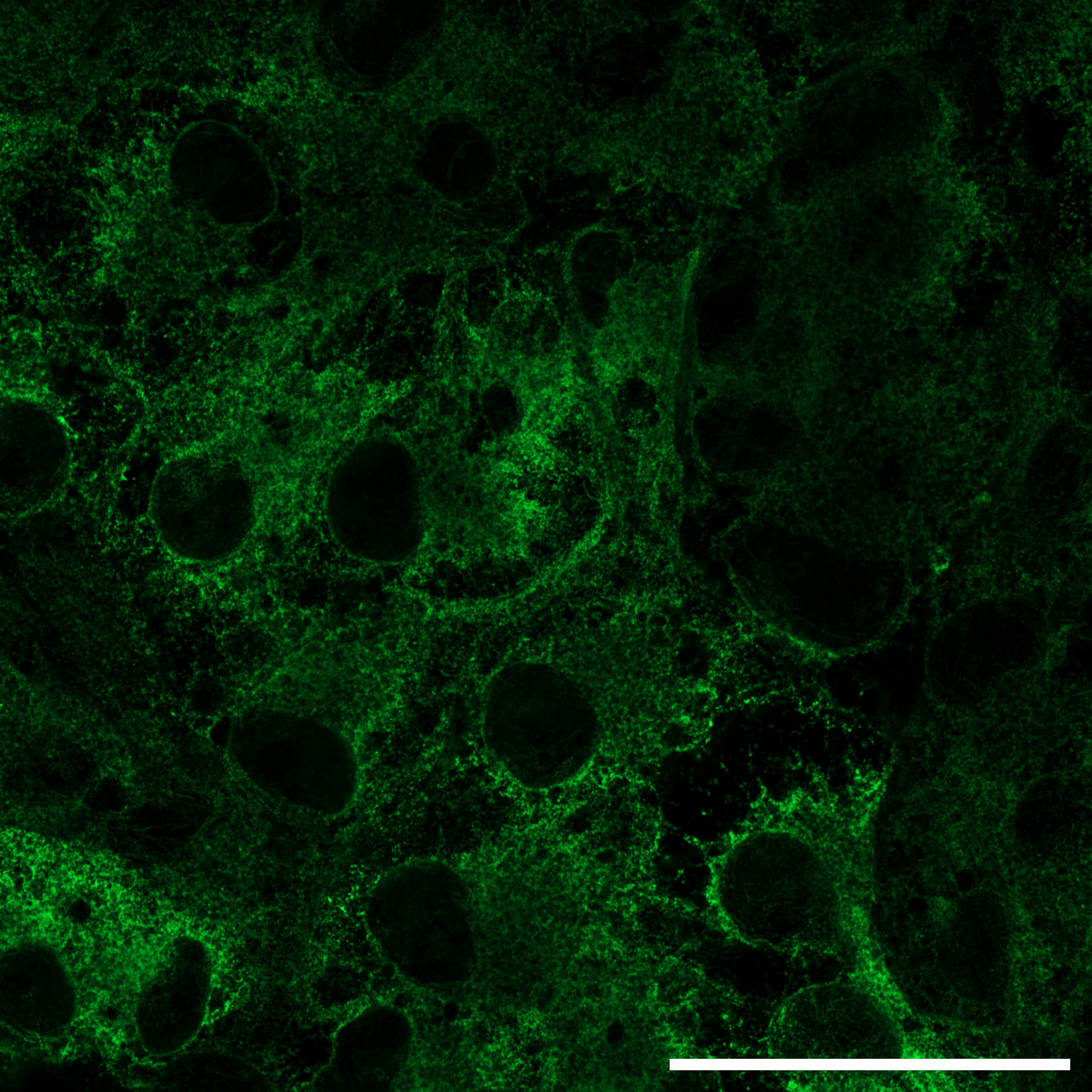

Supplement: Supplementary file 12 — Figure EV3 Source Data [file 44319_2026_736_MOESM12_ESM.zip › Figure EV3/EV3C/KO/NHE3-GFP_HK-2_CTNS KO.tif]

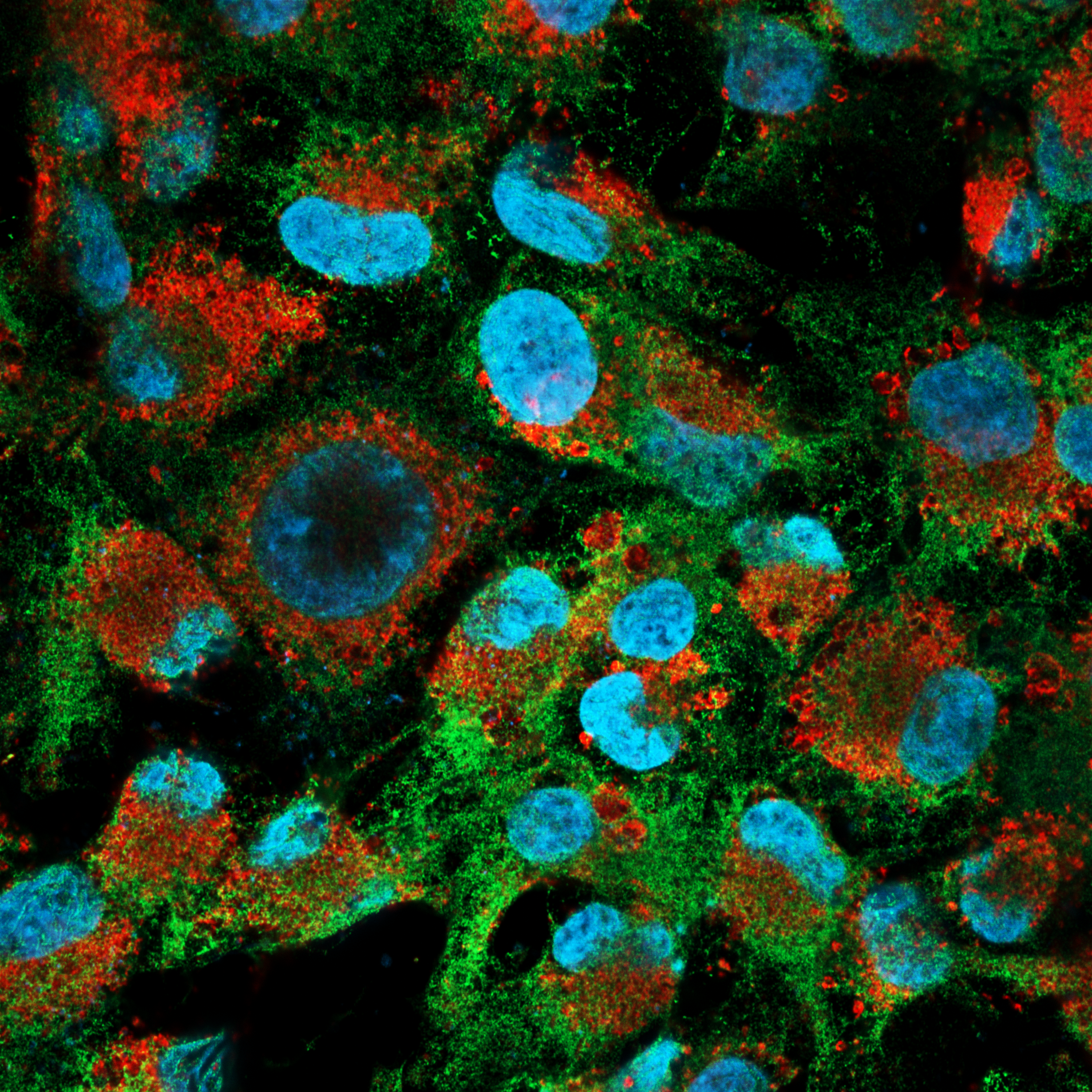

Supplement: Supplementary file 12 — Figure EV3 Source Data [file 44319_2026_736_MOESM12_ESM.zip › Figure EV3/EV3C/WT/Merged_HK-2_WT.tif]

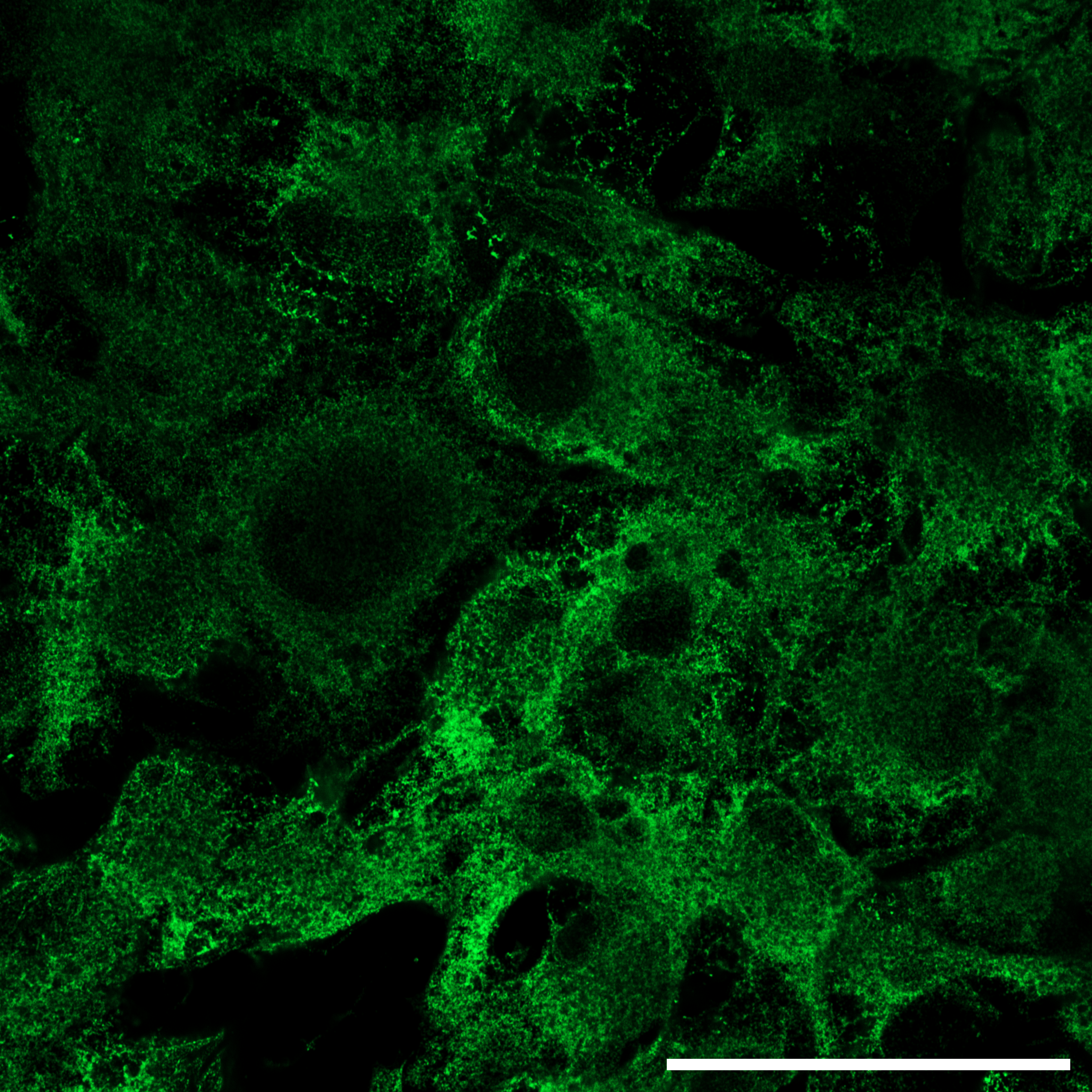

Supplement: Supplementary file 12 — Figure EV3 Source Data [file 44319_2026_736_MOESM12_ESM.zip › Figure EV3/EV3C/WT/NHE3-GFP_HK-2_WT.tif]

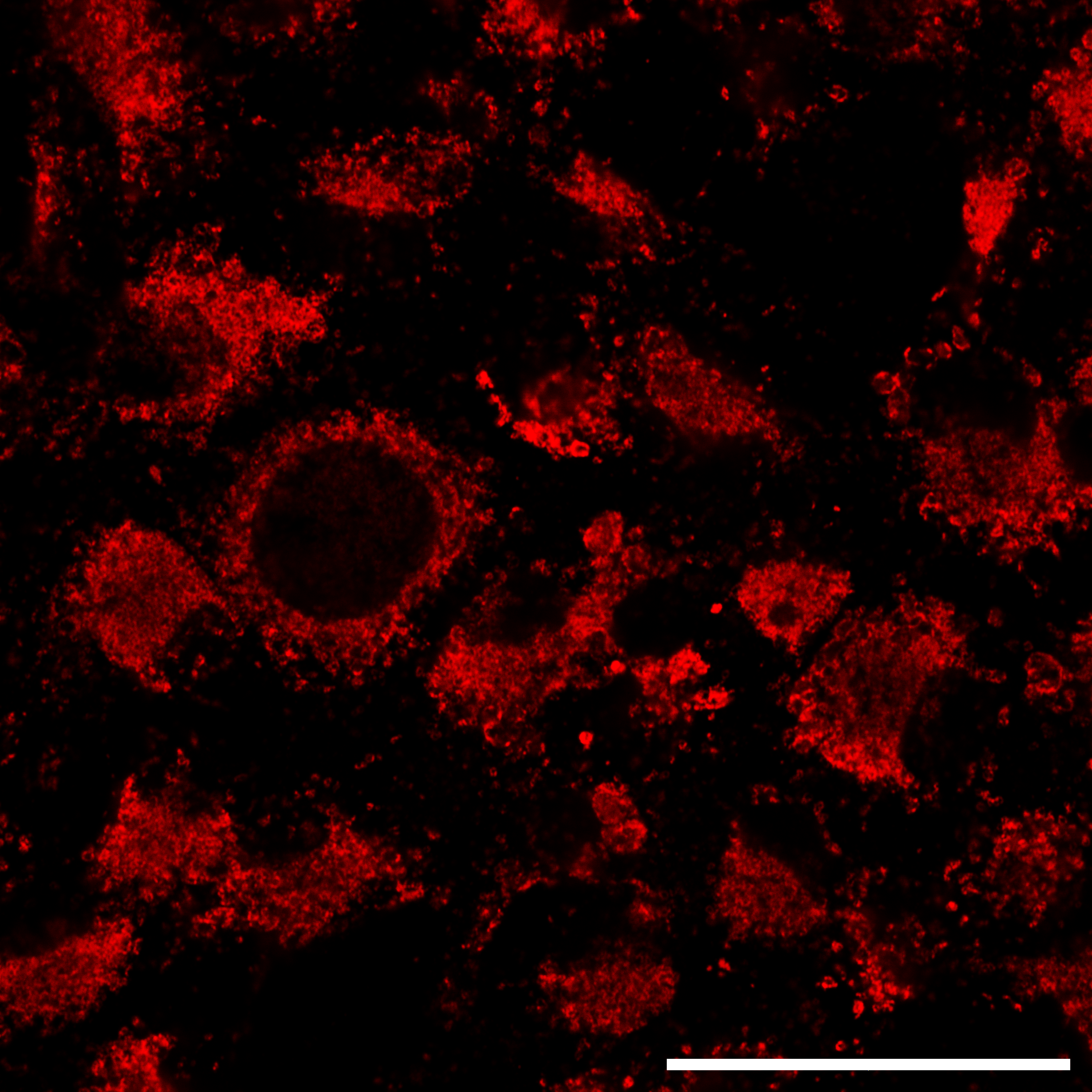

Supplement: Supplementary file 12 — Figure EV3 Source Data [file 44319_2026_736_MOESM12_ESM.zip › Figure EV3/EV3C/WT/LAMP1_HK-2 WT.tif]

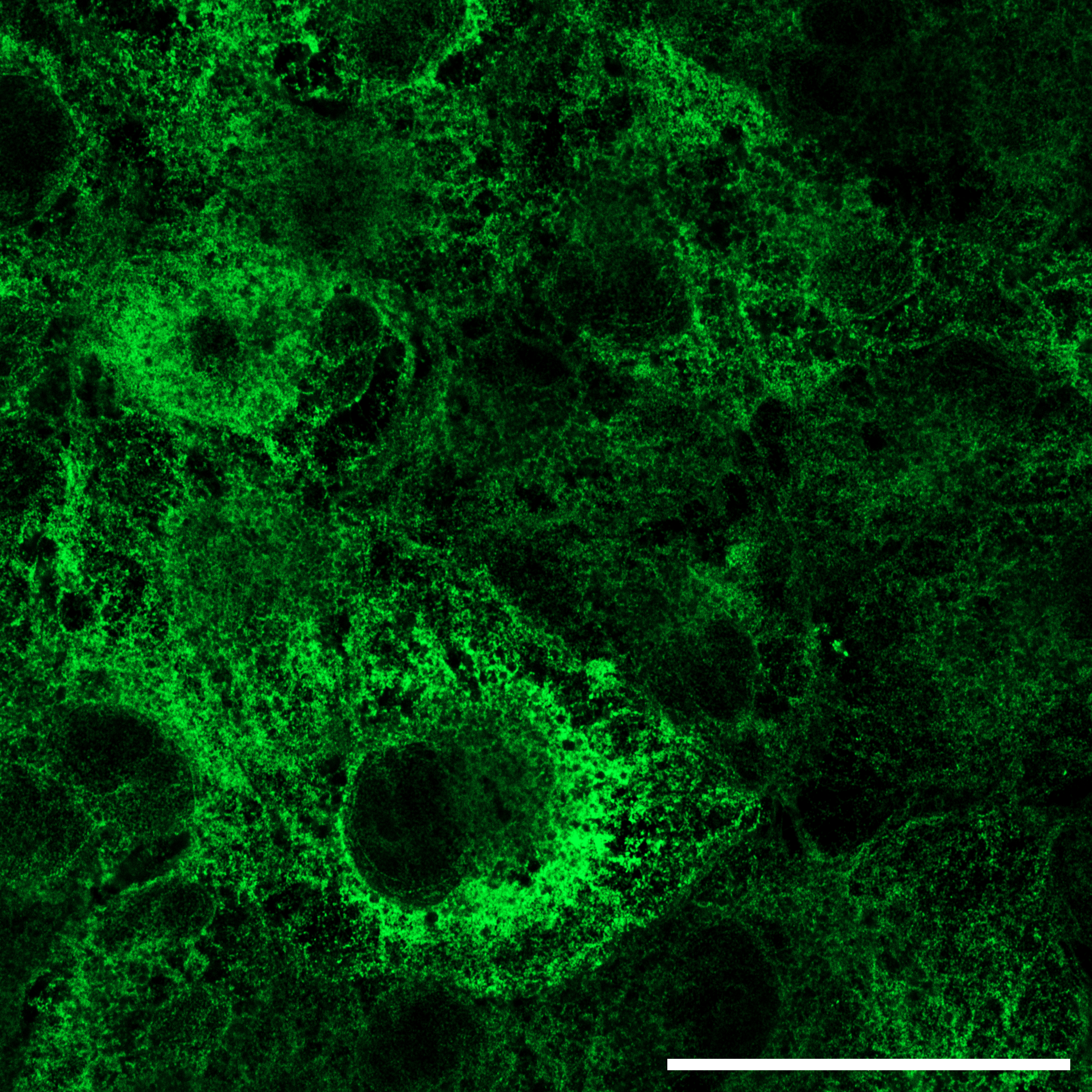

Supplement: Supplementary file 12 — Figure EV3 Source Data [file 44319_2026_736_MOESM12_ESM.zip › Figure EV3/EV3B/KO/NHE3-GFP HK-2 CTNS KO_GM130_NHE3 GFP.tif]

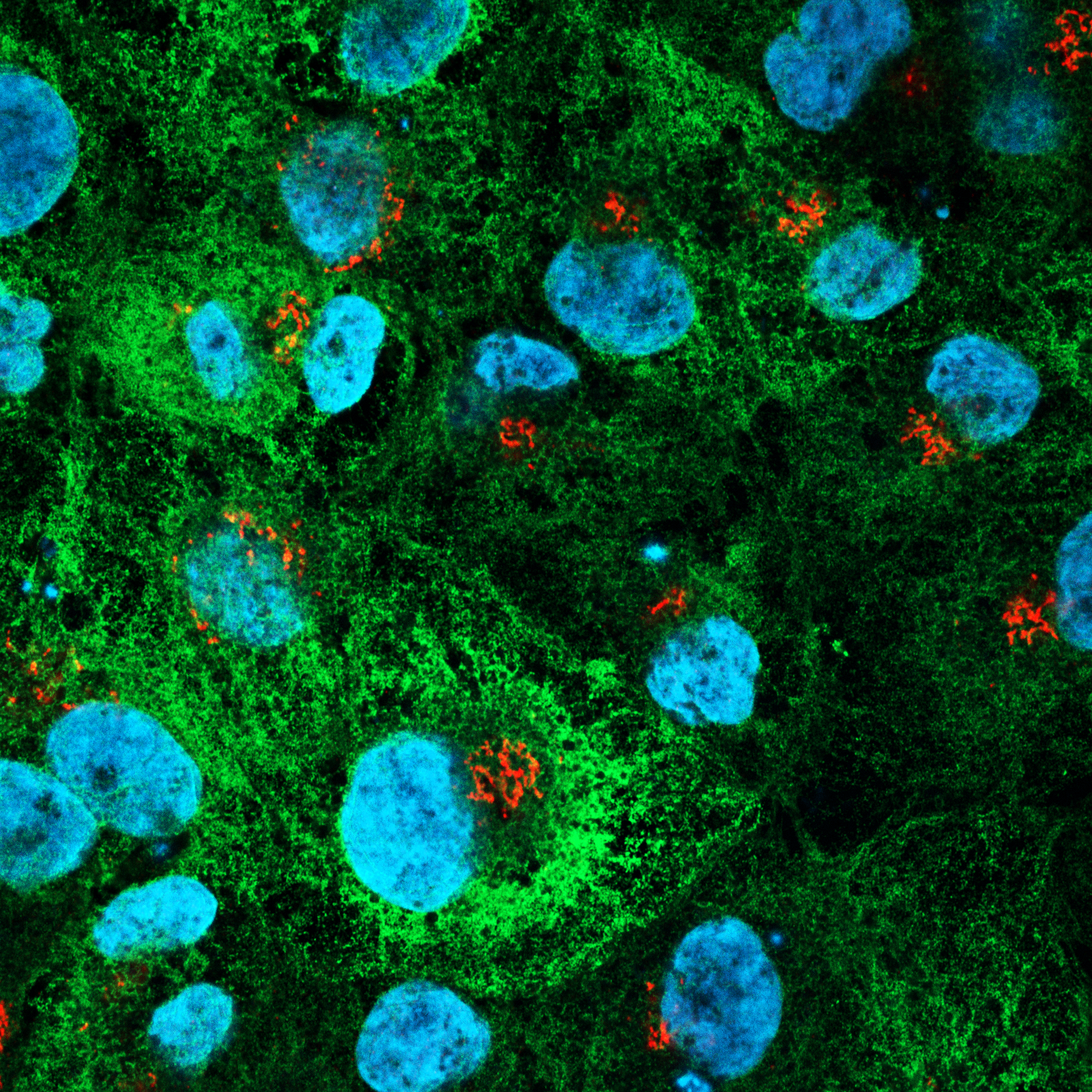

Supplement: Supplementary file 12 — Figure EV3 Source Data [file 44319_2026_736_MOESM12_ESM.zip › Figure EV3/EV3B/KO/NHE3-GFP HK-2 CTNS KO_GM130_Merged.tif]

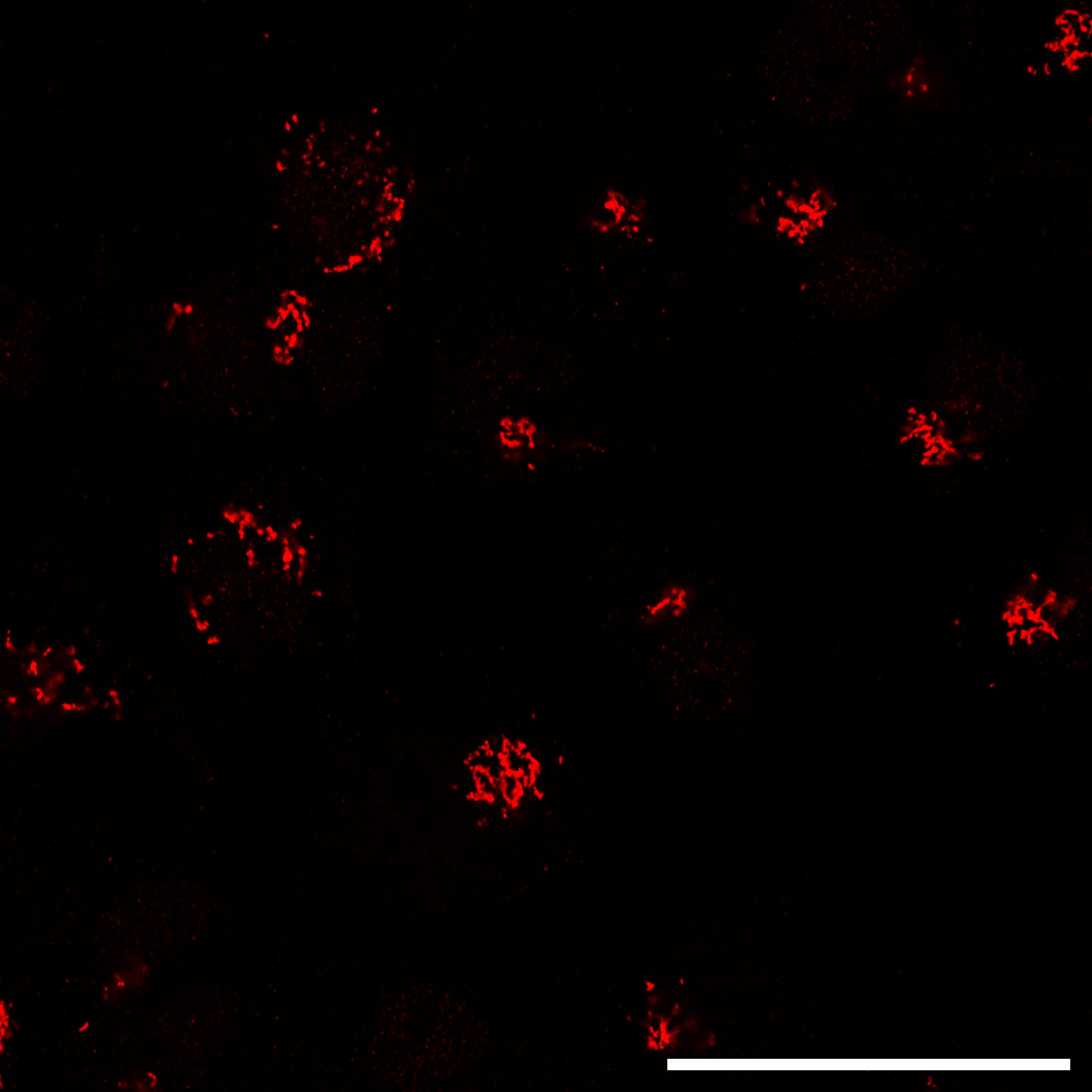

Supplement: Supplementary file 12 — Figure EV3 Source Data [file 44319_2026_736_MOESM12_ESM.zip › Figure EV3/EV3B/KO/NHE3-GFP HK-2 CTNS KO_GM130_GM130.tif]

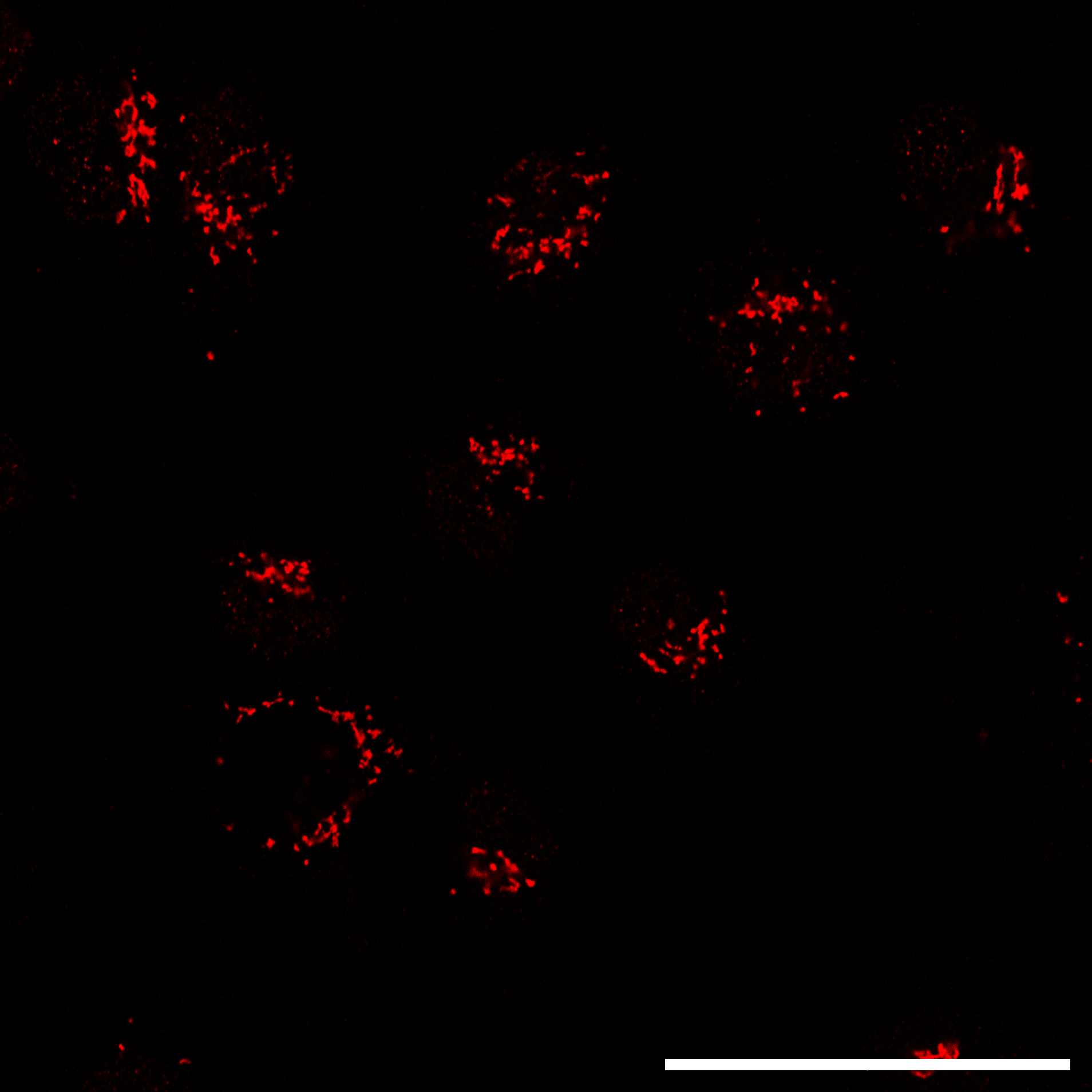

Supplement: Supplementary file 12 — Figure EV3 Source Data [file 44319_2026_736_MOESM12_ESM.zip › Figure EV3/EV3B/WT/NHE3-GFP HK-2 WT GM130_GM130.tif]

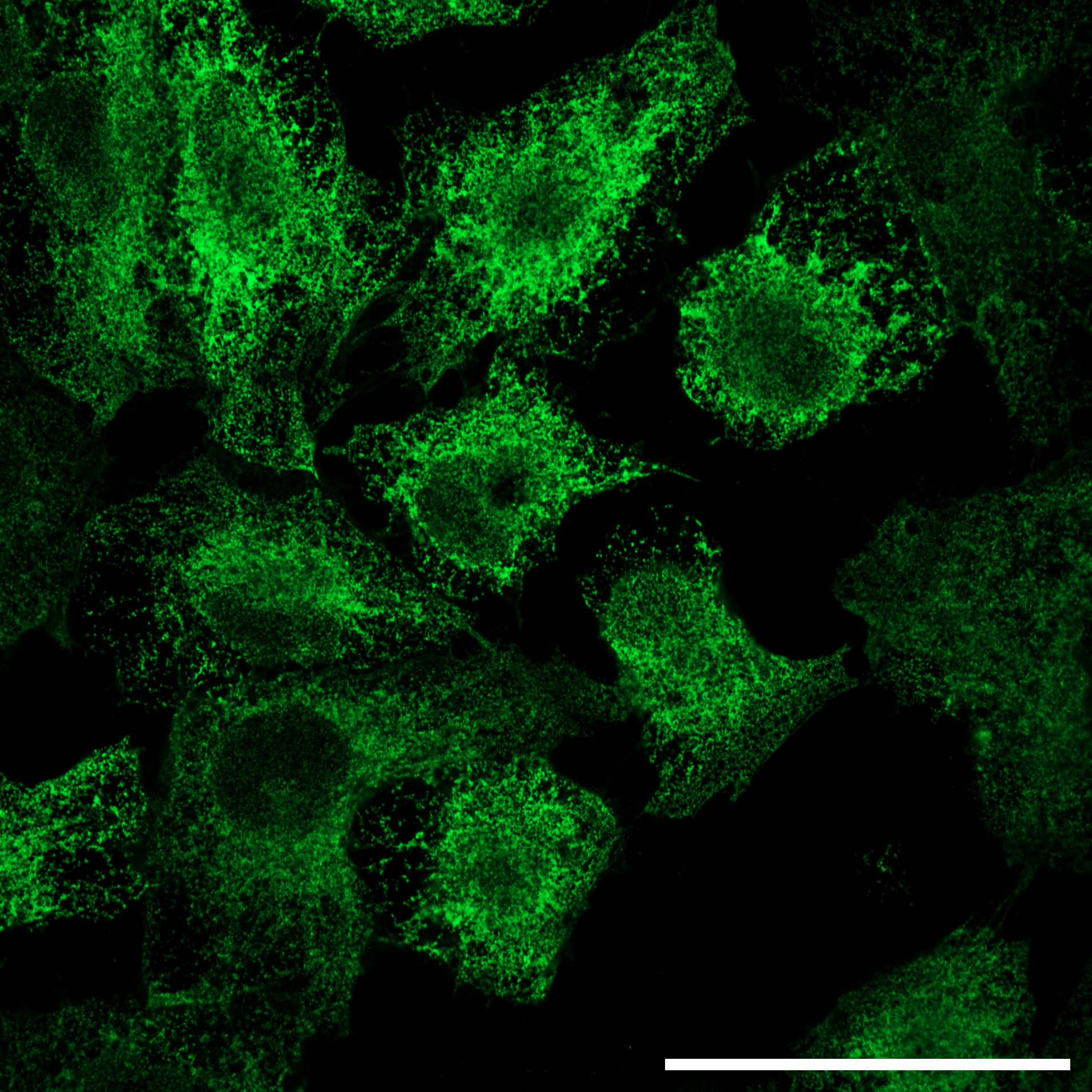

Supplement: Supplementary file 12 — Figure EV3 Source Data [file 44319_2026_736_MOESM12_ESM.zip › Figure EV3/EV3B/WT/NHE3-GFP HK-2 WT GM130_NHE3-GFP.tif]

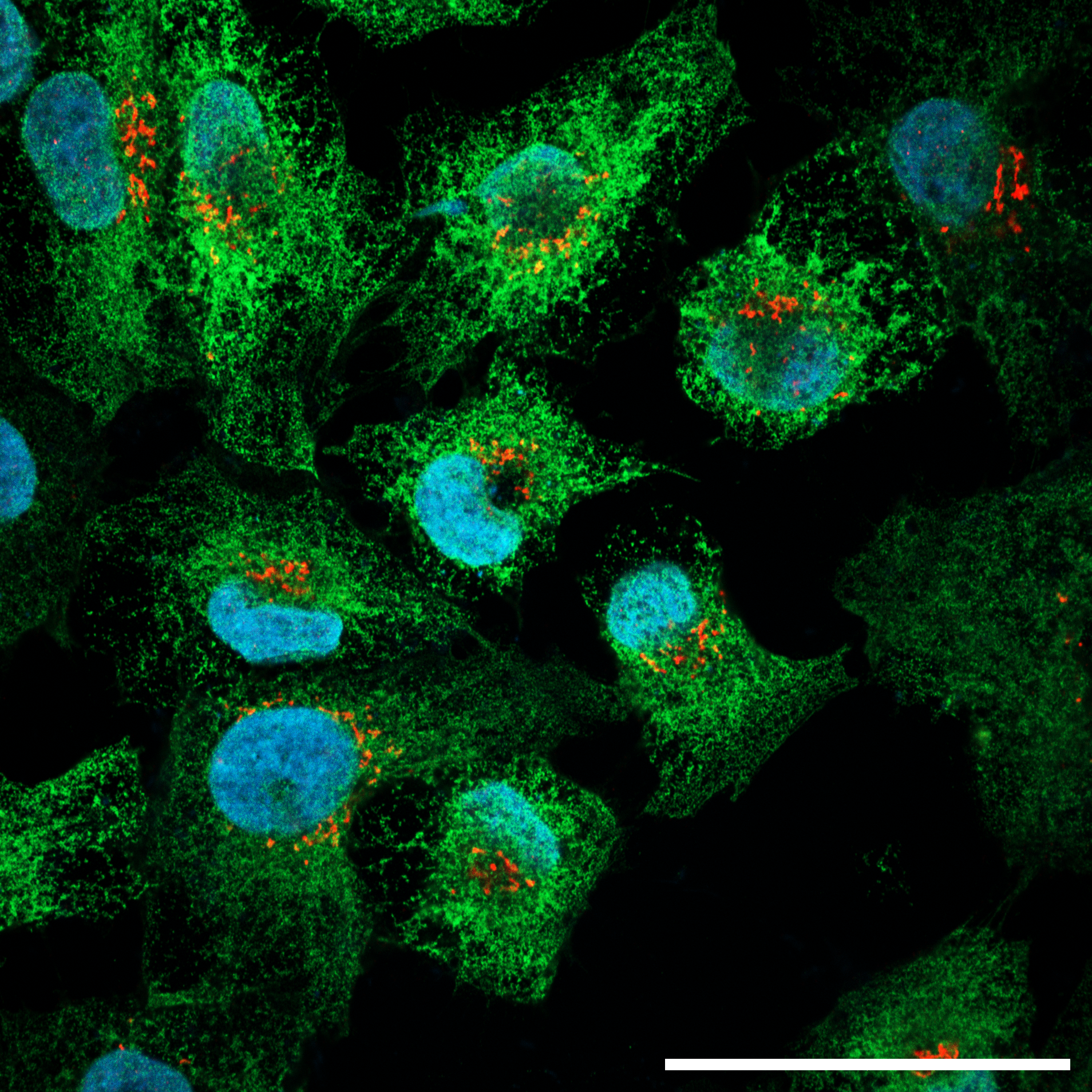

Supplement: Supplementary file 12 — Figure EV3 Source Data [file 44319_2026_736_MOESM12_ESM.zip › Figure EV3/EV3B/WT/NHE3-GFP HK-2 WT GM130_Merged.tif]

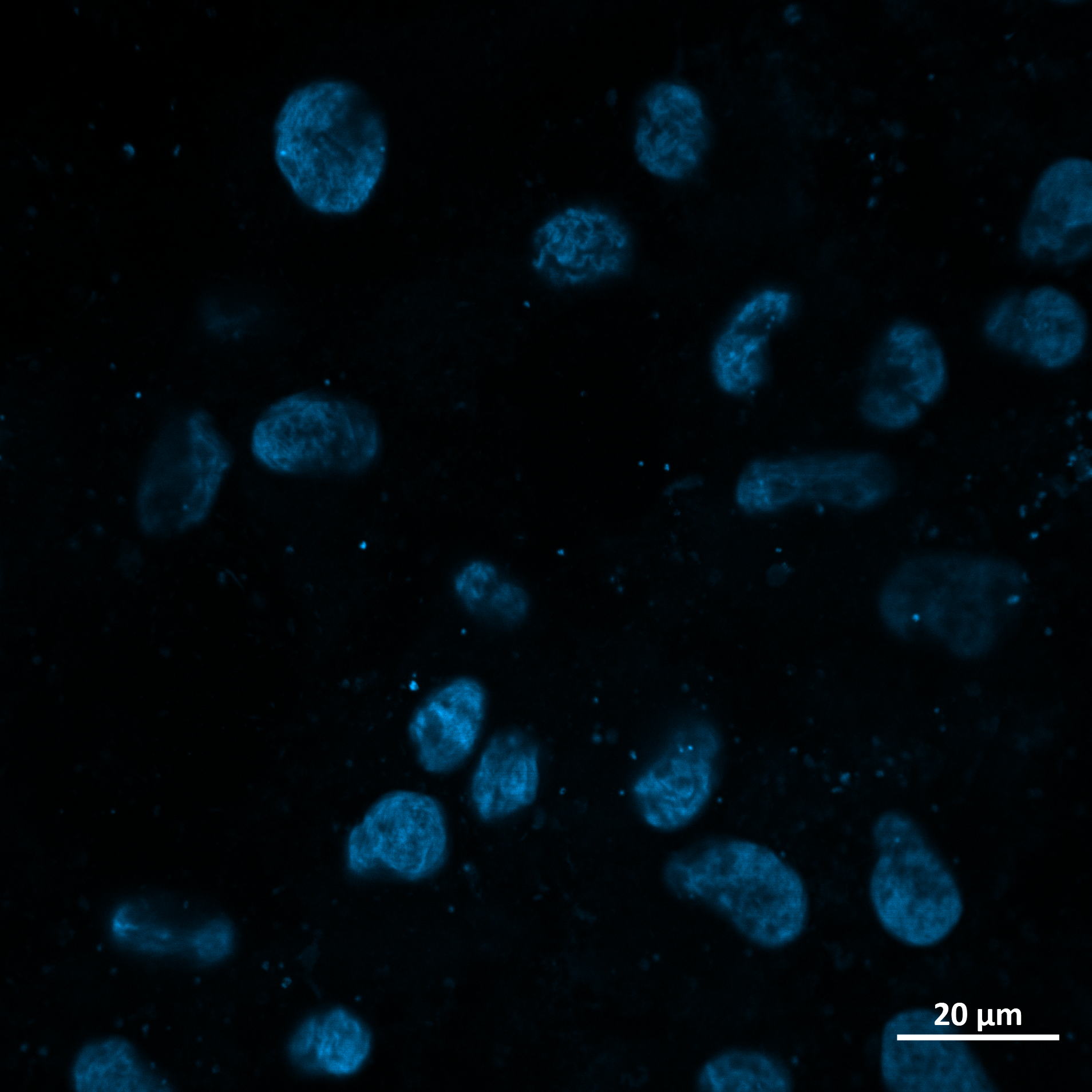

Supplement: Supplementary file 13 — Figure EV3 Replicate Source Data [file 44319_2026_736_MOESM13_ESM.zip › Figure EV3_Replicate/EV3C_Replicate/KO/NHE3-GFP HK-2 CTNS KO LAMP1_DAPI.tif]

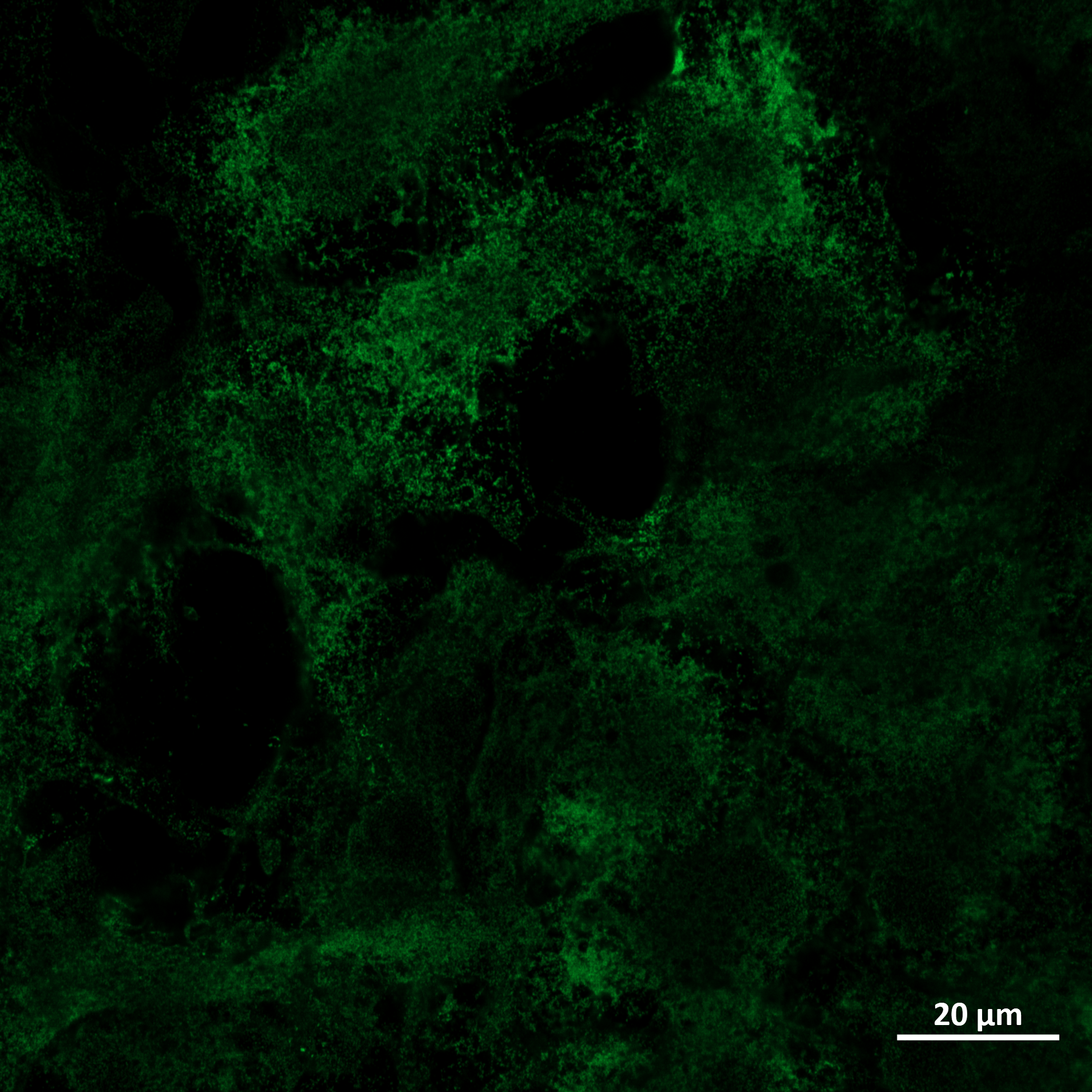

Supplement: Supplementary file 13 — Figure EV3 Replicate Source Data [file 44319_2026_736_MOESM13_ESM.zip › Figure EV3_Replicate/EV3C_Replicate/KO/NHE3-GFP HK-2 CTNS KO LAMP1_NHE3 GFP.tif]

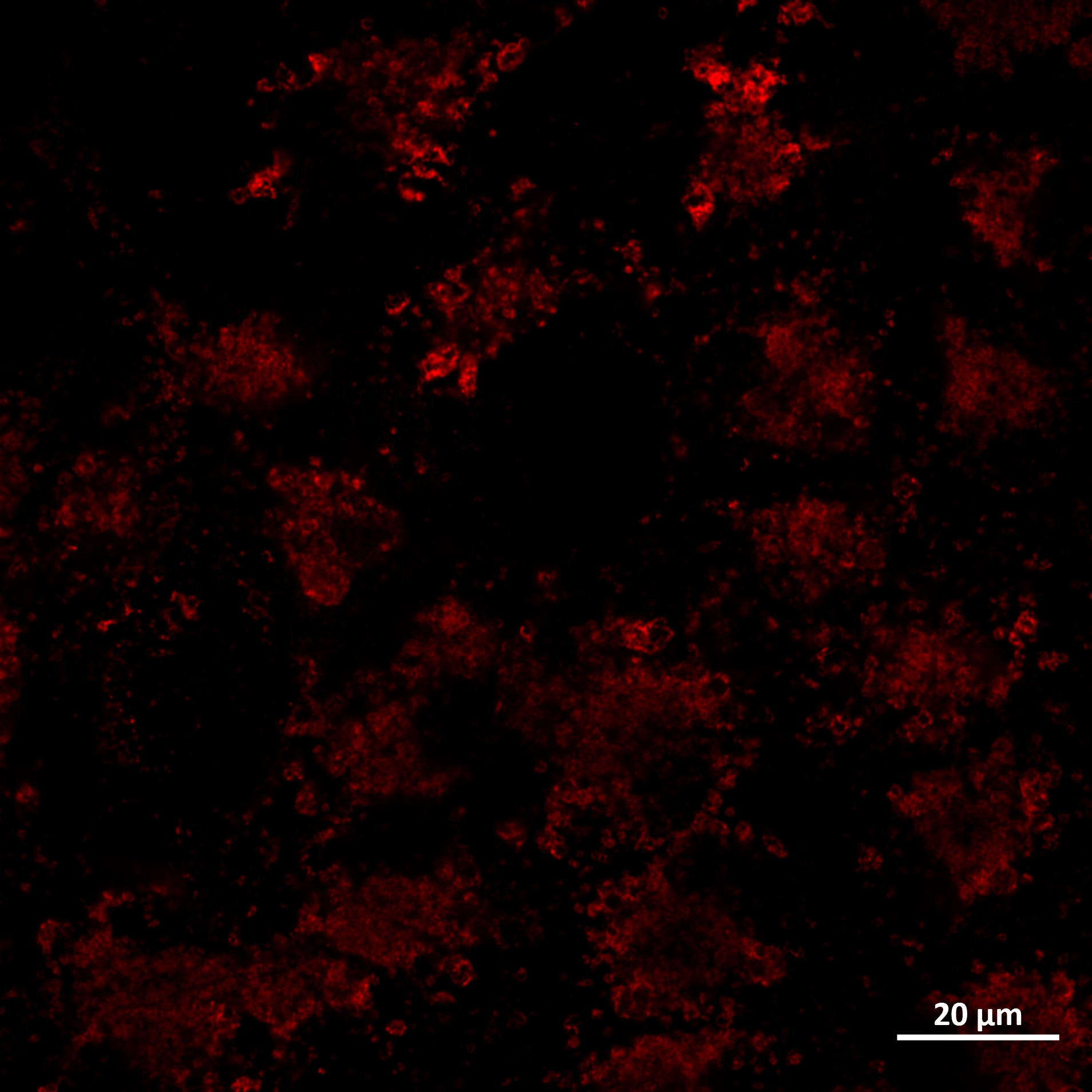

Supplement: Supplementary file 13 — Figure EV3 Replicate Source Data [file 44319_2026_736_MOESM13_ESM.zip › Figure EV3_Replicate/EV3C_Replicate/KO/NHE3-GFP HK-2 CTNS KO LAMP1_LAPM1.tif]

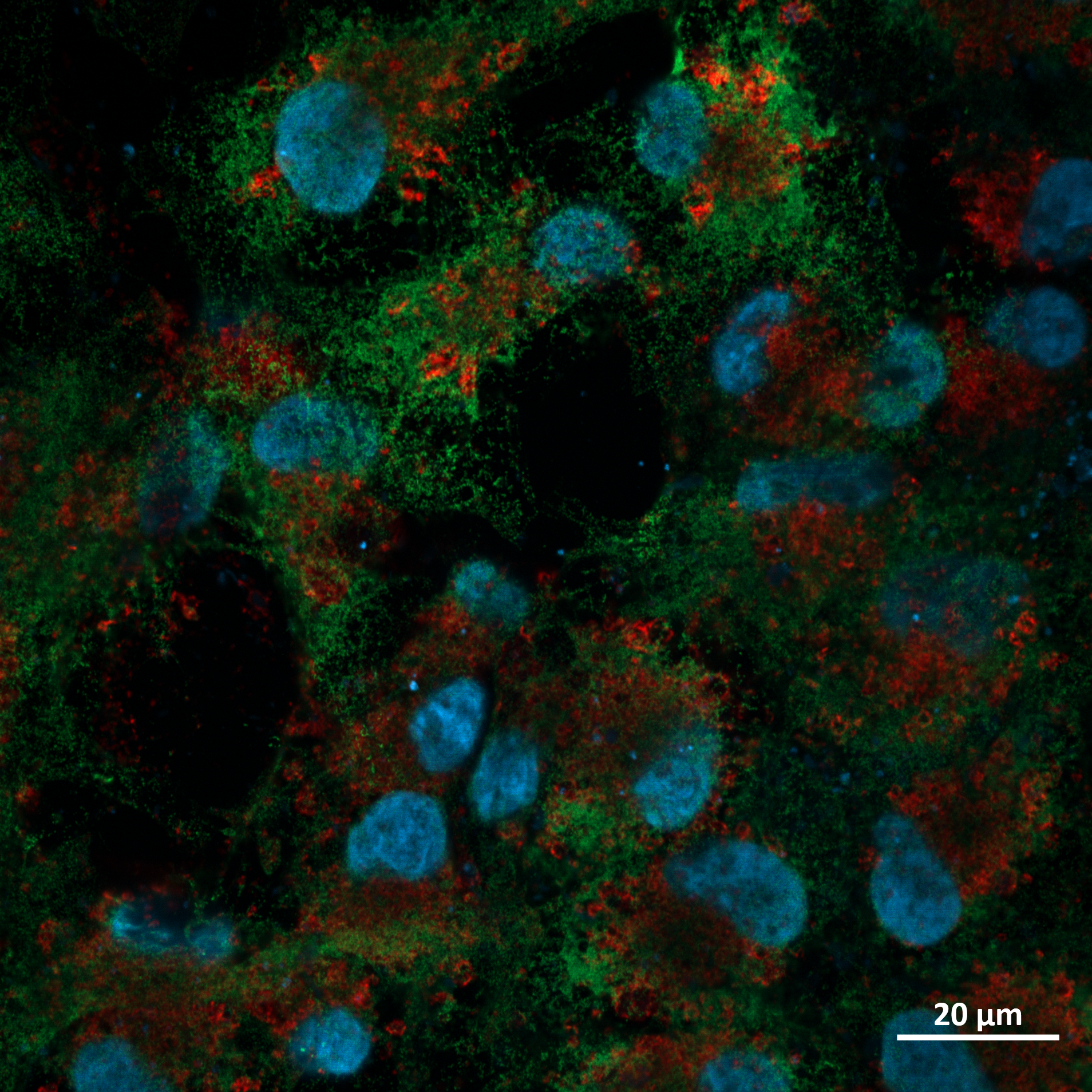

Supplement: Supplementary file 13 — Figure EV3 Replicate Source Data [file 44319_2026_736_MOESM13_ESM.zip › Figure EV3_Replicate/EV3C_Replicate/KO/NHE3-GFP HK-2 CTNS LAMP1_Merged.tif]

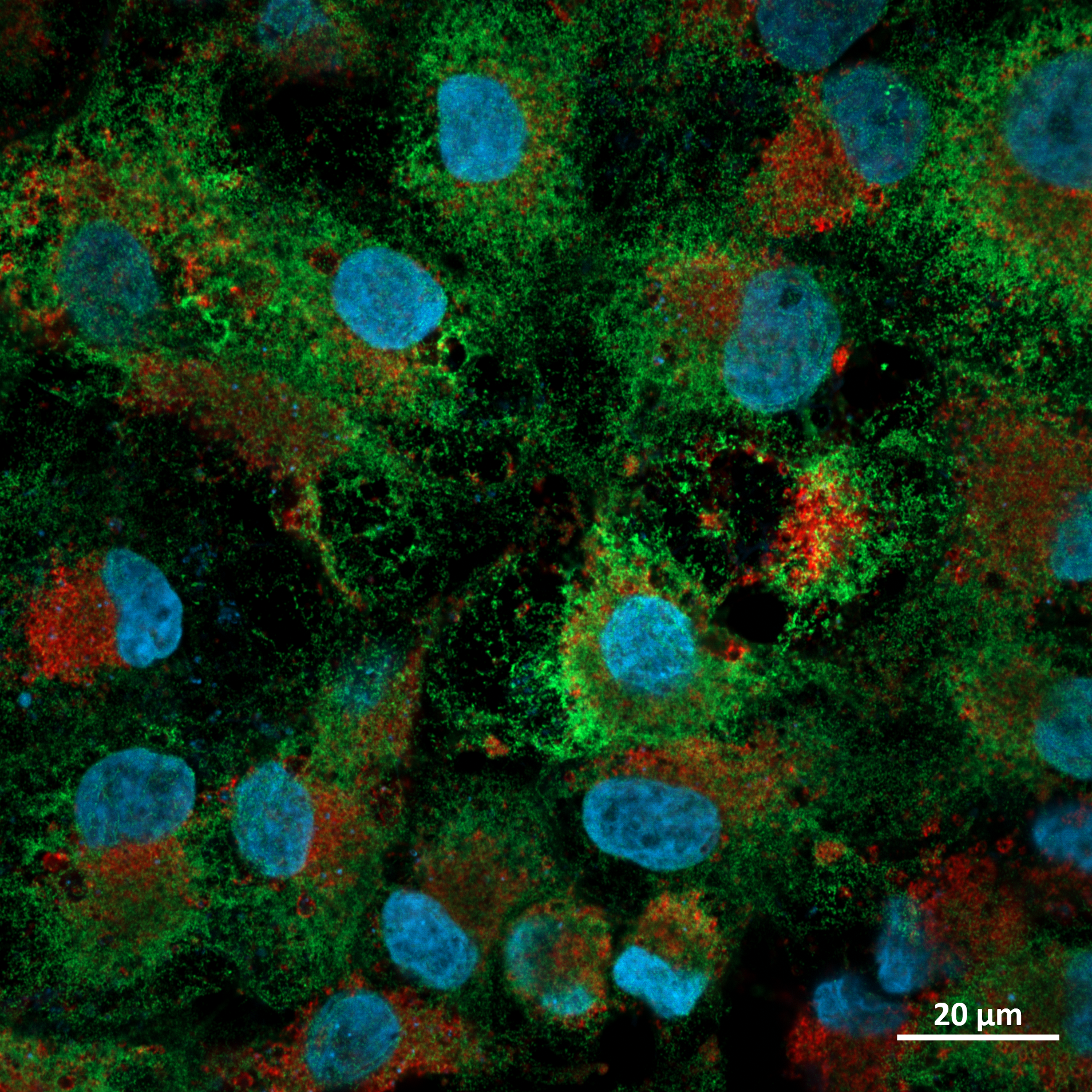

Supplement: Supplementary file 13 — Figure EV3 Replicate Source Data [file 44319_2026_736_MOESM13_ESM.zip › Figure EV3_Replicate/EV3C_Replicate/WT/NHE3-GFP HK-2 WT LAMP1_Merged.tif]

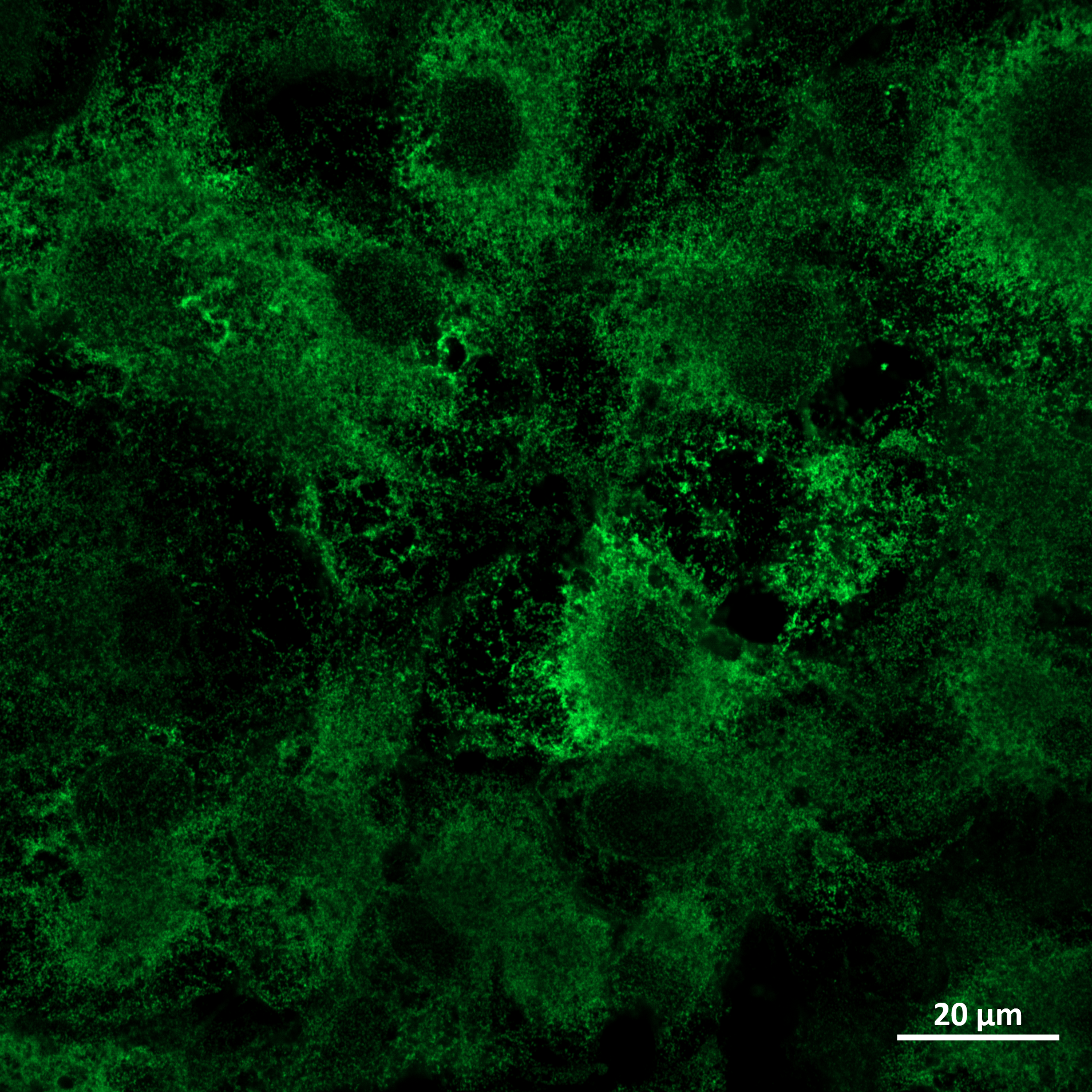

Supplement: Supplementary file 13 — Figure EV3 Replicate Source Data [file 44319_2026_736_MOESM13_ESM.zip › Figure EV3_Replicate/EV3C_Replicate/WT/NHE3-GFP HK-2 WT LAMP1_NHE3-GFP.tif]

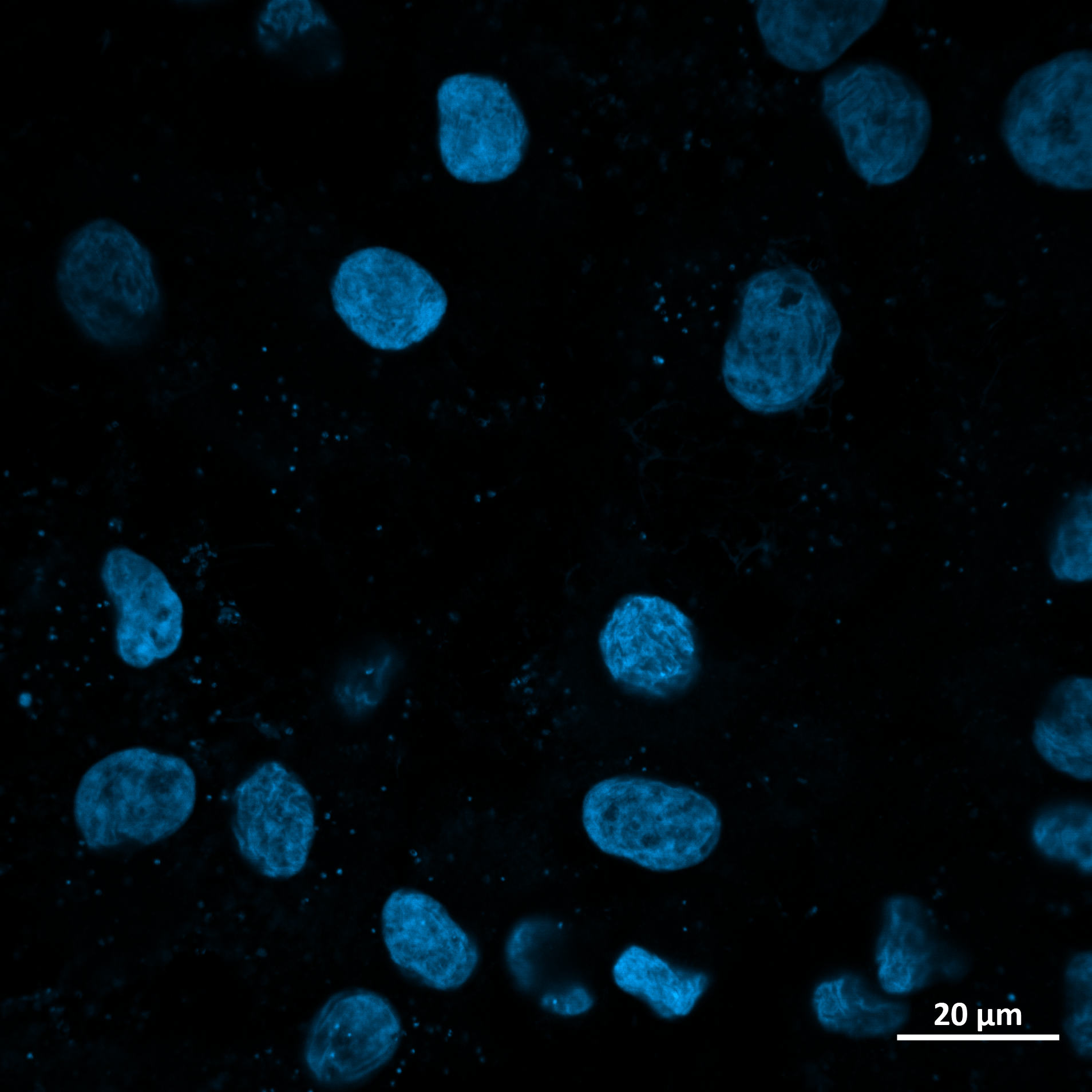

Supplement: Supplementary file 13 — Figure EV3 Replicate Source Data [file 44319_2026_736_MOESM13_ESM.zip › Figure EV3_Replicate/EV3C_Replicate/WT/NHE3-GFP HK-2 WT LAMP1_DAPI.tif]

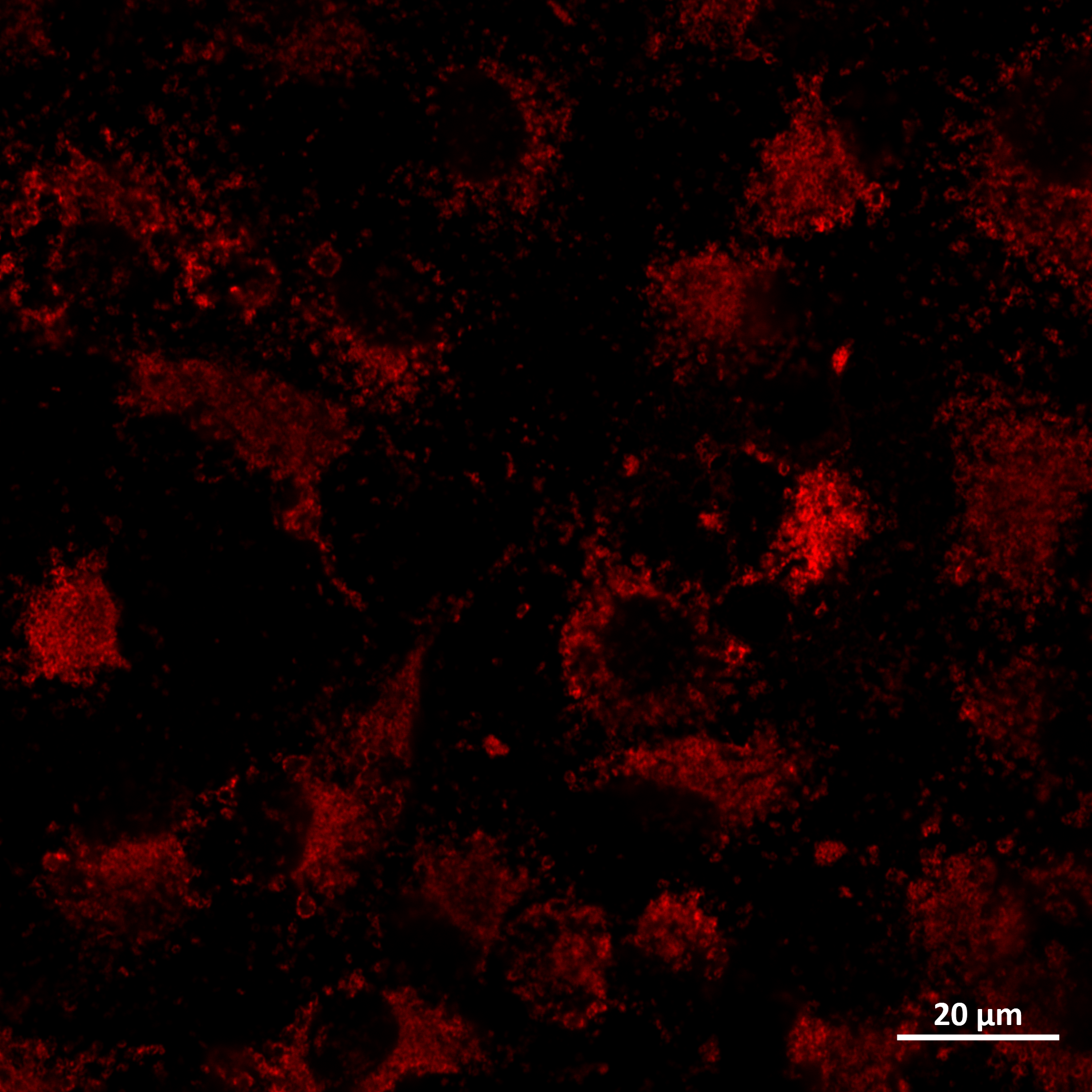

Supplement: Supplementary file 13 — Figure EV3 Replicate Source Data [file 44319_2026_736_MOESM13_ESM.zip › Figure EV3_Replicate/EV3C_Replicate/WT/NHE3-GFP HK-2 WT LAMP1_LAMP1.tif]

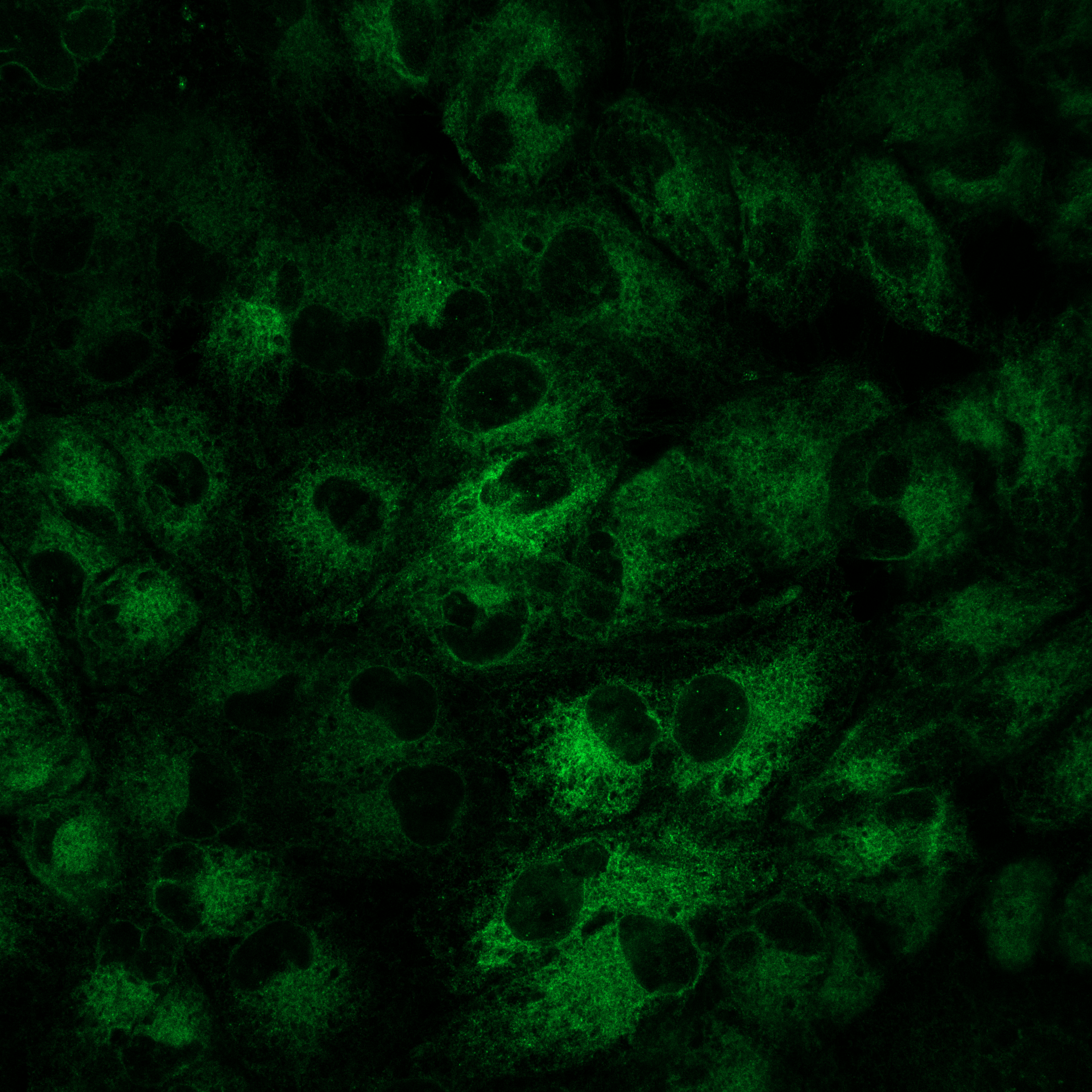

Supplement: Supplementary file 13 — Figure EV3 Replicate Source Data [file 44319_2026_736_MOESM13_ESM.zip › Figure EV3_Replicate/EV3D_Replicate/KO/KO cysteamine_NHE3.tif]

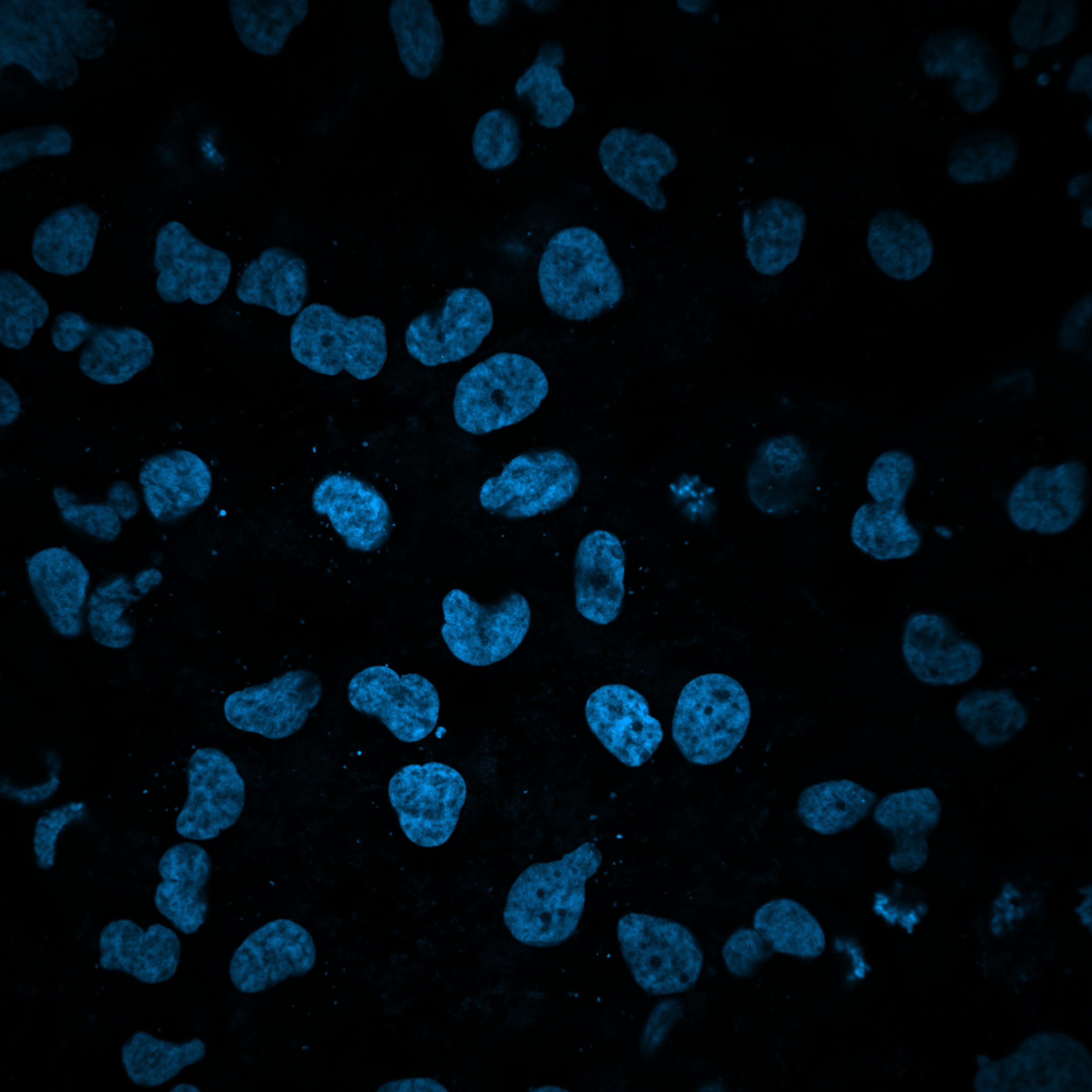

Supplement: Supplementary file 13 — Figure EV3 Replicate Source Data [file 44319_2026_736_MOESM13_ESM.zip › Figure EV3_Replicate/EV3D_Replicate/KO/KO cysteamine_DAPI.tif]

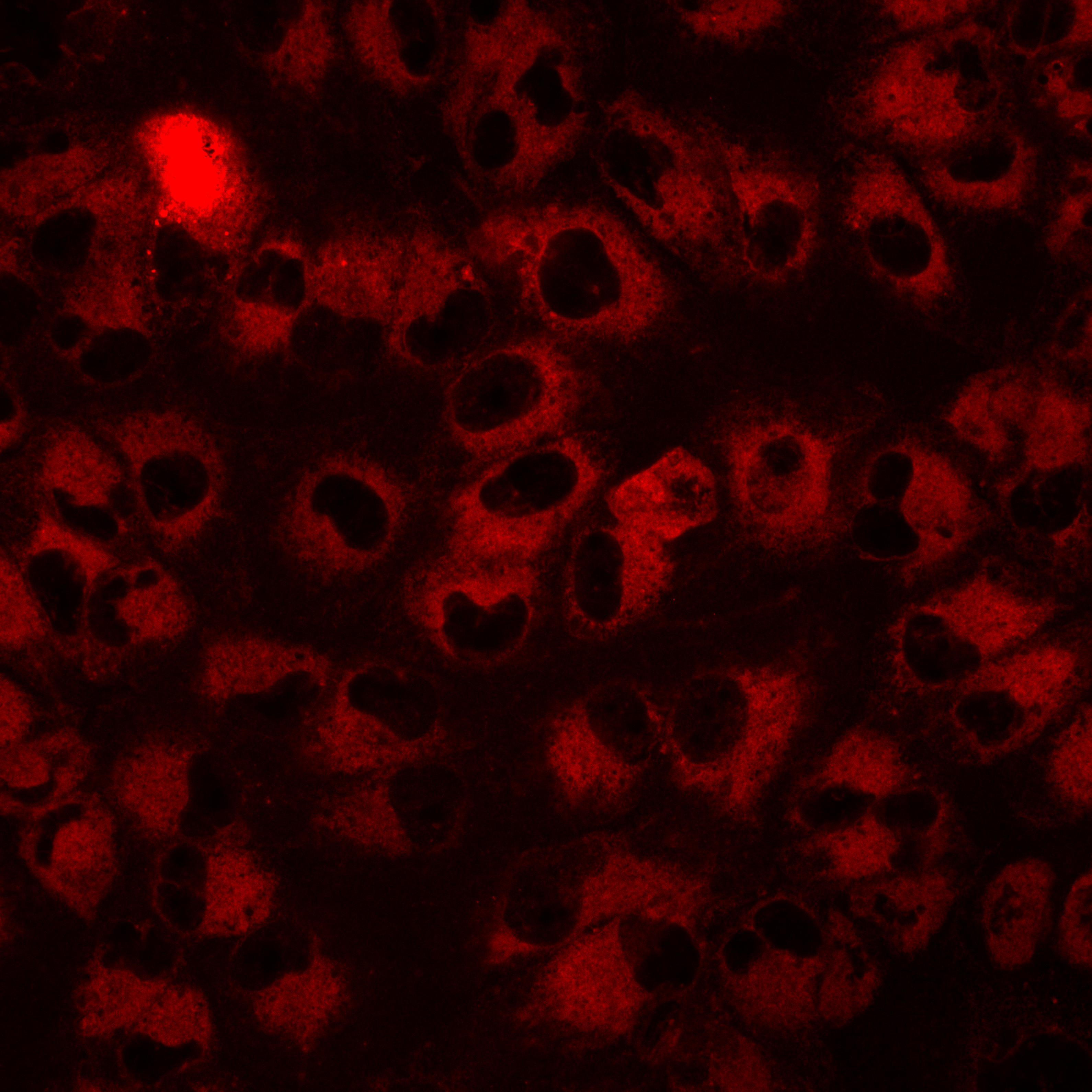

Supplement: Supplementary file 13 — Figure EV3 Replicate Source Data [file 44319_2026_736_MOESM13_ESM.zip › Figure EV3_Replicate/EV3D_Replicate/KO/KO cysteamine_ER Tracker.tif]

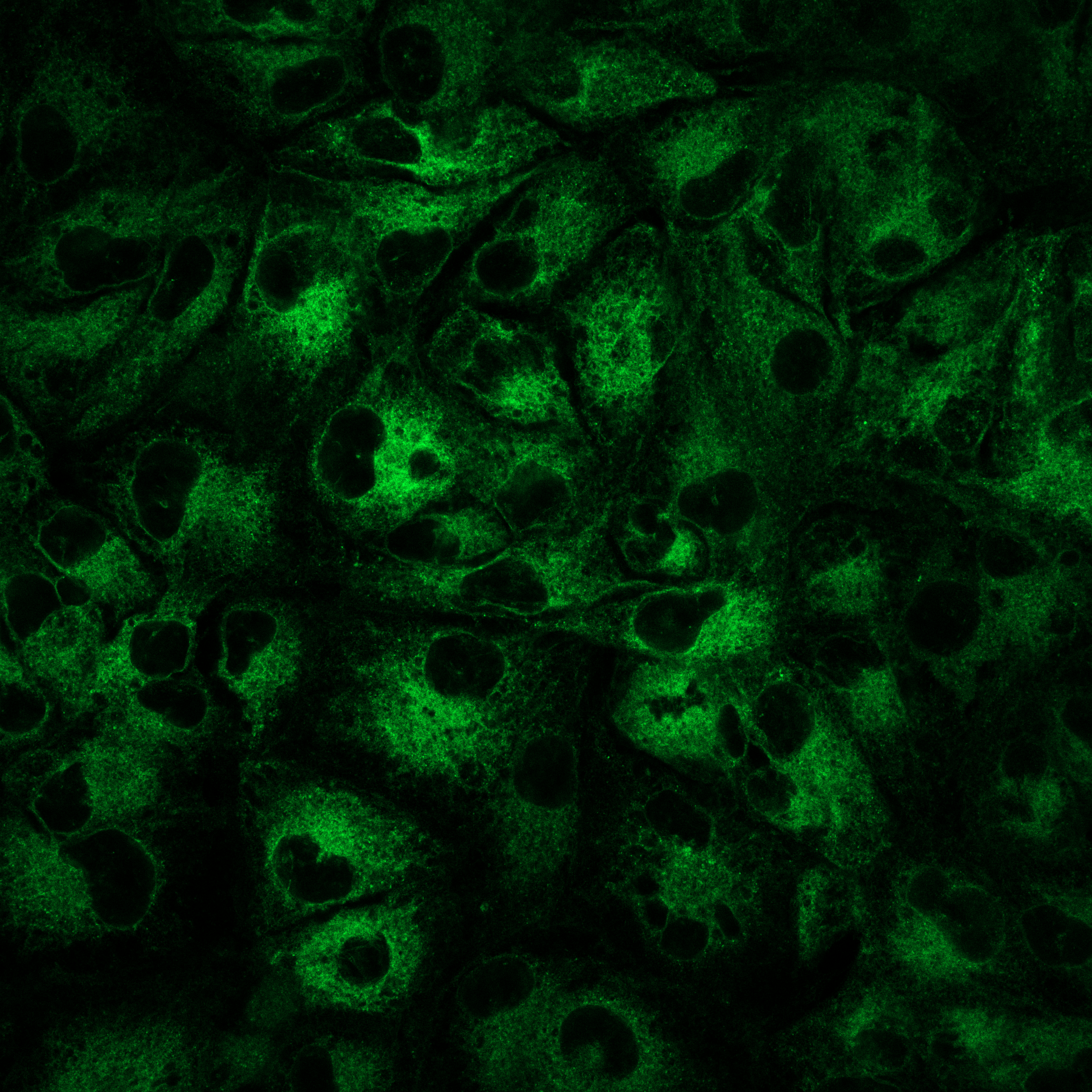

Supplement: Supplementary file 13 — Figure EV3 Replicate Source Data [file 44319_2026_736_MOESM13_ESM.zip › Figure EV3_Replicate/EV3D_Replicate/KO/KO control_NHE3.tif]

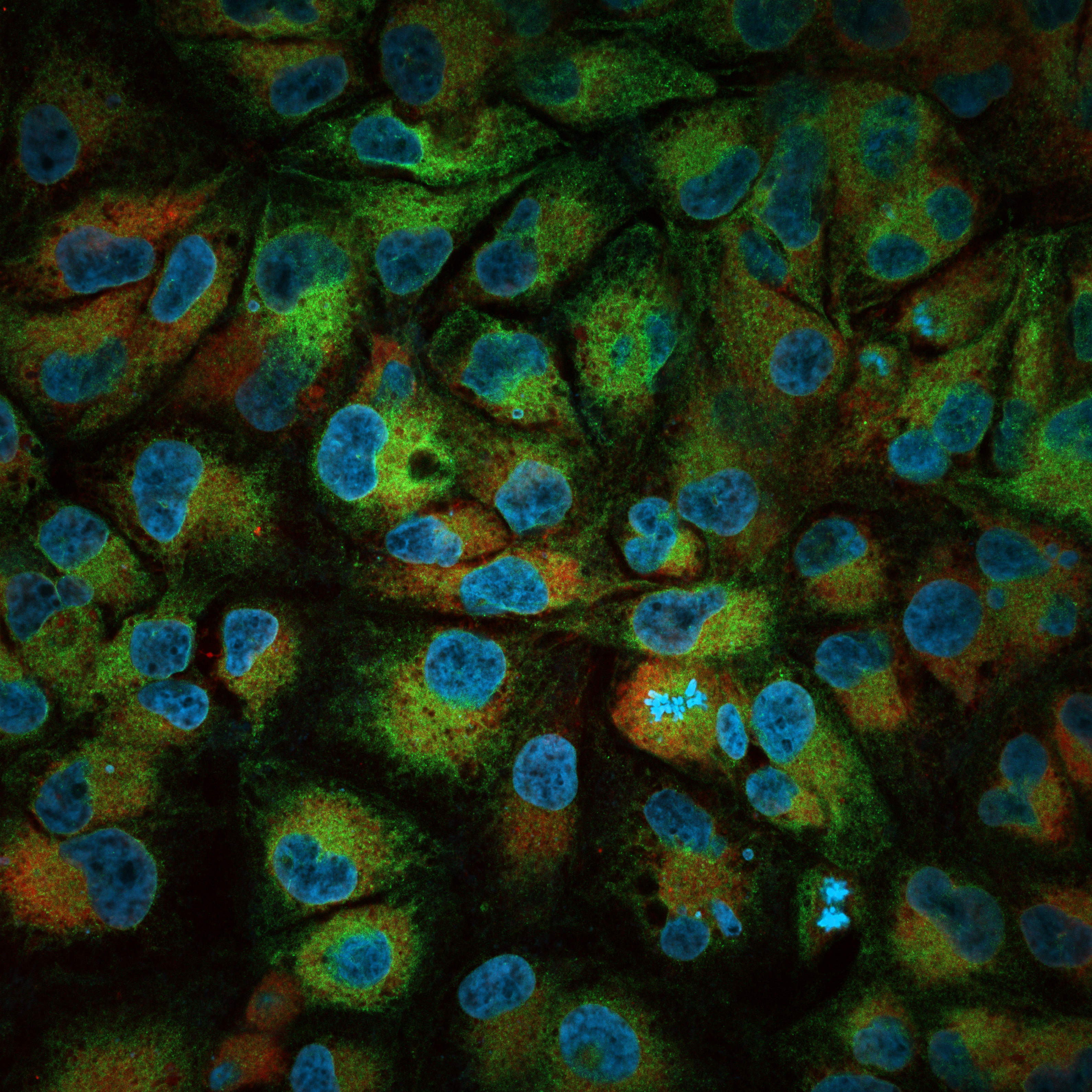

Supplement: Supplementary file 13 — Figure EV3 Replicate Source Data [file 44319_2026_736_MOESM13_ESM.zip › Figure EV3_Replicate/EV3D_Replicate/KO/KO control_Merged.tif]

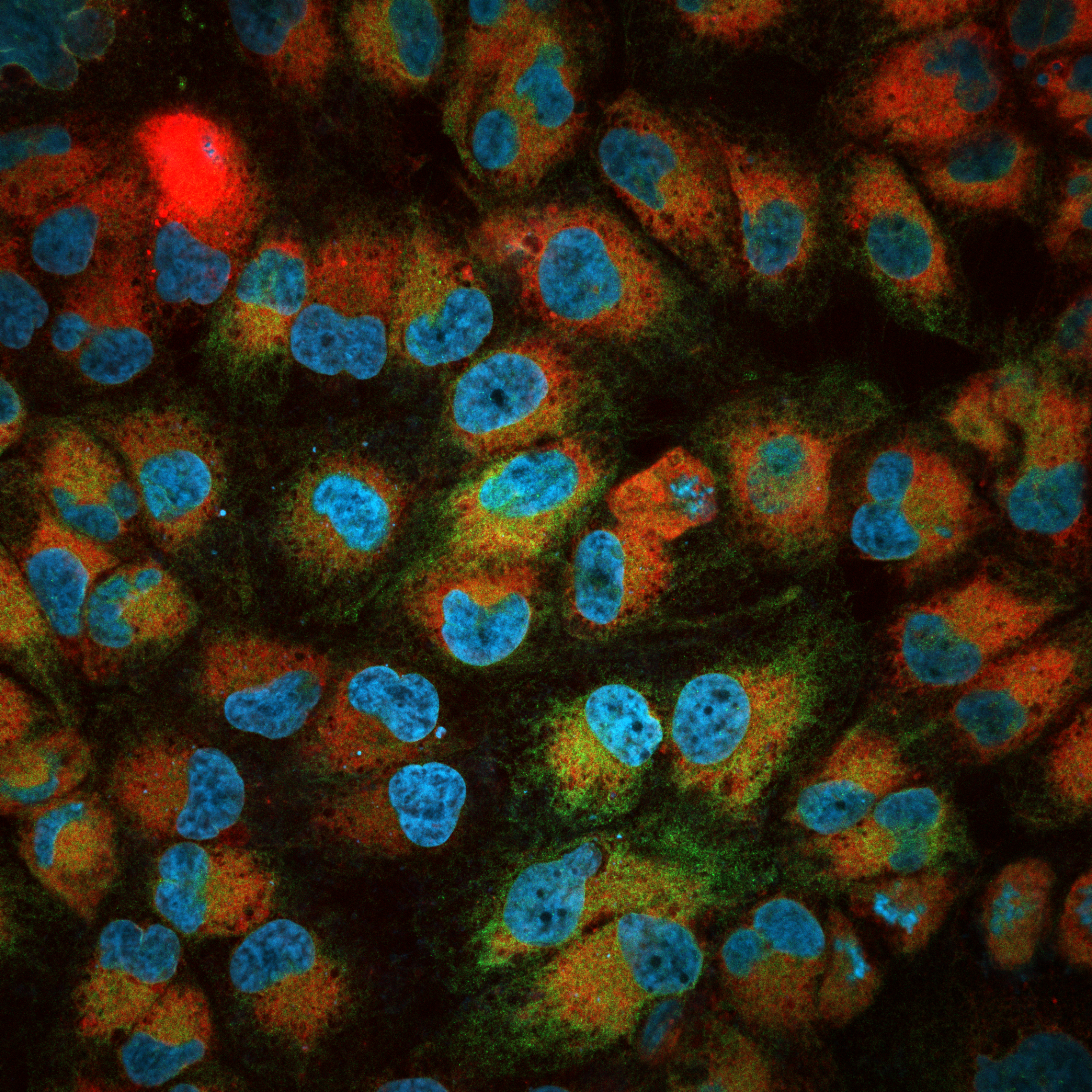

Supplement: Supplementary file 13 — Figure EV3 Replicate Source Data [file 44319_2026_736_MOESM13_ESM.zip › Figure EV3_Replicate/EV3D_Replicate/KO/KO cysteamine_Merged.tif]

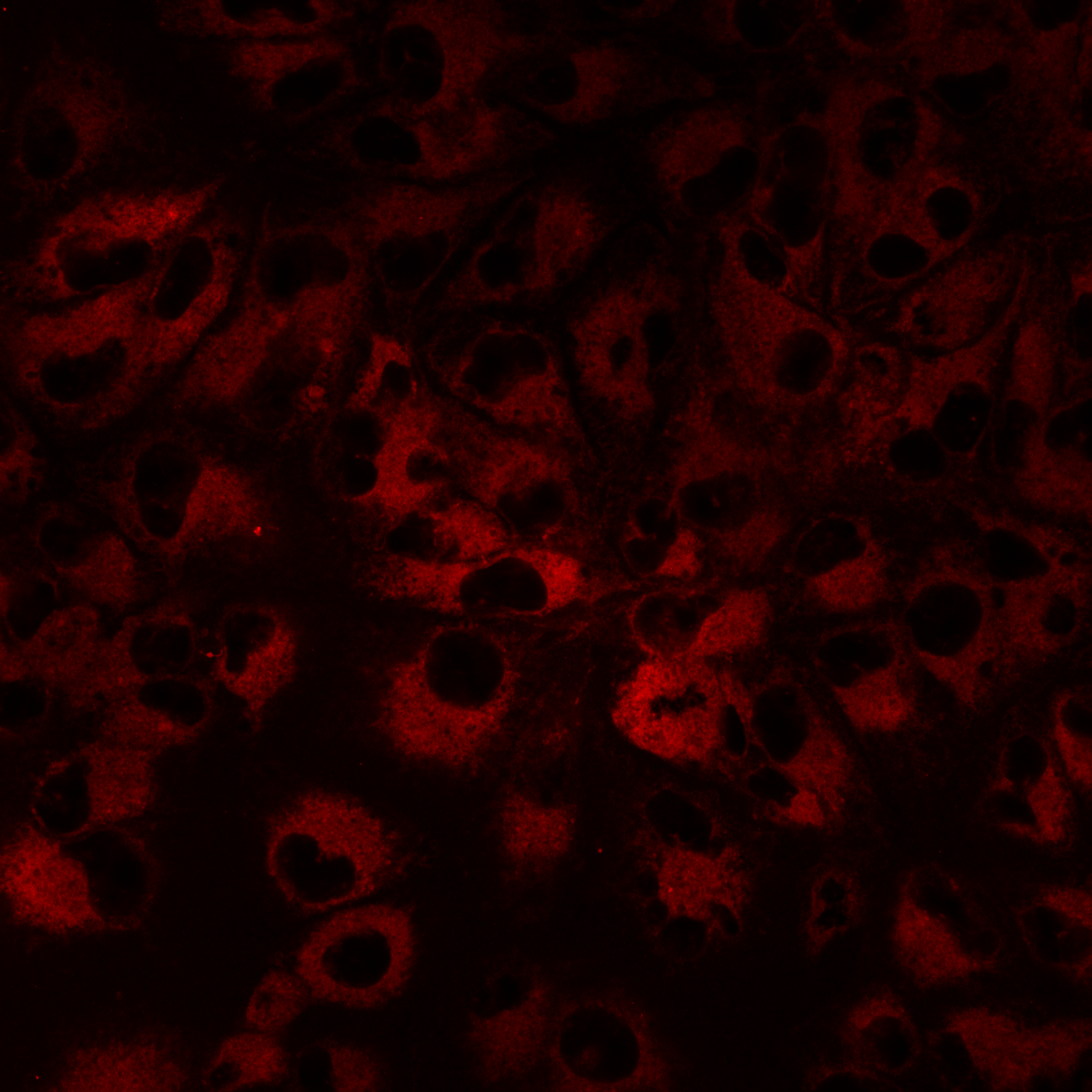

Supplement: Supplementary file 13 — Figure EV3 Replicate Source Data [file 44319_2026_736_MOESM13_ESM.zip › Figure EV3_Replicate/EV3D_Replicate/KO/KO control_ER Tracker.tif]

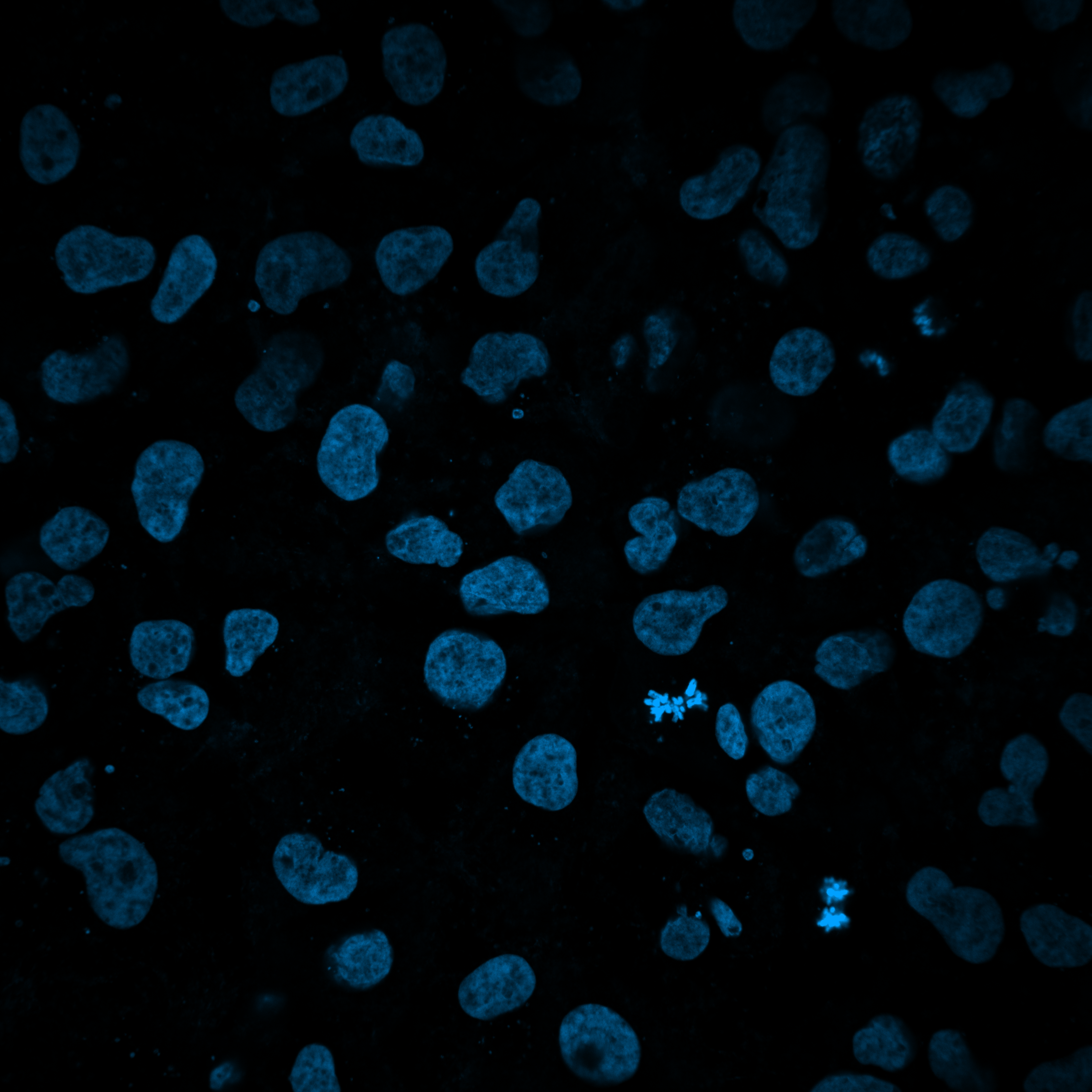

Supplement: Supplementary file 13 — Figure EV3 Replicate Source Data [file 44319_2026_736_MOESM13_ESM.zip › Figure EV3_Replicate/EV3D_Replicate/KO/KO control_DAPI.tif]

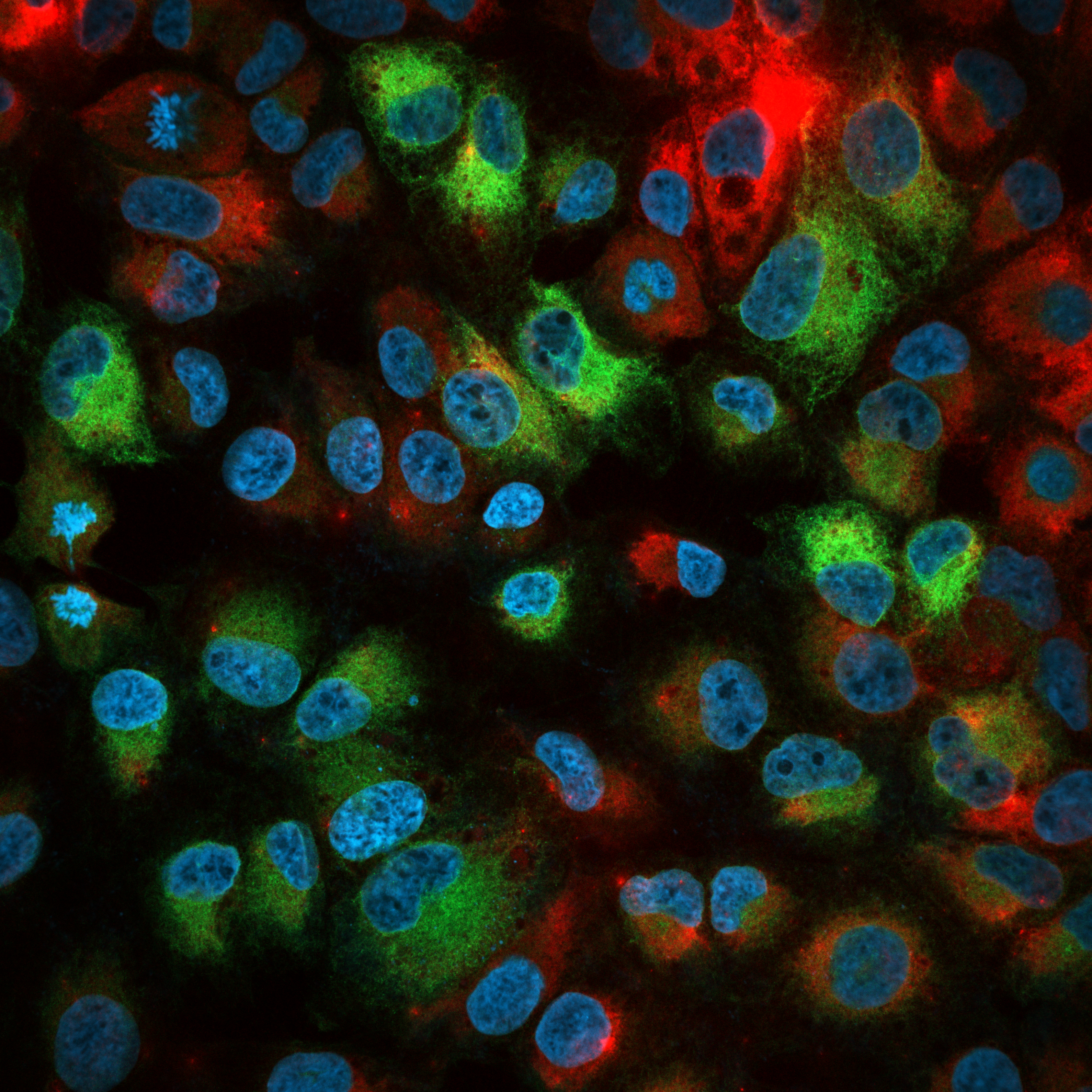

Supplement: Supplementary file 13 — Figure EV3 Replicate Source Data [file 44319_2026_736_MOESM13_ESM.zip › Figure EV3_Replicate/EV3D_Replicate/WT/WT cysteamine_Merged.tif]

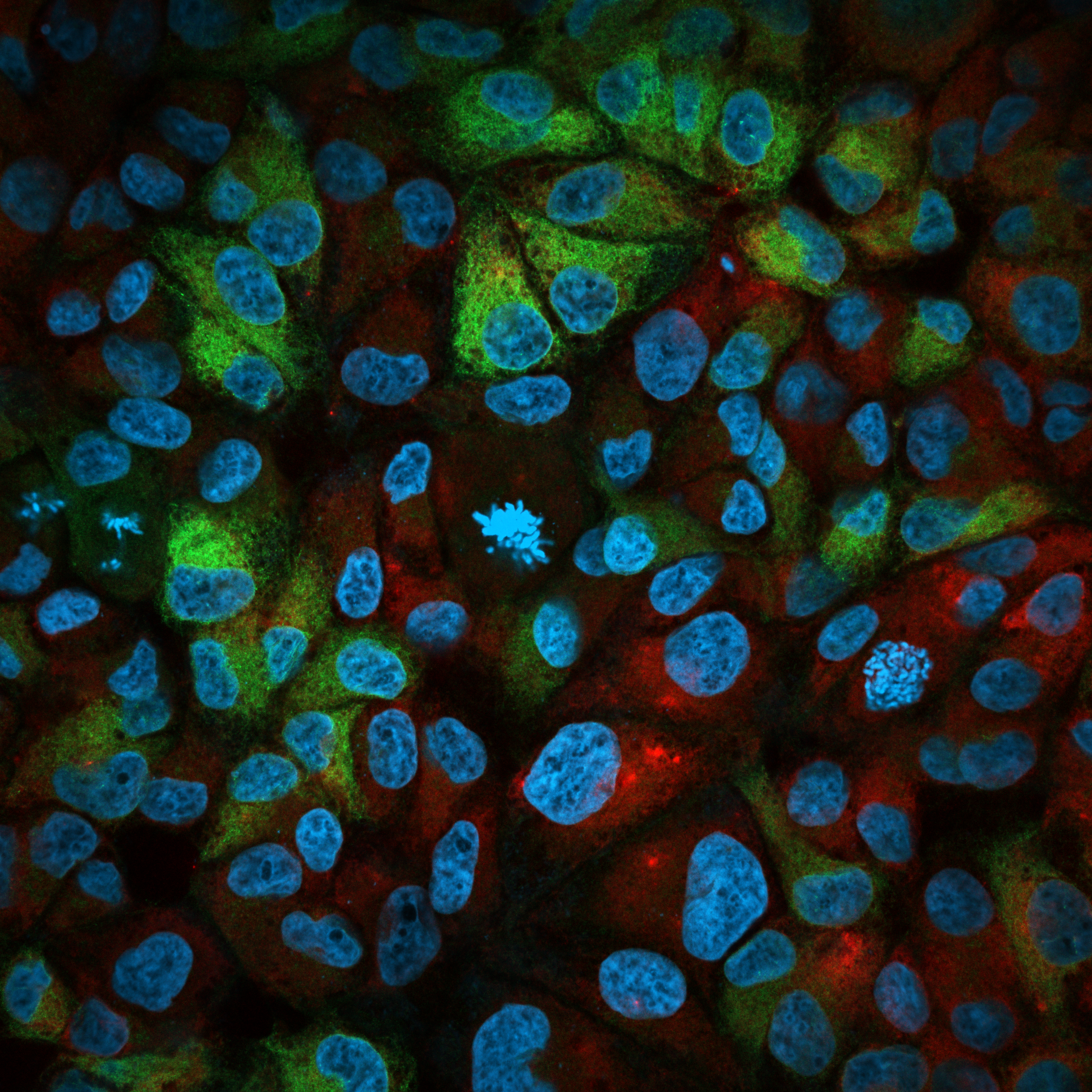

Supplement: Supplementary file 13 — Figure EV3 Replicate Source Data [file 44319_2026_736_MOESM13_ESM.zip › Figure EV3_Replicate/EV3D_Replicate/WT/WT control_Merged.tif]

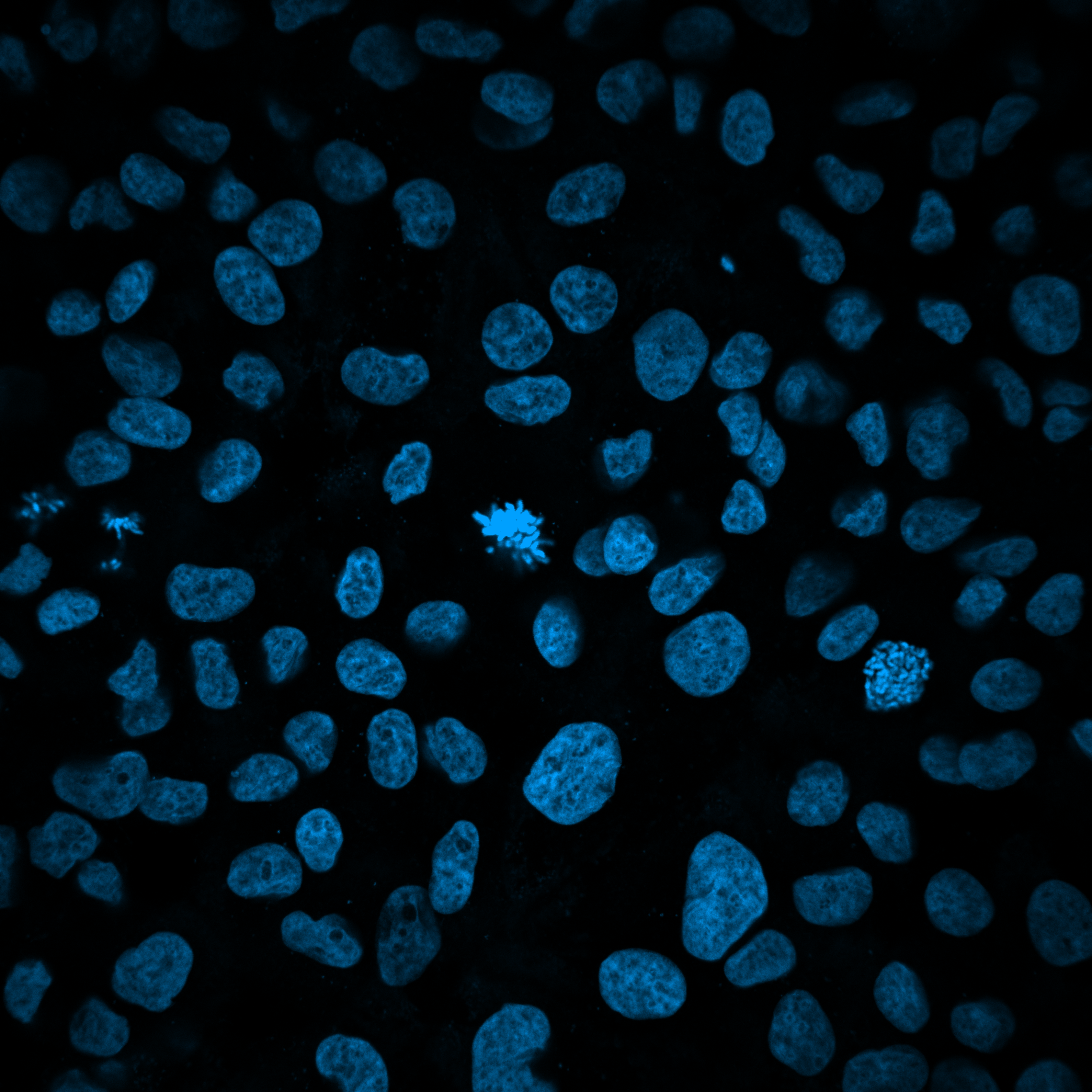

Supplement: Supplementary file 13 — Figure EV3 Replicate Source Data [file 44319_2026_736_MOESM13_ESM.zip › Figure EV3_Replicate/EV3D_Replicate/WT/WT control_DAPI.tif]

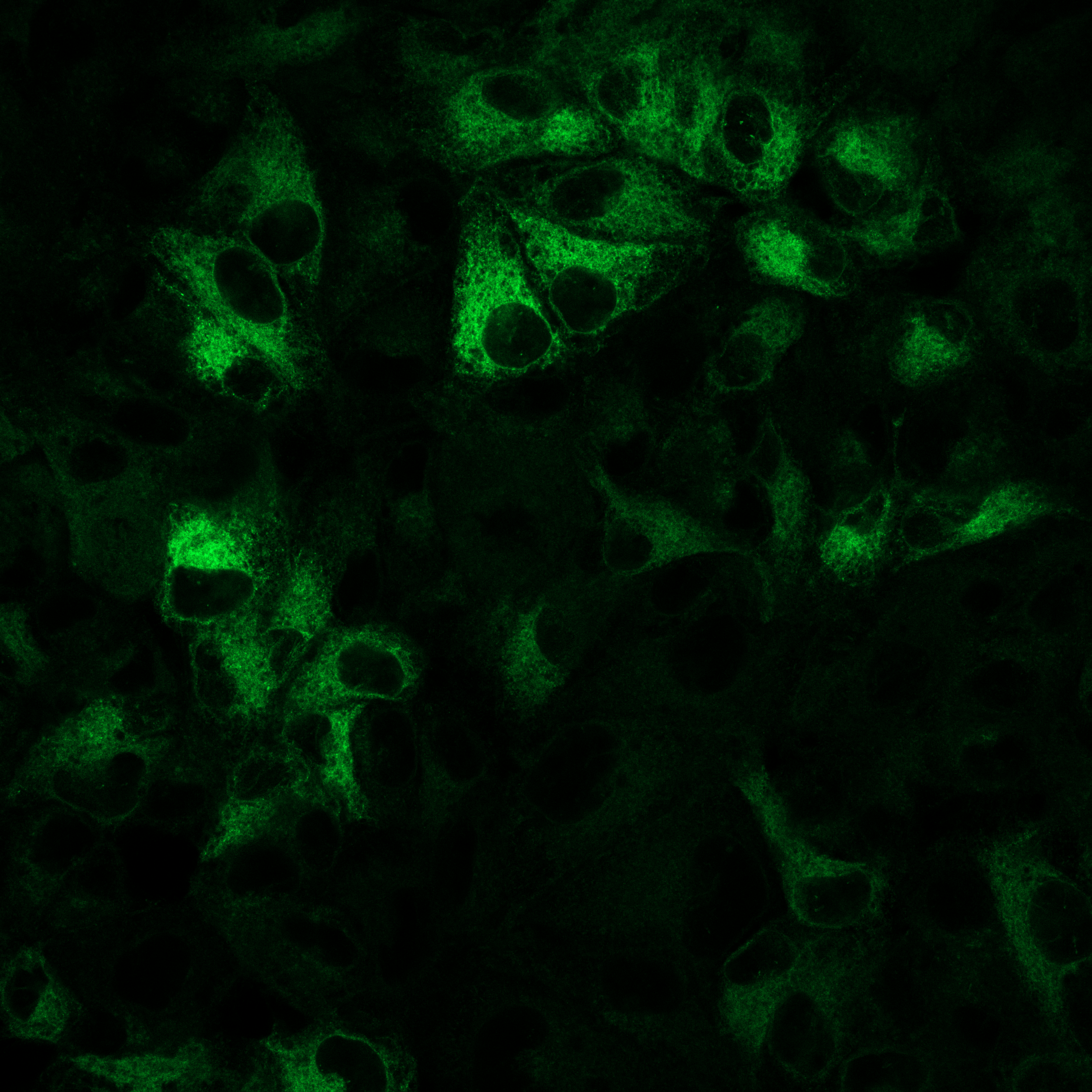

Supplement: Supplementary file 13 — Figure EV3 Replicate Source Data [file 44319_2026_736_MOESM13_ESM.zip › Figure EV3_Replicate/EV3D_Replicate/WT/WT control_NHE3.tif]

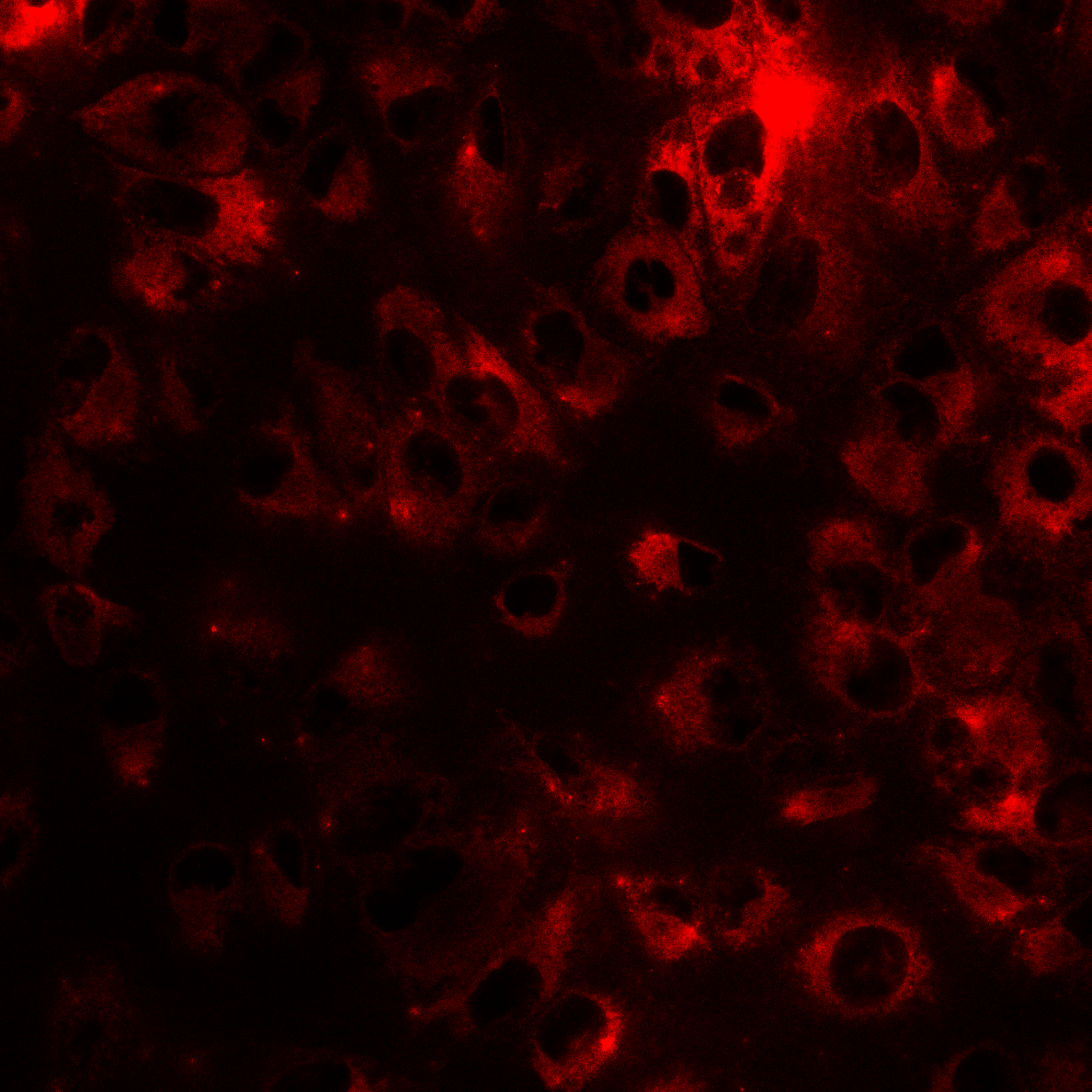

Supplement: Supplementary file 13 — Figure EV3 Replicate Source Data [file 44319_2026_736_MOESM13_ESM.zip › Figure EV3_Replicate/EV3D_Replicate/WT/WT cysteamine_ER Tracker.tif]

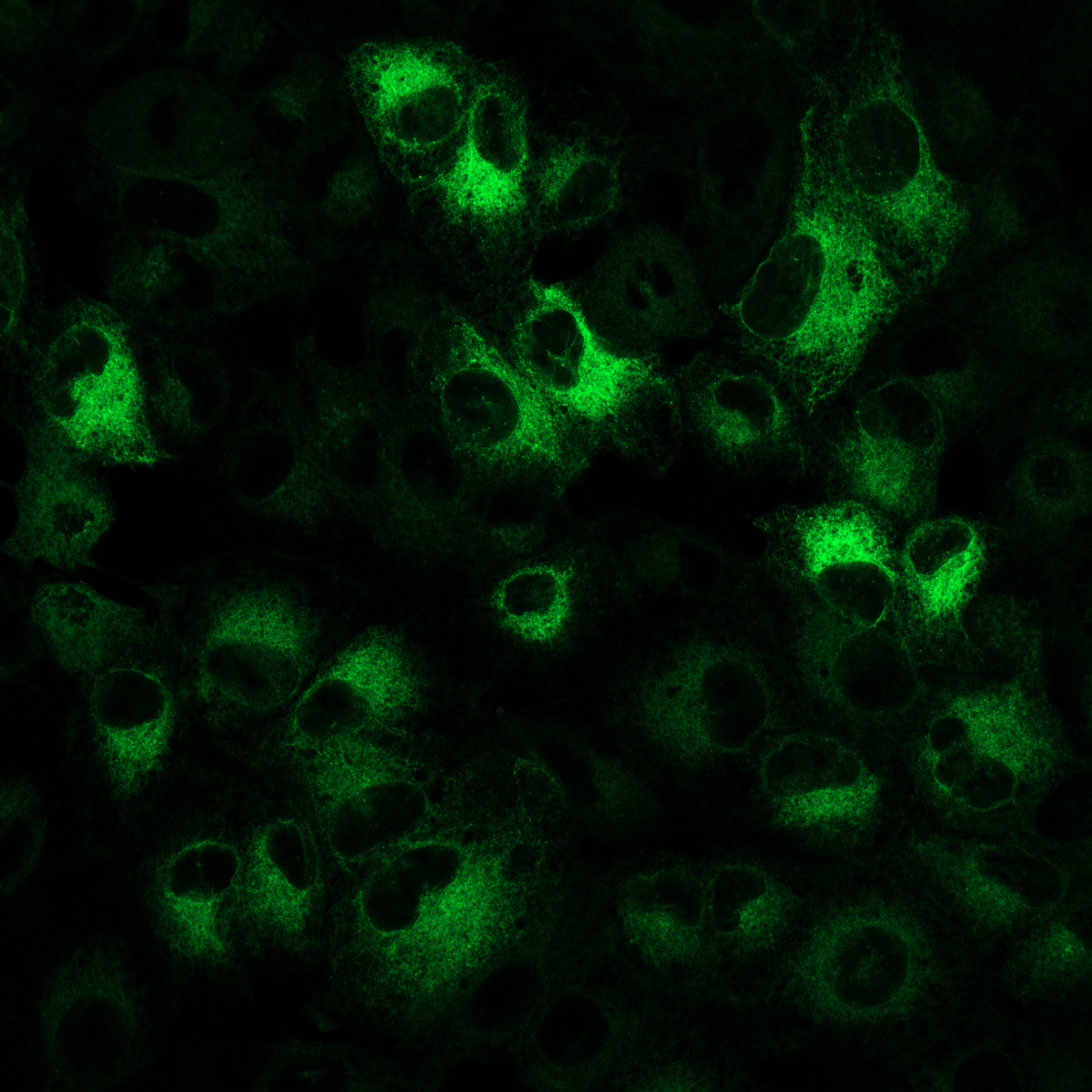

Supplement: Supplementary file 13 — Figure EV3 Replicate Source Data [file 44319_2026_736_MOESM13_ESM.zip › Figure EV3_Replicate/EV3D_Replicate/WT/WT cysteamine_NHE3.tif]

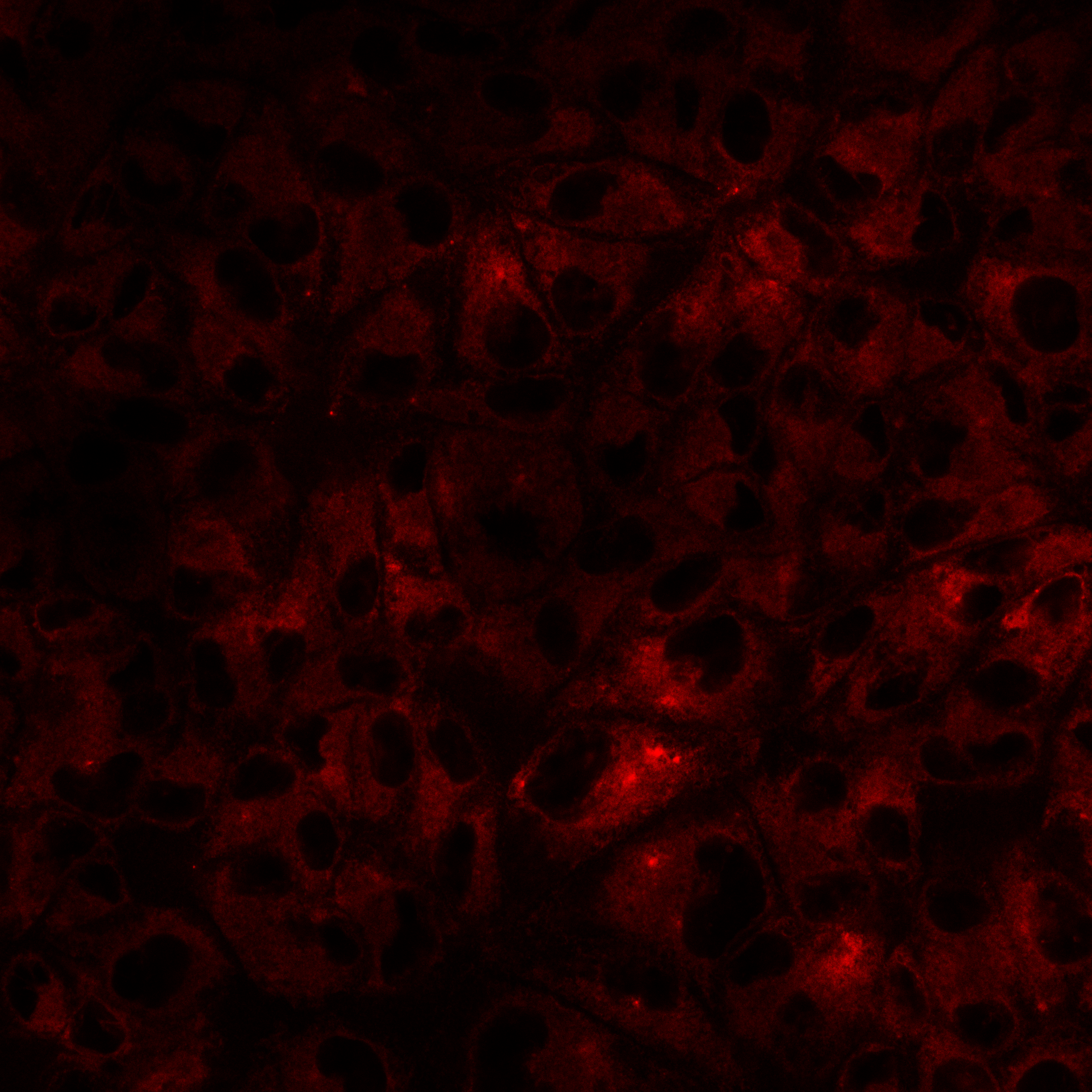

Supplement: Supplementary file 13 — Figure EV3 Replicate Source Data [file 44319_2026_736_MOESM13_ESM.zip › Figure EV3_Replicate/EV3D_Replicate/WT/WT control_ER Tracker.tif]

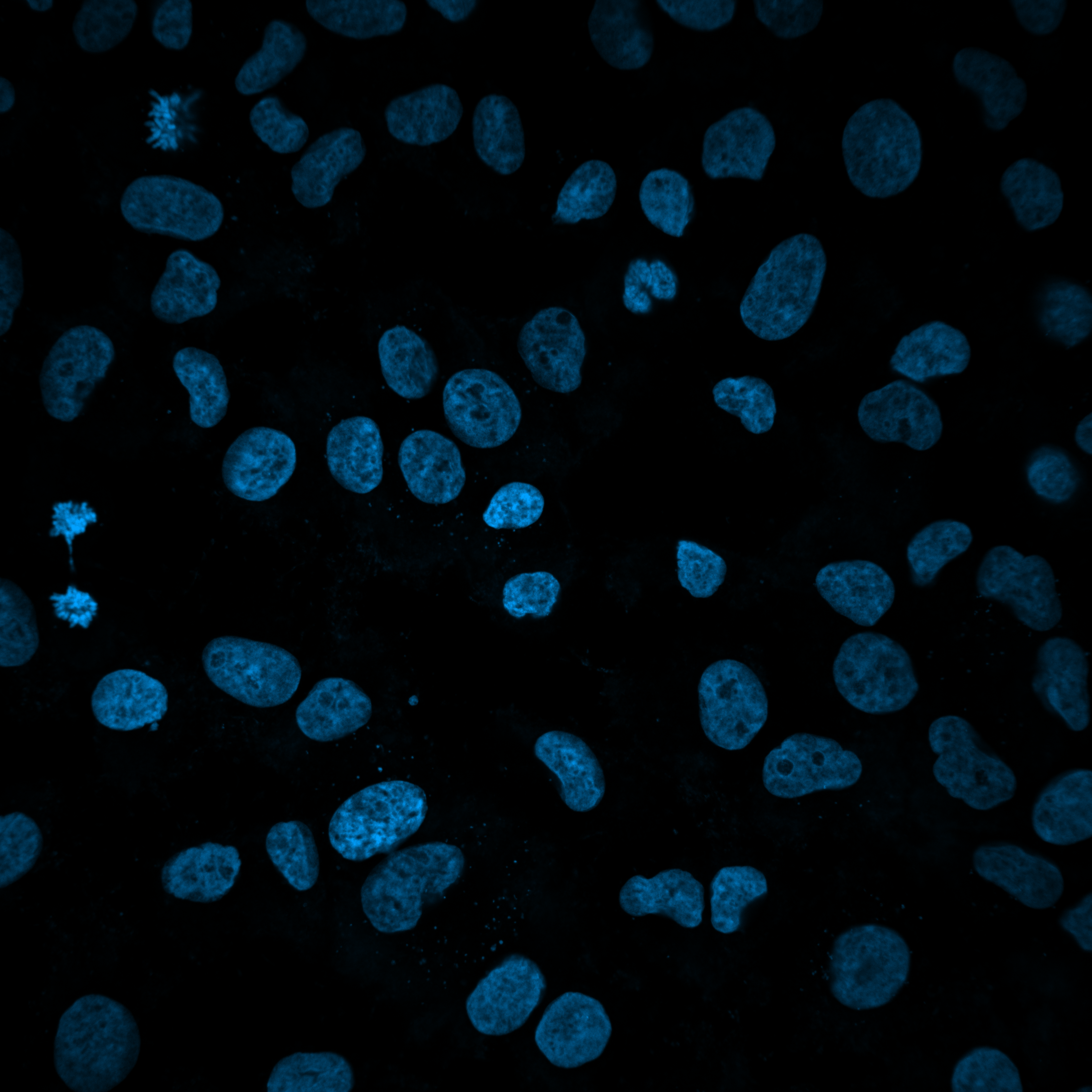

Supplement: Supplementary file 13 — Figure EV3 Replicate Source Data [file 44319_2026_736_MOESM13_ESM.zip › Figure EV3_Replicate/EV3D_Replicate/WT/WT cysteamine_DAPI.tif]

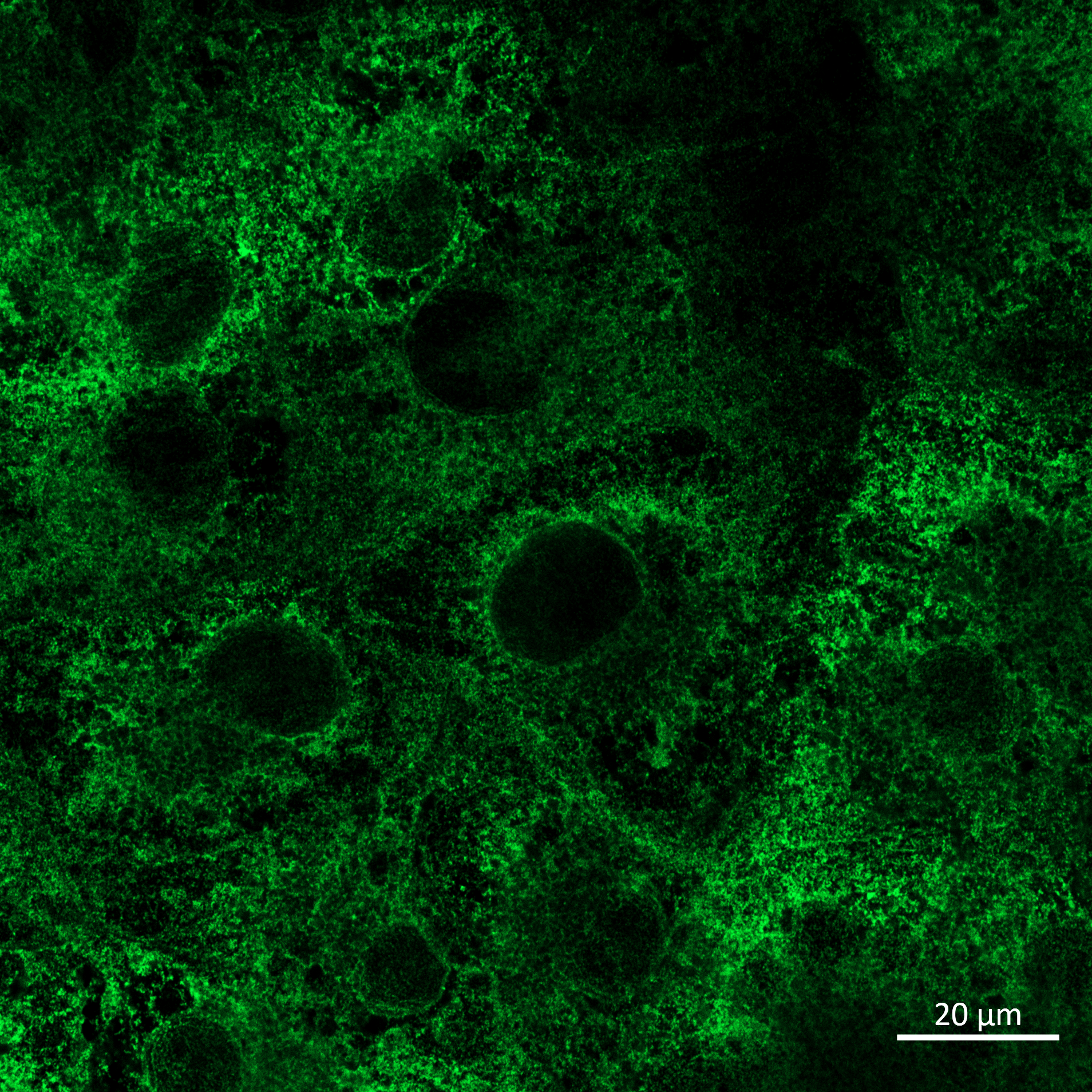

Supplement: Supplementary file 13 — Figure EV3 Replicate Source Data [file 44319_2026_736_MOESM13_ESM.zip › Figure EV3_Replicate/EV3B_Replicate/KO/NHE3-GFP HK-2 CTNS KO_GM130_NHE3 GFP.tif]

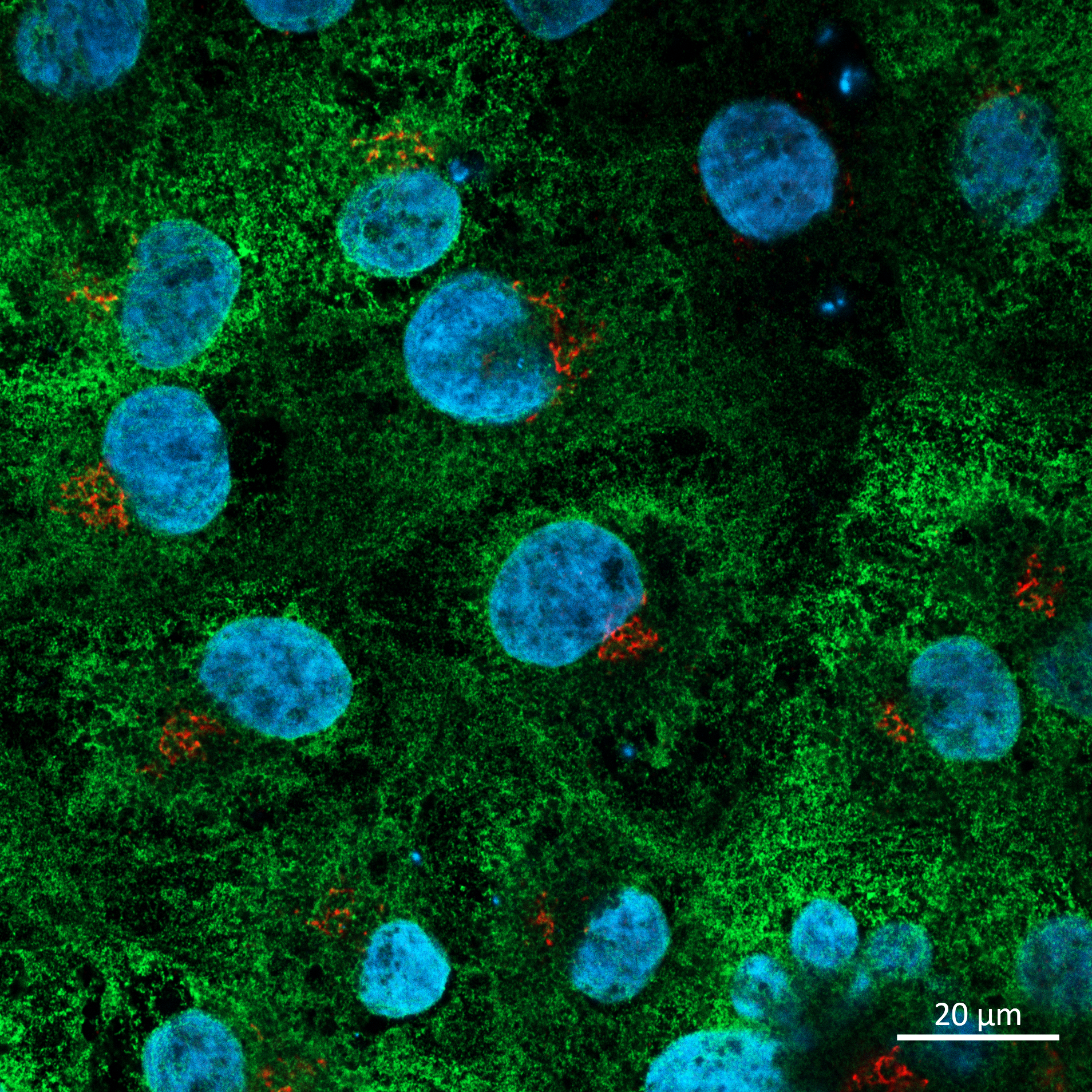

Supplement: Supplementary file 13 — Figure EV3 Replicate Source Data [file 44319_2026_736_MOESM13_ESM.zip › Figure EV3_Replicate/EV3B_Replicate/KO/NHE3-GFP HK-2 CTNS KO_GM130_Merged.tif]

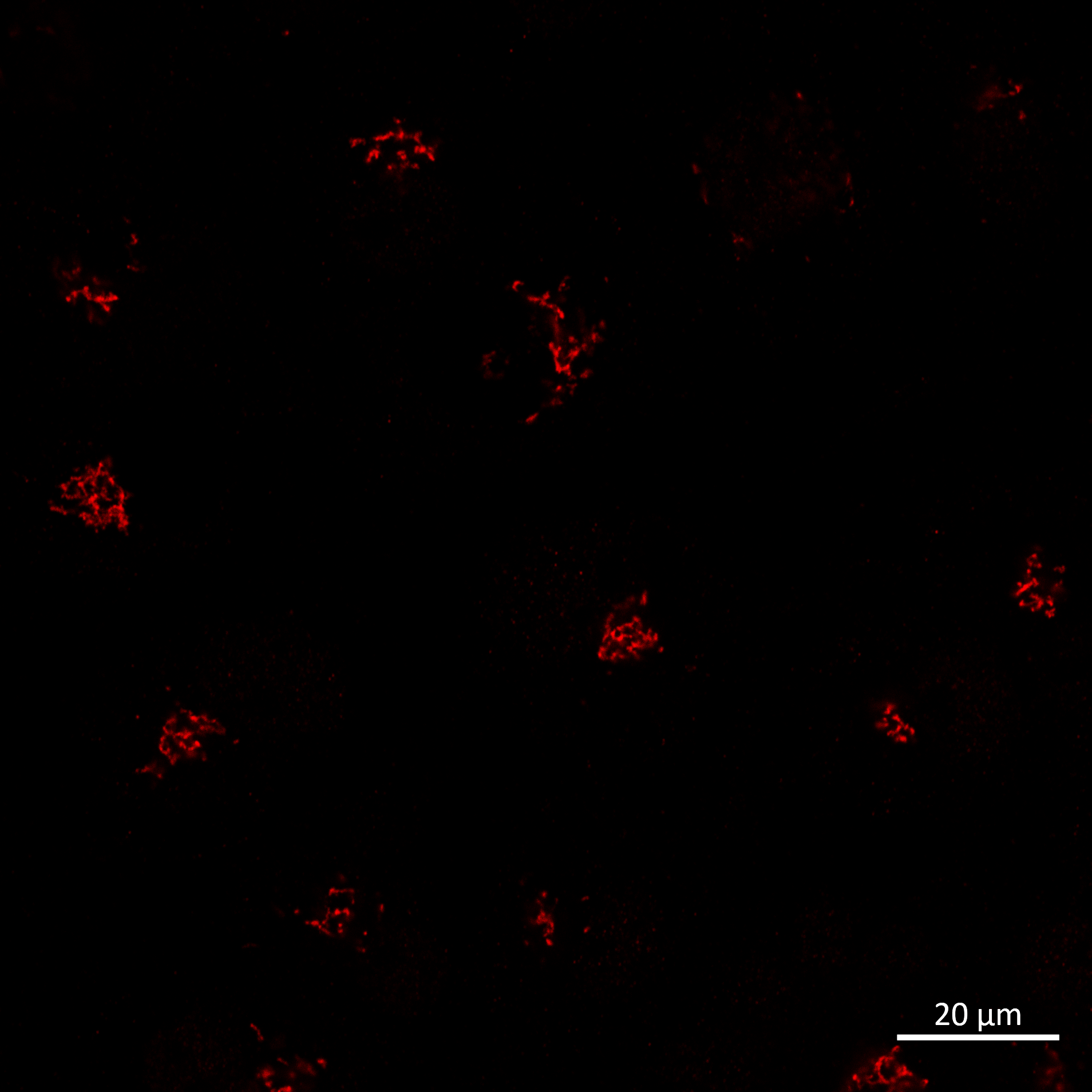

Supplement: Supplementary file 13 — Figure EV3 Replicate Source Data [file 44319_2026_736_MOESM13_ESM.zip › Figure EV3_Replicate/EV3B_Replicate/KO/NHE3-GFP HK-2 CTNS KO_GM130_GM130.tif]

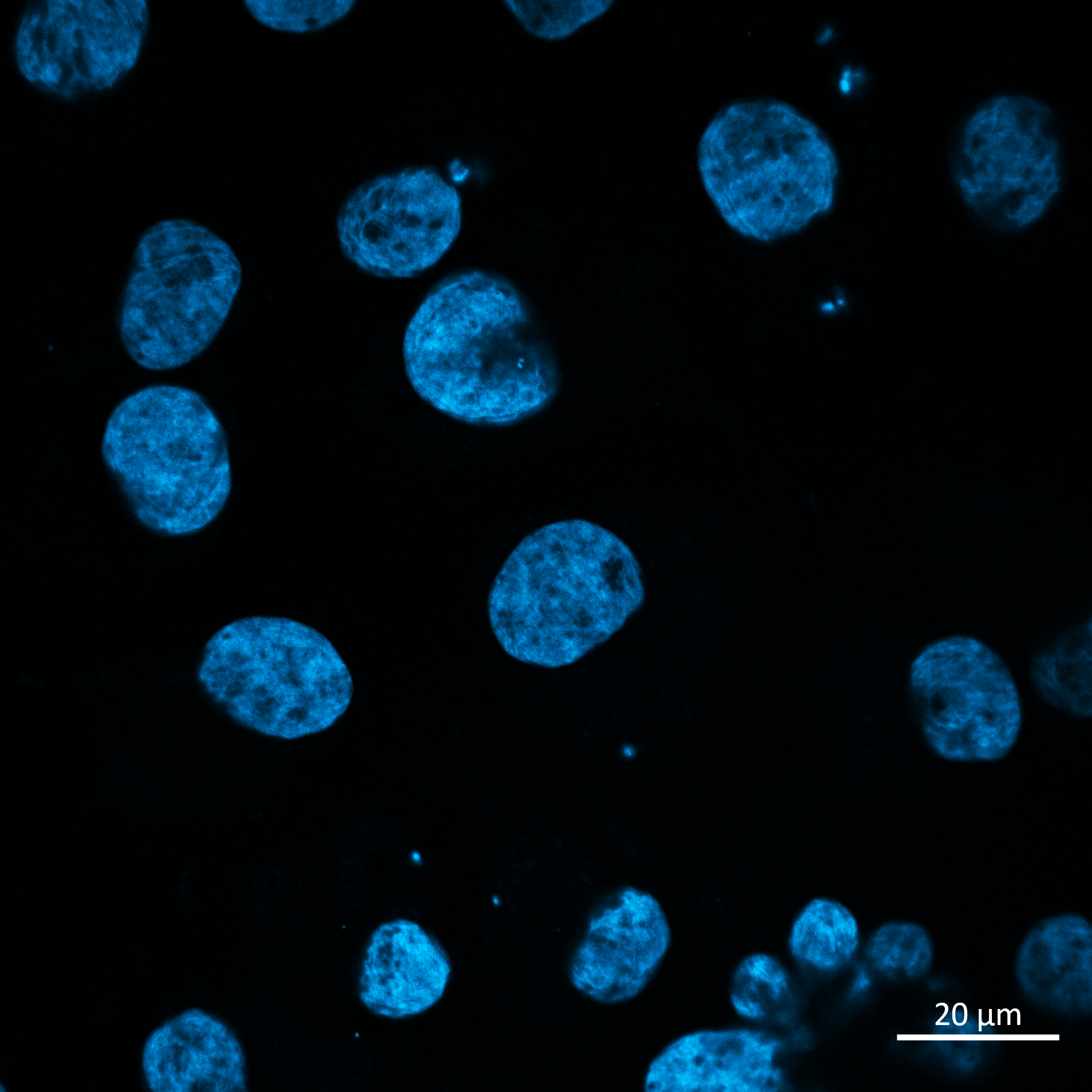

Supplement: Supplementary file 13 — Figure EV3 Replicate Source Data [file 44319_2026_736_MOESM13_ESM.zip › Figure EV3_Replicate/EV3B_Replicate/KO/NHE3-GFP HK-2 CTNS KO_GM130_DAPI.tif]

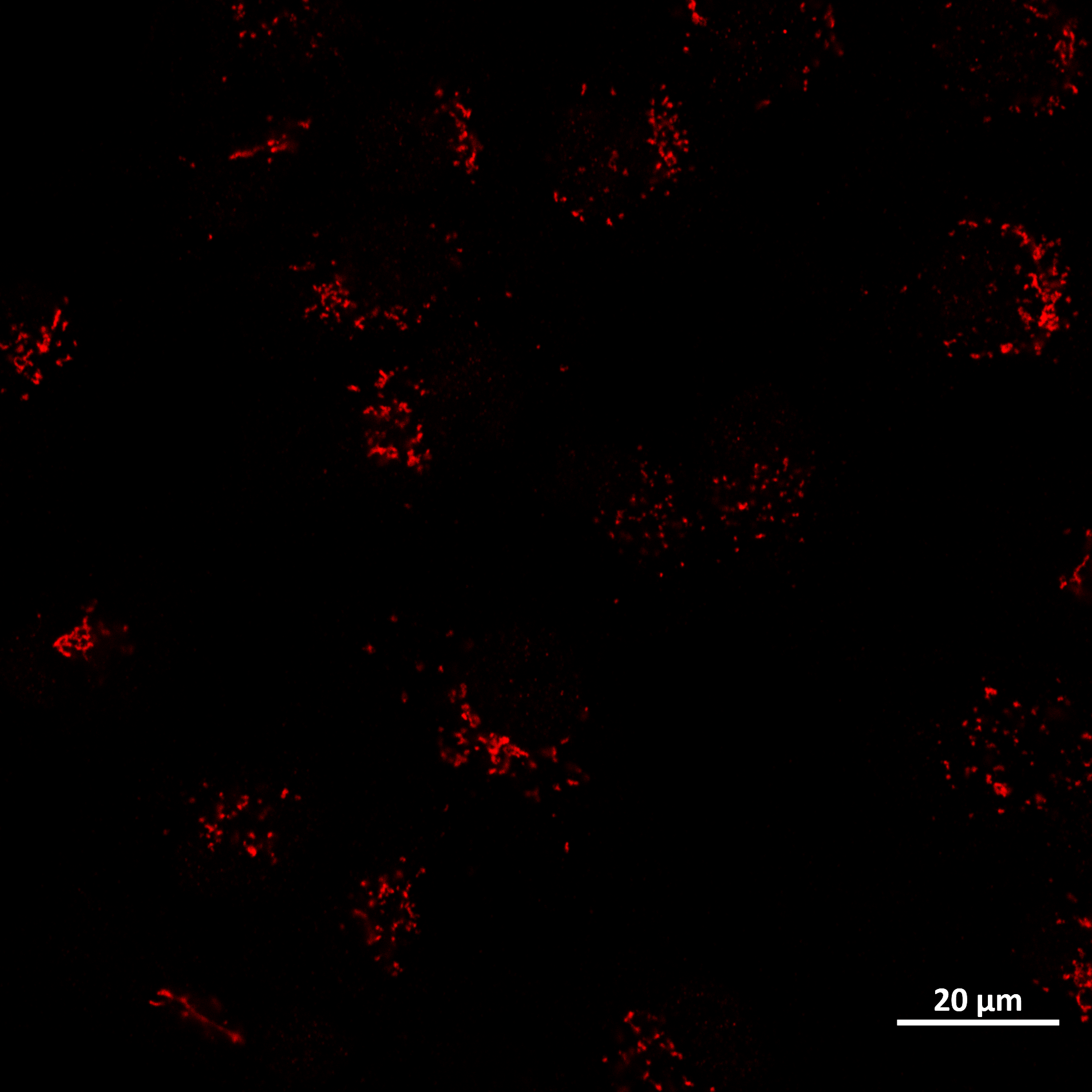

Supplement: Supplementary file 13 — Figure EV3 Replicate Source Data [file 44319_2026_736_MOESM13_ESM.zip › Figure EV3_Replicate/EV3B_Replicate/WT/NHE3-GFP HK-2 WT GM130_GM130.tif]

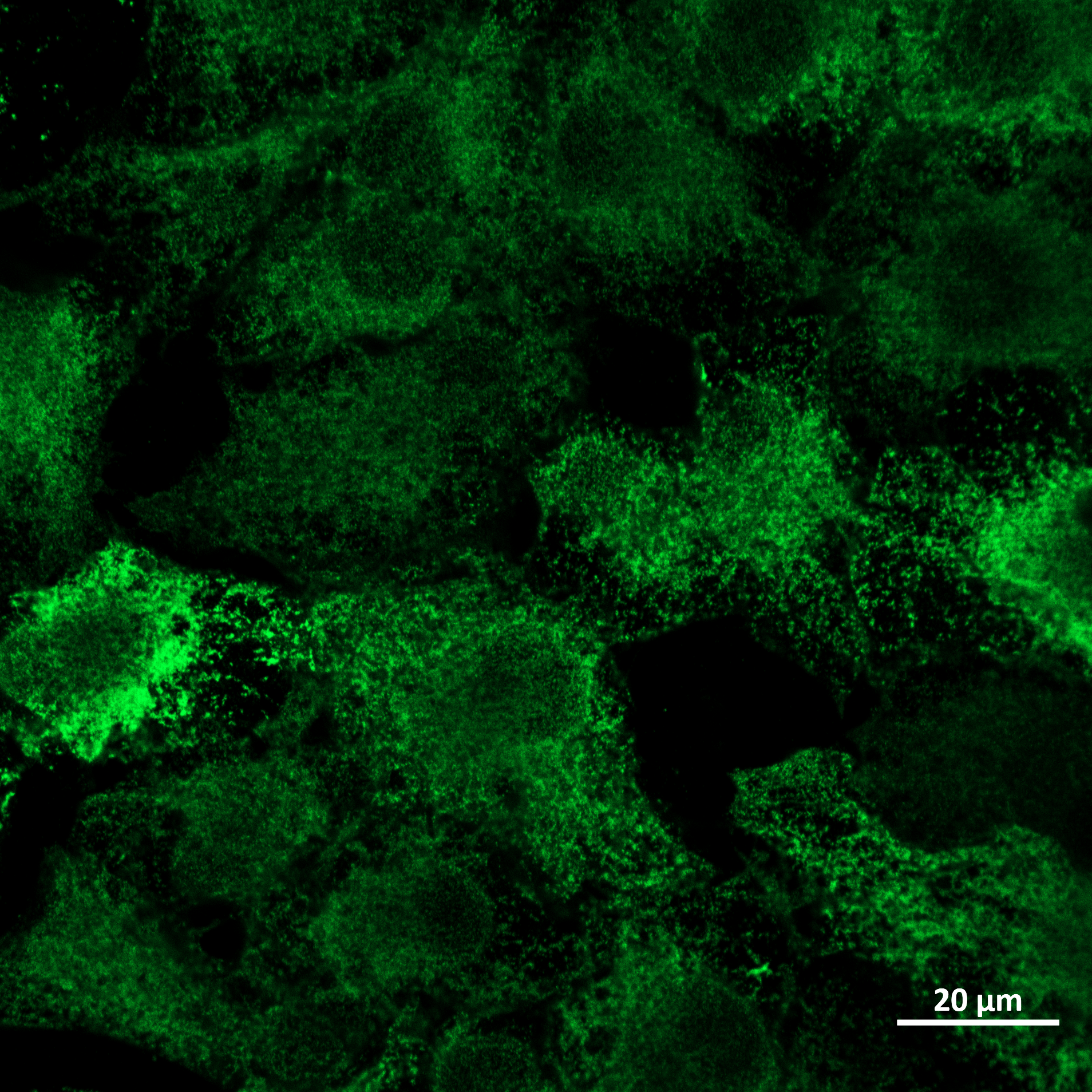

Supplement: Supplementary file 13 — Figure EV3 Replicate Source Data [file 44319_2026_736_MOESM13_ESM.zip › Figure EV3_Replicate/EV3B_Replicate/WT/NHE3-GFP HK-2 WT GM130_NHE3 GFP.tif]

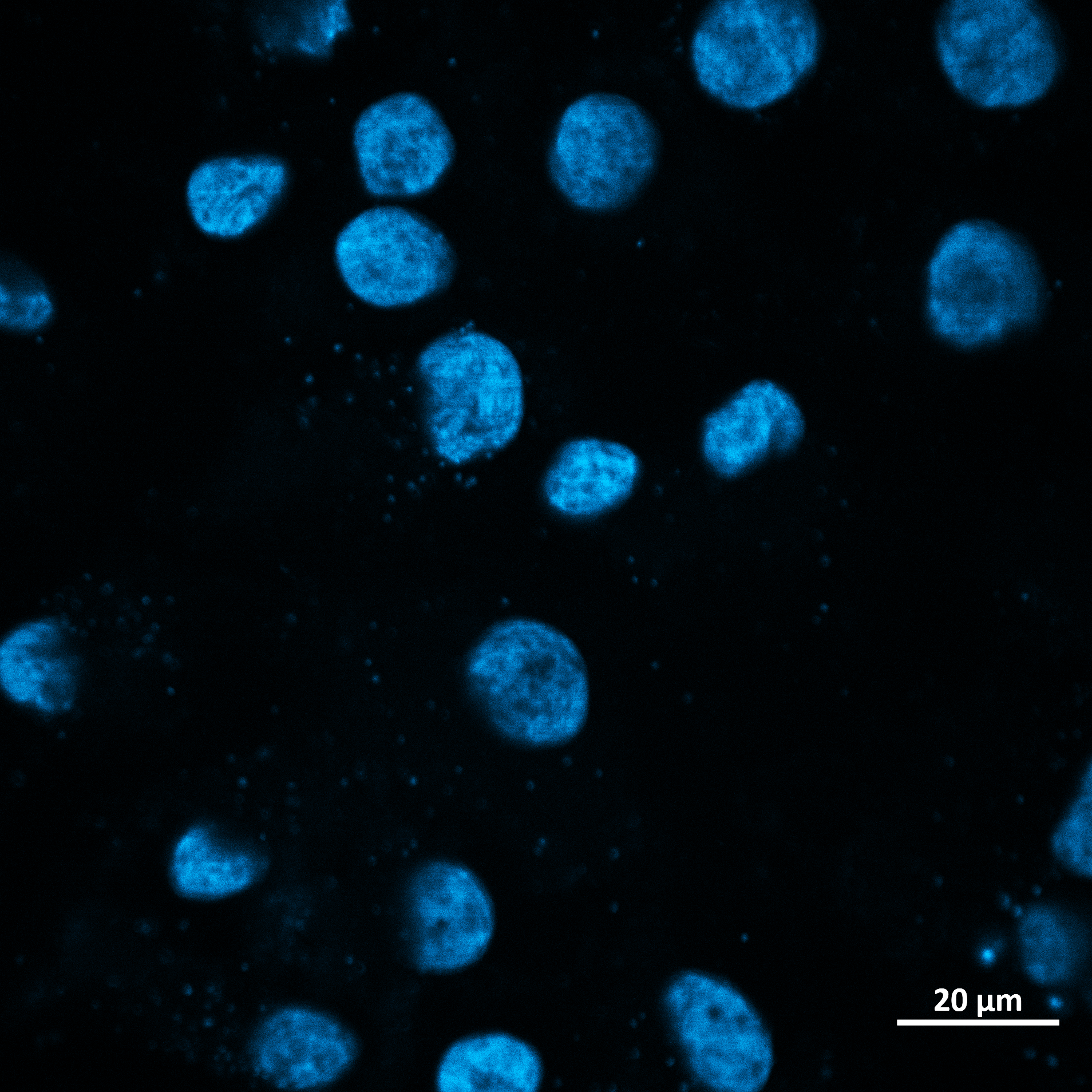

Supplement: Supplementary file 13 — Figure EV3 Replicate Source Data [file 44319_2026_736_MOESM13_ESM.zip › Figure EV3_Replicate/EV3B_Replicate/WT/NHE3-GFP HK-2 WT GM130_DAPI.tif]

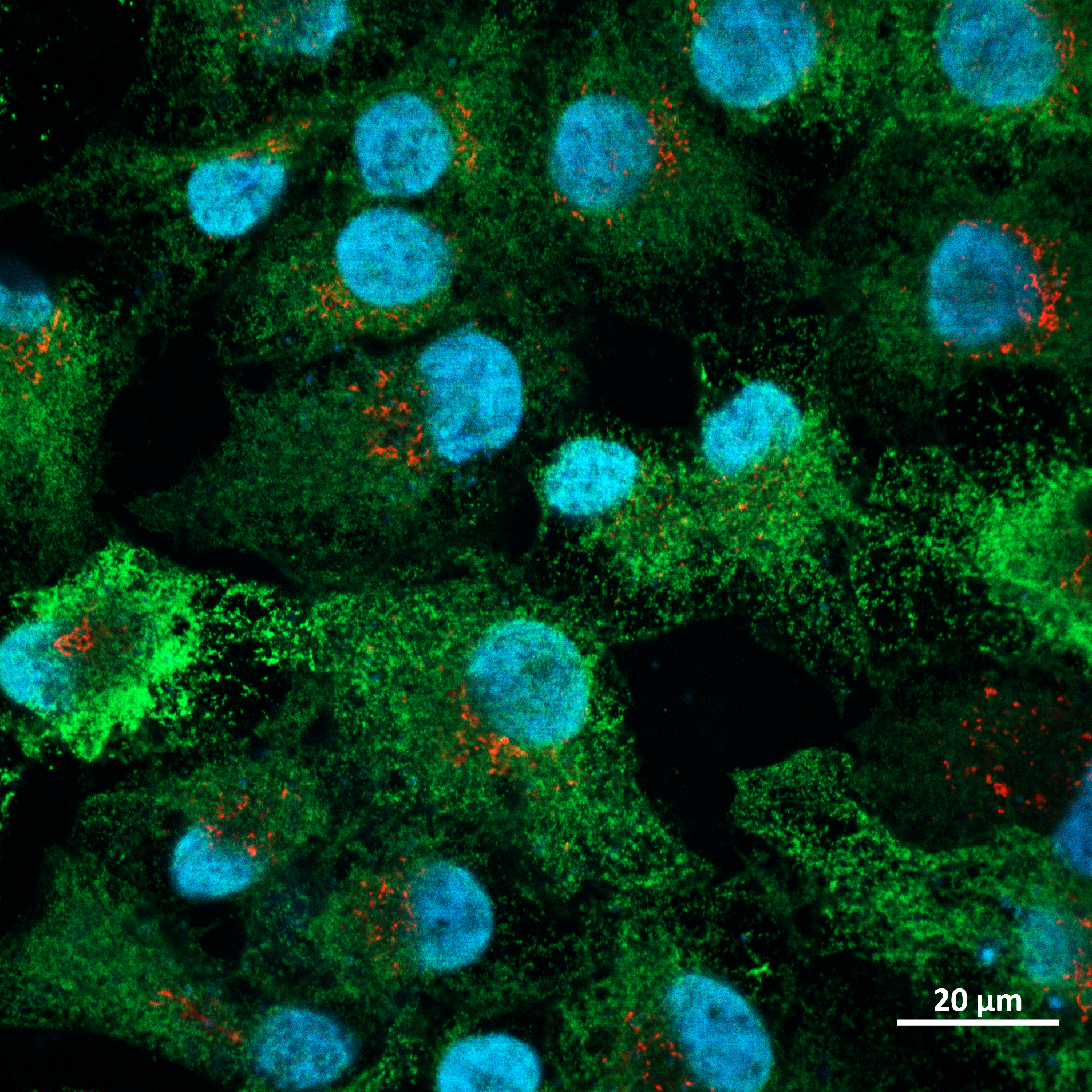

Supplement: Supplementary file 13 — Figure EV3 Replicate Source Data [file 44319_2026_736_MOESM13_ESM.zip › Figure EV3_Replicate/EV3B_Replicate/WT/NHE3-GFP HK-2 WT GM130_Merged.tif]
