# Supplementary material for: Cystinosin regulates Na+/H+ exchanger 3 trafficking and function in kidney proximal tubular cells
Source: EMBO Rep. 2026 Mar 24;27(8):2088–117. doi: 10.1038/s44319-026-00736-1 (PMC13121807; doi:10.1038/s44319-026-00736-1)
Supplement: Supplementary file 14 — Expanded View Figures [file 44319_2026_736_MOESM14_ESM.pdf]

Expanded View Figures

Figure EV1. Cross-species sequence alignment, structural models, interactions/colocalization analysis of Nhx1 and Ers1 and cystine level measurement.

(**A**) Clustal Omega multiple sequence alignment of human cystinosin (Uniprot accession number A0A0S2Z3K3), cystinosinLKG (A0A0S2Z3I9), *P. pastoris* Ers1S (C4R5N7), *P. pastoris* Ers1L (C4R120), and *S. cerevisiae* Ers1 (P1761). Black boxes indicate amino acid identity, gray boxes indicate amino acid similarity, with the red-boxed area highlighting the comparison of the seven-transmembrane domains of cystinosin across the different organisms. We used BoxShade for highlighting (https://junli.netlify.app/apps/boxshade/#forms::boxshade). The multiple sequence alignment was performed using Clustal Omega from EMBL (https://www.ebi.ac.uk/jdispatcher/msa/clustalo). The threshold for comparison is set by default to greater than 50%. (**B**) Displays images of the AlphaFold-predicted structures of the various cystinosin isoforms in each organism described in (**A**). (**C**) BiFC assay performed in *P. pastoris* displaying an interaction between Ers1s and Nhx1, and their colocalization with the early endosome marker, Sec7-DsRed (TGN/EE) (white arrow). Negative controls, the single Venus moieties (Ers1S-VN and Nhx1-VC) do not fluoresce in the green or red channel (yellow arrows). Scale bars: 5 µm. (**D**, **E**) Violin plots of puncta per cell for Nhx1-GFP (**D**) and Vps8-2xmCherry (**E**) in WT, *Δvps1*, *Δvps15*, *Δypt7*, *Δers1S* and *Δers1L*. Each dot is one cell; violins show the distribution. Yellow triangles mark cells that display an Nhx1 vacuolar rim (“ring”) phenotype (continuous or near-continuous Nhx1 signal outlining the vacuole membrane; scored as a binary per-cell attribute). Rim-positive cells still contribute puncta counts as usual; the ring itself is not counted as additional puncta. (**F**) Percentage of Nhx1 puncta that touch a Vps8 punctum in the same cell (Nhx1 on endosome). (**G**) Percentage of Vps8 puncta that touch an Nhx1 punctum (Vps8 with Nhx1). Touching/colocalization was defined operationally on 2D widefield images as overlap or edge-to-edge contact by ≥1 pixel between binary masks after channel alignment; no z-stacks were used. Values are per cell. For (**D–G**) Red squares with error bars = mean ± SEM. Sample sizes (cells): WT $n = 50$, *Δvps15* $n = 50$, *Δvps1* $n = 50$, *Δypt7* $n = 36$, *Δers1S* $n = 50$, *Δers1L* $n = 50$ (≥2 independent experiments). (**H**) Cystine content was measured by mass spectrometry in WT, *Δypt7* yeast strains and in Ers1S and L knockout strains as well as in the double knockout mutants (both Ers1S and L) in both regular glucose culture medium and under starvation conditions. *Δypt7* strain was used as a negative control, as its absence results in low amino acid content during nitrogen starvation due to impaired autophagosome-vacuole fusion. Two-way analysis of variance (ANOVA) was used with comparison done between different cell lines under the same condition. The data in (**H**) represents the mean ± SEM from $n = 3$ biological repeats.

 © The Author(s)

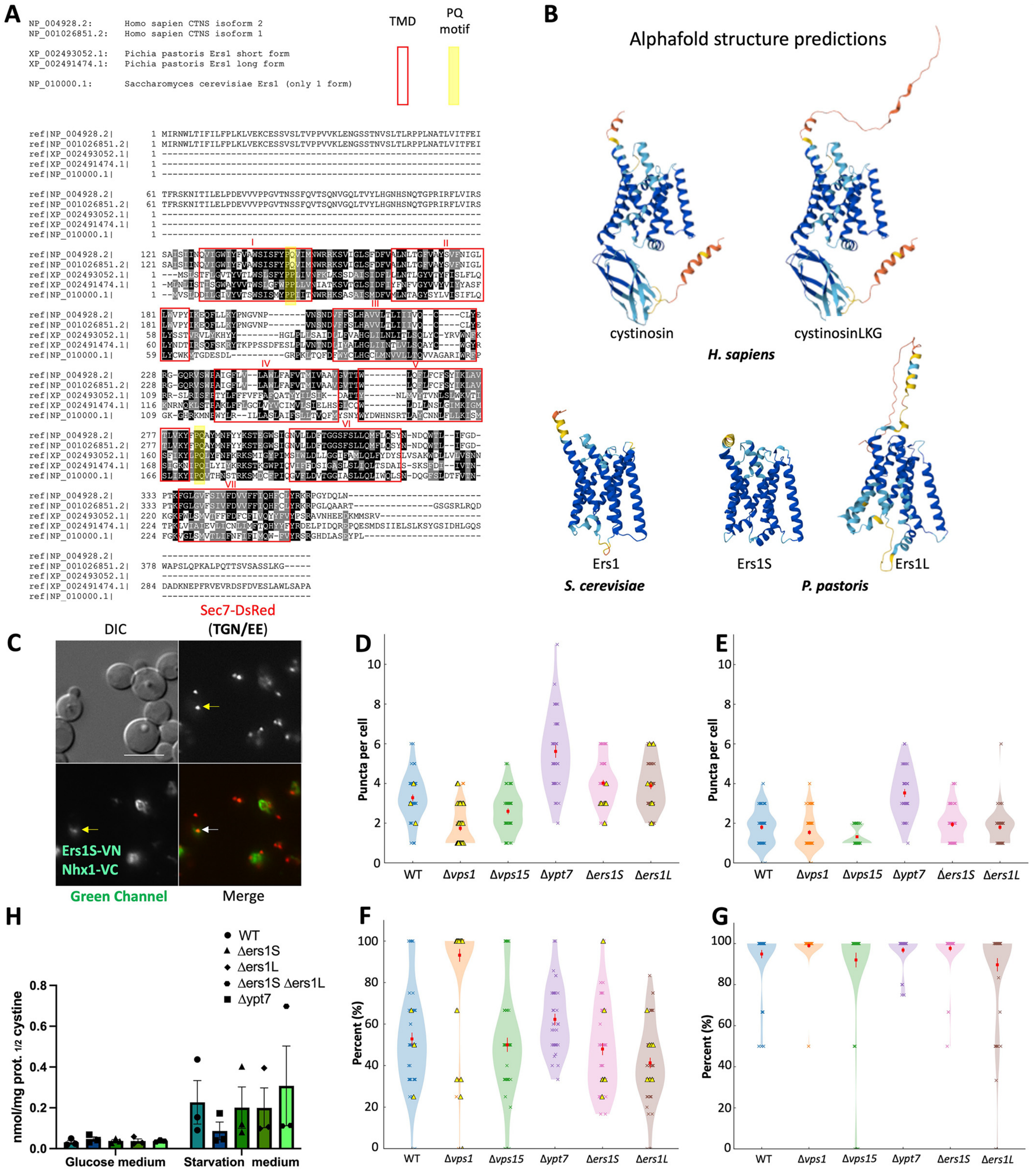
A
NP_004928.2: Homo sapien CTNS isoform 2
NP_001026851.2: Homo sapien CTNS isoform 1
XP_002493052.1: Pichia pastoris Ers1 short form
XP_002491474.1: Pichia pastoris Ers1 long form
NP_010000.1: Saccharomyces cerevisiae Ers1 (only 1 form)
TMD
PQ motif
B
Alphafold structure predictions
cystinosin
cystinosinLKG
H. sapiens
Ers1
S. cerevisiae
Ers1S
Ers1L
P. pastoris
C
DIC
Sec7-DsRed (TGN/EE)
Ers1S-VN
Nhx1-VC
Green Channel
Merge
D
E
Puncta per cell
WT
Δvps1
Δvps15
Δypt7
Δers1S
Δers1L
F
G
Percent (%)
H
WT
Δers1S
Δers1L
Δers1S Δers1L
Δypt7
nmol/mg prot. 1/2 cystine
Glucose medium
Starvation medium

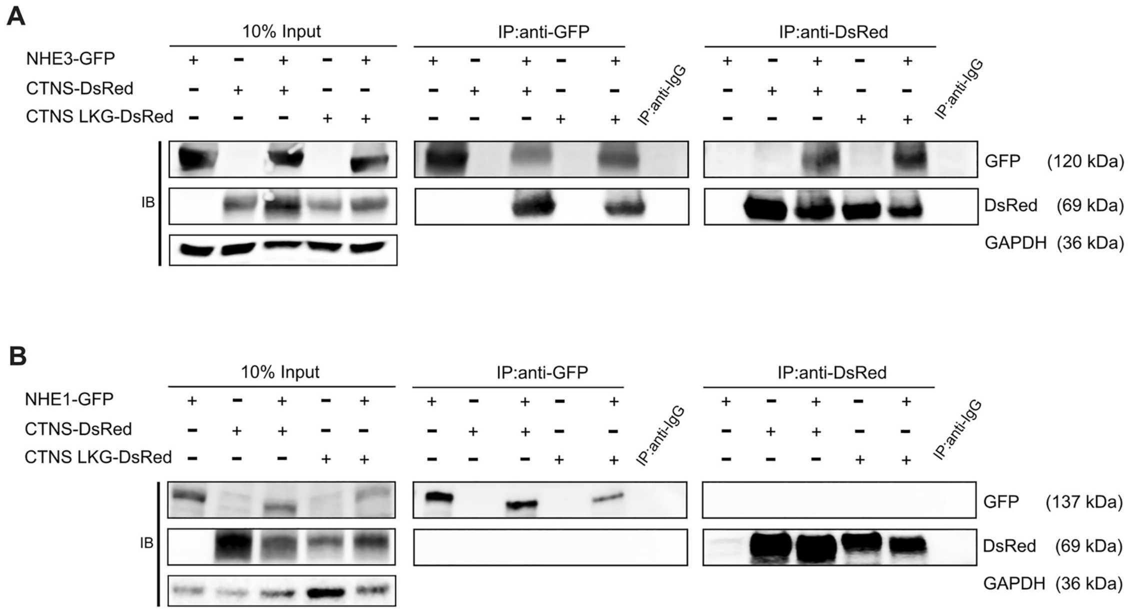

Figure EV2. Cystinosin interacts with NHE3 but not with NHE1.

Immunoprecipitation assay performed in (**A**) CTNS-deficient HK-2 and (**B**) HEK293T cells stably expressing NHE3 and NHE1, respectively, along with cystinosin and cystinosinLKG showing interaction with NHE3 (**A**) and no interaction with NHE1 (**B**) with both cystinosin and cystinosinLKG. Input lysates show proper expression of the proteins with glyceraldehyde-3-phosphate dehydrogenase (GAPDH) used as a loading control. Pull down with anti-IgG was used as a negative control.

 © The Author(s)

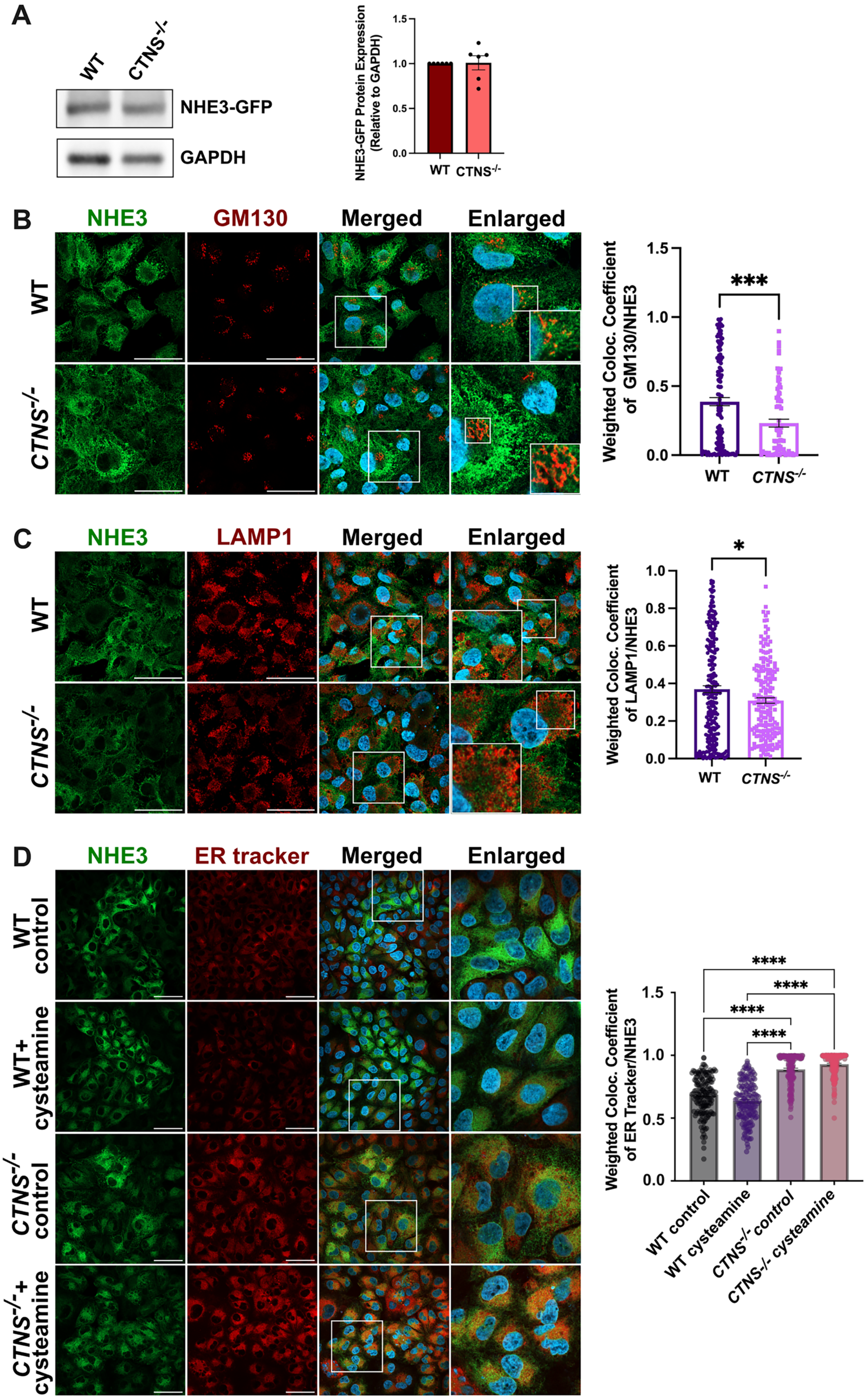
A
WT
CTNS-/-
NHE3-GFP
GAPDH
NHE3-GFP Protein Expression (Relative to GAPDH)
WT
CTNS-/-
B
NHE3
GM130
Merged
Enlarged
WT
CTNS-/-
Weighted Coloc. Coefficient of GM130/NHE3
***
C
NHE3
LAMP1
Merged
Enlarged
Weighted Coloc. Coefficient of LAMP1/NHE3
*
D
NHE3
ER tracker
Merged
Enlarged
WT control
WT+ cysteamine
CTNS-/- control
CTNS-/-+ cysteamine
Weighted Coloc. Coefficient of ER Tracker/NHE3
****
WT control
WT cysteamine
CTNS-/- control
CTNS-/- cysteamine

Figure EV3. Cystinosin deficiency impairs NHE3 trafficking in human WT and *CTNS*$^{-/-}$ HK2 proximal tubular cells.

(**A**) Representative Western blot analysis of NHE3-GFP expression with GAPDH used as a loading control ($n = 6$ biological repeats). (**B**, **C**) Representative immunofluorescence images of NHE3-GFP (green) with markers of Golgi (GM130) (red) (**B**) and lysosomes (LAMP1) (red) (**C**) with their corresponding quantification showing defect in NHE3 subcellular localization in *CTNS*$^{-/-}$ HK2 cells. (**D**) Representative immunofluorescence images of NHE3-GFP (green) in WT and *CTNS*$^{-/-}$ HK2 cells, with or without cysteamine treatment along with ER tracker with their corresponding quantification. Scale bars: 50 µm (**B–D**). Bar graphs are presented as the mean ± SEM. For (**B**) $P = 0.0006$; (**C**) $P = 0.0124$. *$P < 0.05$; ***$P < 0.001$ using two-tailed Student's t test for Figures (**B**, **C**) and One-way ANOVA was done for (**D**); ****$P < 0.0001$. For (**B–D**), $n = 3$ biological repeats. Source data are available online for this figure.

 © The Author(s)

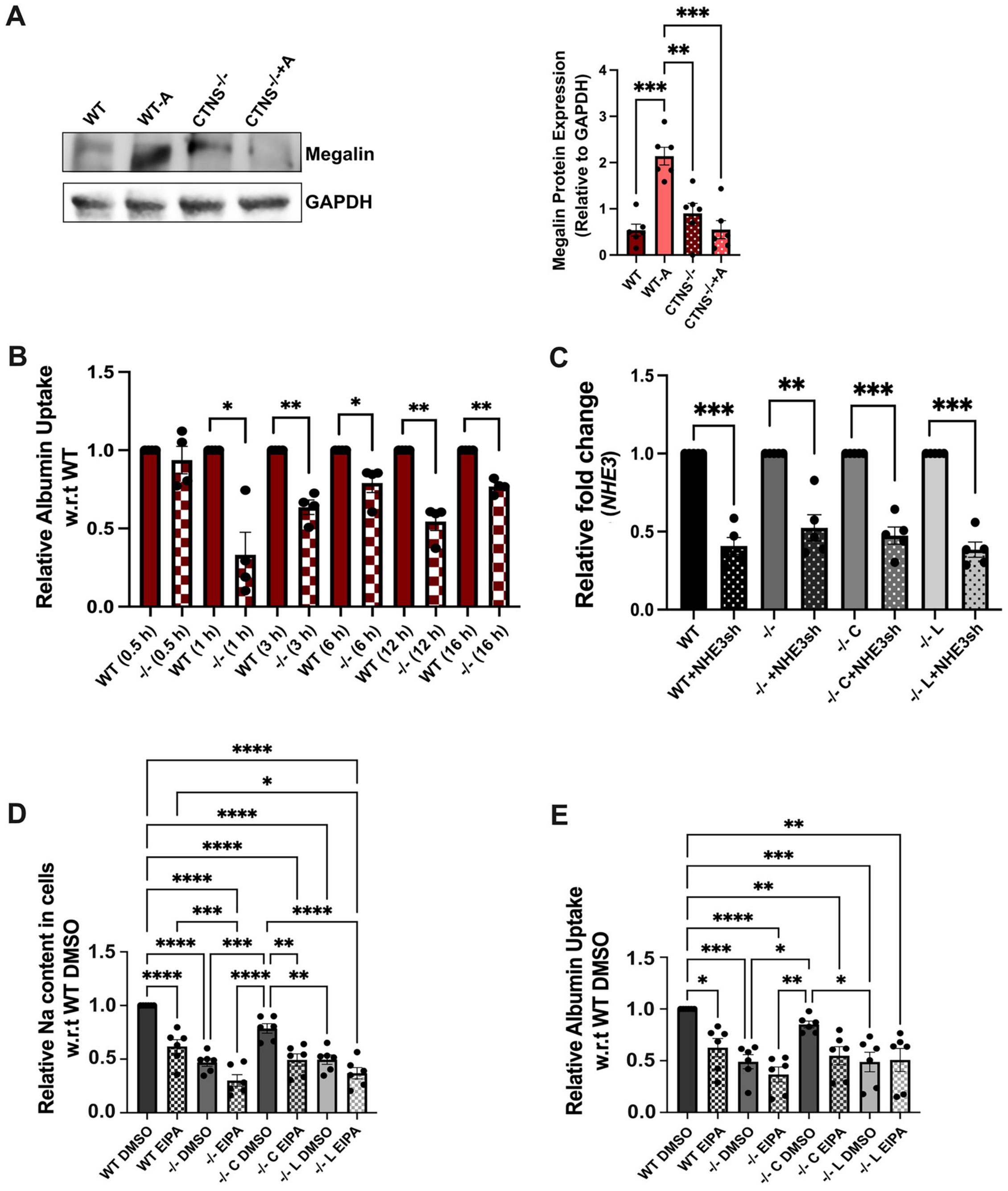

Figure EV4. Cystinosin deficiency impairs NHE3 function in human WT and *CTNS*$^{-/-}$ HK2 proximal tubular cells.

(**A**) Representative Western blot analysis of Megalin expression, with or without Albumin555 treatment for 16 h; GAPDH is used as a loading control ($n = 6$). (**B**) Albumin uptake studies in WT and *CTNS*$^{-/-}$ HK2 cells at different timepoints between 0.5 and 16 h measured using albumin555 by flow cytometry ($n = 4$). (**C**) *NHE3* mRNA expression measured with quantitative PCR in cells treated with or without NHE3sh RNA confirming NHE3 knockdown ($n = 5$). (**D**, **E**) Na^+ and albumin uptake in WT and *CTNS*$^{-/-}$ HK2 cells (−/−), and in *CTNS*$^{-/-}$ HK2 cells transduced with LV-CTNS (C) or LV-CTNS-LKG (L) treated with DMSO (vehicle), or the sodium transporter inhibitor (EIPA) ($n = 6$). For data in figure (**A–E**) each dot represents an independent biological replicate. Bar graphs are presented as the mean ± SEM. Two-tailed Student's t test was used for (**B**, **C**). One-way ANOVA was done for (**A**, **D**, **E**). For (**A**), WT vs. WT-A, $P = 0.0005$; WT-A vs. *CTNS*$^{-/-}$, $P = 0.0049$; WT-A vs. *CTNS*$^{-/-}$*-A*, $P = 0.0005$. For (**B**), WT vs. *CTNS*$^{-/-}$, $P = 0.0185$, $P = 0.0043$, $P = 0.0423$, $P = 0.0041$, $P = 0.0020$, for 1, 3, 6, 12 and 16 h, respectively. For (**C**), $P = 0.0004$, $P = 0.0048$, $P = 0.0007$, $P = 0.0002$, for WT vs. WT+NHE3sh, −/− vs. −/− + NHE3sh, −/− C vs. −/− C + NHE3sh and −/− L vs. −/− L+ NHE3sh, respectively. For (**D**), $P = 0.0008$, $P = 0.0025$, $P = 0.0021$, $P = 0.0007$, $P = 0.0135$, for −/− DMSO vs. −/− C DMSO, −/− C DMSO vs. −/− L DMSO, −/− C DMSO vs. −/− C EIPA, WT EIPA vs. −/− EIPA and WT EIPA vs. −/− L EIPA, respectively. For (**E**), $P = 0.0008$, $P = 0.0008$, $P = 0.0283$, $P = 0.0041$, $P = 0.0013$, $P = 0.0387$, $P = 0.0369$ and $P = 0.0017$ for WT DMSO vs. −/− DMSO, WT DMSO vs. −/− L DMSO, WT DMSO vs. WT EIPA, WT DMSO vs. −/− C EIPA, WT DMSO vs. −/− L EIPA, −/− DMSO vs. −/− C DMSO, −/− C DMSO vs. −/− L DMSO and −/− C DMSO vs. −/− EIPA, respectively. *$P < 0.05$; **$P < 0.01$; ***$P < 0.001$; ****$P < 0.0001$.

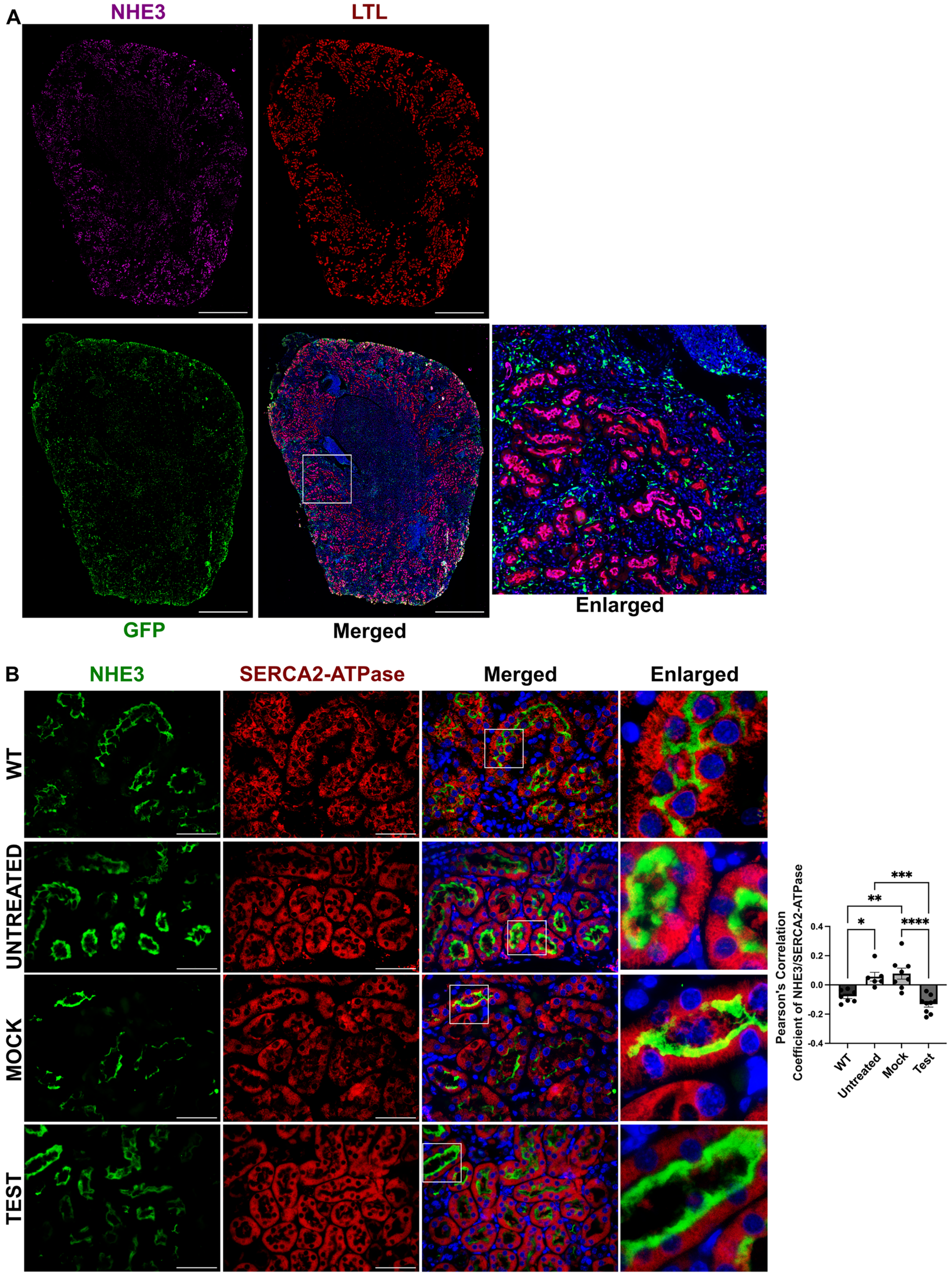
A
NHE3
LTL
GFP
Merged
Enlarged
B
NHE3
SERCA2-ATPase
Merged
Enlarged
WT
UNTREATED
MOCK
TEST
Pearson's Correlation Coefficient of NHE3/SERCA2-ATPase
0.4
0.2
0.0
-0.2
-0.4
WT
Untreated
Mock
Test
*
**
***
****

 © The Author(s)

Figure EV5. GFP^{+} WT HSPC distribution in the kidney following WT HSPC transplantation and colocalization analysis with an ER marker.

(**A**) Overview of immunofluorescence images of entire kidney section from the Test mice stained with anti-GFP (green), anti-NHE3 (magenta) antibodies and anti-LTL (red) conjugate. This image shows the distribution of the GFP^{+} WT HSPC-derived cells as well as the colocalization of LTL and NHE3 within the kidney of *Ctns*$^{-/-}$ mice transplanted with WT HSPCs. (**B**) Representative immunofluorescence images of formalin fixed paraffin embedded (FFPE) kidney sections from WT, Ctns$^{-/-}$, Mock and Test mice, stained with anti-NHE3 (green) and the ER marker anti-SERCA2 (red) antibodies, along with DAPI (nuclei; blue). Corresponding colocalization quantification between NHE3 and SERCA2 was performed using Pearson Correlation Coefficient. The data are presented as mean ± SEM (WT $n = 7$, Untreated $n = 6$, Mock $n = 8$, Test $n = 9$). Each dot represents an individual mouse. Scale bars: 4 mm (**A**); 50 µm (**B**). One-way analysis of variance (ANOVA) was used, WT vs. Untreated, $P = 0.0170$; WT vs. Mock, $P = 0.0025$; Untreated vs. Test, $P = 0.0003$. *$P < 0.05$; **$P < 0.01$; ***$P < 0.001$; ****$P < 0.0001$.

© The Author(s)

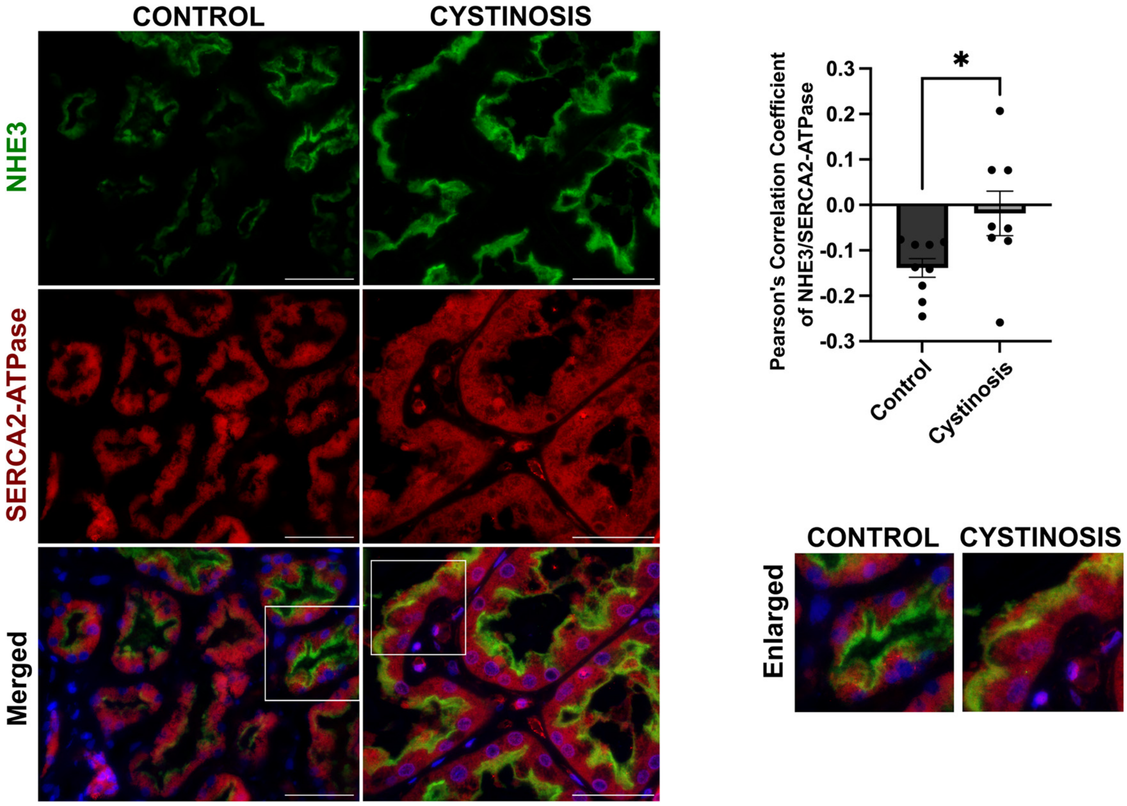

Figure EV6. NHE3 localization study with an ER marker in human control donor and cystinosis patient kidney tissue sample.

Representative immunofluorescence images of formalin fixed paraffin embedded (FFPE) human kidney sections from a control donor and a cystinosis patient. Sections were stained with anti-NHE3 (green), anti-SERCA2 (red) antibodies and DAPI (nuclei). Scale bars: 50 µm. The quantitative data of colocalization between NHE3 and SERCA2 was determined by Pearson Correlation Coefficient. The data is presented as mean ± SEM from $n = 3$ biological repeats. Two-tailed Student's t-test was used; $P = 0.0495$; ($^*P < 0.05$).

 © The Author(s)
